# Predicting Gene Expression from Sequence: A Reexamination

**DOI:** 10.1371/journal.pcbi.0030243

**Published:** 2007-11-30

**Authors:** Yuan Yuan, Lei Guo, Lei Shen, Jun S Liu

**Affiliations:** Department of Statistics, Harvard University, Cambridge, Massachusetts, United States of America; University of California San Diego, United States of America

## Abstract

Although much of the information regarding genes' expressions is encoded in the genome, deciphering such information has been very challenging. We reexamined Beer and Tavazoie's (BT) approach to predict mRNA expression patterns of 2,587 genes in Saccharomyces cerevisiae from the information in their respective promoter sequences. Instead of fitting complex Bayesian network models, we trained naïve Bayes classifiers using only the sequence-motif matching scores provided by BT. Our simple models correctly predict expression patterns for 79% of the genes, based on the same criterion and the same cross-validation (CV) procedure as BT, which compares favorably to the 73% accuracy of BT. The fact that our approach did not use position and orientation information of the predicted binding sites but achieved a higher prediction accuracy, motivated us to investigate a few biological predictions made by BT. We found that some of their predictions, especially those related to motif orientations and positions, are at best circumstantial. For example, the combinatorial rules suggested by BT for the PAC and RRPE motifs are not unique to the cluster of genes from which the predictive model was inferred, and there are simpler rules that are statistically more significant than BT's ones. We also show that CV procedure used by BT to estimate their method's prediction accuracy is inappropriate and may have overestimated the prediction accuracy by about 10%.

## Author Summary

Through binding to certain sequence-specific sites upstream of the target genes, a special class of proteins called transcription factors (TFs) control transcription activities, i.e., expression amounts, of the downstream genes. The DNA sequence patterns bound by TFs are called motifs. It has been shown in an article by Beer and Tavazoie (BT) published in *Cell* in 2004 that a gene's expression pattern can be well-predicted based only on its upstream sequence information in the form of matching scores of a set of sequence motifs and the location and orientation of corresponding predicted binding sites. Here we report a new naïve Bayes method for such a prediction task. Compared to BT's work, our model is simpler, more robust, and achieves a higher prediction accuracy using only the motif matching score. In our method, the location and orientation information do not further help the prediction in a global way. Our result also casts doubt on several biological hypotheses generated by BT based on their model. Finally, we show that the cross-validation procedure used by BT to estimate their method's prediction accuracy is inappropriate and may have overestimated the accuracy by about 10%.

## Introduction

Developing computational strategies for predicting transcription factor binding sites (TFBSs) and transcription regulatory networks has been a central problem in computational biology for more than a decade. Reviews on this problem and various proposed methods can be found in [1–3]. A popular strategy is to search from upstream sequences of a set of co-regulated genes for over-represented (i.e., enriched) sequence features (motifs) [4–7]. With the help of gene expression microarray technology, the expression level of thousands of genes can be measured at the same time [8–10], which makes the discovery of sets of co-regulated genes and their respective regulatory signals at the genome-wide level a reality for many species.

Bussemaker et al. [11] pioneered the use of regression models to relate a gene's expression with numbers of occurrences of certain *k*-mer “words” in the upstream sequence of this gene. Motivated by their work, researchers have developed various methods to extract features that are predictive of gene expression levels. Keles et al. [12,13] tackled the problem using logic regression, which treats motif occurrences as binary covariates and selects important predictors adaptively. Conlon et al. [14] proposed a stepwise regression procedure called Motif Regressor, which uses motif matching scores at promoter regions instead of *k*-mer occurrences as covariates. Zhong et al. [15] extended these methods by introducing a more flexible regression model with an unspecified nonlinear link function. Das et al. [16] implemented a smoothing-spline regression in the place of the linear regression used by Motif Regressor. Further along this general direction, Segal et al. [17] showed that DNA sequence and gene expression information can be combined to construct transcriptional modules. Lee et al. [18] used the ChIP-chip technology and genome-wide location analysis to infer transcriptional regulatory networks in S. cerevisiae.

Beer and Tavazoie (BT) [19] proposed a novel formulation of the sequence–expression problem. They asked the very intriguing, but seemingly impossible, question: how much can we predict gene expressions from gene upstream sequences? To address the question, they first clustered a large portion of genes in S. cerevisiae into 49 tight co-expression groups, found enriched sequence patterns (motifs) among the promoter sequences of genes in each group using de novo motif prediction tools [6,20], and then trained a set of Bayesian network models to predict the group membership of each gene using the matching scores of its promoter sequence to the set of sequence motifs as well as the orientation and position of the predicted binding sites. They conducted a 5-fold cross-validation (CV) procedure to estimate their model's prediction power and found its prediction accuracy to be as high as 73%. A great benefit of the Bayesian network, as shown by BT, is its ability to learn “combinatorial codes” for gene regulation. Hvidsten et al. [21] have applied a similar approach to infer “IF–THEN” rules for transcription regulation. While Bussemaker et al. [11] and Conlon et al. [14] aimed at using gene expression information to help discover transcription factor binding motifs (TFBMs) and binding sites, BT focused directly on the prediction problem.

However, a few key questions remain. First, BT's assessment of their method's prediction power is over-optimistic, as their CV procedure did not include the motif-finding step (more details later). But, how much can we really predict? Second, is the Bayesian network an appropriate model for the task or just too complex a black box, prone to overfitting for the stated tasks? Third, do those inferred combinatorial rules have real predictive power, or are they only observational oddities after the model fitting? How should we think about and quantify uncertainties inherent in such inferred models? Given the limited amount of data and the vast number of potential predictors (e.g., 666 sequence motifs, orientations, and positions of candidate motif sites, etc.), it is not clear if a complex-structured model can be fitted with any confidence.

Our plan to address the above concerns is as follows. We first use the same data and the same (but wrong) CV procedure as in [19] to develop our predictive models, naïve Bayes classifiers with feature preselections, so as to study the problem of model fitting. Then, we study contributions of various sequence features, such as orientations and positions of the predicted binding sites, to the prediction accuracy. Lastly, we implement a correct CV procedure and show the difference of prediction accuracies resulting from correct versus incorrect CV procedures.

Based on the same gene clustering information, putative TF binding motifs, and gene upstream sequences as in [19], our naïve Bayes classifiers outperformed BT's Bayesian network without using any information regarding the position and orientation of the predicted TFBSs. Our classifiers typically select more motif features, but have far fewer model parameters than the Bayesian network models in [19]. We also found that adding the information regarding TFBS orientation and position cannot further improve the naïve Bayes classifier's predictive power in a global way, which casts doubts on several biological predictions made in [19] regarding combinatorial rules of gene regulation. We further studied a few cases in detail and found that the supports for the inferred combinatorial rules are at best circumstantial. Finally, we speculate that the incorrect CV procedure used in [19] has likely overestimated the accuracy rate of their method by 10%.

## Results

### Data and Procedure

The data used in this study were obtained from the supplemental Web site of [19], which contains matching scores (i.e., the likelihood of a promoter sequence to contain good sequence matches to a candidate TFBM), and orientations and positions of the predicted matches of 666 putative TFBMs for 2,587 genes in S. cerevisiae. In [19], these 2,587 genes were clustered into 49 different co-expression groups according to their expression profiles in 255 conditions, such as environmental stress [22] and cell cycle [8]. We trained a set of naïve Bayes classifiers to predict the cluster label (membership) for each gene using only its motif matching scores. Since genes in the same cluster have very similar expression profiles, a gene's cluster membership can serve as a surrogate of its expression behavior under different conditions.

**Figure 1:**
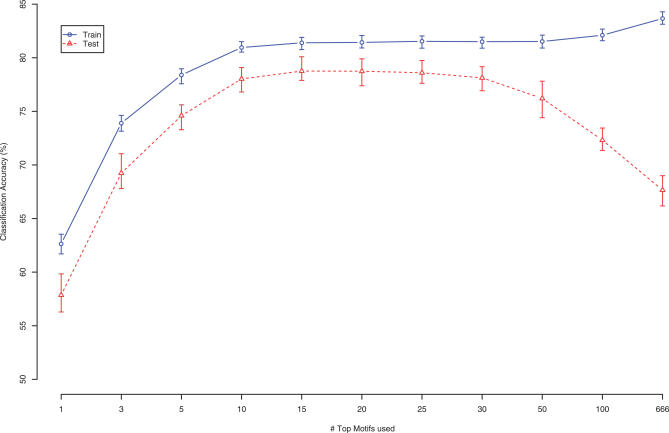
Training and Test Set Classification Accuracy for Naïve Bayes Method Using Motif Scores Only Classification accuracies for training sets increases with the number of top motifs selected in models, while test set accuracies only increase when model sizes are small. Including too many features will overfit the training set and thus decrease the test set accuracies. 100 random repeats of 5-fold CVs were performed, and the curves display the mean accuracies. The error bars denote the maximum and minimum accuracy achieved in the 100 random repeats.

We built one naïve Bayes model for each cluster, resulting in a total of 49 classifiers. For each cluster, we first ranked all the 666 sequence motifs according to a Chi-square test procedure, which reflects these motifs' capability of differentiating genes in this cluster from all other genes. Then, we selected the top *m* most significant motifs as explanatory variables to train a naïve Bayes classifier (for this cluster), where *m* can range from 1 to 666. We used the same 5-fold CV procedure as that in BT to test the predictive power of our models. As shown in Figure 1, using the same criteria for classification accuracy as in [19] (i.e., for any pair of clusters, if the correlation between their mean expression is greater than 0.65, then misclassifying genes in one cluster into the other is *not* counted as errors), naïve Bayes classifiers correctly predicted expression patterns for 75% of the genes when the number of preselected motifs *m* is 5. When *m* is increased to 20, naïve Bayes classifiers achieved a 79% prediction accuracy (see Table S1). In addition, the naïve Bayes models contain almost all the motif features selected by BT in [19] and include many more (see Figures S1 and S2). It can also be seen that, although the training accuracy always increases as *m* increases, the prediction accuracy starts to plateau and then decrease as *m* exceeds 20, which is indicative of overfitting as more variables are included. Following BT, we also calculated the mean correlation of each gene to its predicted expression pattern. For a gene, its predicted expression pattern is the mean expression pattern of the cluster that it is predicted to belong to. With our 20-motif naïve Bayes model, we obtained a mean correlation of 0.56 without using any position and orientation information, which is also higher than BT's result of 0.51.

### Biological Interpretations of Predictive Models

Having fitted the classification models, we now study how the 666 motifs are present in the model of each cluster. Our first observation is that most clusters have their distinct sets of motif features. But a few motifs are selected by multiple clusters, which may indicate that either the transcription factors corresponding to these motifs are somewhat multi-taskers, or the clusters that share these common motifs are closely related. For example, Motifs PAC and RRPE are selected in the models for clusters 4, 10, 17, 26, and 29. This suggests that many genes in these five clusters may be targeted by the TFs that bind to PAC and RRPE. Clusters 47 and 48 share 17 out of 20 motifs in their models (*p* < 1 × 10^−21^). Coupled with the fact that the correlation of the mean expression patterns of these two clusters is more than 0.8, it strongly suggests that genes in these two clusters are co-regulated.

Motif PAC is associated with polymerase A and C subunits [20,23]. Motif RRPE specifically exists in genes involved in rRNA processing [20]. BT extracted from their model a combinatorial prediction rule for cluster 4 [19]: PAC should have a score higher than 0.6 and be within 140 bp of ATG; RRPE should have a score higher than 0.65 and be within 240 bp of ATG. Table 1 shows numbers of genes in a few different clusters that satisfy these constraints. The statistics suggest that PAC and RRPE are both significantly enriched in cluster 4, but not uniquely. Clusters 10, 17, 26, and 29 also have significant portions of genes that satisfy the constraints of both motifs. Our naïve Bayes method successfully picked PAC and RRPE for all these five clusters, whereas BT did not select RRPE for cluster 10, or PAC for cluster 29. It suggests that, due to its complex nature, the Bayesian network model in [19] can easily miss important features. Furthermore, our method using no information about TFBS orientation and position correctly predicted 94% of the genes in cluster 4 and 87% of the genes in clusters 10, 17, 26, and 29, which is comparable to the 92% and 87% accuracy of [19] for the same clusters.

**Table 1:**
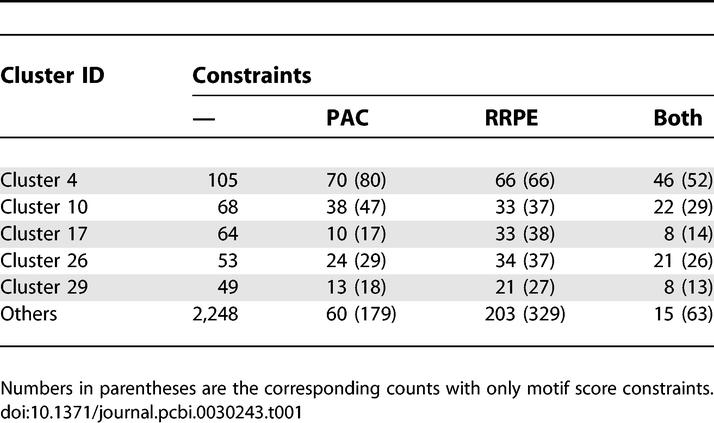
Number of Genes That Satisfy PAC and RRPE Constraints (PAC score >0.6, Located within 140 bp of ATG; RRPE score >0.65, Located within 240 bp of ATG)

RAP1 is a main regulator of ribosomal proteins in S. cerevisiae, and many ribosomal protein coding genes are reported to have RAP1 binding site(s) in their upstream sequences [24]. BT [19] found that cluster 1 is enriched with RAP1 binding sites, and their Bayesian network inferred a rule for genes in this cluster: their RAP1 score on upstream sequences has to be greater than 0.6, and their RAP1 sites have to be oriented toward a certain direction. We examined this rule carefully and observed the following. First, we found that 82 genes in cluster 1 (a total of 124 genes) and 165 genes in other clusters (a total of 2,463 genes) have putative RAP1 binding sites (i.e., with RAP1 matching score >0.6), which gives rise to a *p*-value of 1 × 10^−59^ (based on Fisher's exact test) for the enrichment of RAP1 sites in cluster 1. Seventy-three genes in cluster 1 and only 85 genes in other clusters satisfy both the orientation and the site score requirements, which yields an even more significant contrast *p*-value, 1 × 10^−64^. It seems that the RAP1 orientation can indeed help enhance the prediction specificity, although only slightly.

**Figure 2:**
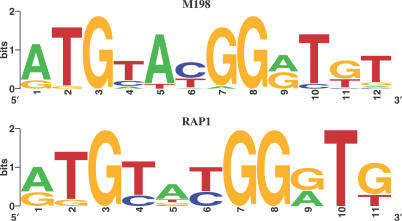
Motif Logos of M198 and RAP1 These two TFBMs are very similar, except that M198 is one position longer than RAP1 on the right end. Compared to RAP1, M198 can help distinguish genes in cluster 1 from other genes in a higher statistical significance, without using any position or orientation constraints.

However, our naïve Bayes model selected motif M198 as its main predictor for genes in cluster 1. This motif has a very similar weight matrix to that of RAP1 but includes an extra position (Figure 2). By setting 0.6 as the score threshold of M198, we found that 100 genes in cluster 1 and 126 genes in other clusters contain the M198 site, which gives us a *p*-value of 4 × 10^−94^ for the M198 enrichment in cluster 1. Thus, if judged by statistical significance of the prediction specificity, the naïve Bayes model with one simple predictor easily outperformed the more complex combinatorial rule inferred by BT's Bayesian network.

In order to evaluate the effectiveness of RAP1 (with orientation constraint, denoted as RAP1d for short) and M198 as covariates in our classifier, we compared two procedures. In both procedures, one single best motif was selected for each cluster. The only difference was that, for cluster 1, M198 was used in Procedure One and RAP1d was used in Procedure Two. As a result, Procedure One predicted 20 more genes correctly than Procedure Two, and the improvement is mainly in cluster 1. For cluster 1 alone, Procedure One has a 30% false positive and 18% false negative rates, while Procedure Two has a 38% false positive and a 34% false negative rate. These results further suggest that M198 is a better motif for cluster 1 than the oriented RAP1. In the next subsection, we provide a more thorough investigation on the biological relevancy of motif site orientation and its effect on the classification accuracy.

### Effect of TFBS Orientation and Position

The result in the previous subsection does not mean that the motif site orientation is not biologically important. In fact, we found that 91 of the 100 predicted M198 sites for genes in cluster 1 are oriented toward one direction. In comparison, only 56 of the 126 predicted M198 sites for genes in other clusters are oriented the same way. Clearly, including both the M198-score and its site orientation constraints can improve the prediction specificity for cluster 1, as observed by BT for RAP1. However, in a similar procedure comparison as in the previous subsection, adding the orientation constraint of M198 does not improve the global prediction. This orientation constraint may help reduce the false positive rate for cluster 1, but it at the same time increases false positive rates in other clusters. Thus, a fundamental question is: is it appropriate to justify the “authenticity” of a prediction model based on its prediction performance? Our analysis suggests that a combinatorial regulation rule, and perhaps many other causal relationships, may not be reliably inferred using an automatic “learning machine” under a global classification accuracy criterion.

To assess globally whether the TFBS orientation and position information can further help predict gene expression, we added the covariates representing TFBS orientations and positions to the feature list of our model. We performed the same feature preselection and naïve Bayes procedures as described above on the augmented dataset. The classification accuracies for the training sets were very close to the result from using motif score alone. However, the classification accuracies for the test sets were slightly worse than before. This result implies that, although it may be biologically true that orientations and positions of authentic TFBSs have an effect on the binding of the corresponding TFs in some cases, such information for predicted TFBSs do not help in predicting co-expression of genes globally when motif matching scores are given. Even in BT's Bayesian network models, position and orientation constraints were selected only 5.1% and 0.6% of the time, respectively. In both of the strong cases detailed in [19], we were able to find a simpler rule (matching scores only) that is as sensitive and specific as or better than the combinatorial rules reported by BT.

We would like to caution the reader again, however, that our results cast doubts on some of these delicate model interpretations of BT but do not imply that the position and orientation of TFBSs are biologically unimportant.

### The Cross-Validation Procedure

So far we have followed BT's approach as closely as possible: using the same set of motif features generated by [19] and employing exactly the same CV procedure as theirs. The only difference between our and their approach is that we used the naïve Bayes model, whereas they used the more complex Bayesian network.

However, we cannot help notice that the 615 de novo motifs (excluding the 51 known motifs) generated by [19] were found by using the Gibbs motif sampler AlignACE [20] to search the upstream sequences of *all* genes in both the training and the test datasets for each cluster. These motifs were further optimized so as to be more specific to the respective clusters they were discovered from by a simulated annealing procedure [19], still using all genes in both the training and test datasets. These steps inevitably generate motifs (features) that are already biased in favor of the existing clustering in the test set. In a valid CV procedure, only the information for the training set genes, including both their upstream sequences and their cluster labels, are allowed to be used in both feature extraction and model training.

To correctly measure how much of gene expression information can be predicted by DNA sequence features, we implemented a valid 5-fold CV procedure, still using the gene clustering result of BT. First, genes in each cluster were divided into five sets of approximately equal sizes at random. Each time, we left out 20% of genes (one subset of genes for each cluster), and used the remaining 80% of genes (i.e., the training set) and their upstream sequences for de novo motif finding via AlignACE [20]. These motifs were then optimized by a simulated annealing algorithm. The total number of motifs we found ranged from 600 to 700 for each training set, which is consistent with the number of 666 motifs in [19]. We then preselected the top 20 motifs (see Figure S3) for each cluster and trained naïve Bayes classifiers based on the training set and the preselected motifs. Finally, the classifiers so trained were used to predict the cluster memberships of the left-out 20% genes. The classification accuracy of this correct CV procedure is 61% according to the criterion in [19], which is still significantly higher than random guessing. When we further added the 51 known motifs to the motif sets, the classification accuracy increased to 64%.

Note that we cannot directly use the motif finding and model-fitting procedure of [19] because their complete algorithm is not publicly available. Furthermore, their-model fitting procedure needs bootstrapping replications and can be overly time consuming, unstable, and nonreproducible. Thus, there is a possibility that the low accuracy of our correct CV procedure is caused by the lower capability of our motif finding strategy compared to that of [19]. To calibrate with BT's approach, we also applied the exact same incorrect CV procedure as in [19] using our own motif finding, optimization, and model-fitting strategies described above. When using all the genes in all clusters, our de novo motif discovery strategy found altogether 650 motifs, and the whole procedure yielded a classification accuracy of 75%, which is slightly higher than the result of [19] (73%). Based on these results, we conclude that the incorrect CV procedure of [19] has likely overestimated the true prediction accuracy of their expression prediction method by 10%–15%.

## Discussion

The naïve Bayes model we adopted is essentially the simplest version of the Bayesian network. The assumption of conditional independence of the covariates is far from realistic in most applications, as well as in this study. However, it outperformed the more complicated Bayesian network, as well as SVM, CART, logistic regression, and Bayesian logistic regression [25] (unpublished data) for this study. As described by Domingos and Pazzani [26], optimality in terms of zero-one loss (classification error) is not necessarily directly connected to the quality of the fit of a probability distribution. Rather, as long as both actual and estimated distributions agree on a most-probable class, the classifier will have a reasonable performance.

Although it is not rare to see successful examples of the naïve Bayes method, the feature selection step is always challenging. In our method, features are considered independently. Each feature is dichotomized to 0 or 1 according to a threshold that maximizes a Chi-square test statistic. In this way, features that are highly associated with a target cluster will be selected as covariates in the naïve Bayes model of this cluster. Our method selects not only the features that are enriched in the target cluster, but also those that are “depleted” in the target cluster but enriched in other clusters. The latter type of features can be explained as a logic operator “NOT”.

Dichotomization of motif scores in our procedure is a gross simplification. Although the binding of a TF to DNA may not be a simple 0–1 trigger, it is easier to model it in this way, and it is also interesting to see whether this simple model can help predict gene expression. We expect to lose some information through discretization, but it is not clear how much the lost information can help the classification problem. It is a worthwhile future project to explore possibilities of using the continuous data, both motif scores, and gene expression values, directly and more efficiently.

Our study has shown that it is perhaps not very sensible to justify a model's “authenticity” by its global prediction performance, and one may easily inject subjective interpretations into the inference results, especially when the prediction uncertainty is not explicitly quantified. This in fact is a challenge for many machine learning approaches, and researchers have begun to pay attention to the problem of estimating prediction uncertainties. In this regard, it is perhaps beneficial to act more like a real Bayesian when using Bayesian tools. That is, these tools not only provide point estimates, but also posterior distributions, which summarize all the information in the data and quantify uncertainties of the estimates.

The keen difference between the correct and incorrect CV procedures reminds us how easy it is to be overconfident. Similar mistakes have also been uncovered in some computational biology studies in which knowledge from literature is used to help construct gene clusters or biological networks and these results are then evaluated and validated by GO analysis, which is by itself a product partially based on the literature.

Although it has been accepted as common knowledge in biology that TFBSs' orientation and position have a functional role in affecting gene regulation activities, and anecdotal examples abound [27,28], it is still nonconclusive how the orientation and position information of putative TFBSs can help one discern true TFBSs from sporadic sequence matches that exert no regulatory functions. In particular, the TFBS orientation and position information did not help us improve the classification accuracy globally, and was not even obviously useful in the two strongest cases detailed in [19]. Since the Bayesian network in [19] is more prone to overfitting, the danger of overinterpreting the fitted models can be a serious threat. In a recent study of nucleosome positioning in yeast, Yuan et al. [29] observed that true regulatory elements are highly enriched in nucleosome depleted regions. Thus, certain sequence information at a scale of nucleosome binding regions (larger than TF binding sites) may be more useful than orientation and position information in differentiating true TFBSs from false ones.

## Materials and Methods

### Data.

For motif *j*, its score for gene *i* is denoted as *s_ij_*, which is computed in [19] as either zero, when motif *j* has no predicted occurrence in the promoter of gene *i*, or the highest matching score among all predicted occurrences of the motif in the promoter of gene *i*. In this way, a score matrix **S** = (*s_ij_*)_2587×666_ can be built directly from the supplement data of [19].

### Discretization and feature selection.

The continuous scores *s_ij_* are discretized into 0 or 1 by a thresholding procedure described below. In a word, a threshold for the scores corresponding to a motif is chosen so as to maximize the *specificity* of TFBSs for the cluster of interest. Let *N* be the number of all the genes in consideration (i.e., 2,587) and let *y_i_* be the class label of gene *i* (*i* ∈ {1, ···,}*N*). Among these *N* genes, *N_k_*_,1_ of them are in class *k* (defined as positive set) and *N_k_*_,0_ are not in class *k* (defined as negative set). Thus *N_k_*_,1_ = #{*i*:*y_i_* = *k*}, *N_k_*_,0_ = #{*i*:*y_i_* ≠ *k*} and *N_k_*_,1_ + *N_k_*_,0_ = *N*. For motif *j* (*j* ∈ {1, ···,666}) and a threshold *c*, define

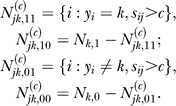



The best threshold for motif *j* in model *k* is defined as:

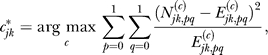
where

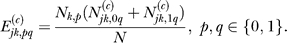



More intuitively, the above procedure finds the most significant Chi-square test result for the 2 × 2 contingency table of the *N*'s. This procedure makes the distribution of TFBSs in positive set and negative set most different. The thresholds calculated above discretize the score matrix **S** into a 0–1 matrix and it is denoted as **X**. Note that the discretized covariate matrix **X** will be different for fitting models in different classes.

The feature preselection step is simply an extension of the threshold finding procedure. For model *k*, the best threshold 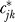 is calculated for motif *j* along with its highest *χ* ^2^ statistic. Features (motifs) are sorted by their *χ* ^2^ statistics, and the top *m* ones are included the models. This selection is done for each model separately.

### The naïve Bayes model.

The naïve Bayes method has been widely used in statistical learning. It is based on the very simple assumption that all feature variables (covariates) are independent given the class label of the sample. We use cluster 1 and its preselected *m* motifs as an example to describe our naïve Bayes model fitting procedure. Denote the class label variable as *Y* and the preselected top *m* covariates as *X*_1_, ···, *X _m_*. Using the Bayes theorem, we have

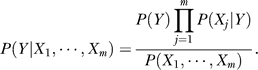
Thus, the odds ratio can be computed as

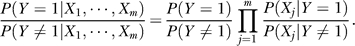



We further assume Bernoulli models for each *X_j_* given *Y* and class label variable *Y* itself, i.e.,

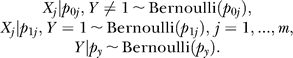



The prior distributions for *p_y_*, *p*_0*j*_, and *p*_1*j*_ are set to be uniform. The training set consists of a class label vector **y** = (*y*_1_, ···,*y_N_*) and the discretized TFBS score matrix **X** = (*x_ij_*),*i* = 1, ···,*N*; *j* = 1, ···,*m*. Given the training set, the posterior distribution of *p_y_*, *p*_0*j*_, and *p*_1*j*_ can be easily calculated as

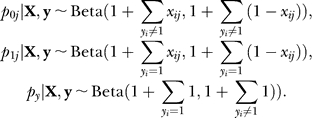



For a new observation with the covariates vector **X** *_new_* = (*X*_1,*new*_,...,*X_m_*_,*new*_), we have

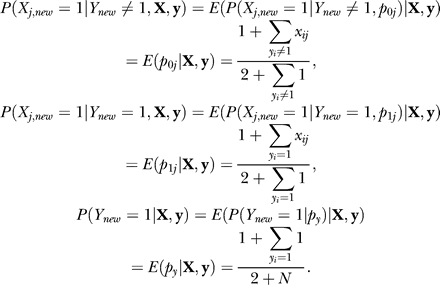
Thus, we have the predictive odds ratio for this new observation as

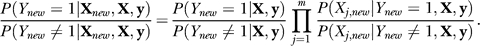



For the 49 classes, 49 models are fitted and the genes in the test set are assigned to the class with the respective model that fits the data best. Specifically, for *k* = 1, ···,49, the odds ratio

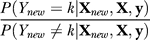
can be calculated and a gene will be assigned to a class *k* ^*^ with the highest odds ratio.

### TFBS position and orientation.

To reduce the complexity, for each motif on each gene we only consider the orientation and position of the site with the highest matching score. The site orientation is coded into two separate binary variables, *x_l_* and *x_r_*, where *x_l_* = 1 indicates that the predicted site is left-oriented (away from ATG), *x_r_* = 1 for right-oriented, and *x_l_* = 0 or *x_r_* = 0 otherwise. Note that when a gene does not contain TFBS for a specific motif, the corresponding *x_l_* and *x_r_* are both 0. The TFBS position in [19] is a continuous variable representing the distance of the TFBS to ATG. We set it to a very large number if a motif has no occurrence in the promoter region of a gene. In our naïve Bayes procedure, the new variable *d* is a dichotomized version of the original position variable based on an optimized distance threshold, so that *d* = 1 means that the distance from the predicted site to ATG is smaller than the chosen threshold.

## Supporting Information

Figure S1 Motif Selection in Clusters (Top 15 Motifs for Each Cluster) Rows are clusters and columns are motifs. A red bar represents the column motif selected in the model for the row cluster. Motifs and clusters are arranged such that similar selection patterns are close to each other. Most clusters have a unique selection of motifs. The green rectangle shows that six clusters share some motifs in their models.
(24 KB PNG)

Figure S2 Top Five Motifs Selected in Each Cluster All 2,587 genes are used to make this list.
(1.2 MB PDF)

Figure S3 Top Five Motifs Selected in Each Cluster in 5-Fold CV In each CV, a set of motifs are generated using the training set only. The known 51 motifs are included too.
(5.4 MB PDF)

Table S1 Classification Accuracy of 49 Clusters Using Top 5/20 Motifs in Each Cluster (126 KB DOC)

## Abbreviations

BT: Beer and Tavazoie
PAC: polymerase A and C box
RRPE: ribosomal RNA processing element
TF: transcription factor
TFBM: transcription factor binding motif
TFBS: transcription factor binding site
