# Supplementary figures and images for: Predicting Gene Expression from Sequence: A Reexamination
Source: PLoS Comput Biol. 2007 Nov 30;3(11):e243. doi: 10.1371/journal.pcbi.0030243 (PMC2098866; doi:10.1371/journal.pcbi.0030243)

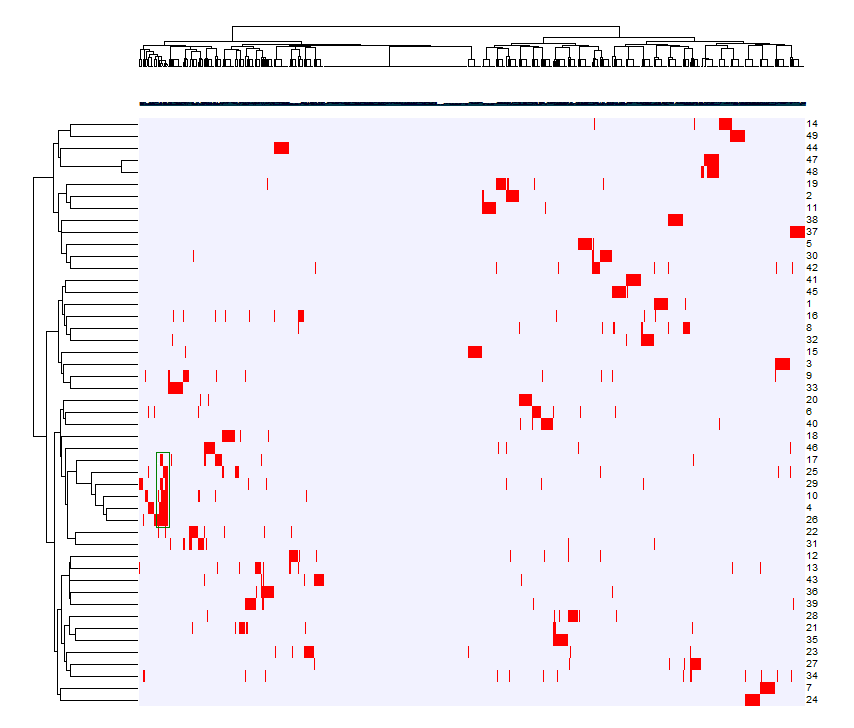

Supplement: Figure S1 — Rows are clusters and columns are motifs. A red bar represents the column motif selected in the model for the row cluster. Motifs and clusters are arranged such that similar selection patterns are close to each other. Most clusters have a unique selection of motifs. The green rectangle shows that six clusters share some motifs in their models. (24 KB PNG) [file pcbi.0030243.sg001.png]
