# Supplementary material for: Predicting Gene Expression from Sequence: A Reexamination
Source: PLoS Comput Biol. 2007 Nov 30;3(11):e243. doi: 10.1371/journal.pcbi.0030243 (PMC2098866; doi:10.1371/journal.pcbi.0030243)
Supplement: Figure S2 — All 2,587 genes are used to make this list. (1.2 MB PDF) [file pcbi.0030243.sg002.pdf]

Figure S2.

Top 5 motifs selected in each cluster. All 2587 genes are used to make this list.

Cluster	Motif	Chi-square statistic	Score cutoff	Posterior probability within cluster	Posterior probability outside cluster
1	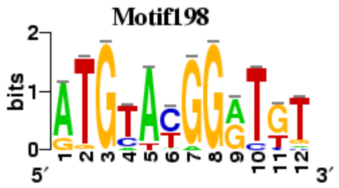	844.72	0.6	0.802	0.052
	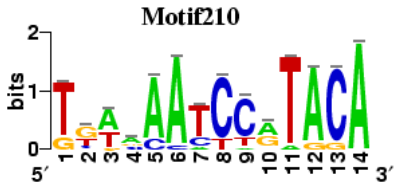	518.41	0.55	0.675	0.065
	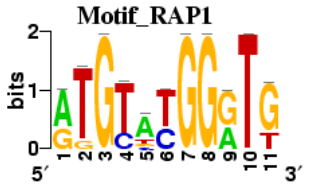	515.83	0.8	0.635	0.057
	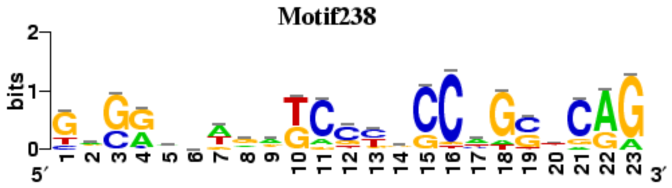	415.41	0.7	0.246	0.005
	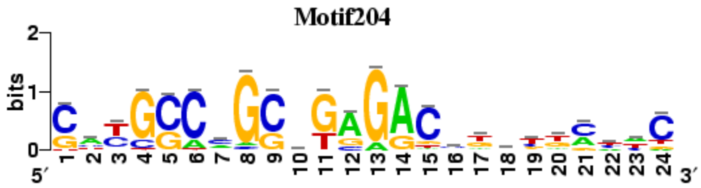	383.13	0.6	0.254	0.007
	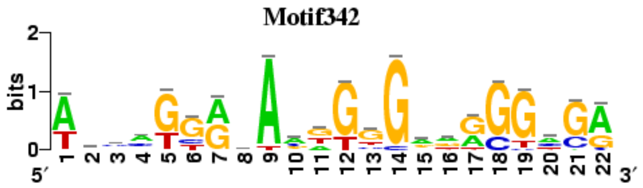	182.86	0.6	0.200	0.013
	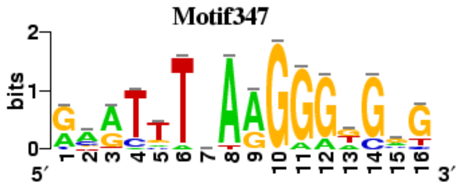	172.45	0.65	0.217	0.017

	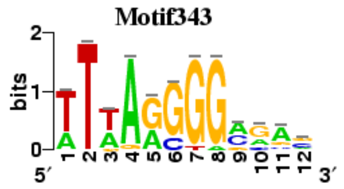	137.25	0.75	0.278	0.037
2	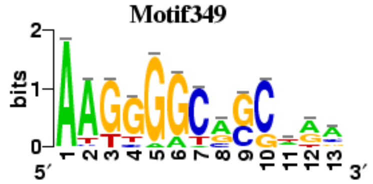	123.37	0.75	0.183	0.017
	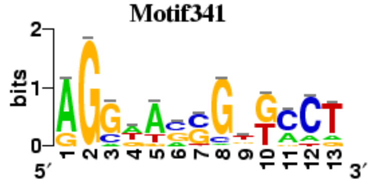	116.26	0.75	0.148	0.011
	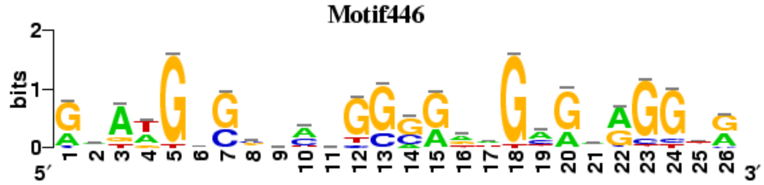	203.33	0.6	0.229	0.015
	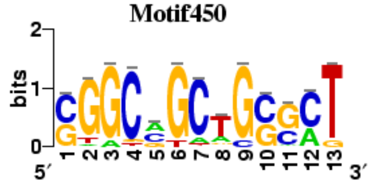	176.68	0.65	0.165	0.008
3	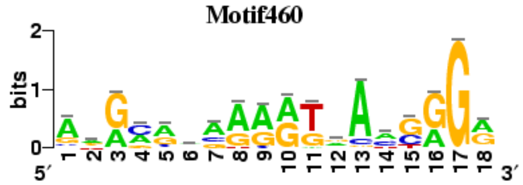	165.65	0.7	0.330	0.042
	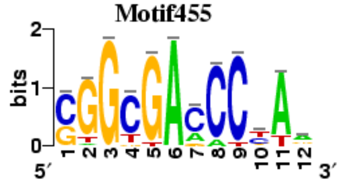	142.20	0.65	0.165	0.010
	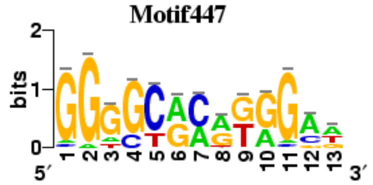	140.23	0.7	0.156	0.009
		455.34	0.6	0.776	0.092

	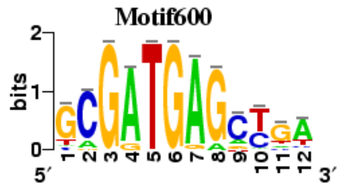				
	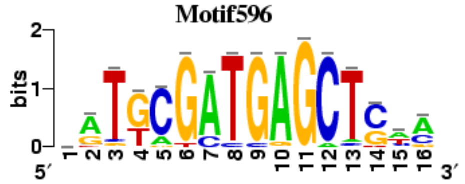	422.07	0.5	0.617	0.059
**4**	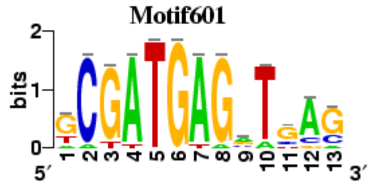	411.40	0.6	0.664	0.072
	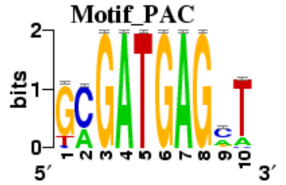	400.45	0.75	0.710	0.087
	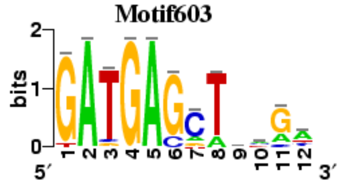	389.92	0.7	0.664	0.076
	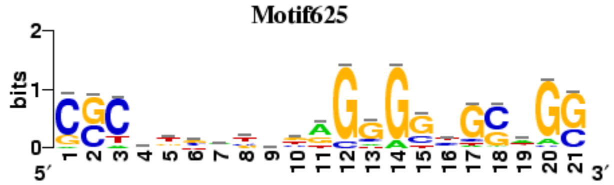	463.89	0.65	0.256	0.003
**5**	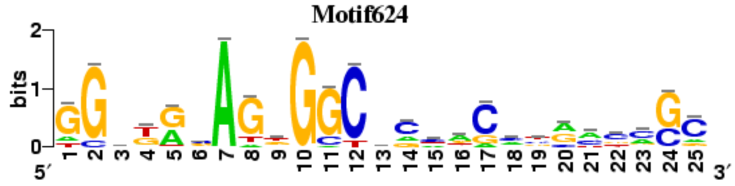	441.93	0.65	0.302	0.007
	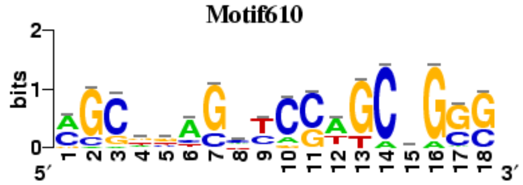	379.83	0.75	0.221	0.003
		363.76	0.65	0.256	0.006

	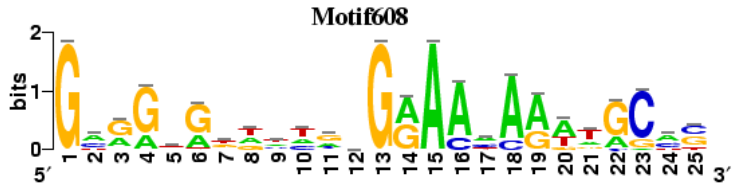				
	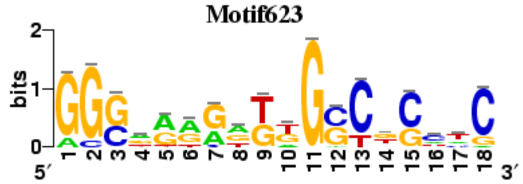	320.50	0.75	0.174	0.002
**6**	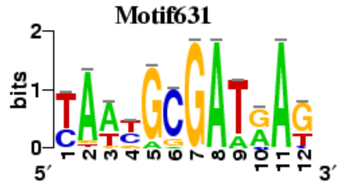	107.33	0.8	0.174	0.014
	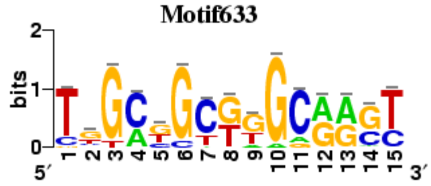	101.78	0.7	0.151	0.010
	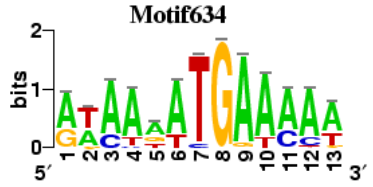	68.43	0.5	0.570	0.198
	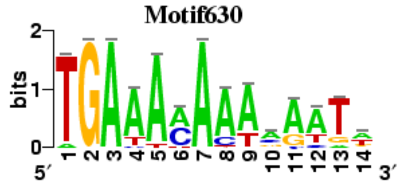	57.94	0.7	0.465	0.153
	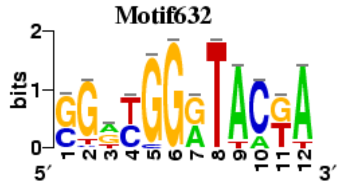	54.07	0.65	0.128	0.014
	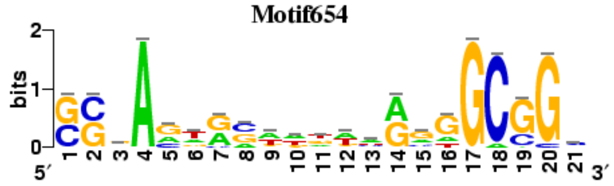	247.46	0.6	0.286	0.015
		230.63	0.65	0.250	0.012

7	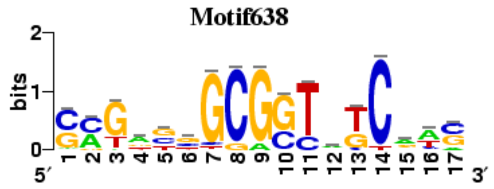				
	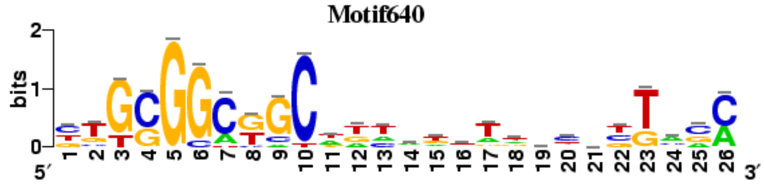	229.58	0.6	0.286	0.017
	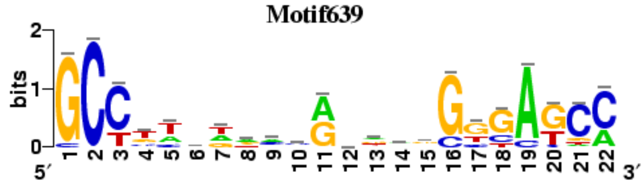	199.80	0.65	0.286	0.020
	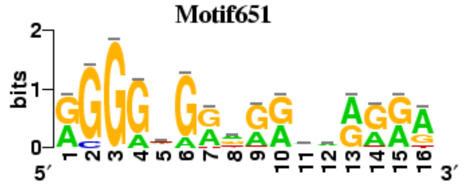	189.87	0.65	0.429	0.051
8	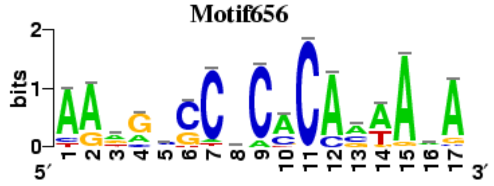	179.49	0.7	0.207	0.010
	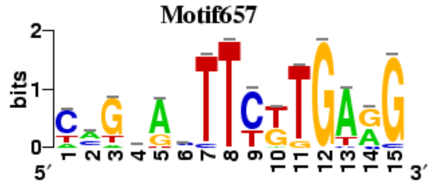	155.95	0.75	0.195	0.011
	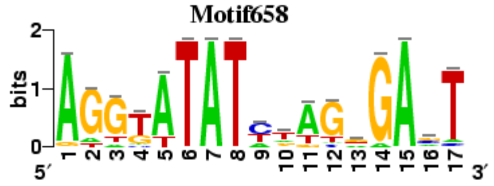	126.75	0.65	0.207	0.016
	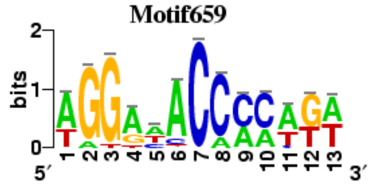	117.55	0.7	0.244	0.025
		103.43	0.75	0.110	0.004

	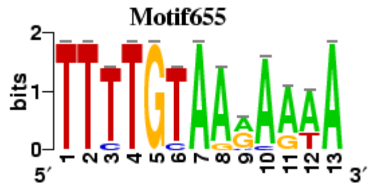				
	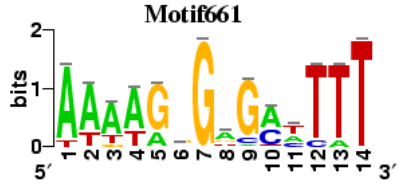	182.64	0.75	0.244	0.014
	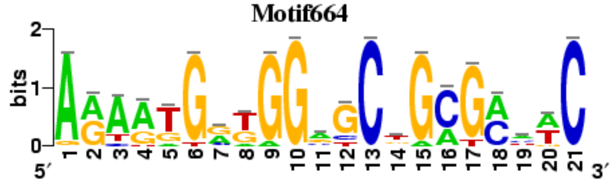	172.02	0.65	0.167	0.006
**9**	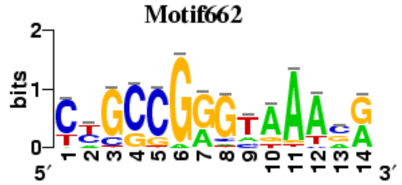	130.97	0.75	0.167	0.009
	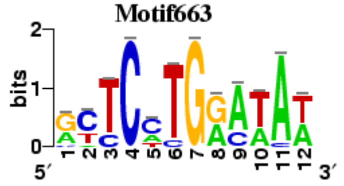	80.38	0.65	0.269	0.041
	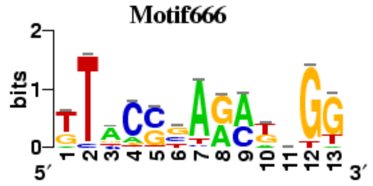	72.02	0.65	0.410	0.100
	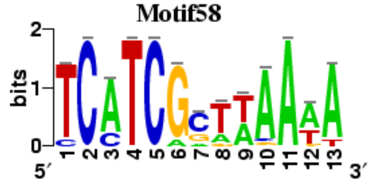	244.77	0.7	0.343	0.020
**10**	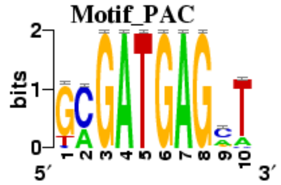	189.47	0.65	0.686	0.118
		185.58	0.75	0.086	0.000

	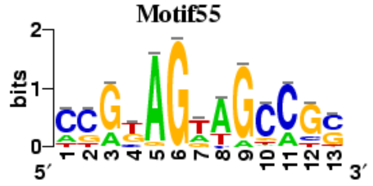				
	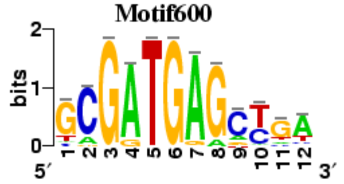	180.23	0.55	0.714	0.134
	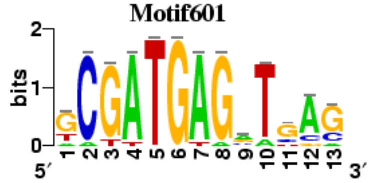	166.74	0.55	0.600	0.100
**11**	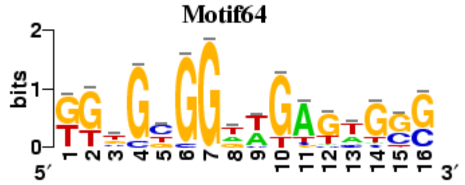	341.43	0.7	0.289	0.009
	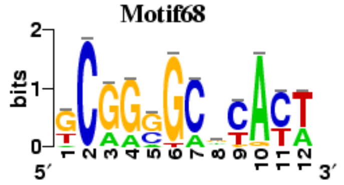	313.84	0.8	0.184	0.002
	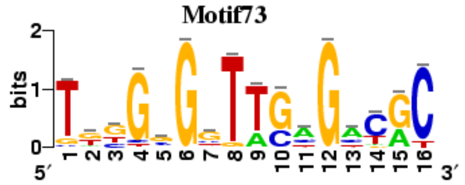	300.17	0.65	0.224	0.005
	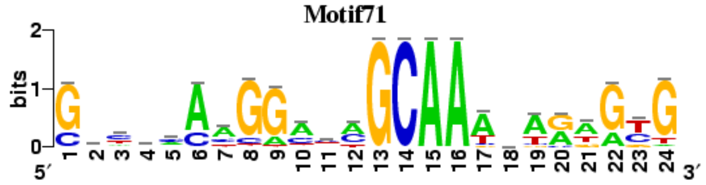	296.62	0.6	0.211	0.004
	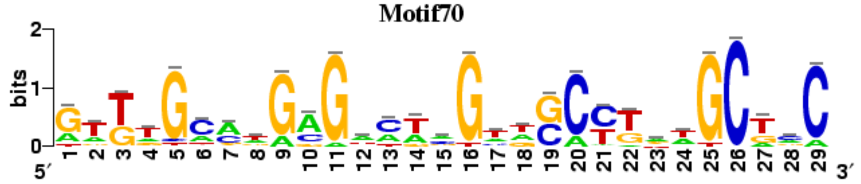	288.84	0.7	0.224	0.006
		265.30	0.75	0.290	0.012

12	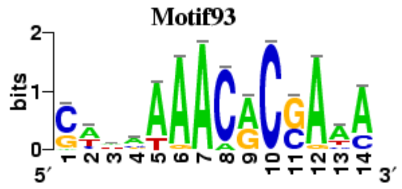				
	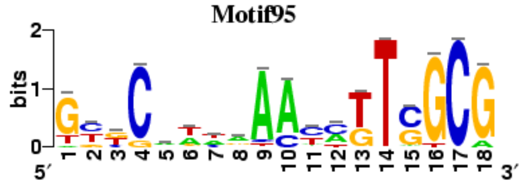	255.44	0.7	0.203	0.005
	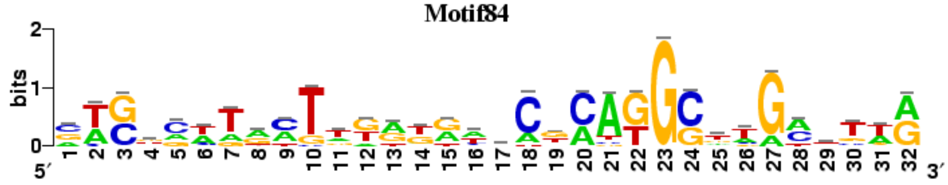	244.29	0.7	0.203	0.005
	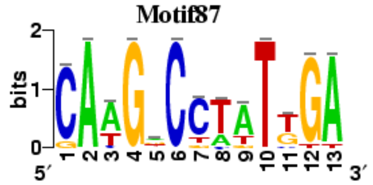	229.34	0.8	0.116	0.001
	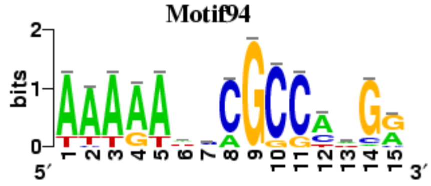	218.36	0.7	0.290	0.015
13	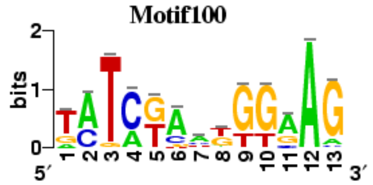	147.81	0.75	0.197	0.010
	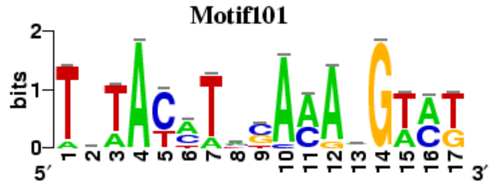	85.71	0.6	0.254	0.032
	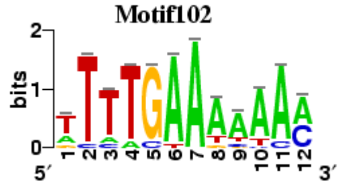	65.91	0.7	0.394	0.093
		36.51	0.95	0.028	0.000

	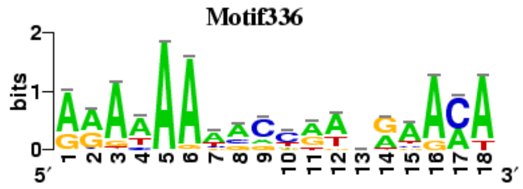				
	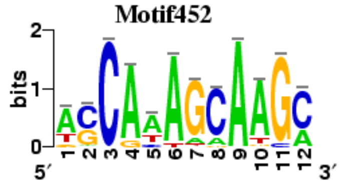	36.51	0.9	0.028	0.000
**14**	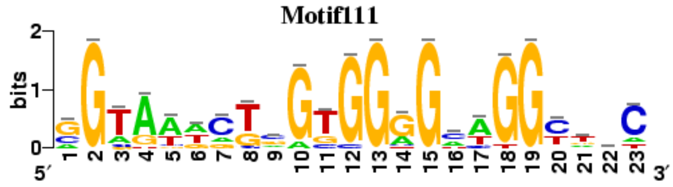	453.75	0.6	0.254	0.003
	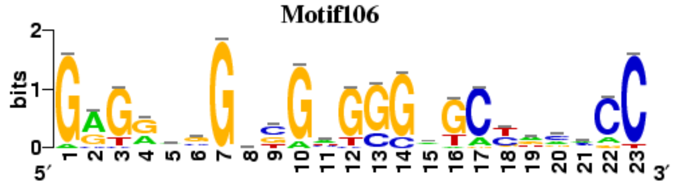	328.97	0.65	0.254	0.006
	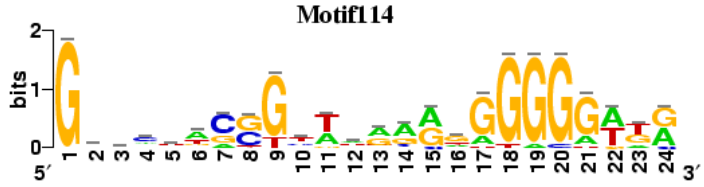	328.23	0.65	0.324	0.012
	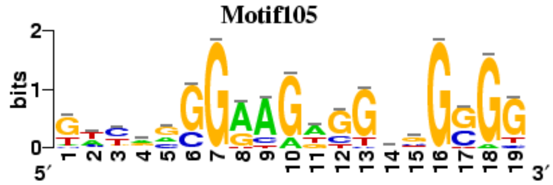	297.33	0.7	0.254	0.007
	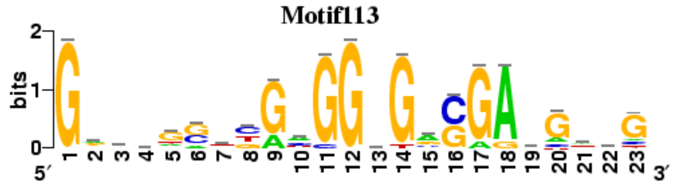	290.35	0.65	0.282	0.010
	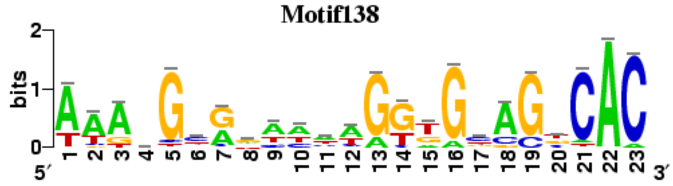	537.43	0.65	0.318	0.004
		518.39	0.6	0.318	0.004

15	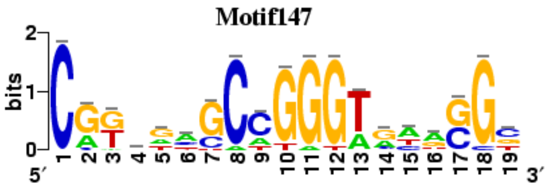				
	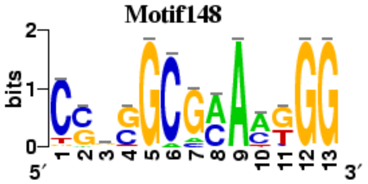	464.59	0.65	0.348	0.008
	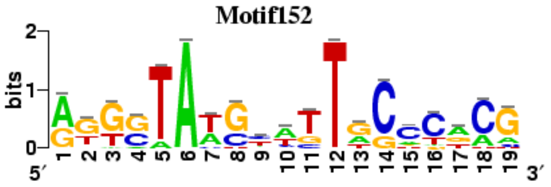	458.44	0.75	0.333	0.007
	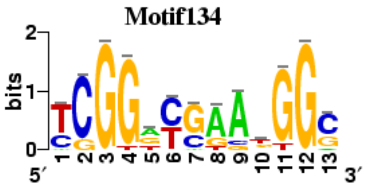	436.46	0.7	0.424	0.015
16	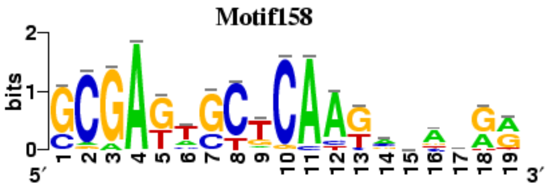	230.00	0.75	0.145	0.002
	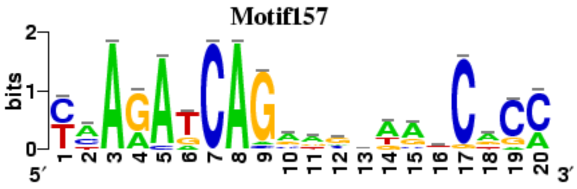	207.40	0.65	0.203	0.007
	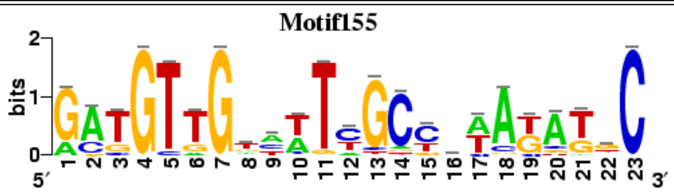	146.99	0.7	0.101	0.002
	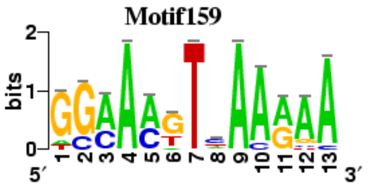	129.15	0.8	0.145	0.006
		79.32	0.7	0.116	0.006

	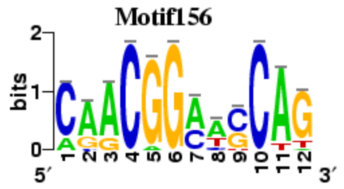				
	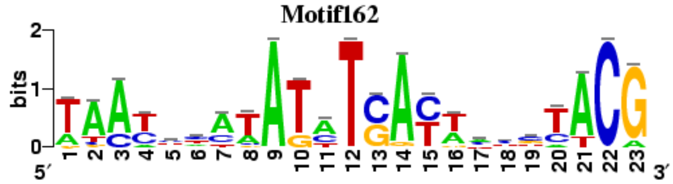	218.83	0.6	0.242	0.010
	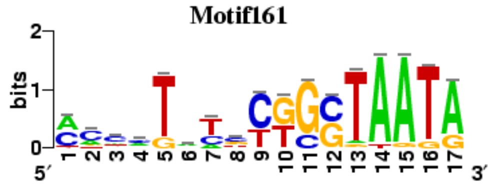	158.27	0.65	0.258	0.017
**17**	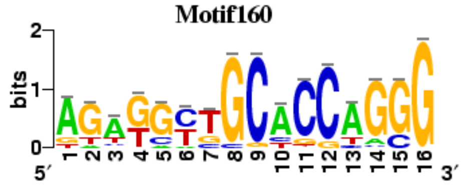	79.19	0.75	0.121	0.006
	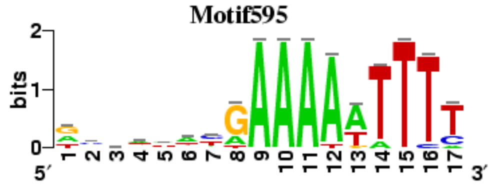	78.50	0.65	0.545	0.143
	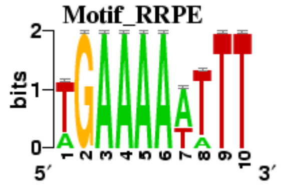	74.42	0.8	0.591	0.172
	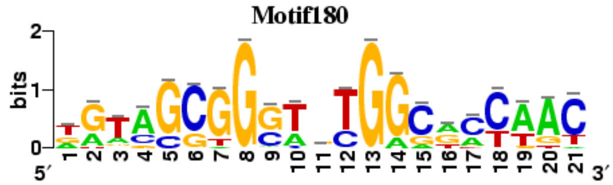	312.47	0.55	0.220	0.004
**18**	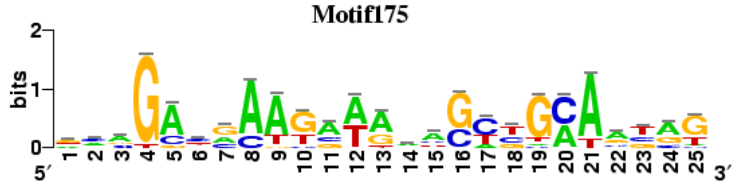	254.60	0.7	0.186	0.003
		232.50	0.75	0.153	0.002

	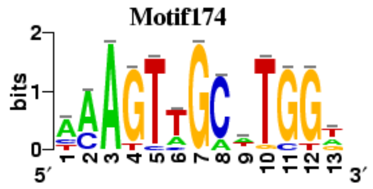				
	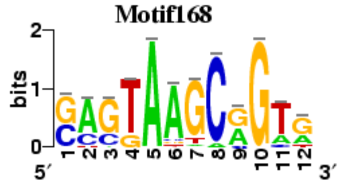	217.11	0.75	0.220	0.007
	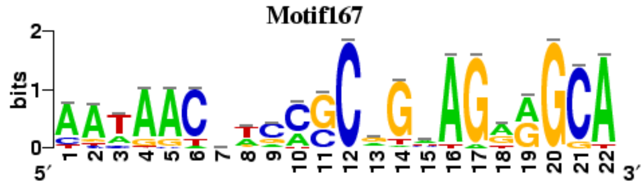	213.69	0.5	0.186	0.004
**19**	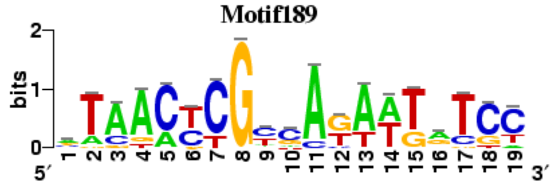	363.94	0.75	0.250	0.004
	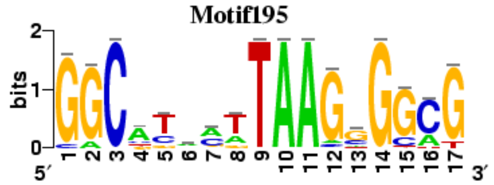	347.91	0.7	0.200	0.002
	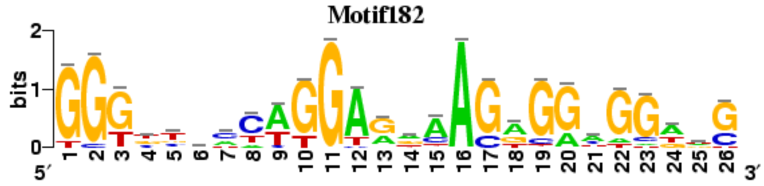	291.16	0.6	0.217	0.004
	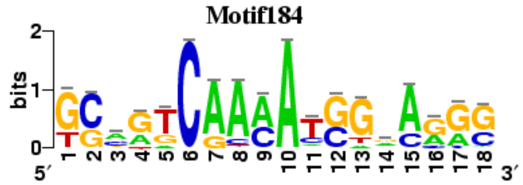	249.96	0.65	0.183	0.003
	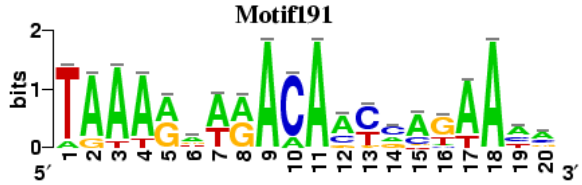	229.65	0.8	0.167	0.003
		528.24	0.7	0.379	0.007

	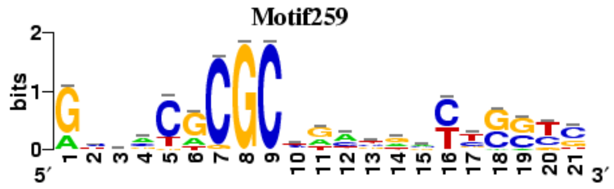				
	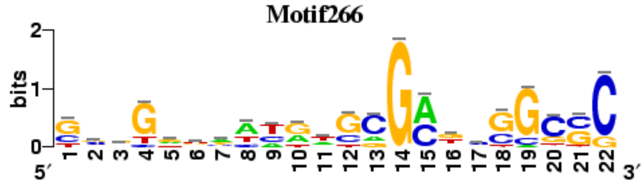	508.35	0.75	0.397	0.008
**20**	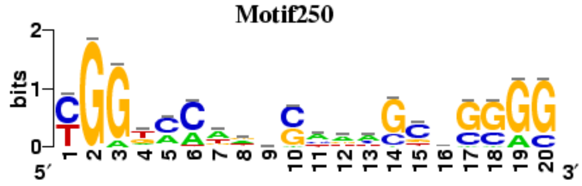	486.06	0.7	0.379	0.008
	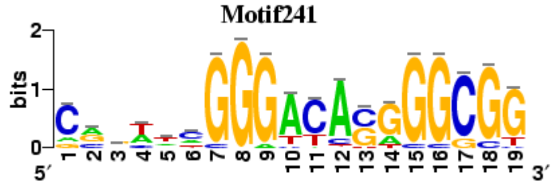	433.06	0.7	0.328	0.006
	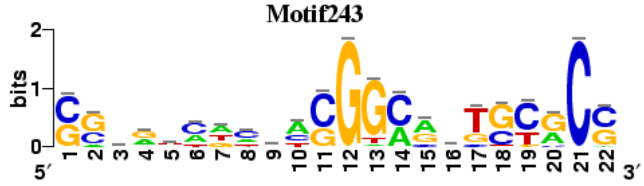	419.97	0.65	0.293	0.005
	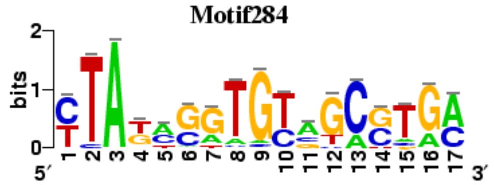	382.92	0.65	0.246	0.003
**21**	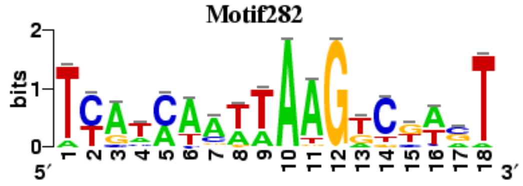	204.81	0.75	0.123	0.001
	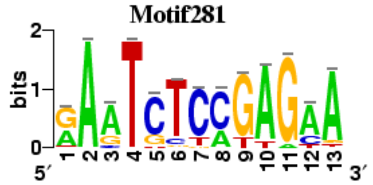	174.02	0.75	0.193	0.006
		143.49	0.75	0.228	0.012

	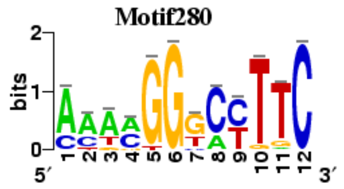				
	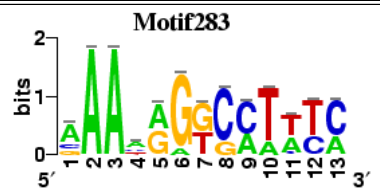	113.38	0.85	0.158	0.007
**22**	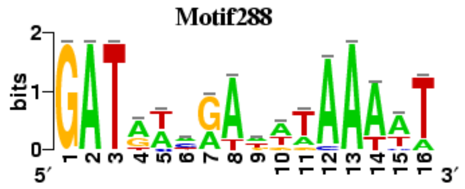	151.19	0.7	0.436	0.047
	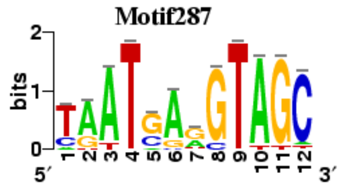	130.55	0.75	0.145	0.004
	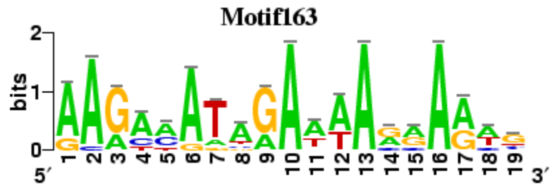	29.33	0.9	0.055	0.002
	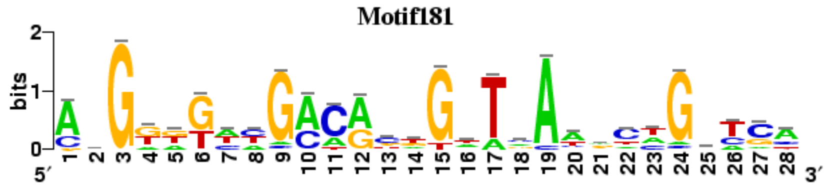	22.93	0.85	0.036	0.001
	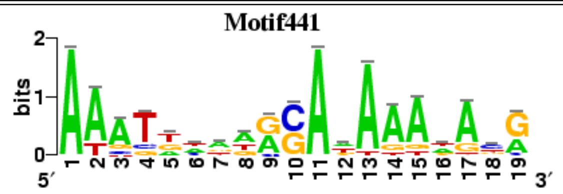	22.93	0.95	0.036	0.001
	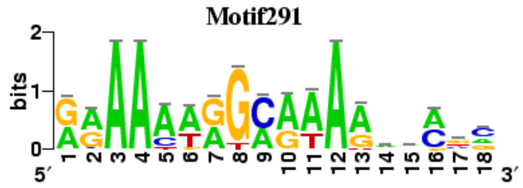	101.71	0.7	0.411	0.060
		80.06	0.7	0.393	0.067

23	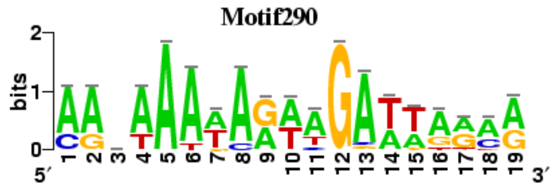				
	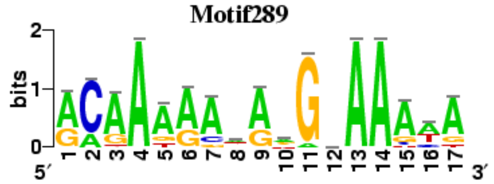	56.99	0.65	0.643	0.215
	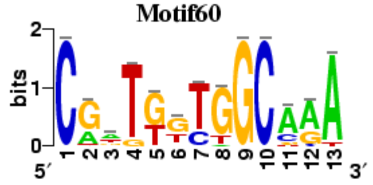	28.73	0.8	0.054	0.002
	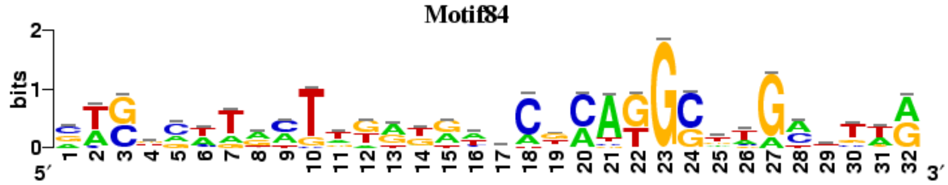	22.48	0.95	0.036	0.001
24	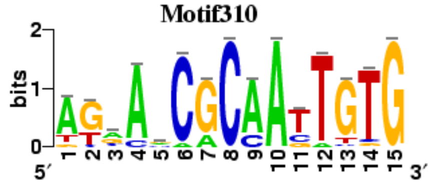	523.60	0.65	0.264	0.002
	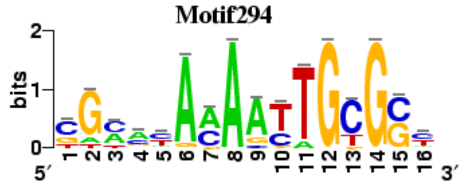	502.59	0.7	0.321	0.004
	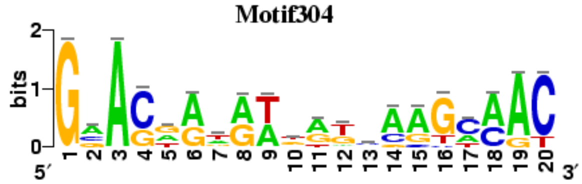	458.54	0.7	0.283	0.003
	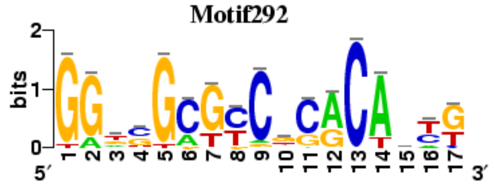	402.53	0.8	0.189	0.001
		381.31	0.7	0.283	0.005

	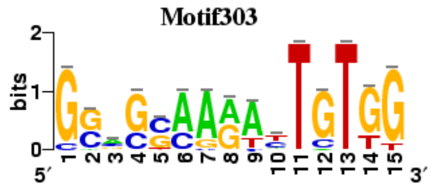				
	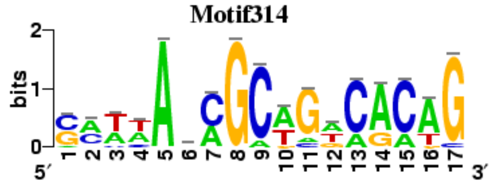	270.97	0.6	0.250	0.006
	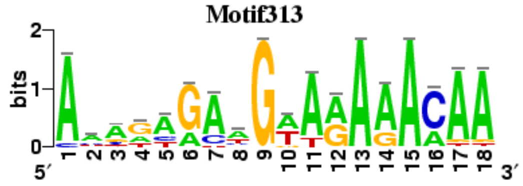	125.19	0.75	0.327	0.029
**25**	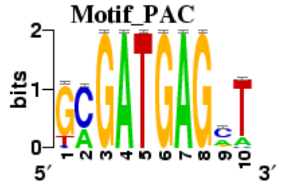	94.64	0.6	0.615	0.134
	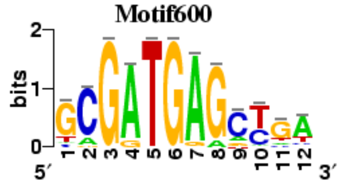	77.78	0.6	0.519	0.112
	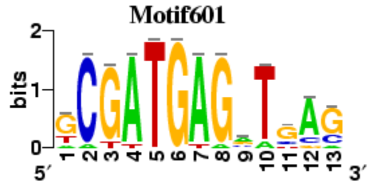	65.36	0.5	0.519	0.127
	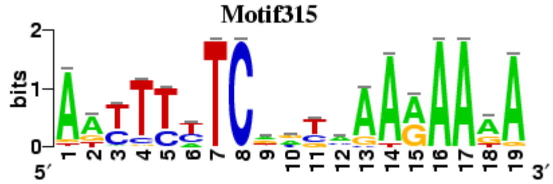	260.57	0.7	0.382	0.019
**26**	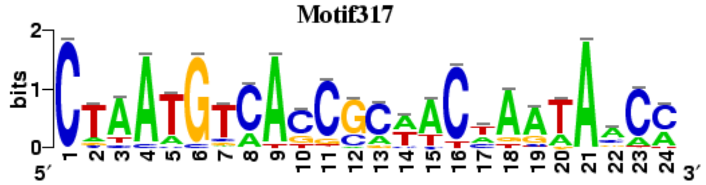	195.90	0.6	0.182	0.004
		122.77	0.65	0.564	0.095

	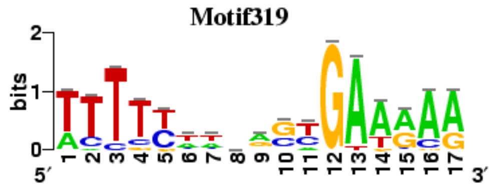				
	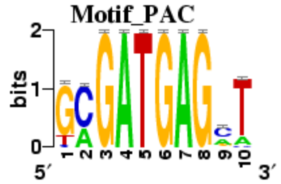	94.63	0.75	0.527	0.103
	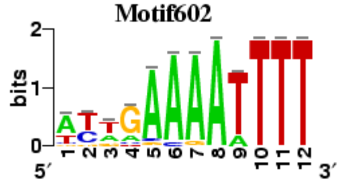	91.22	0.75	0.582	0.129
27	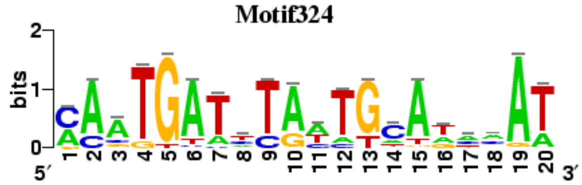	394.73	0.65	0.345	0.008
	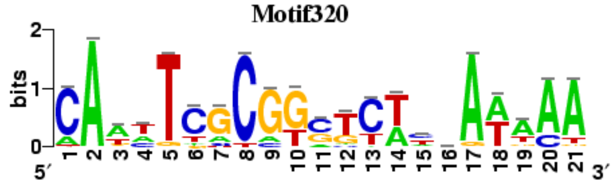	320.24	0.7	0.236	0.004
	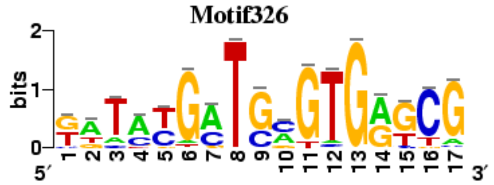	244.05	0.65	0.200	0.004
	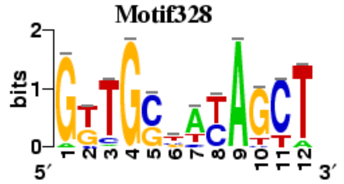	220.87	0.8	0.182	0.004
	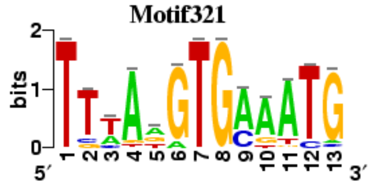	197.74	0.75	0.164	0.003
		530.22	0.7	0.630	0.025

	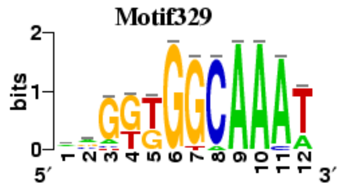				
	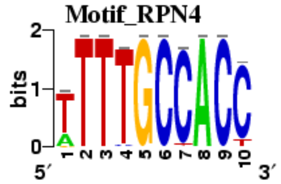	450.87	0.8	0.500	0.018
**28**	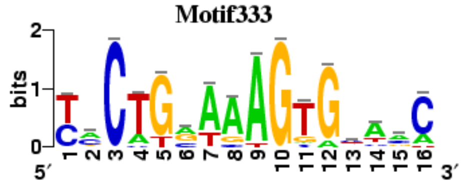	214.55	0.7	0.241	0.008
	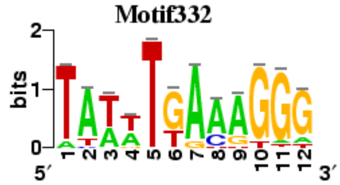	140.64	0.8	0.204	0.009
	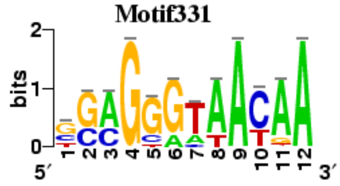	112.32	0.7	0.296	0.027
	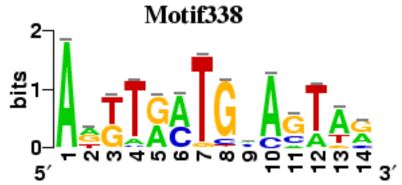	204.81	0.75	0.275	0.011
**29**	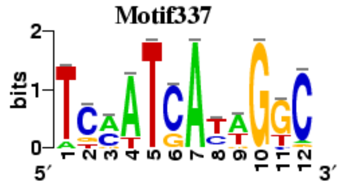	166.55	0.75	0.176	0.005
	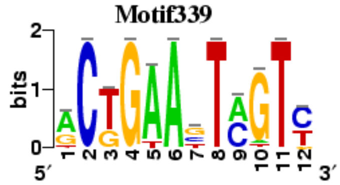	127.11	0.7	0.137	0.004
		51.82	0.9	0.039	0.000

	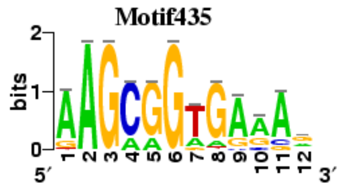				
	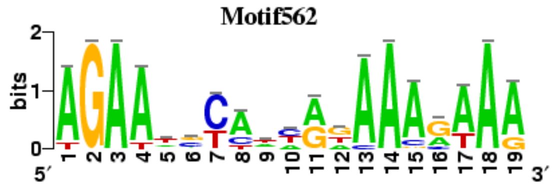	51.82	0.95	0.039	0.000
**30**	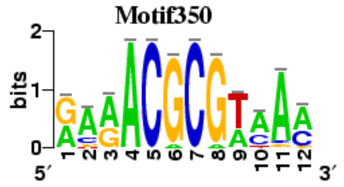	838.31	0.65	0.843	0.026
	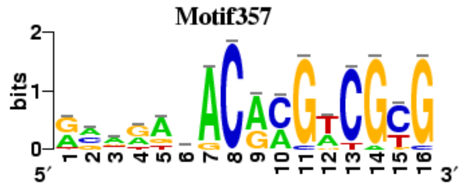	569.75	0.7	0.353	0.004
	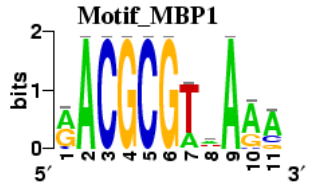	431.55	0.6	0.627	0.031
	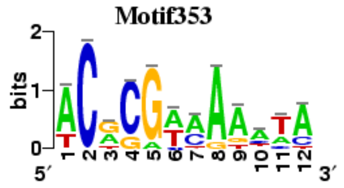	386.52	0.75	0.608	0.033
	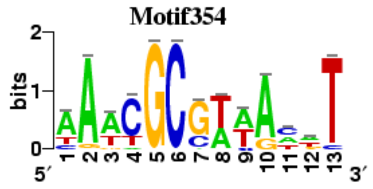	381.11	0.65	0.686	0.044
	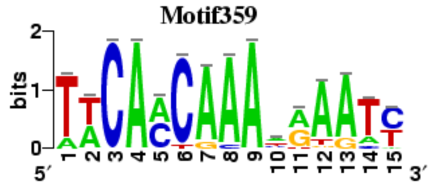	206.04	0.8	0.200	0.005
		188.16	0.8	0.240	0.009

31	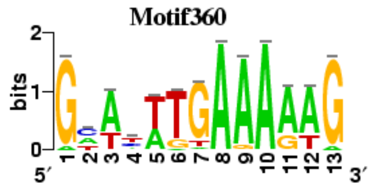				
	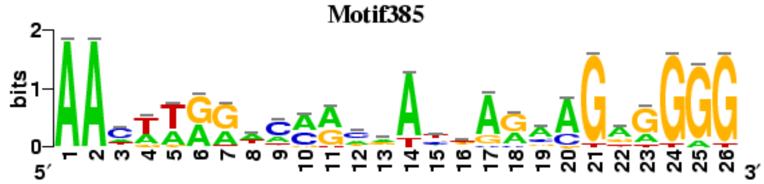	52.92	0.9	0.040	0.000
	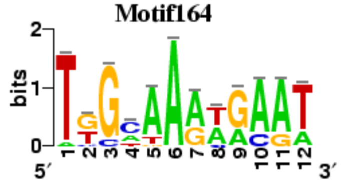	24.35	0.75	0.240	0.058
	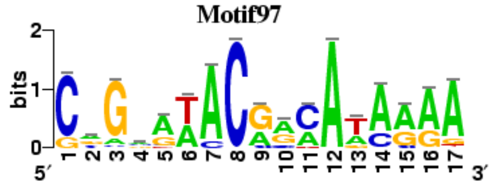	20.57	0.85	0.060	0.003
32	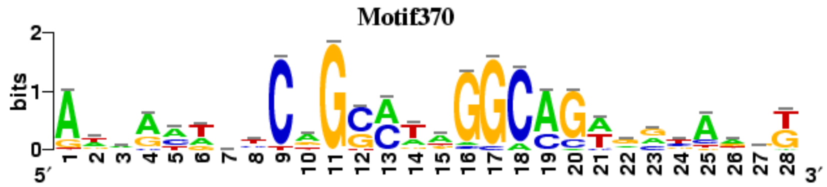	454.21	0.75	0.273	0.002
	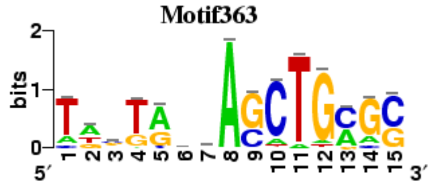	295.34	0.85	0.250	0.004
	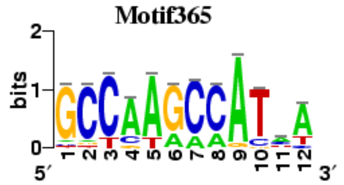	280.44	0.75	0.250	0.005
	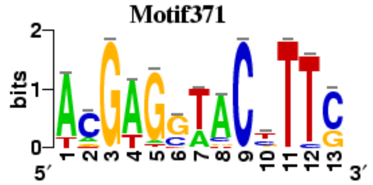	206.26	0.75	0.182	0.003
		204.40	0.7	0.227	0.006

	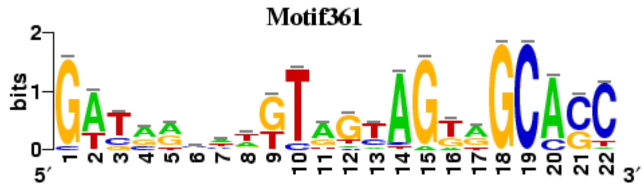				
33	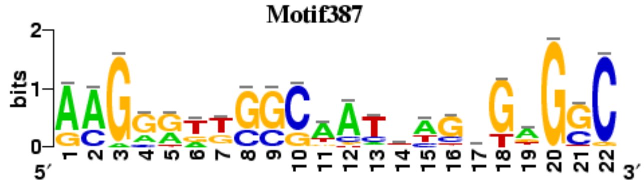	672.21	0.8	0.317	0.001
	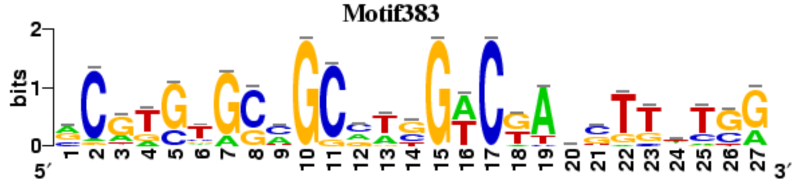	590.05	0.8	0.244	0.000
	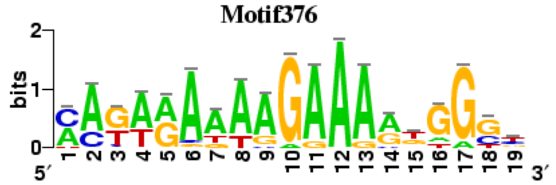	526.84	0.8	0.366	0.004
	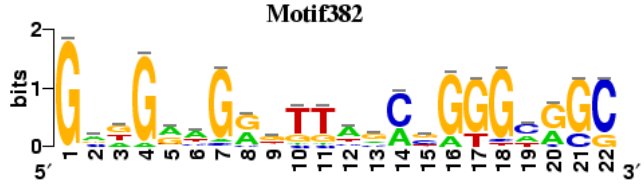	489.95	0.75	0.317	0.003
	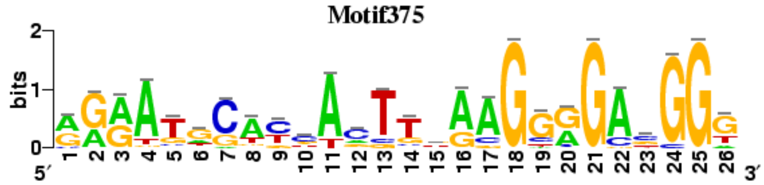	415.95	0.55	0.341	0.005
34	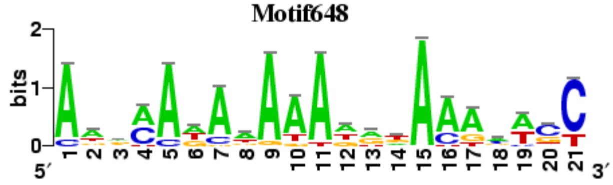	63.70	0.95	0.048	0.000
	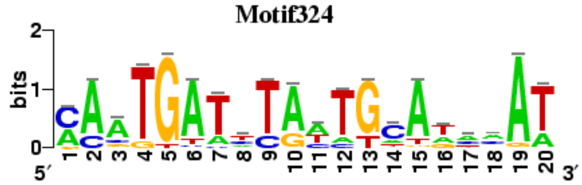	45.09	0.7	0.119	0.007
		40.34	0.8	0.119	0.007

	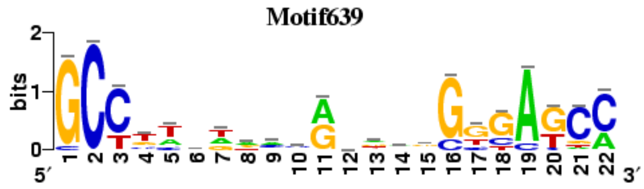				
	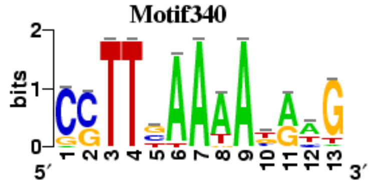	39.59	0.75	0.167	0.016
	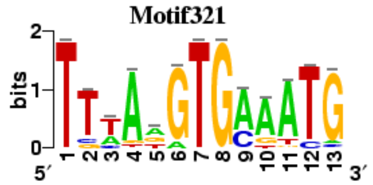	30.87	0.9	0.048	0.001
35	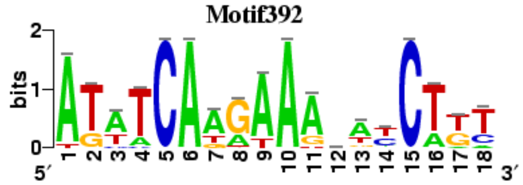	409.87	0.7	0.293	0.004
	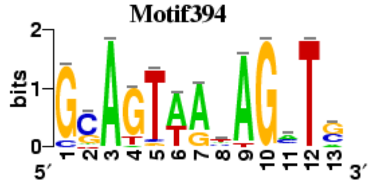	401.21	0.75	0.317	0.005
	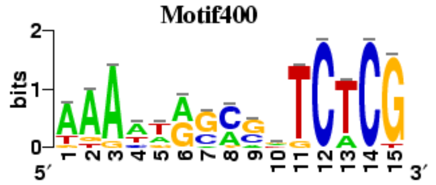	388.43	0.65	0.293	0.004
	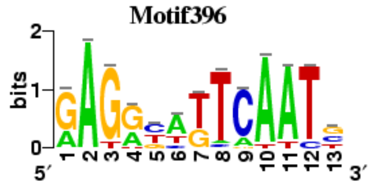	298.09	0.65	0.390	0.013
	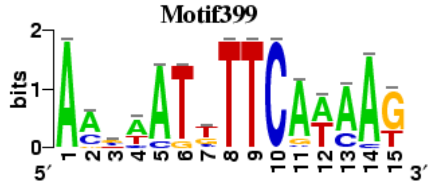	287.07	0.75	0.244	0.004
		224.63	0.55	0.231	0.005

	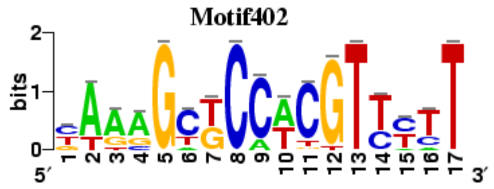				
	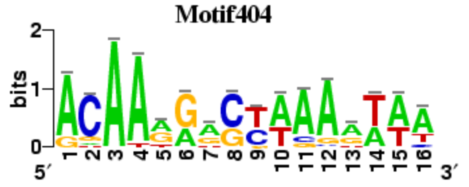	196.99	0.75	0.256	0.007
**36**	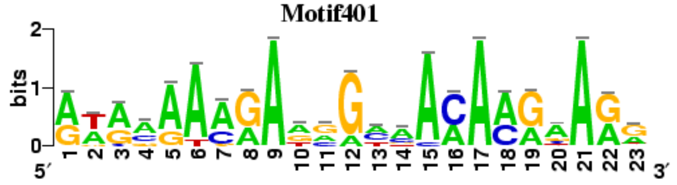	184.23	0.8	0.333	0.015
	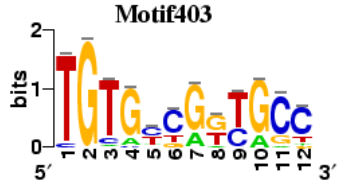	124.71	0.7	0.282	0.016
	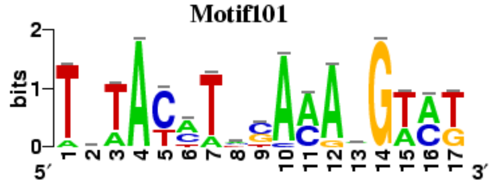	68.95	0.95	0.051	0.000
	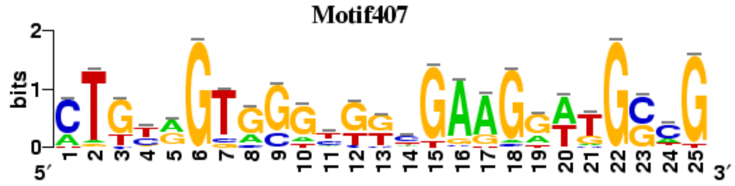	549.56	0.7	0.364	0.003
**37**	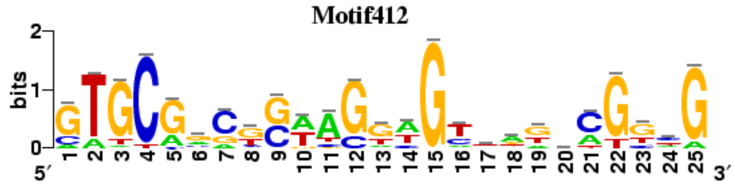	487.30	0.55	0.394	0.005
	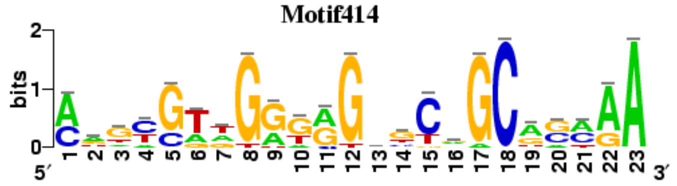	471.54	0.6	0.424	0.007
		471.54	0.7	0.424	0.007

	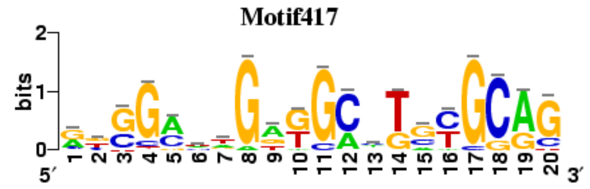				
	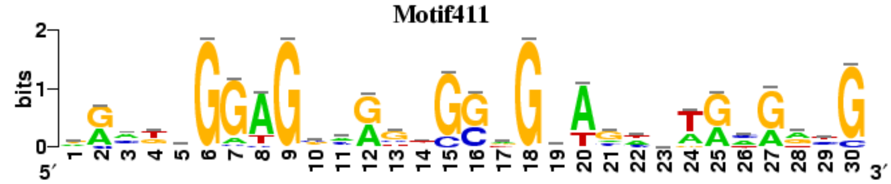	425.63	0.65	0.455	0.009
38	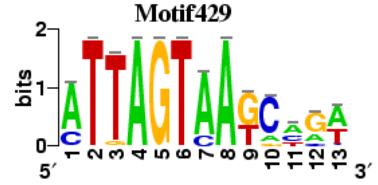	897.37	0.7	0.606	0.006
	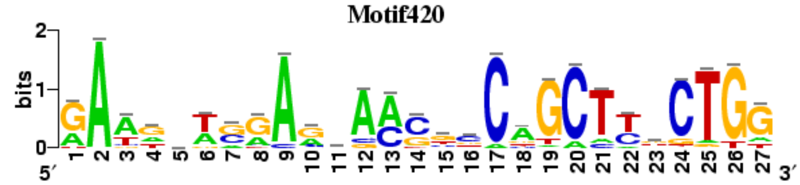	791.33	0.75	0.394	0.002
	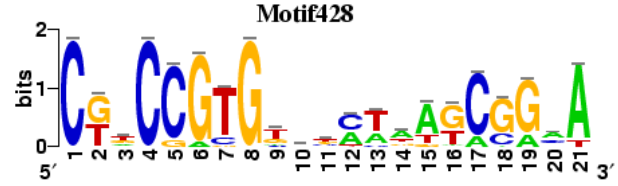	772.30	0.7	0.424	0.002
	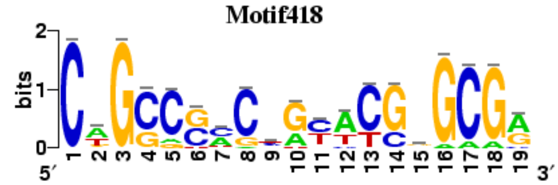	767.91	0.65	0.364	0.001
	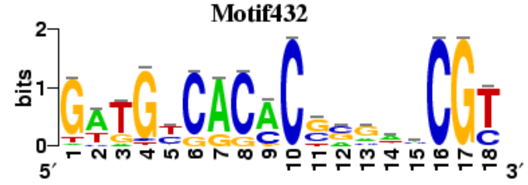	620.52	0.6	0.364	0.002
	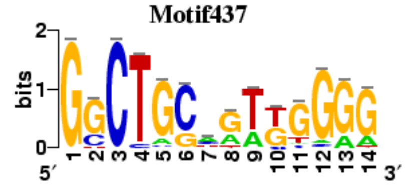	148.20	0.75	0.176	0.004
		144.24	0.75	0.206	0.005

39	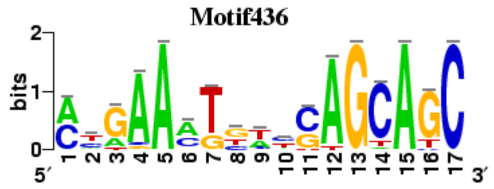				
	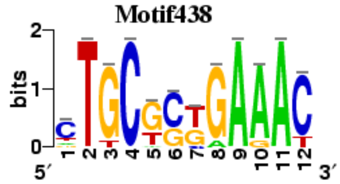	136.96	0.7	0.176	0.004
	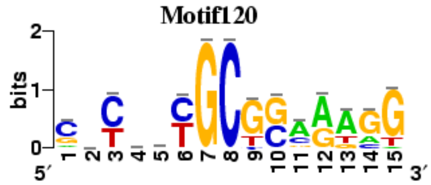	38.96	0.95	0.059	0.001
	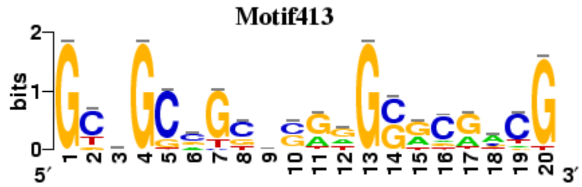	38.96	0.95	0.059	0.001
40	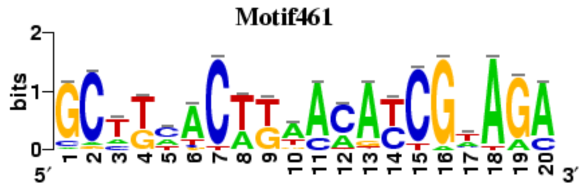	664.32	0.7	0.387	0.002
	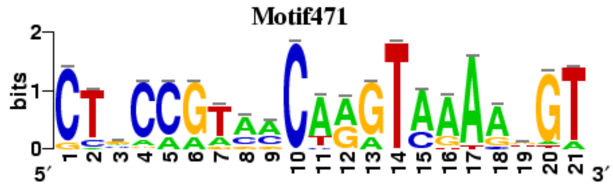	593.66	0.75	0.323	0.002
	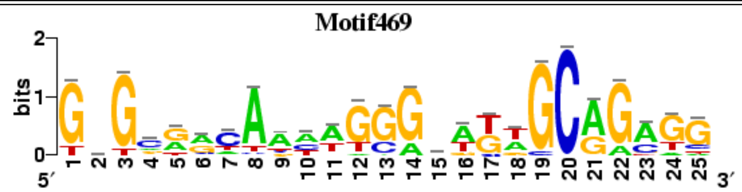	546.82	0.75	0.323	0.002
	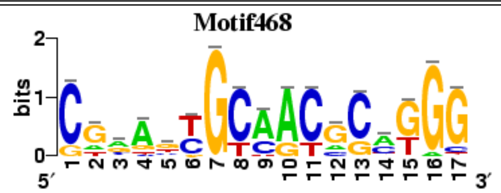	511.02	0.8	0.290	0.002
		484.56	0.65	0.355	0.004

	Motif470 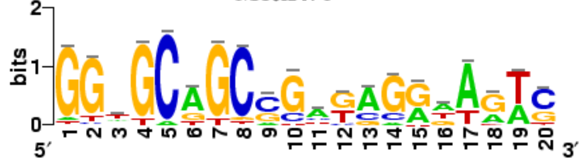				
41	Motif480 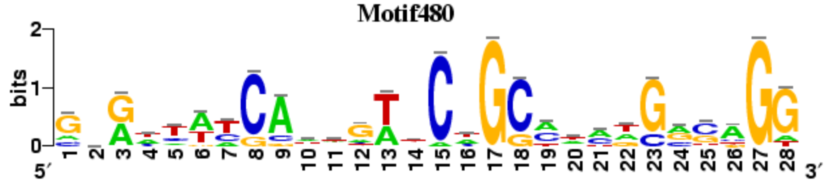	883.57	0.7	0.414	0.001
	Motif485 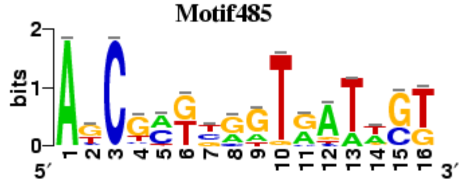	714.60	0.75	0.414	0.002
	Motif474 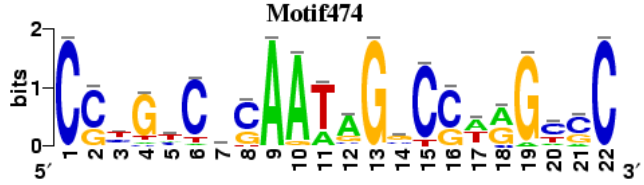	588.20	0.65	0.345	0.002
	Motif488 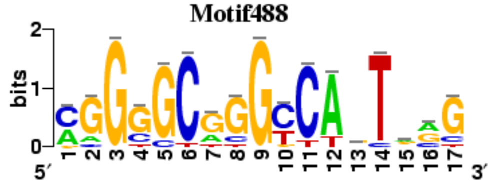	588.20	0.7	0.345	0.002
	Motif476 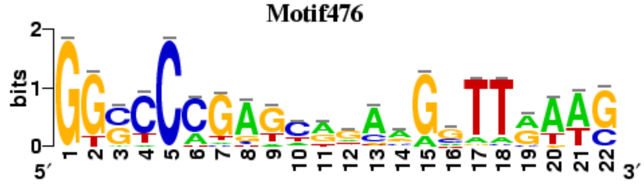	549.63	0.8	0.310	0.002
42	NA	NA	NA	NA	NA
	Motif509 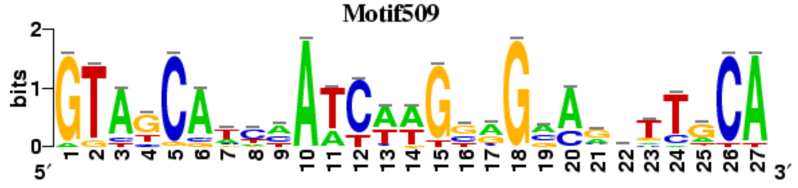	510.99	0.65	0.350	0.002
	Motif508 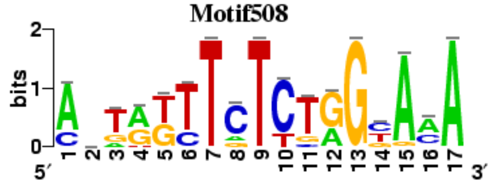	508.24	0.85	0.300	0.001

	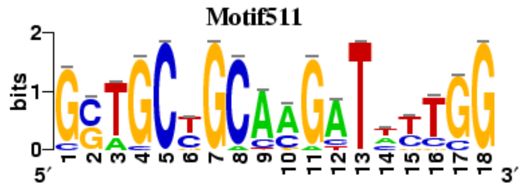	461.46	0.8	0.400	0.004
**43**	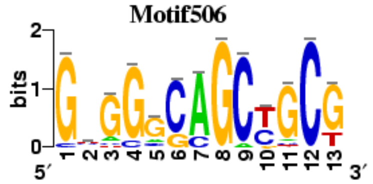	393.39	0.8	0.300	0.002
	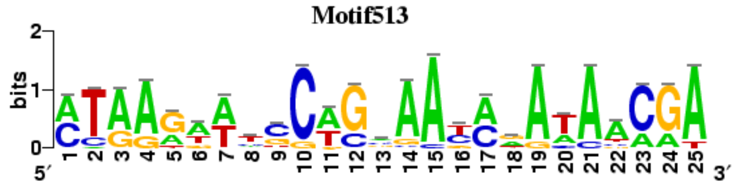	390.76	0.75	0.350	0.003
	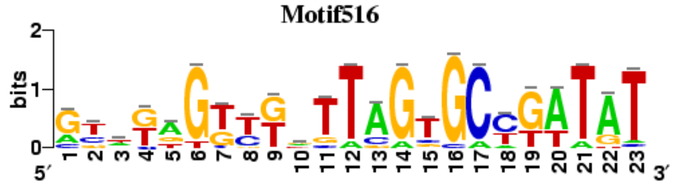	1897.91	0.55	0.800	0.001
	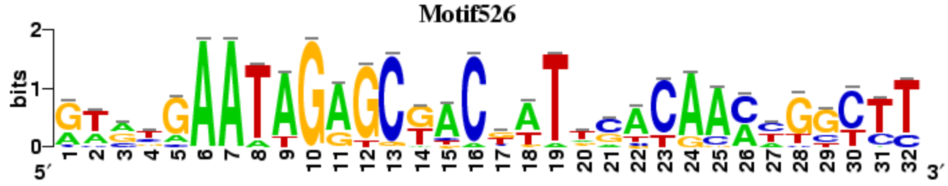	1897.91	0.5	0.800	0.001
**44**	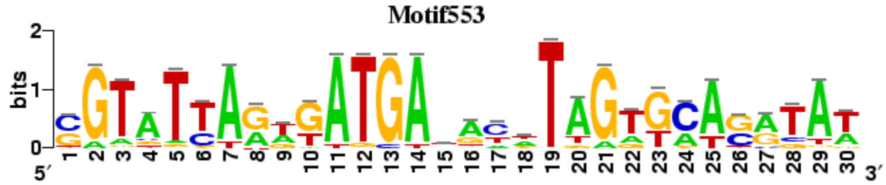	1897.91	0.65	0.800	0.001
	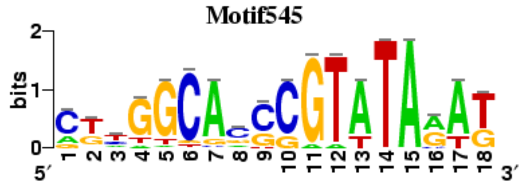	1834.63	0.7	0.850	0.002
	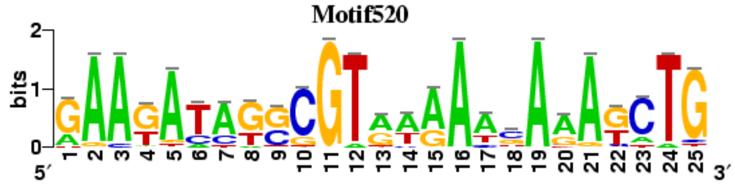	1791.50	0.5	0.800	0.002
		969.17	0.7	0.800	0.007

	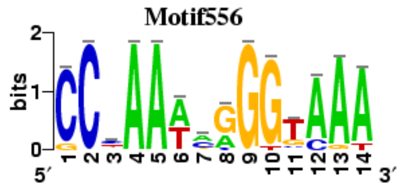				
	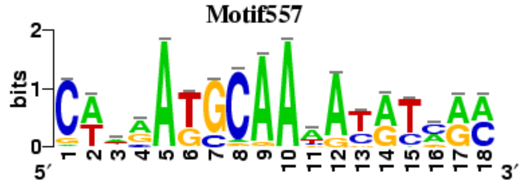	697.84	0.9	0.400	0.002
**45**	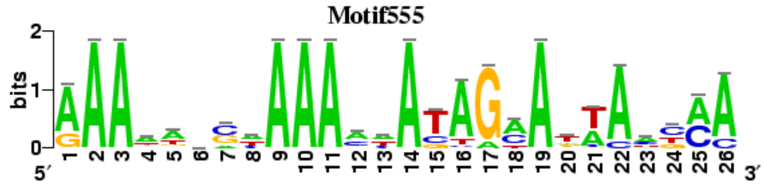	313.84	0.6	0.700	0.024
	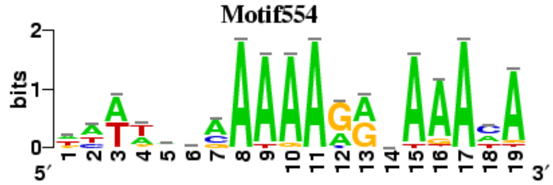	227.92	0.85	0.600	0.024
	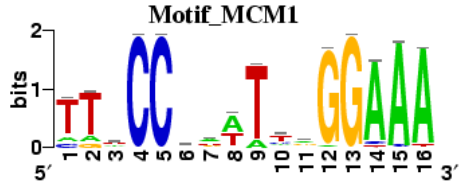	162.07	0.75	0.550	0.028
	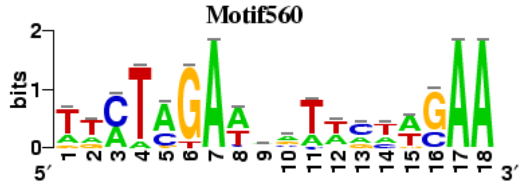	766.15	0.75	0.765	0.008
**46**	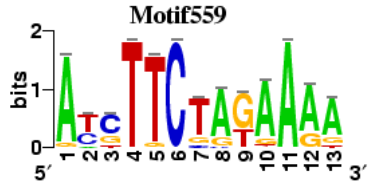	485.77	0.8	0.706	0.012
	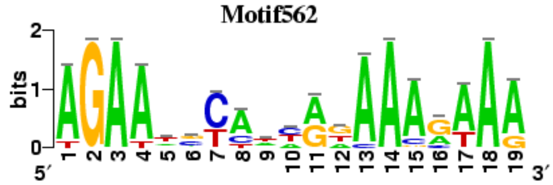	485.77	0.75	0.706	0.012
		384.90	0.75	0.235	0.001

	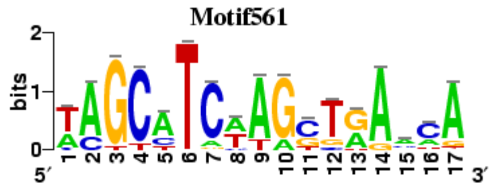				
	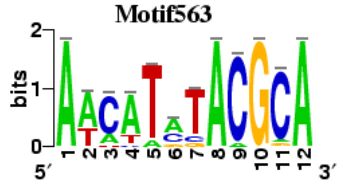	282.36	0.7	0.471	0.009
	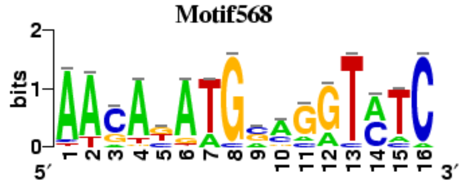	1370.59	0.8	0.833	0.004
	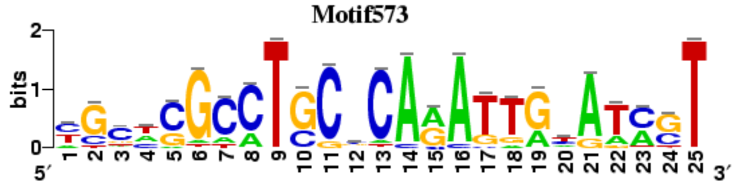	1359.23	0.65	0.778	0.003
47	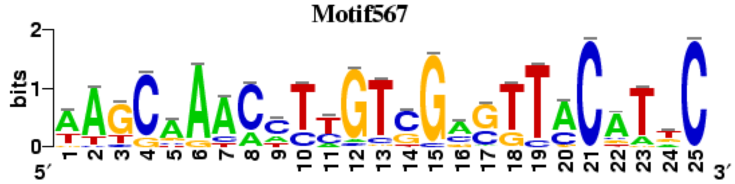	1293.77	0.75	0.778	0.003
	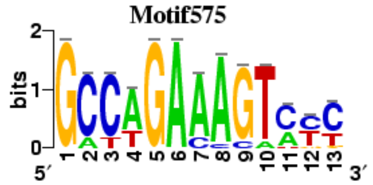	1179.92	0.75	0.778	0.004
	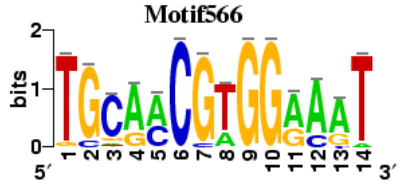	1100.53	0.9	0.722	0.004
	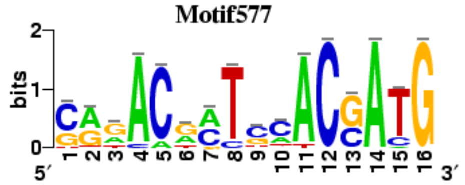	935.62	0.8	0.900	0.006
		935.62	0.75	0.900	0.006

48	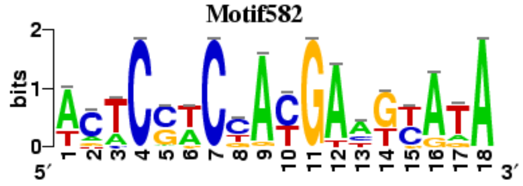				
	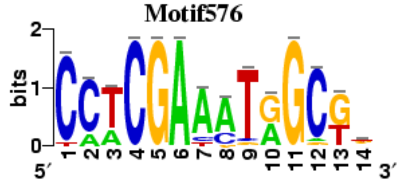	894.59	0.75	0.900	0.006
	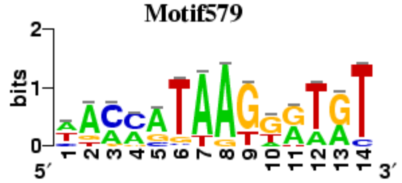	856.98	0.85	0.900	0.007
	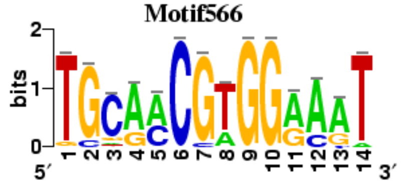	770.99	0.95	0.700	0.004
49	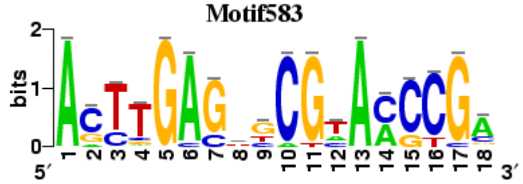	976.36	0.65	0.462	0.001
	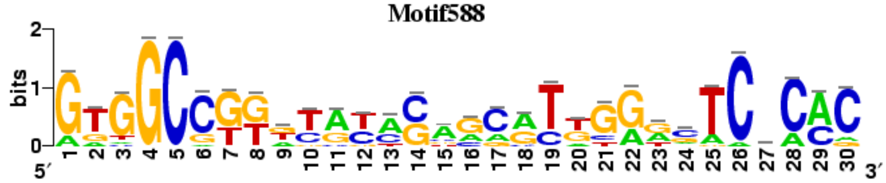	938.18	0.6	0.385	0.000
	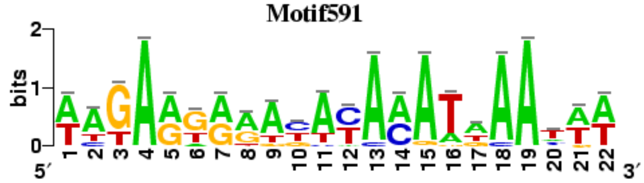	841.51	0.8	0.538	0.002
	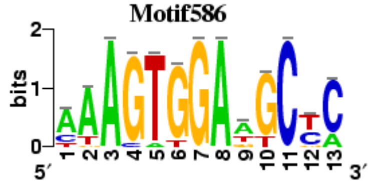	725.13	0.7	0.769	0.007
		558.10	0.75	0.538	0.004

	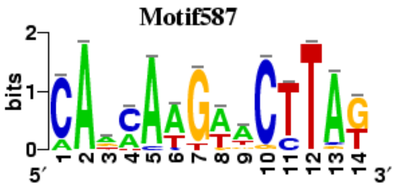Motif587<br>bits<br>5′<br>3′				
