# Supplementary material for: Predicting Gene Expression from Sequence: A Reexamination
Source: PLoS Comput Biol. 2007 Nov 30;3(11):e243. doi: 10.1371/journal.pcbi.0030243 (PMC2098866; doi:10.1371/journal.pcbi.0030243)
Supplement: Figure S3 — In each CV, a set of motifs are generated using the training set only. The known 51 motifs are included too. (5.4 MB PDF) [file pcbi.0030243.sg003.pdf]

Figure S3.

Top 5 motifs selected in each cluster in five-fold cross-validation. In each cross-validation, a set of motifs are generated using the training set only. The known 51 motifs are included too.

CV01:

Cluster	Motif	Chi-square statistic	Score cutoff	Posterior probability within cluster	Posterior probability outside cluster
1	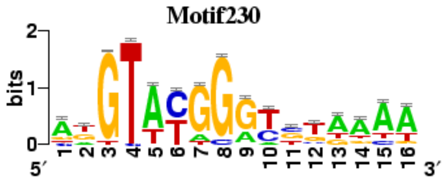	810.08	0.6	0.608	0.015
	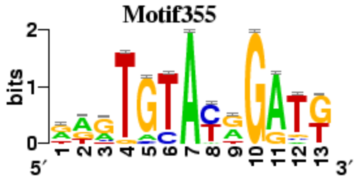	689.50	0.65	0.686	0.032
	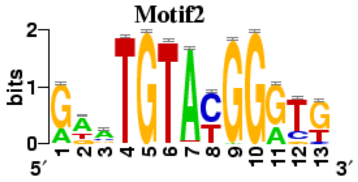	660.15	0.5	0.627	0.027
	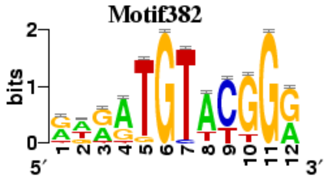	432.86	0.6	0.676	0.063
	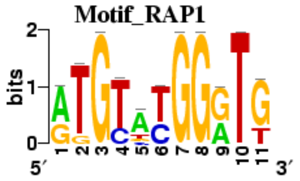	381.53	0.8	0.490	0.034
	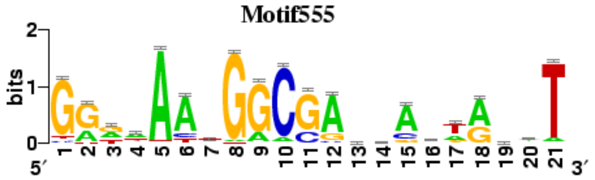	239.28	0.75	0.172	0.003
	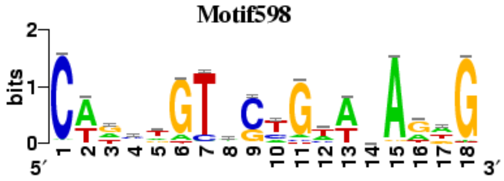	175.03	0.8	0.108	0.001

	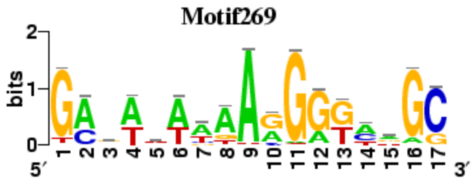	170.67	0.8	0.129	0.003
2	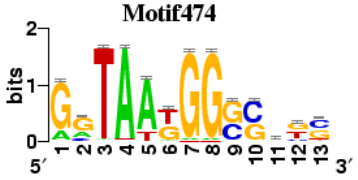	170.67	0.85	0.129	0.003
	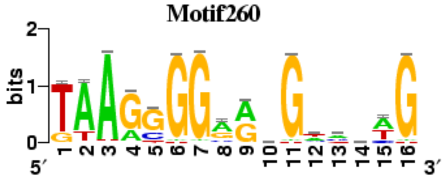	153.39	0.85	0.097	0.001
	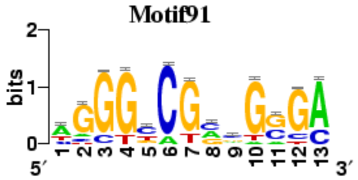	200.31	0.7	0.241	0.012
	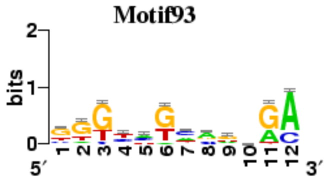	109.47	0.9	0.103	0.003
3	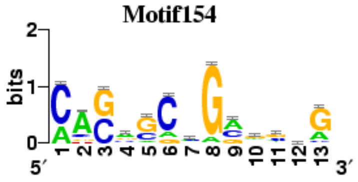	107.93	0.75	0.207	0.019
	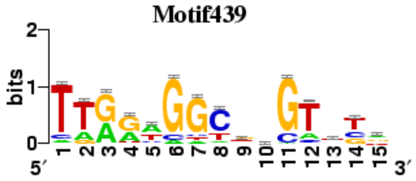	95.82	0.9	0.069	0.001
	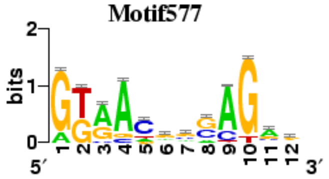	43.00	0.55	0.724	0.375
		312.45	0.75	0.686	0.083

4	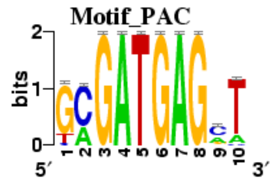				
	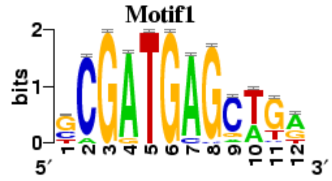	275.93	0.5	0.709	0.101
	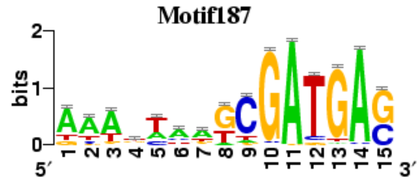	264.08	0.6	0.709	0.106
	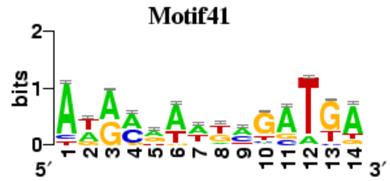	246.14	0.8	0.360	0.025
	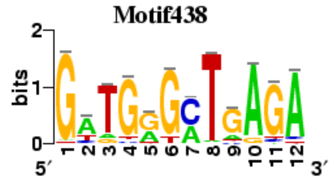	173.96	0.65	0.395	0.046
5	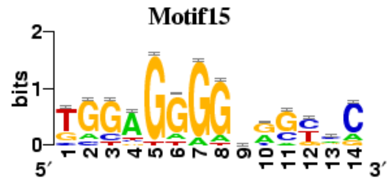	231.47	0.7	0.304	0.014
	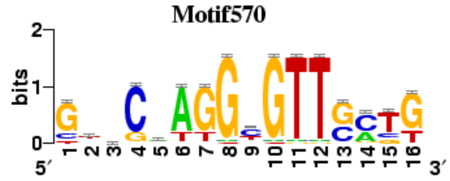	209.07	0.7	0.159	0.002
	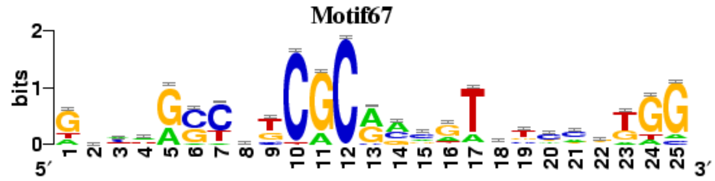	206.58	0.7	0.174	0.003
		195.96	0.55	0.362	0.027

	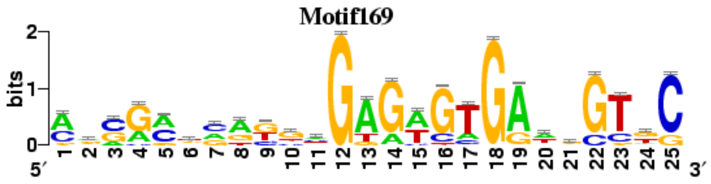				
	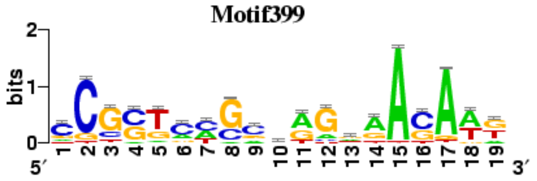	107.50	0.6	0.449	0.079
6	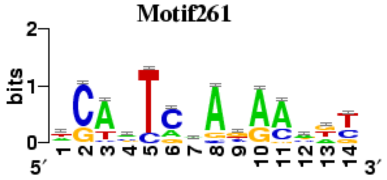	209.07	0.85	0.159	0.002
	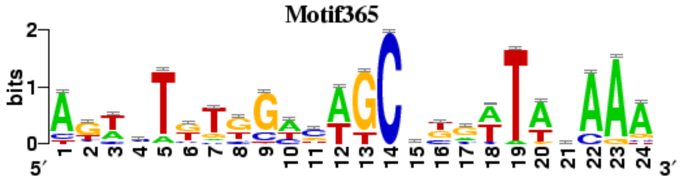	143.28	0.55	0.304	0.026
	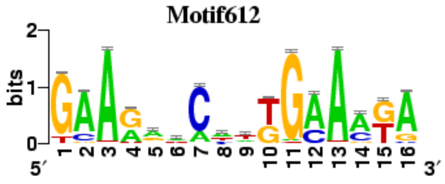	142.86	0.85	0.116	0.002
	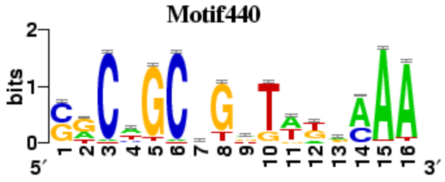	135.07	0.75	0.145	0.004
	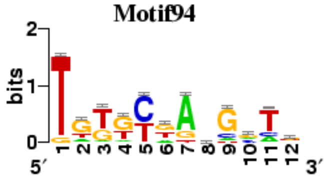	89.73	0.95	0.058	0.000
	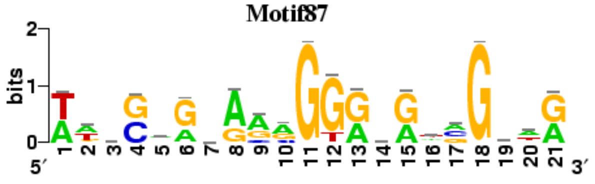	221.19	0.75	0.294	0.014
		208.36	0.55	0.250	0.010

7	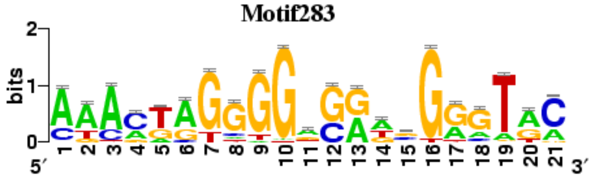				
	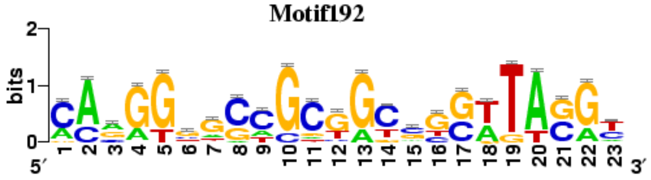	170.28	0.65	0.147	0.003
	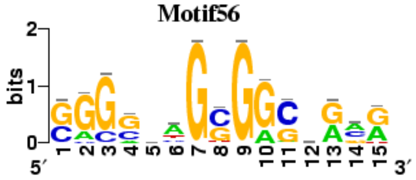	164.47	0.75	0.250	0.014
	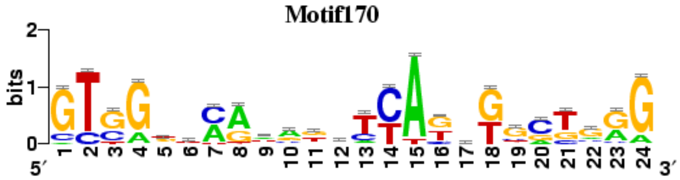	152.91	0.6	0.206	0.009
8	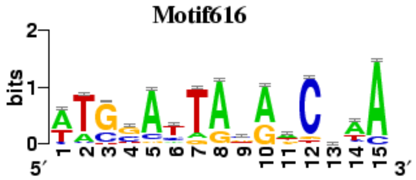	420.72	0.85	0.258	0.002
	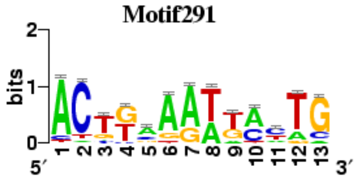	351.88	0.85	0.212	0.001
	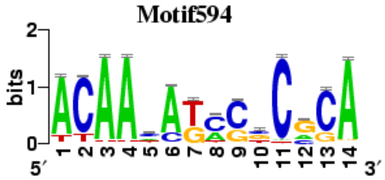	203.61	0.8	0.167	0.003
	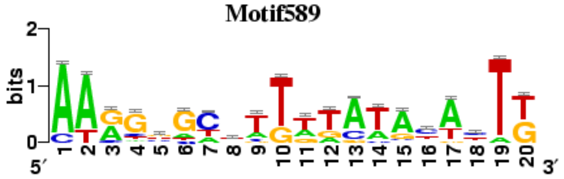	144.02	0.65	0.242	0.015
		122.81	0.85	0.121	0.003

	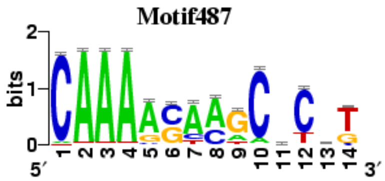				
9	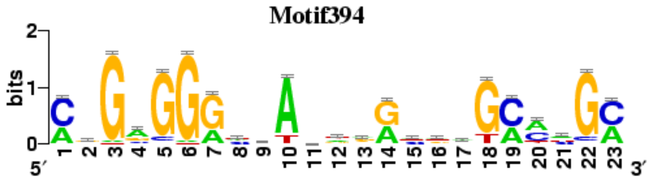	281.58	0.75	0.190	0.002
	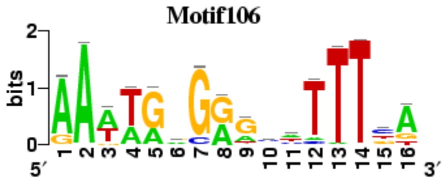	197.04	0.8	0.317	0.018
	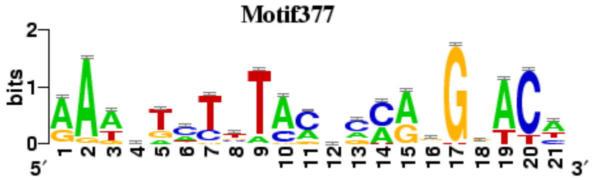	188.09	0.8	0.143	0.002
	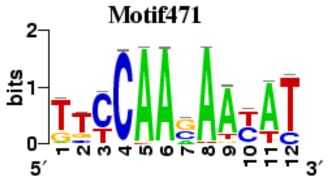	188.09	0.9	0.143	0.002
	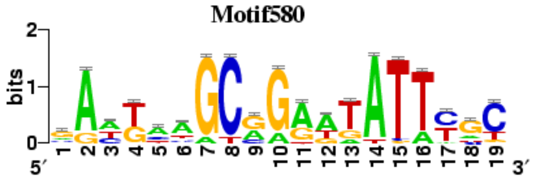	171.69	0.65	0.206	0.007
10	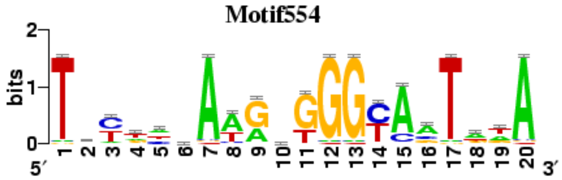	311.46	0.75	0.232	0.003
	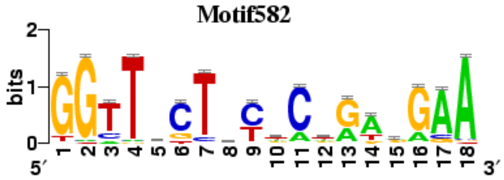	297.17	0.7	0.214	0.002
		283.45	0.7	0.429	0.021

	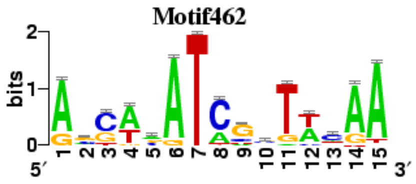				
	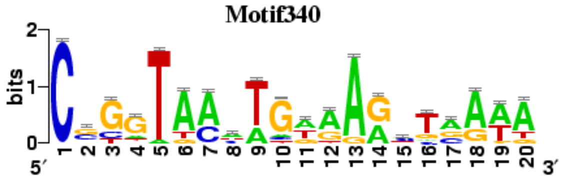	259.89	0.75	0.214	0.003
	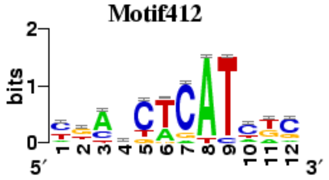	186.20	0.65	0.804	0.136
	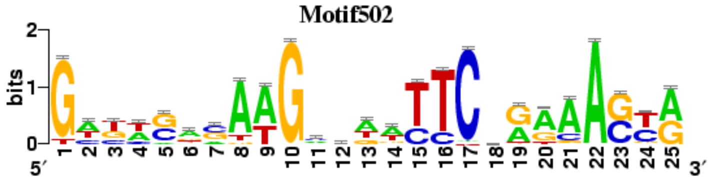	650.51	0.6	0.371	0.002
	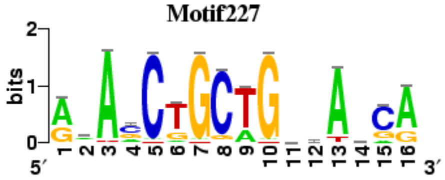	372.64	0.8	0.258	0.003
**11**	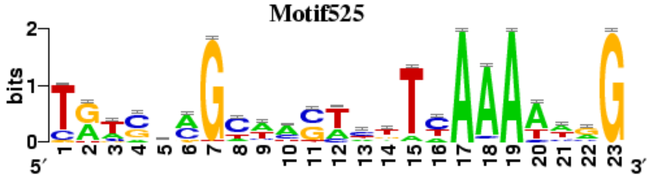	309.73	0.6	0.226	0.003
	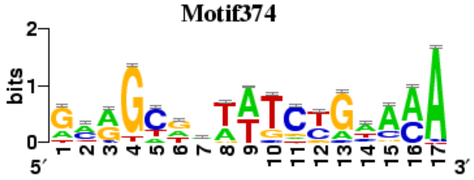	296.43	0.8	0.274	0.006
	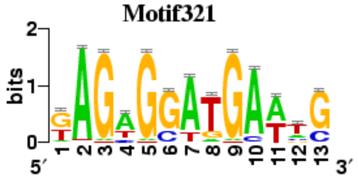	276.44	0.75	0.226	0.004
		232.52	0.7	0.250	0.007

	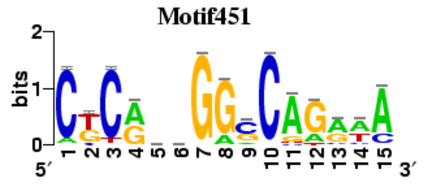				
	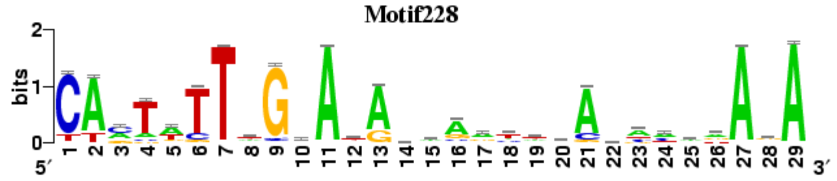	230.46	0.75	0.214	0.004
**12**	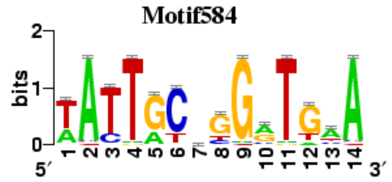	227.57	0.8	0.143	0.001
	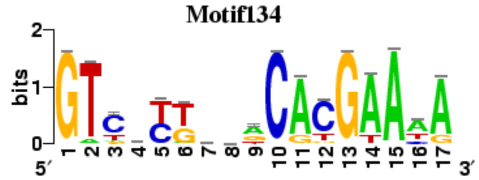	227.42	0.75	0.196	0.003
	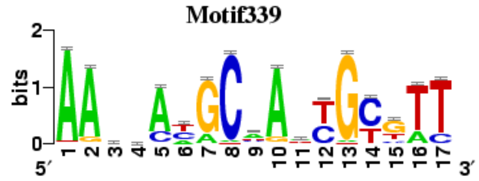	210.83	0.8	0.179	0.003
	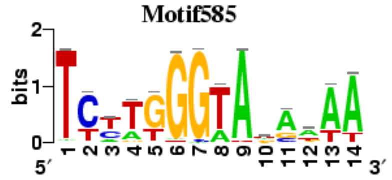	191.01	0.8	0.158	0.002
**13**	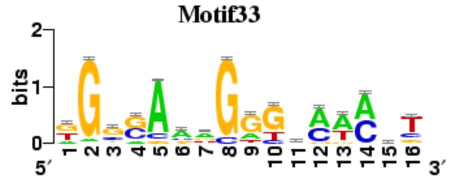	36.62	0.95	0.035	0.000
	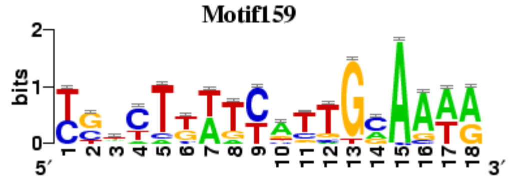	36.62	0.95	0.035	0.000
		36.62	0.95	0.035	0.000

	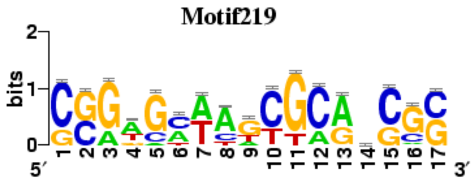				
	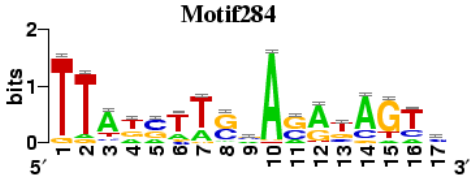	36.62	0.85	0.035	0.000
	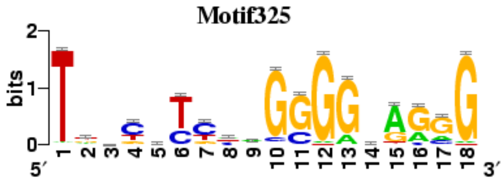	339.42	0.75	0.211	0.001
	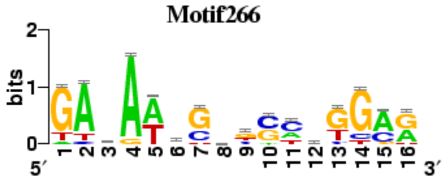	319.37	0.85	0.263	0.004
**14**	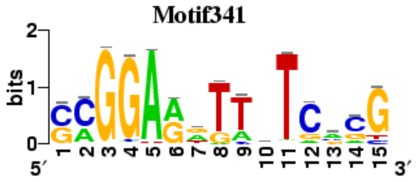	303.17	0.7	0.246	0.004
	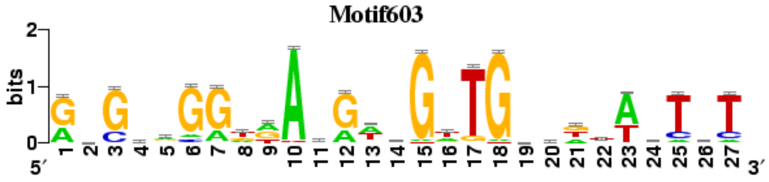	296.09	0.8	0.175	0.001
	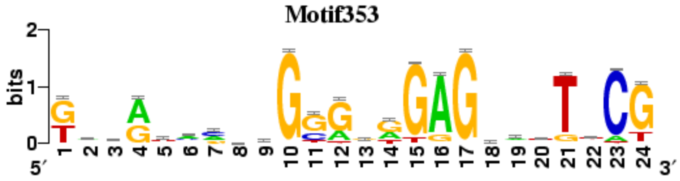	293.94	0.8	0.158	0.000
	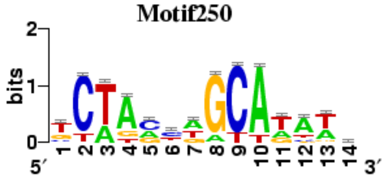	424.58	0.85	0.333	0.005
		338.90	0.75	0.278	0.004

15	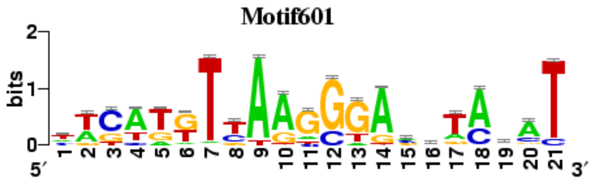				
	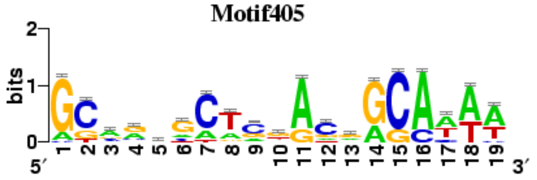	273.59	0.65	0.333	0.011
	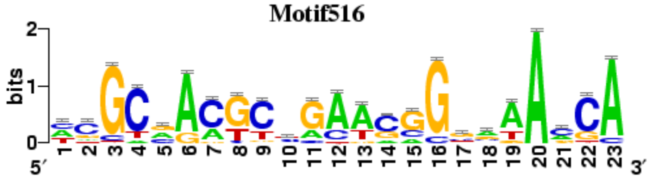	261.10	0.65	0.278	0.007
	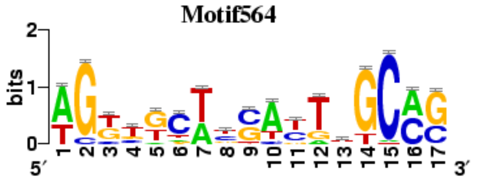	227.67	0.65	0.333	0.015
16	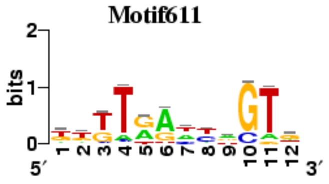	99.07	0.85	0.236	0.018
	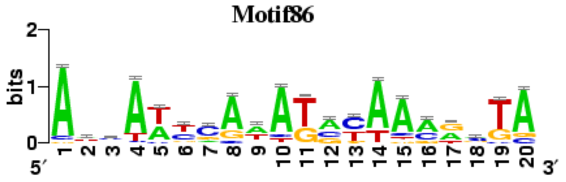	76.11	0.95	0.055	0.000
	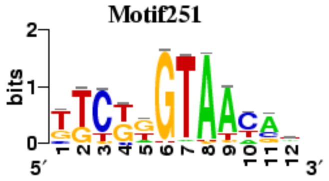	42.64	0.85	0.164	0.019
	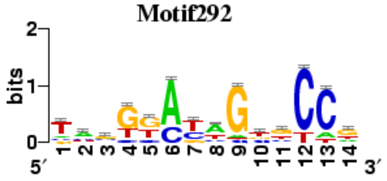	38.04	0.95	0.036	0.000
		38.04	0.95	0.036	0.000

	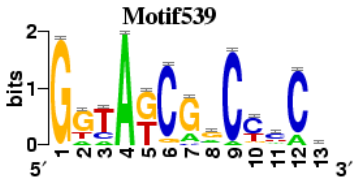				
	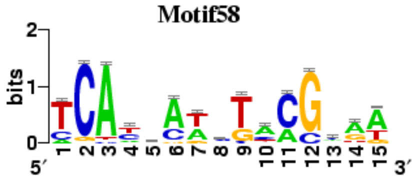	114.26	0.9	0.151	0.005
	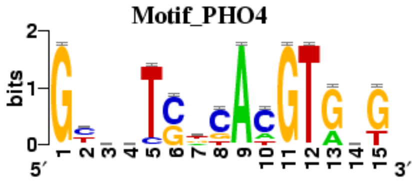	79.17	0.95	0.057	0.000
17	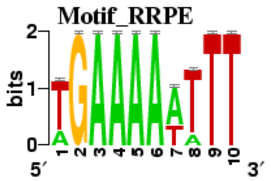	63.25	0.8	0.604	0.172
	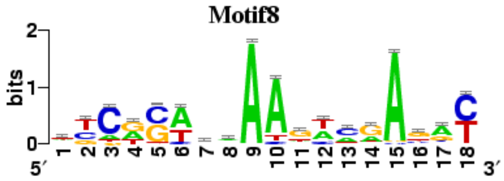	51.48	0.95	0.057	0.001
	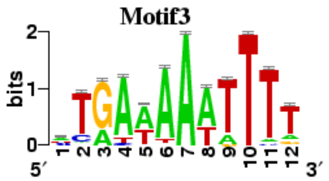	46.15	0.6	0.736	0.300
	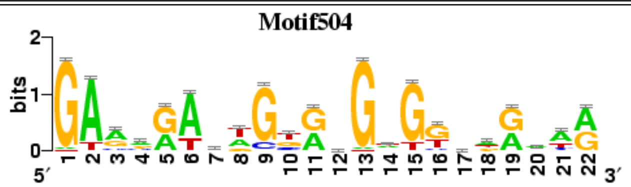	144.21	0.75	0.234	0.010
18	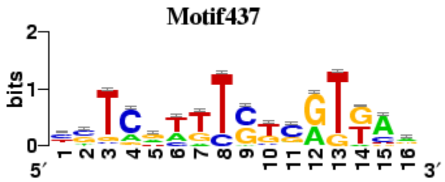	130.93	0.75	0.170	0.005
		122.10	0.7	0.191	0.007

	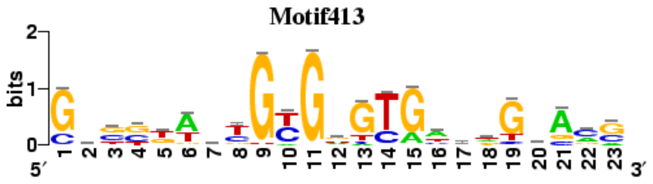				
	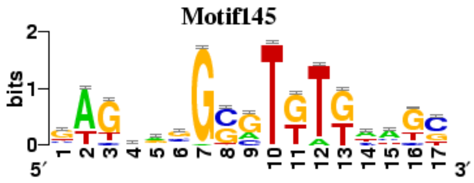	121.00	0.9	0.128	0.002
	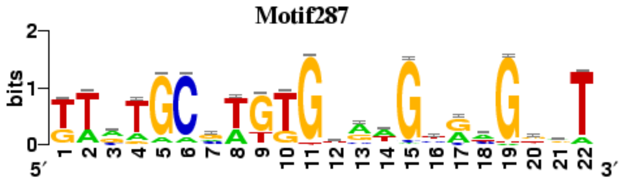	96.86	0.65	0.191	0.010
	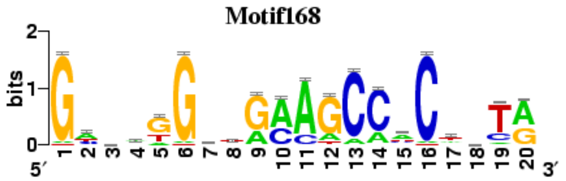	351.29	0.75	0.250	0.002
	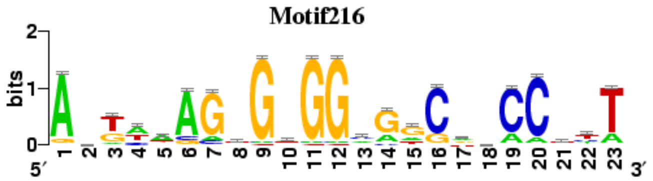	322.13	0.75	0.208	0.001
**19**	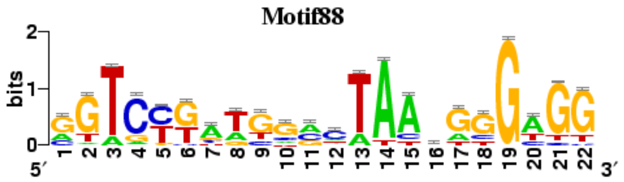	252.75	0.65	0.188	0.002
	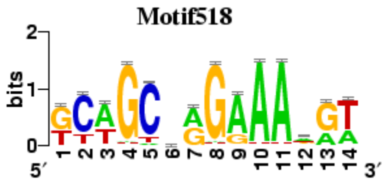	230.47	0.8	0.188	0.002
	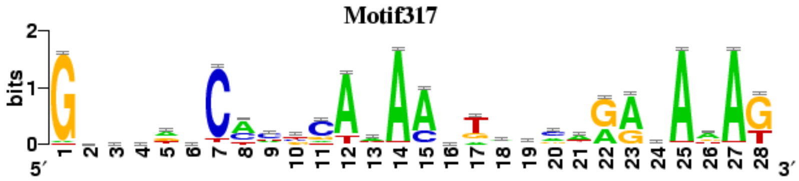	228.20	0.7	0.292	0.009
		355.56	0.75	0.298	0.004

20	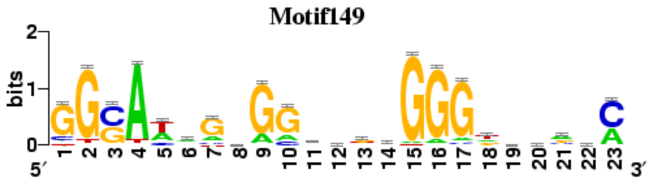				
	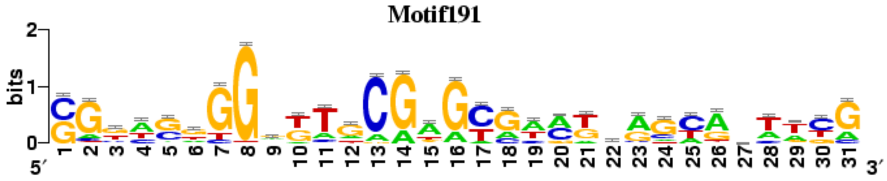	300.71	0.6	0.213	0.002
	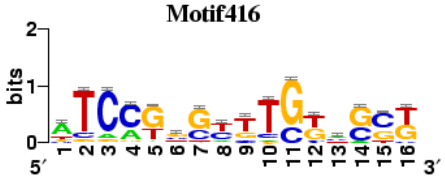	300.71	0.85	0.213	0.002
	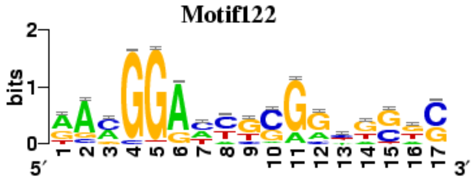	295.22	0.8	0.234	0.003
	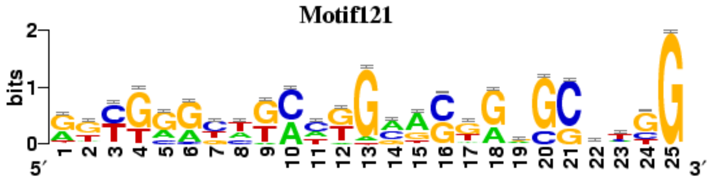	295.12	0.55	0.298	0.006
21	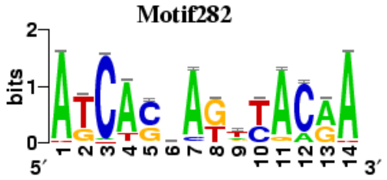	243.01	0.85	0.217	0.003
	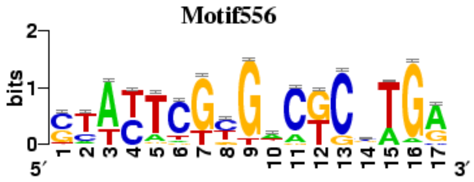	160.10	0.65	0.239	0.009
	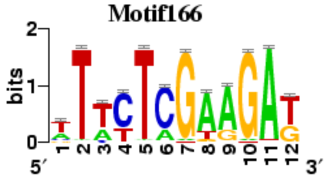	146.78	0.75	0.261	0.012
		118.02	0.75	0.217	0.010

	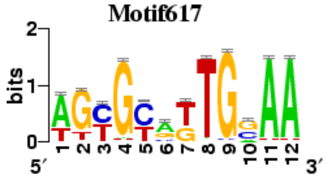				
	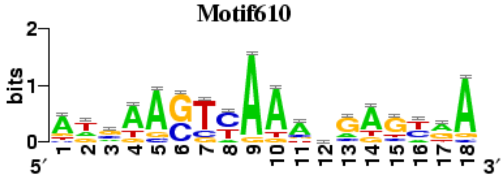	107.22	0.7	0.261	0.018
	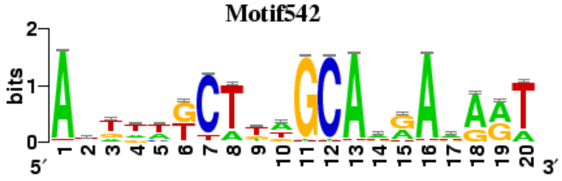	240.69	0.75	0.244	0.005
	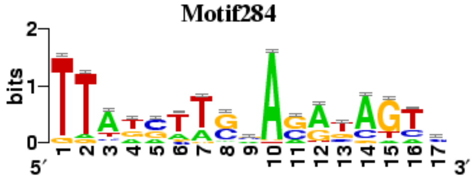	111.68	0.7	0.244	0.014
22	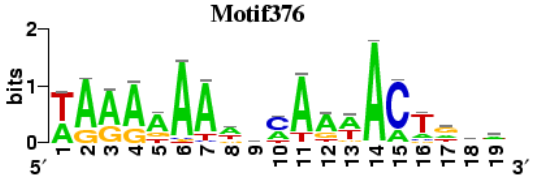	58.90	0.85	0.200	0.018
	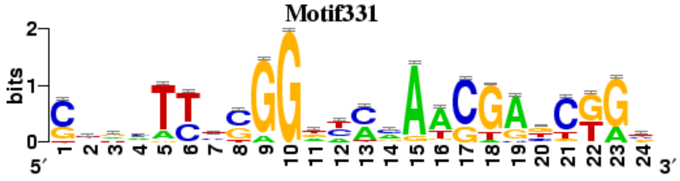	47.12	0.75	0.044	0.000
	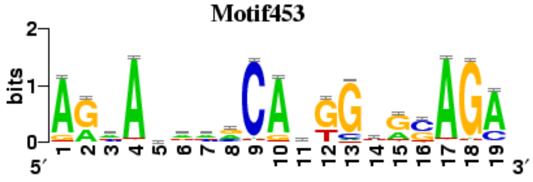	22.58	0.95	0.044	0.001
	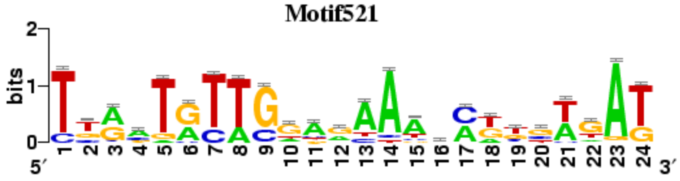	287.81	0.8	0.178	0.001
		236.60	0.8	0.333	0.012

23	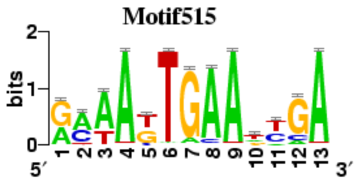				
	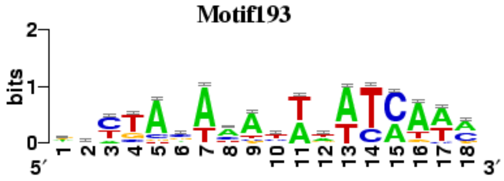	149.46	0.85	0.111	0.001
	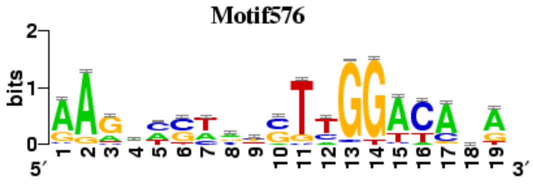	141.48	0.85	0.089	0.000
	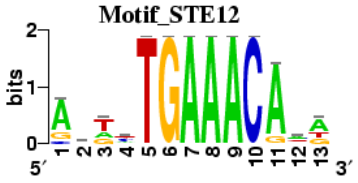	61.55	0.95	0.067	0.001
24	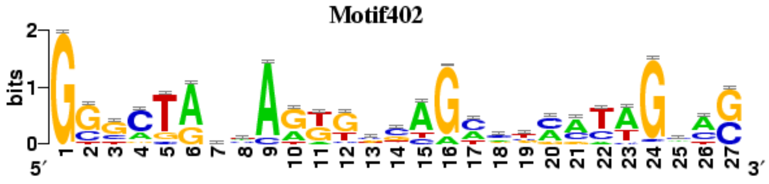	461.55	0.65	0.262	0.001
	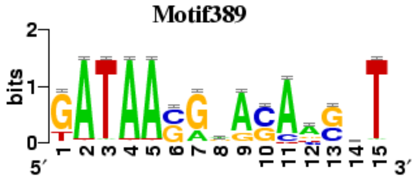	372.05	0.75	0.238	0.001
	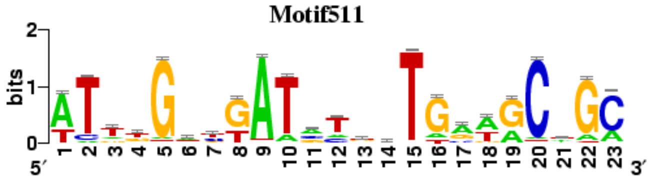	372.05	0.7	0.238	0.001
	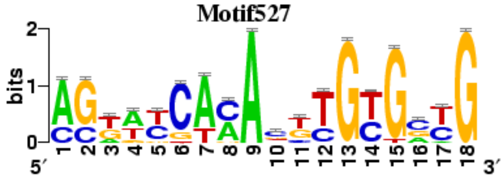	358.88	0.5	0.262	0.002
		335.23	0.7	0.286	0.004

	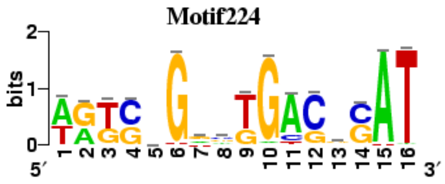				
25	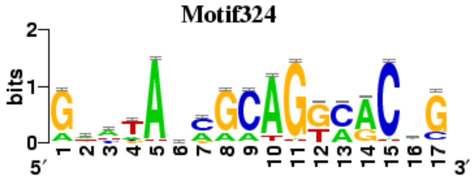	245.02	0.7	0.214	0.003
	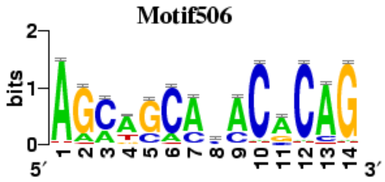	122.39	0.75	0.143	0.003
	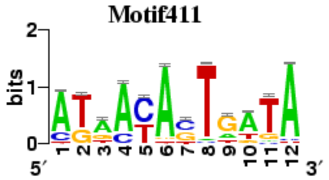	92.02	0.9	0.143	0.004
	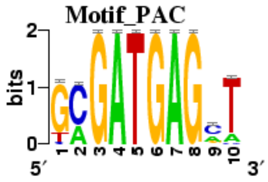	74.09	0.65	0.571	0.118
	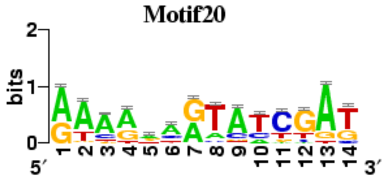	73.06	0.85	0.143	0.006
26	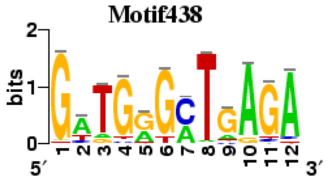	115.70	0.7	0.386	0.037
	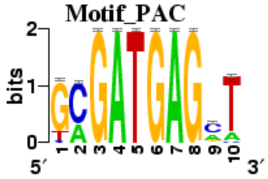	96.94	0.75	0.568	0.098
		64.60	0.75	0.682	0.190

	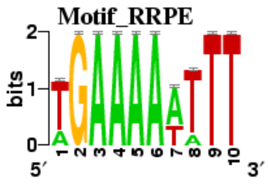				
	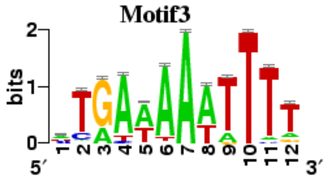	55.24	0.9	0.364	0.064
	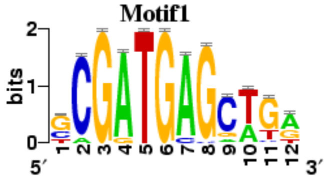	54.63	0.5	0.500	0.118
	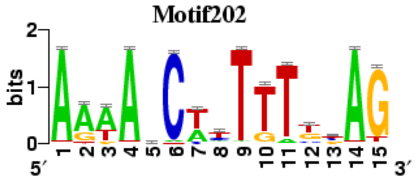	300.63	0.8	0.289	0.005
	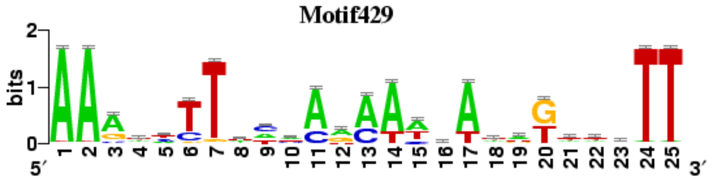	274.19	0.8	0.311	0.008
**27**	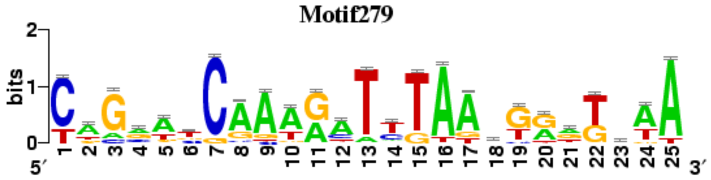	240.69	0.7	0.244	0.005
	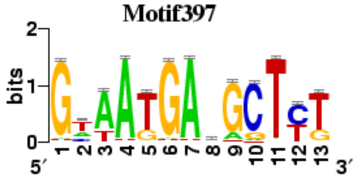	205.81	0.8	0.178	0.002
	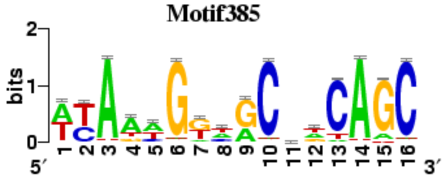	165.91	0.85	0.133	0.001
		415.01	0.5	0.659	0.030

	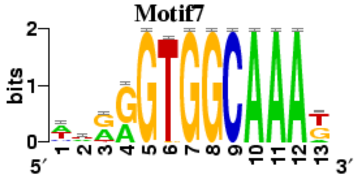				
	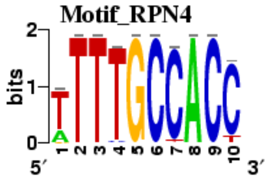	315.84	0.8	0.477	0.019
28	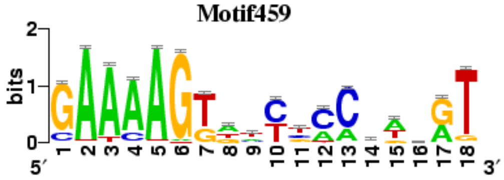	277.79	0.8	0.205	0.002
	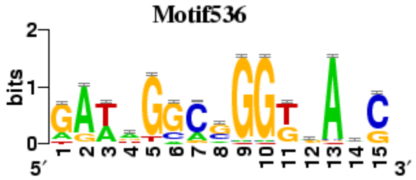	257.16	0.7	0.273	0.006
	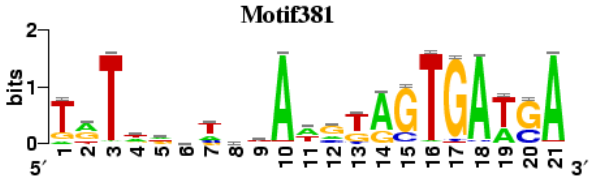	253.43	0.8	0.205	0.002
	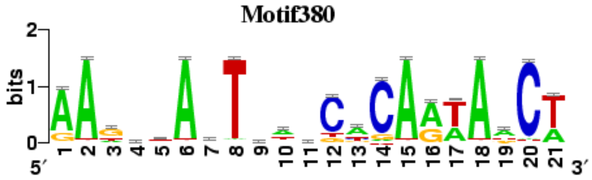	369.79	0.75	0.220	0.001
	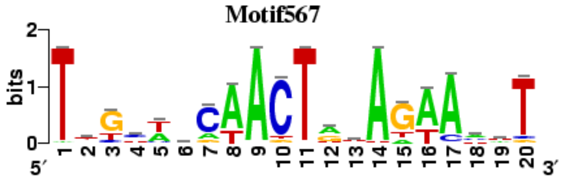	275.78	0.75	0.244	0.003
29	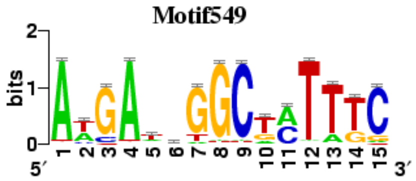	232.02	0.8	0.171	0.001
		86.61	0.8	0.317	0.031

	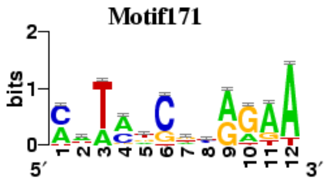				
	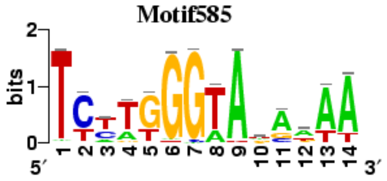	52.05	0.95	0.049	0.000
	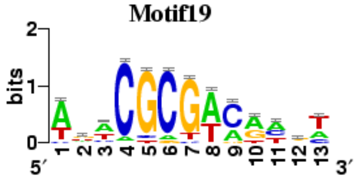	722.44	0.7	0.756	0.017
	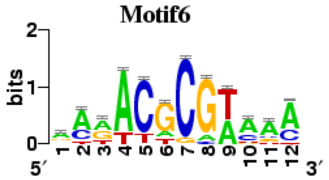	669.69	0.8	0.707	0.016
**30**	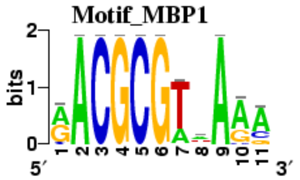	553.64	0.8	0.610	0.015
	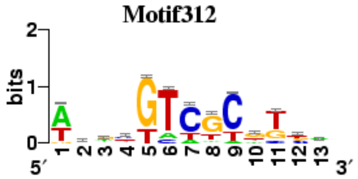	470.28	0.9	0.244	0.000
	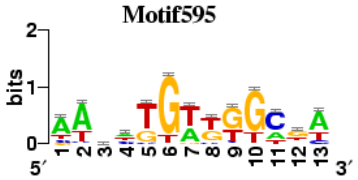	216.14	0.85	0.220	0.004
	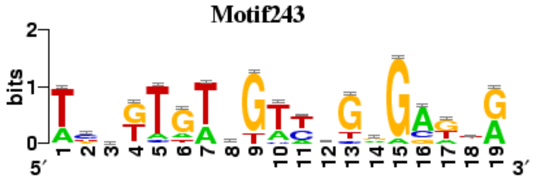	299.94	0.85	0.220	0.002
		273.75	0.7	0.220	0.002

31	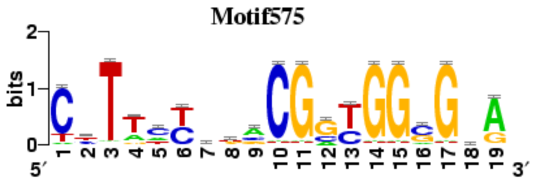				
	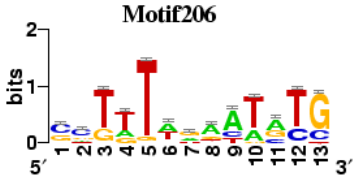	103.23	0.9	0.146	0.004
	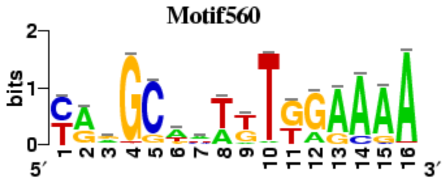	50.14	0.95	0.073	0.001
	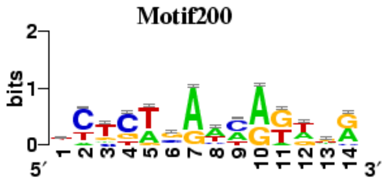	27.03	0.9	0.073	0.003
32	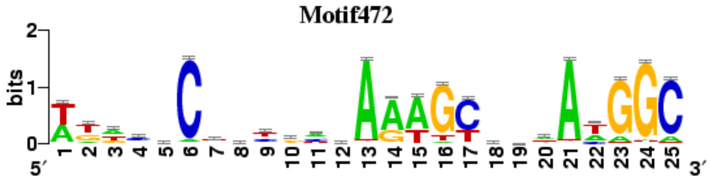	395.05	0.8	0.306	0.003
	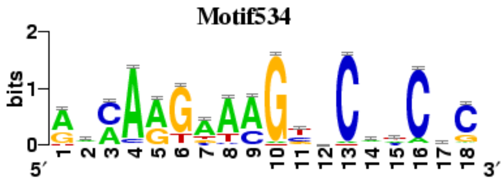	227.52	0.7	0.361	0.012
	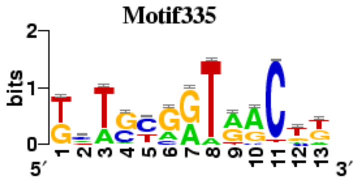	181.09	0.7	0.472	0.030
	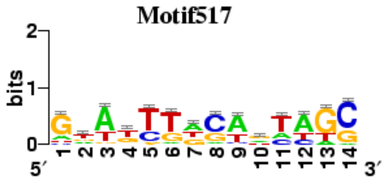	165.76	0.85	0.250	0.007
		59.85	0.95	0.056	0.000

	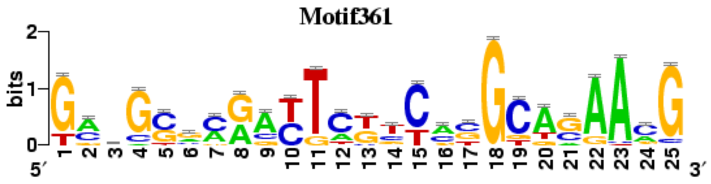				
33	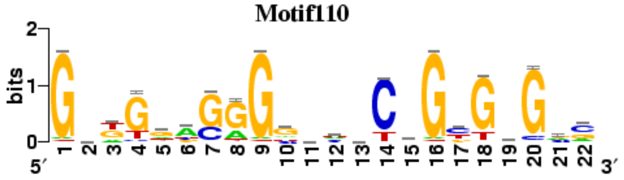	395.41	0.75	0.212	0.000
	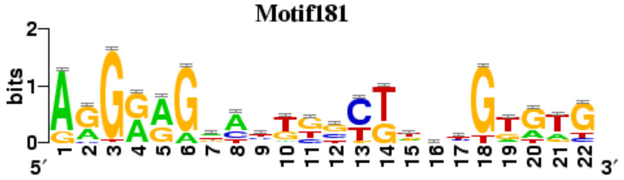	329.34	0.7	0.182	0.000
	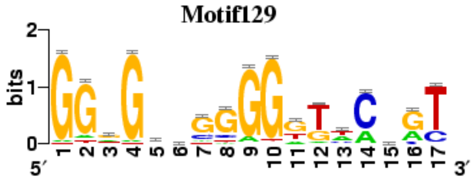	290.97	0.65	0.333	0.006
	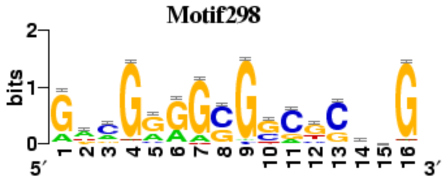	264.05	0.85	0.242	0.003
	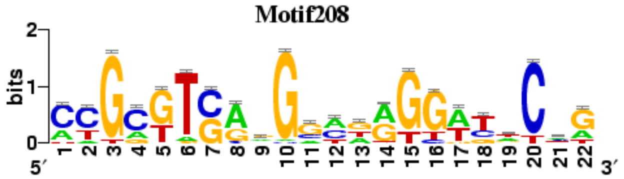	194.84	0.55	0.333	0.011
34	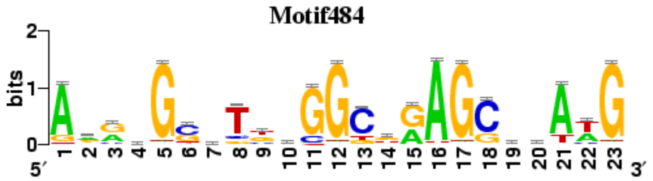	529.96	0.75	0.324	0.001
	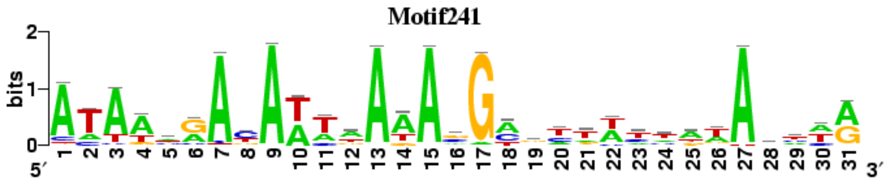	454.42	0.7	0.441	0.007
		427.46	0.9	0.294	0.002

	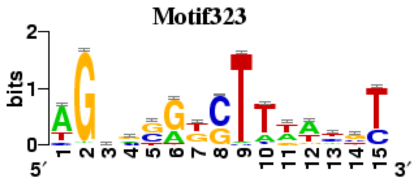				
	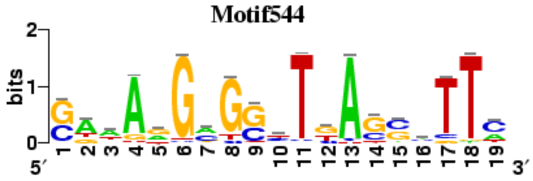	369.11	0.7	0.324	0.004
	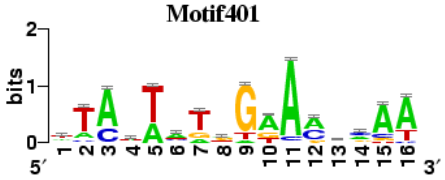	316.73	0.9	0.294	0.004
35	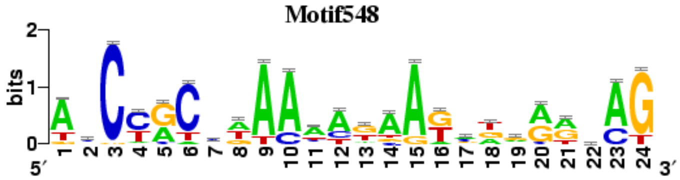	391.49	0.8	0.364	0.005
	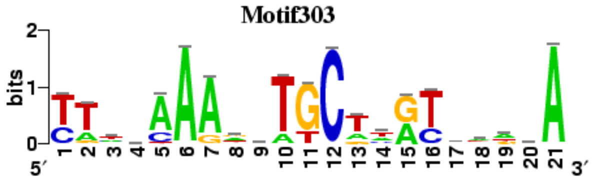	288.67	0.7	0.424	0.012
	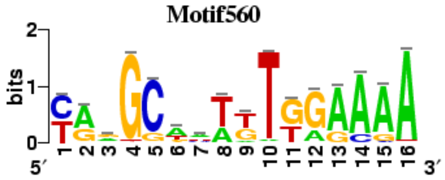	258.82	0.85	0.303	0.006
	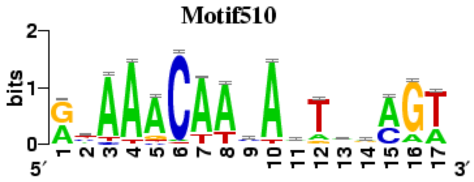	251.87	0.75	0.424	0.015
	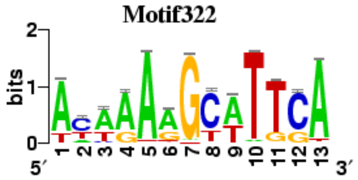	230.16	0.7	0.364	0.011
		448.96	0.65	0.290	0.001

36	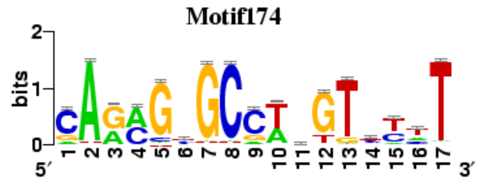				
	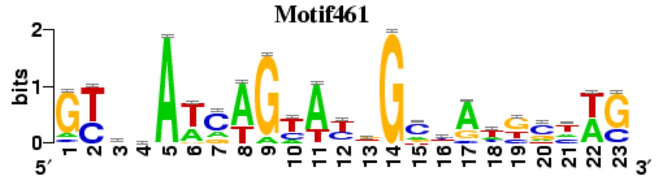	173.78	0.6	0.355	0.014
	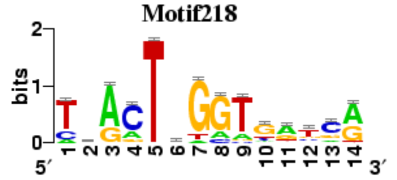	157.65	0.75	0.290	0.010
	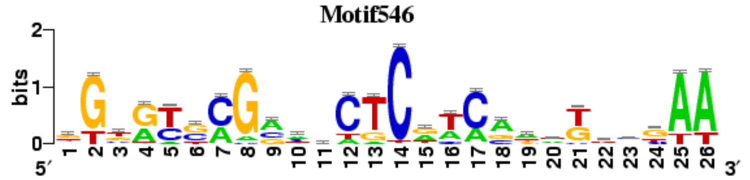	70.34	0.8	0.065	0.000
	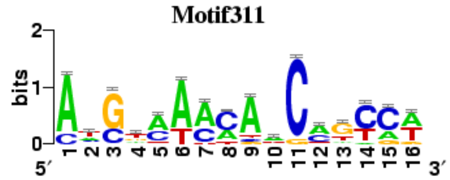	63.94	0.7	0.452	0.066
37	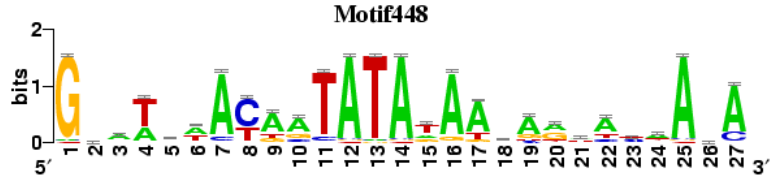	506.84	0.8	0.370	0.002
	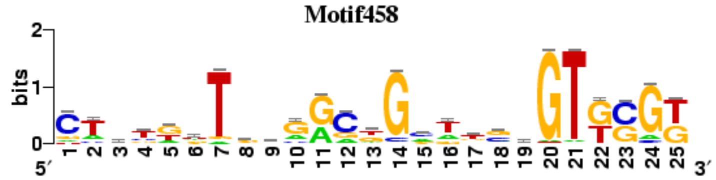	476.99	0.7	0.556	0.010
	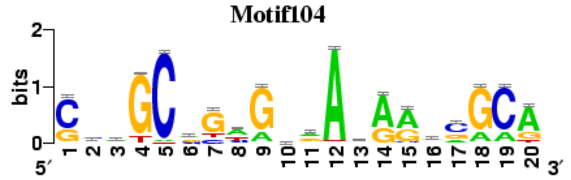	419.98	0.9	0.259	0.001
		289.78	0.8	0.444	0.011

	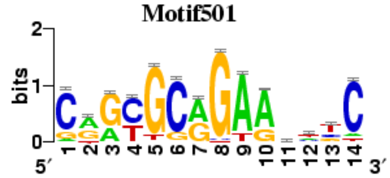				
	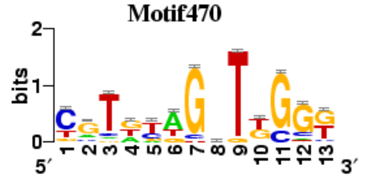	254.45	0.85	0.333	0.006
	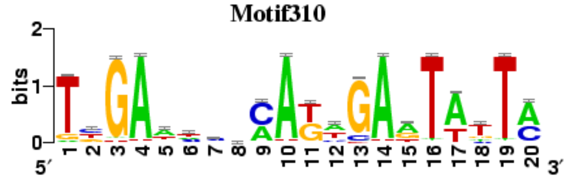	738.69	0.8	0.370	0.000
	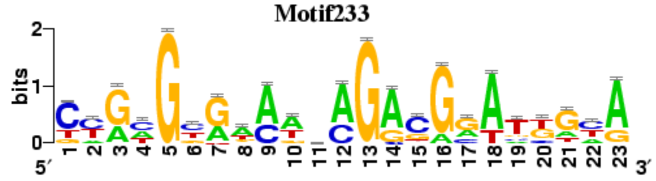	706.37	0.7	0.444	0.002
**38**	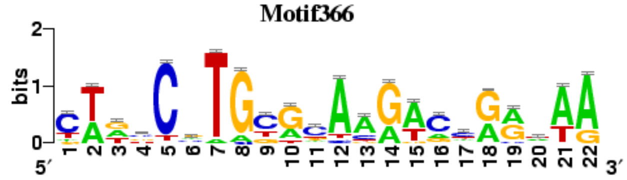	581.95	0.65	0.407	0.002
	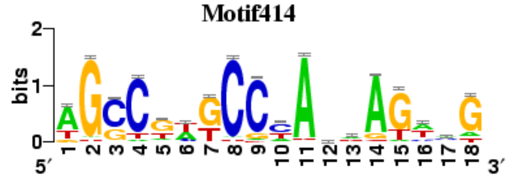	581.88	0.8	0.333	0.001
	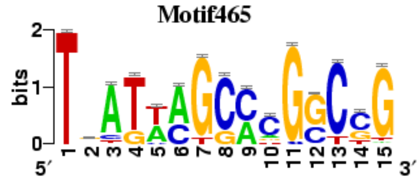	505.85	0.6	0.481	0.006
	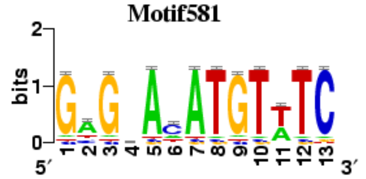	327.51	0.8	0.185	0.000
		81.76	0.95	0.074	0.000

39	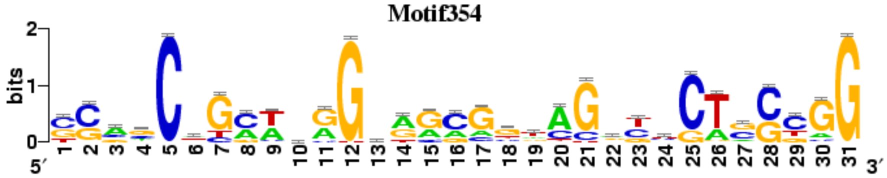				
	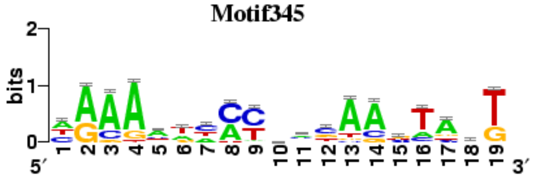	69.75	0.9	0.148	0.004
	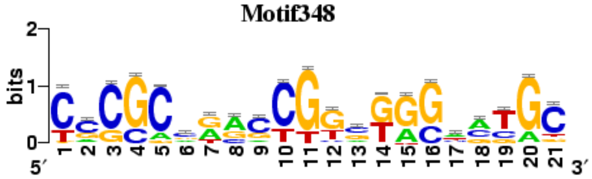	39.90	0.85	0.074	0.001
	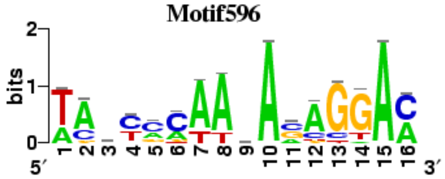	39.90	0.95	0.074	0.001
40	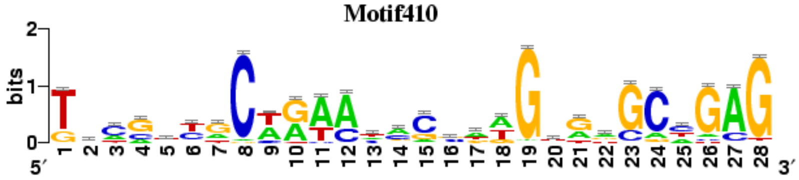	599.15	0.6	0.400	0.002
	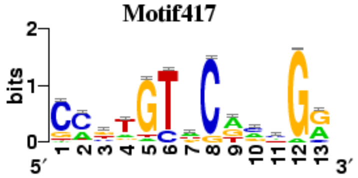	285.10	0.9	0.320	0.004
	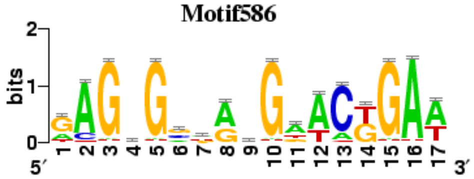	283.61	0.9	0.200	0.001
	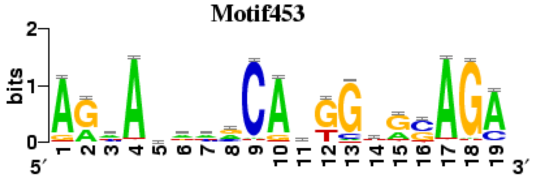	266.54	0.7	0.320	0.005
		88.96	0.95	0.080	0.000

	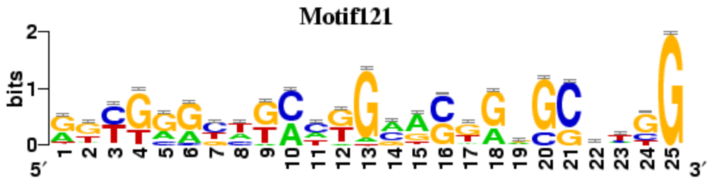				
41	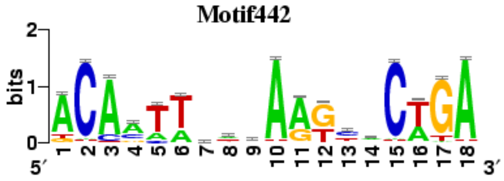	489.42	0.75	0.435	0.004
	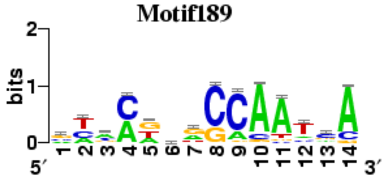	476.75	0.85	0.391	0.003
	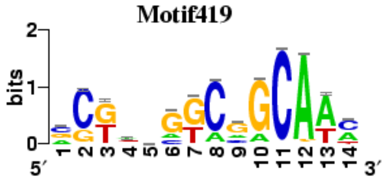	384.92	0.85	0.391	0.004
	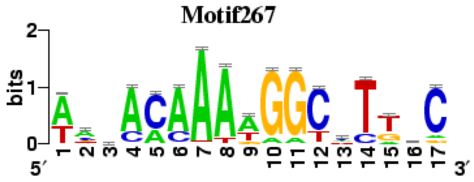	363.26	0.9	0.348	0.003
	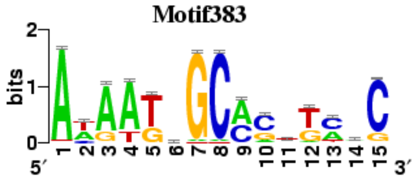	361.52	0.85	0.391	0.005
42	NA	NA	NA	NA	NA
43	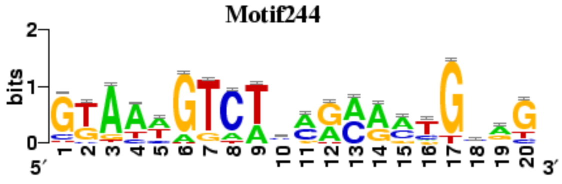	440.78	0.8	0.250	0.000
	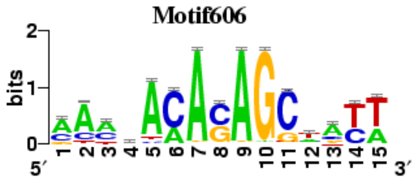	418.12	0.8	0.500	0.005
		394.31	0.8	0.500	0.006

	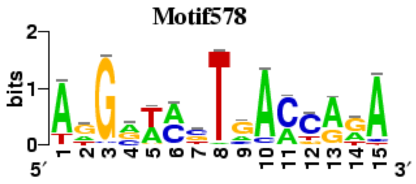				
	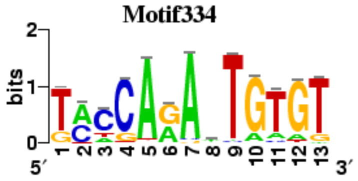	373.01	0.75	0.500	0.006
	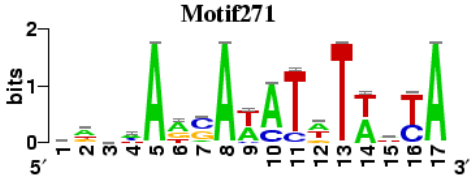	285.98	0.9	0.562	0.012
	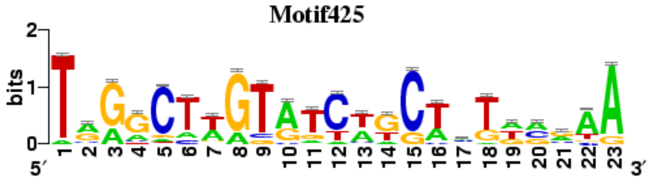	1661.57	0.65	0.875	0.001
	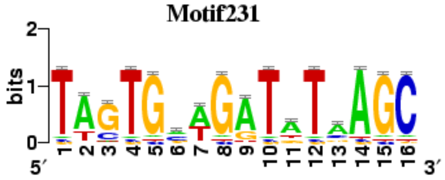	1556.84	0.75	0.875	0.002
**44**	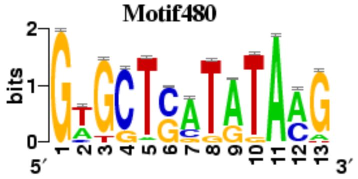	1556.84	0.7	0.875	0.002
	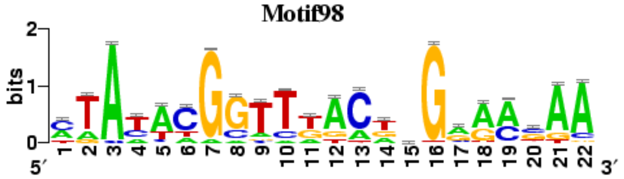	1486.16	0.7	0.750	0.001
	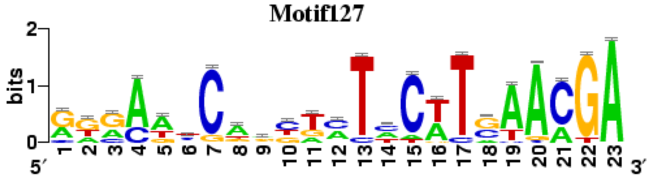	1486.16	0.6	0.750	0.001
		477.26	0.85	0.438	0.003

	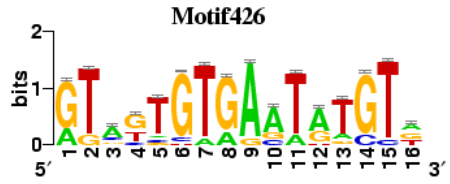				
	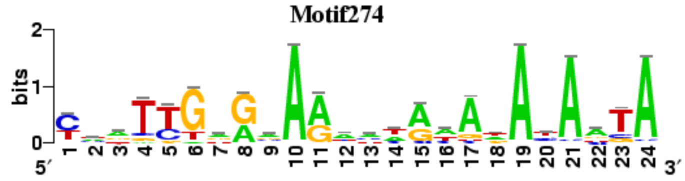	436.70	0.8	0.438	0.003
**45**	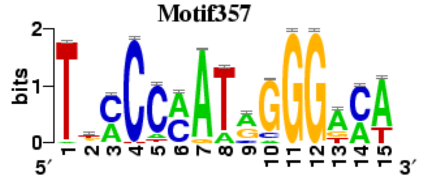	402.36	0.65	0.562	0.008
	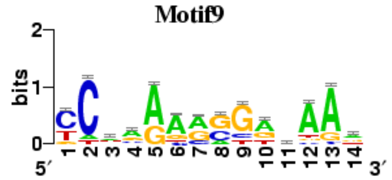	296.58	0.8	0.812	0.028
	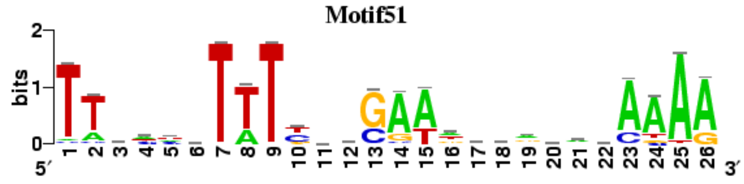	208.42	0.7	0.938	0.057
	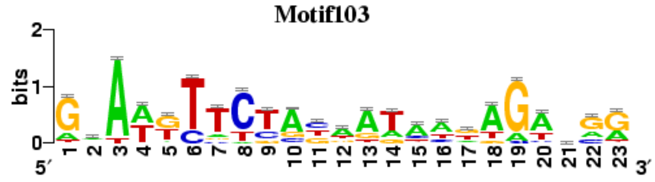	770.99	0.75	0.500	0.001
	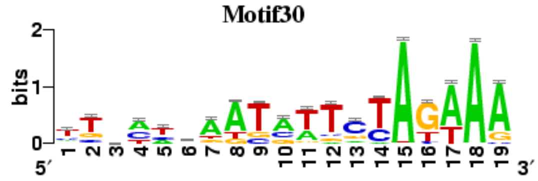	630.27	0.75	0.786	0.009
**46**	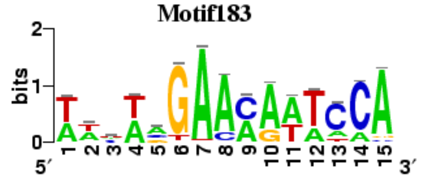	514.75	0.9	0.286	0.000
		471.62	0.9	0.643	0.008

	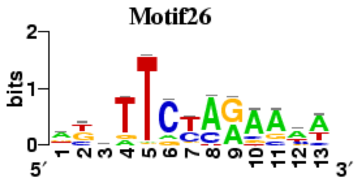				
	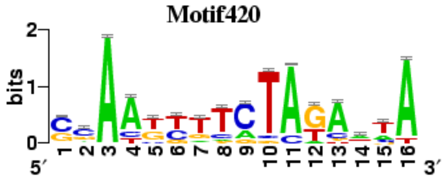	470.98	0.85	0.500	0.004
	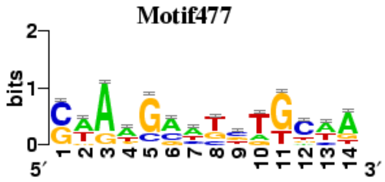	720.68	0.95	0.600	0.003
	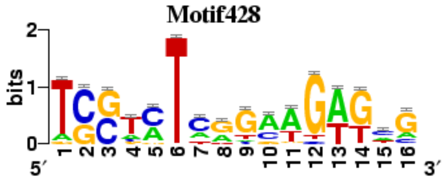	636.51	0.8	0.667	0.006
47	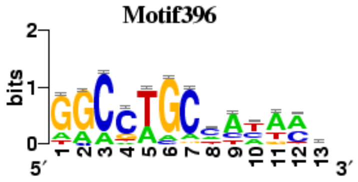	567.03	0.9	0.467	0.002
	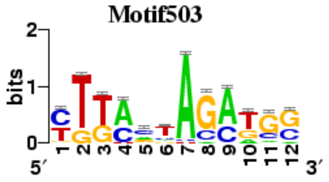	492.15	0.95	0.400	0.002
	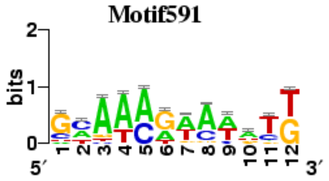	434.32	0.9	0.600	0.008
	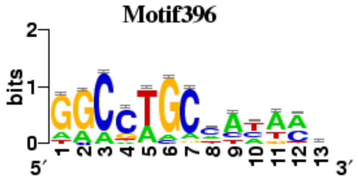	439.92	0.95	0.500	0.002
		202.90	0.85	0.500	0.006

48	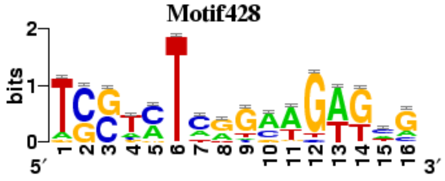				
	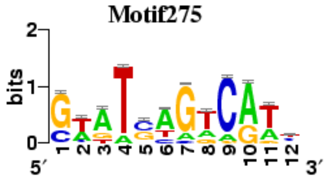	189.94	0.9	0.500	0.007
	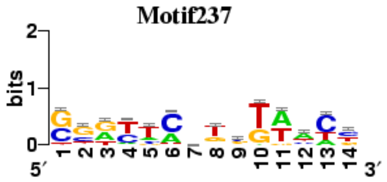	171.00	0.9	0.250	0.001
	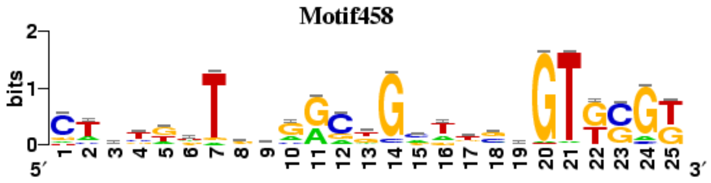	171.00	0.95	0.250	0.001
49	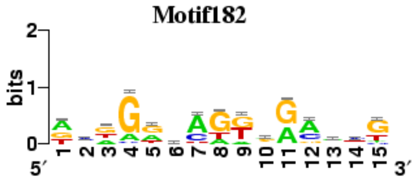	1030.49	0.95	0.636	0.001
	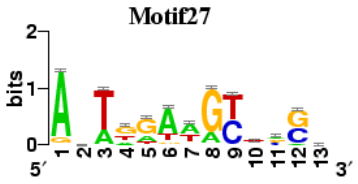	975.74	0.85	0.818	0.004
	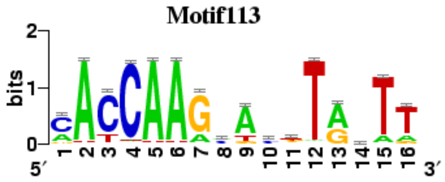	954.34	0.8	0.545	0.001
	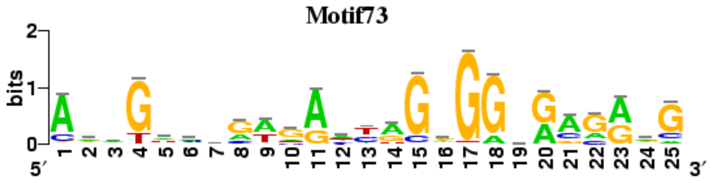	933.77	0.75	0.727	0.003
		713.95	0.85	0.545	0.002

	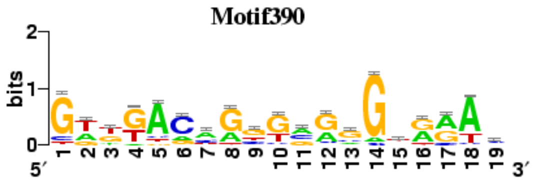				

CV02:

Cluster	Motif	Chi-square statistic	Score cutoff	Posterior probability within cluster	Posterior probability outside cluster
1	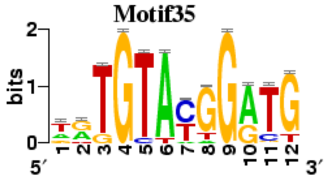	690.22	0.6	0.733	0.039
	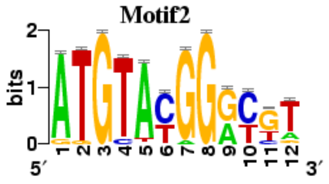	620.00	0.5	0.743	0.048
	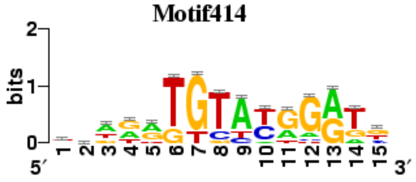	577.32	0.7	0.733	0.052
	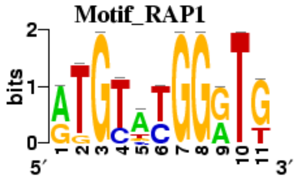	396.60	0.75	0.554	0.043
	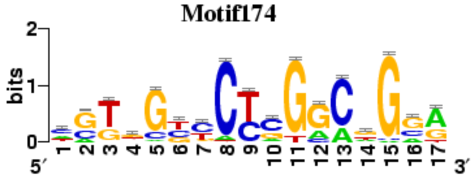	319.49	0.5	0.515	0.048
	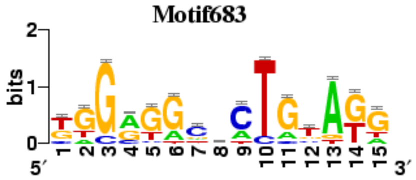	181.34	0.8	0.120	0.002
	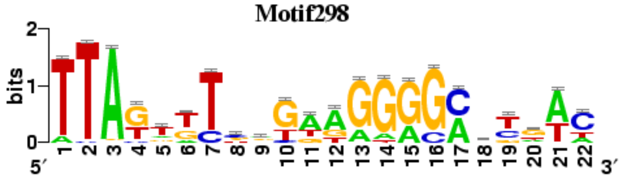	173.51	0.6	0.196	0.009

2	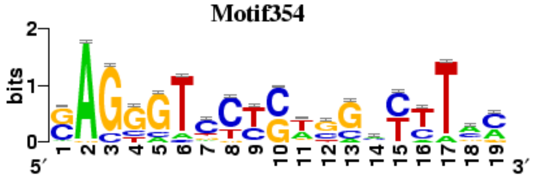	170.43	0.7	0.163	0.006
	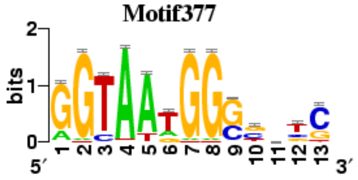	126.41	0.75	0.152	0.008
	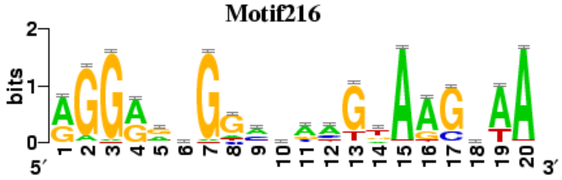	124.79	0.7	0.130	0.005
3	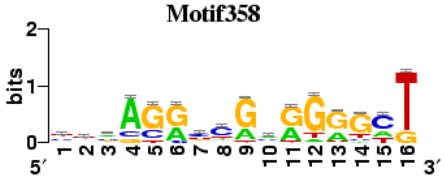	162.28	0.8	0.091	0.001
	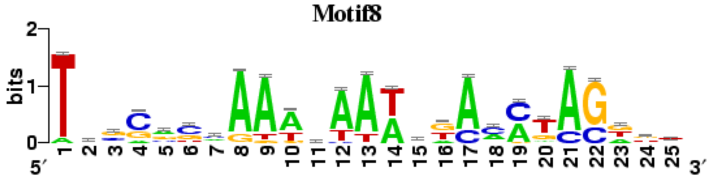	145.39	0.7	0.102	0.002
	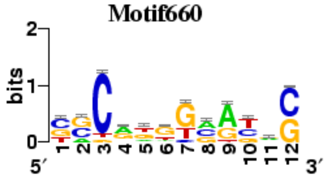	29.53	0.95	0.034	0.001
	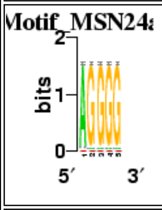	23.94	0.65	0.580	0.327
	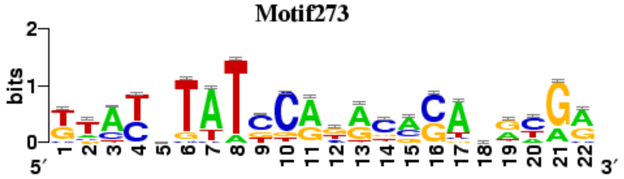	23.12	0.9	0.023	0.001
		394.47	0.6	0.686	0.063

4	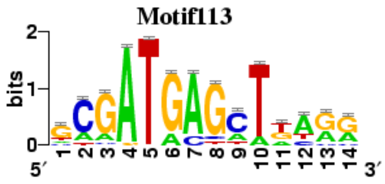				
	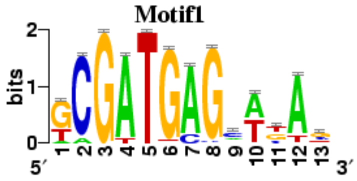	351.10	0.55	0.709	0.078
	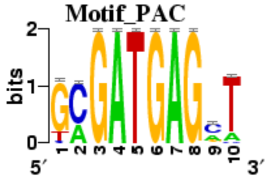	305.89	0.75	0.674	0.081
	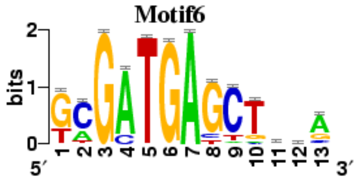	286.69	0.55	0.698	0.094
	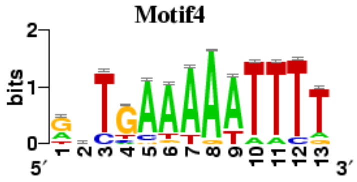	249.44	0.6	0.802	0.146
5	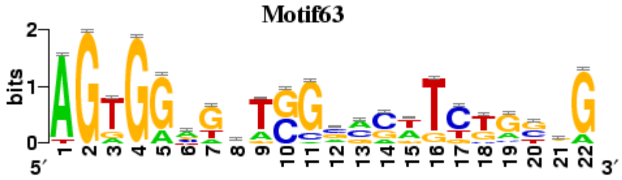	394.66	0.6	0.420	0.015
	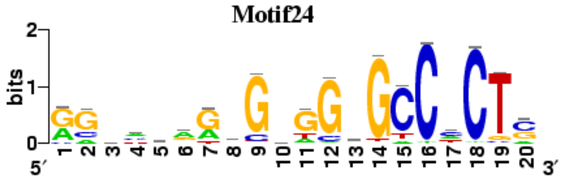	332.58	0.65	0.232	0.003
	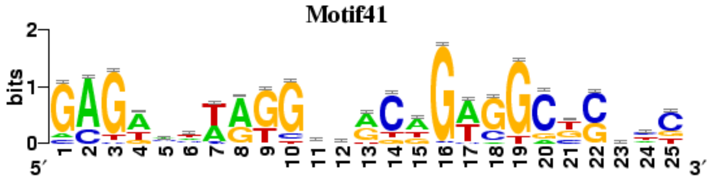	272.71	0.6	0.232	0.005
		263.76	0.6	0.246	0.006

	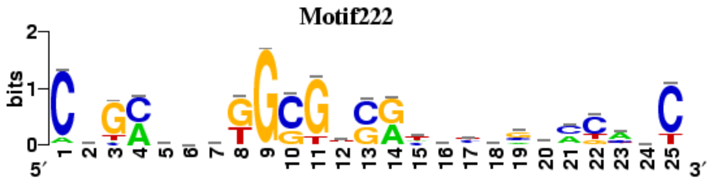				
	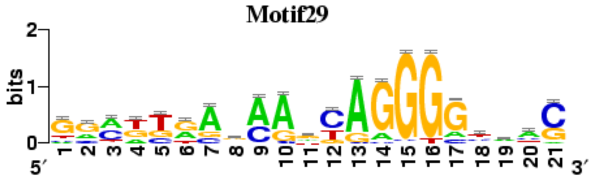	262.13	0.6	0.464	0.034
	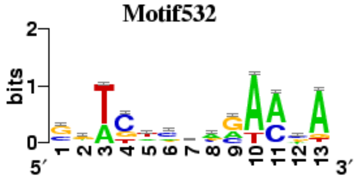	132.26	0.9	0.101	0.001
	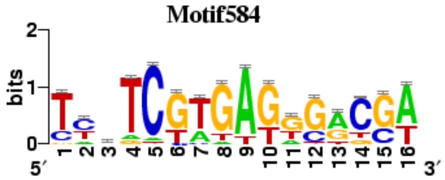	103.33	0.6	0.319	0.041
**6**	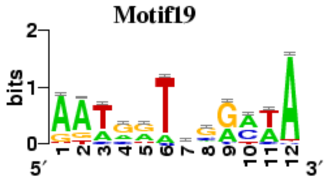	43.05	0.8	0.188	0.030
	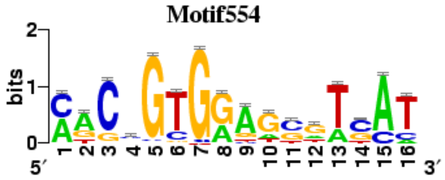	35.26	0.85	0.058	0.002
	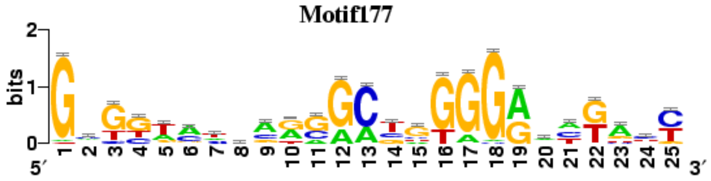	29.95	0.95	0.029	0.000
	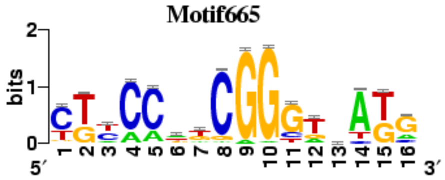	269.10	0.7	0.209	0.003
		173.54	0.8	0.149	0.003

7	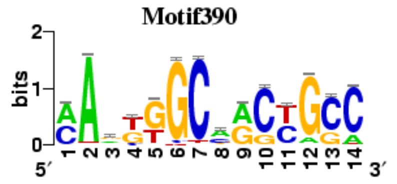				
	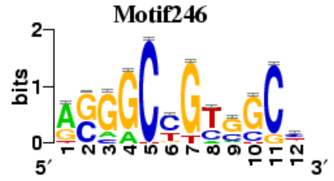	121.08	0.85	0.119	0.003
	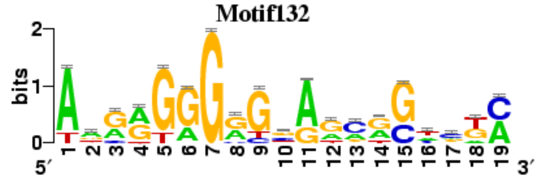	95.11	0.55	0.373	0.059
	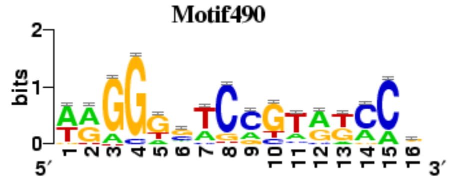	95.04	0.65	0.328	0.046
8	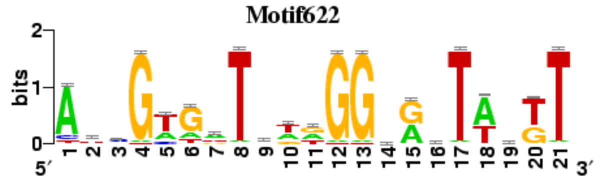	238.37	0.8	0.167	0.002
	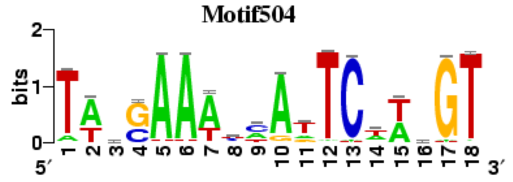	229.10	0.75	0.152	0.001
	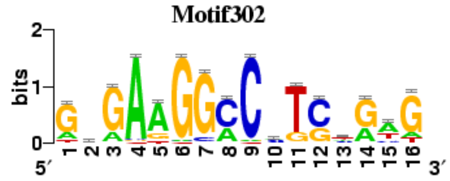	198.67	0.8	0.136	0.001
	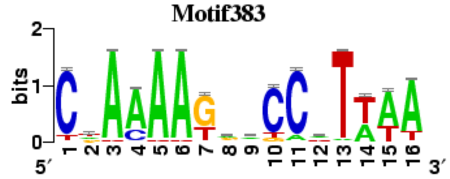	163.57	0.75	0.152	0.003
		146.23	0.7	0.379	0.040

	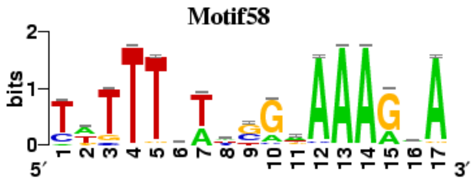				
9	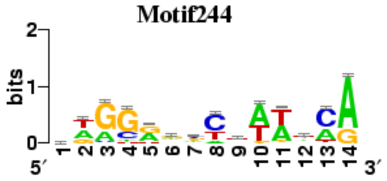	272.31	0.9	0.222	0.004
	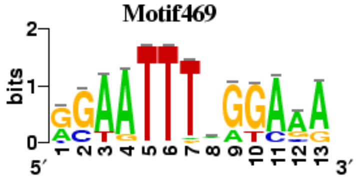	228.04	0.7	0.270	0.010
	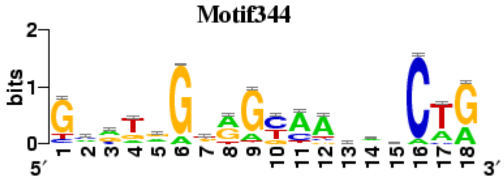	201.27	0.75	0.159	0.002
	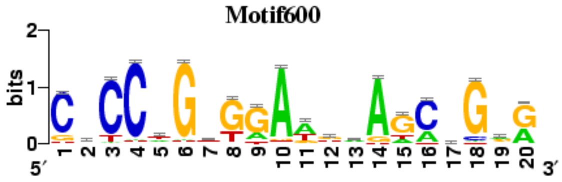	200.26	0.7	0.175	0.003
	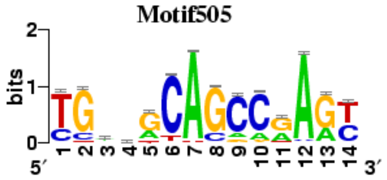	171.61	0.75	0.143	0.002
10	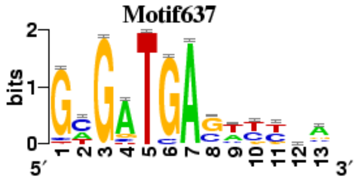	221.24	0.65	0.804	0.116
	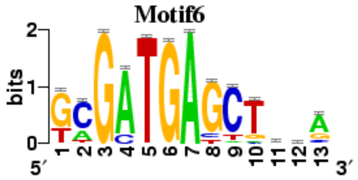	221.24	0.5	0.839	0.128
		213.53	0.7	0.196	0.004

	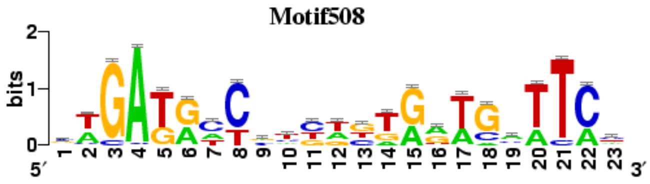				
	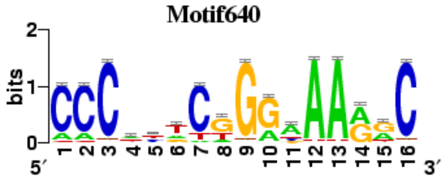	179.09	0.7	0.161	0.003
	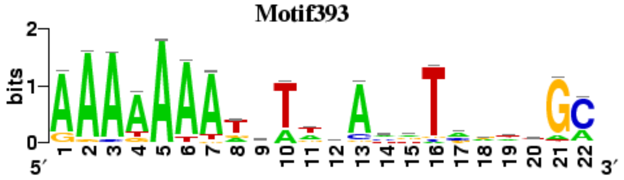	171.18	0.85	0.179	0.004
	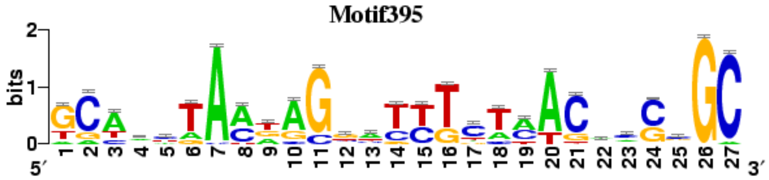	379.58	0.6	0.344	0.008
	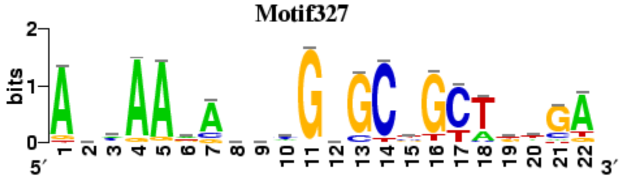	357.67	0.75	0.279	0.004
**11**	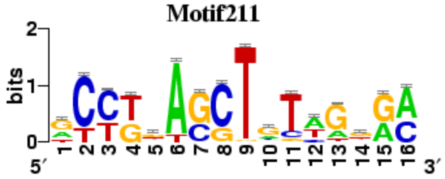	344.36	0.8	0.197	0.001
	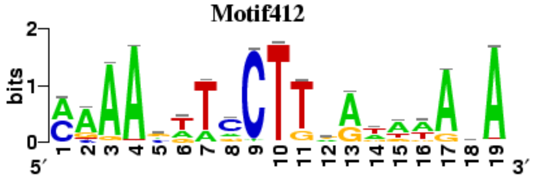	238.57	0.75	0.213	0.004
	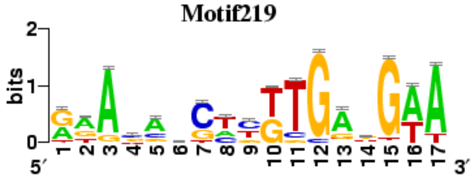	207.12	0.75	0.377	0.025
		320.62	0.75	0.214	0.002

12	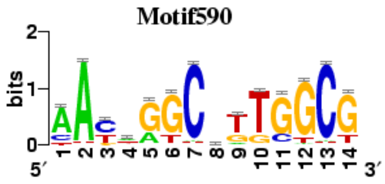				
	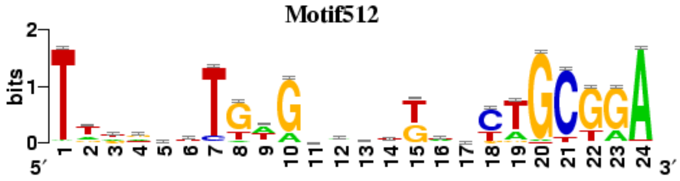	249.34	0.75	0.179	0.002
	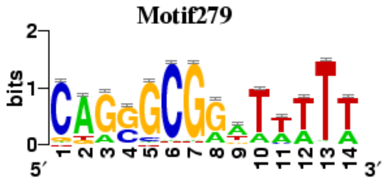	207.18	0.7	0.214	0.005
	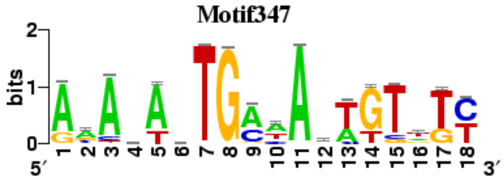	197.80	0.75	0.393	0.027
	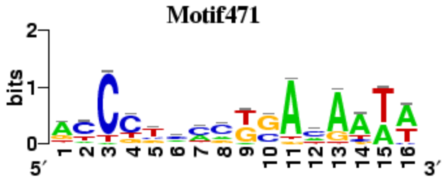	160.77	0.8	0.179	0.005
13	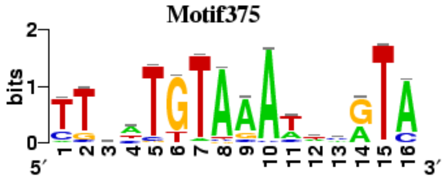	195.04	0.8	0.246	0.009
	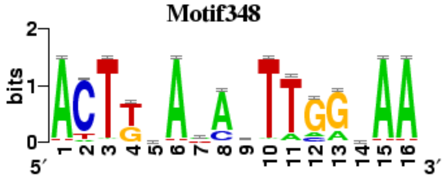	159.24	0.8	0.140	0.002
	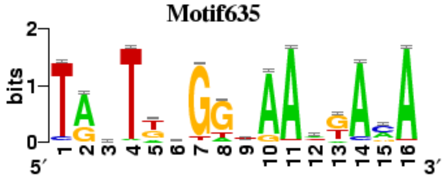	137.93	0.75	0.228	0.012
		58.47	0.9	0.123	0.007

	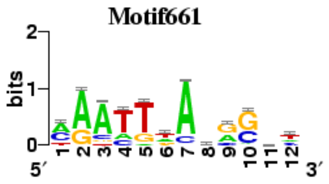				
	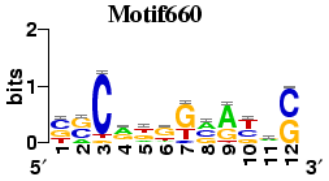	26.68	0.5	0.246	0.585
	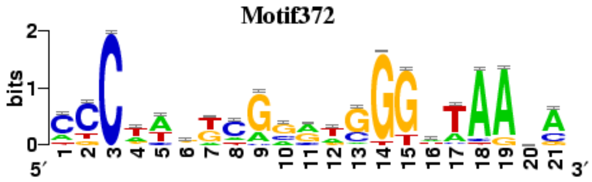	390.18	0.75	0.404	0.011
	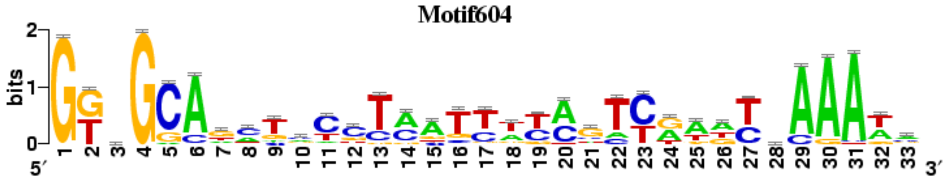	320.18	0.5	0.263	0.004
**14**	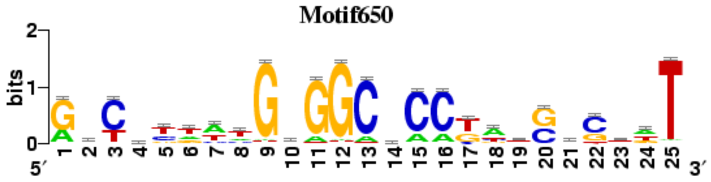	279.39	0.65	0.193	0.002
	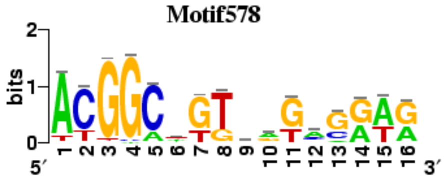	232.76	0.85	0.158	0.001
	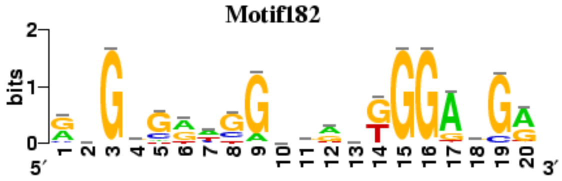	210.75	0.75	0.228	0.006
	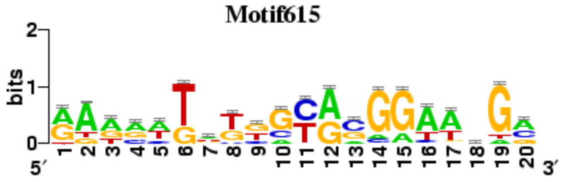	360.52	0.85	0.208	0.001
		331.53	0.8	0.245	0.003

15	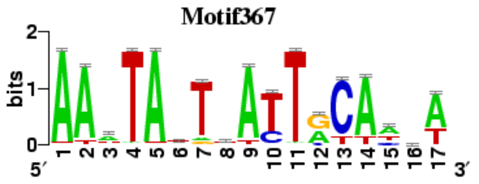				
	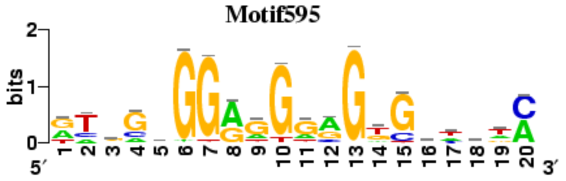	312.40	0.8	0.264	0.004
	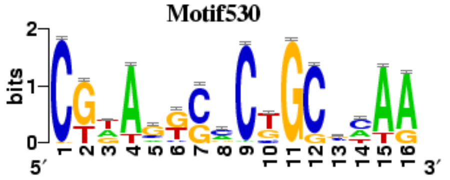	297.46	0.6	0.302	0.007
	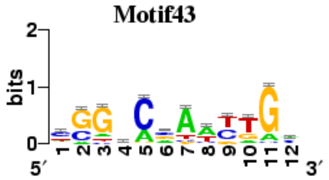	264.36	0.9	0.245	0.005
16	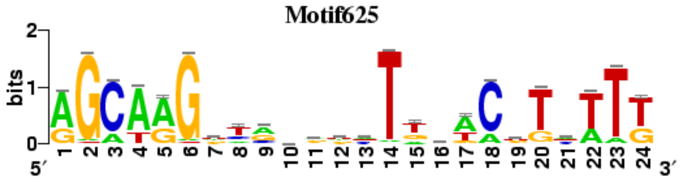	248.72	0.65	0.232	0.005
	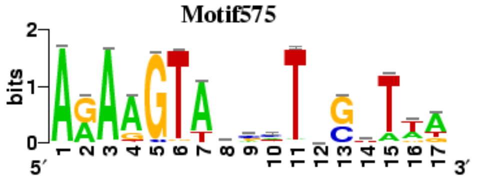	228.89	0.8	0.179	0.002
	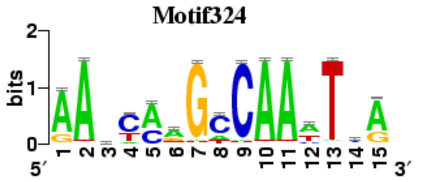	228.14	0.85	0.143	0.001
	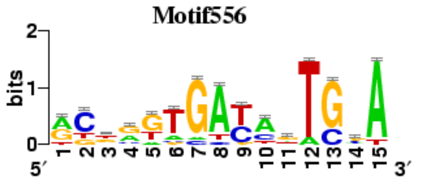	223.65	0.8	0.250	0.007
		214.33	0.75	0.161	0.002

	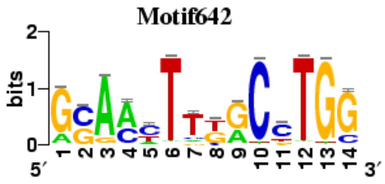				
17	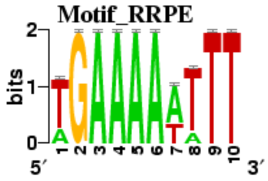	58.01	0.8	0.574	0.168
	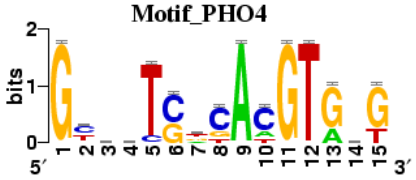	50.57	0.95	0.056	0.001
	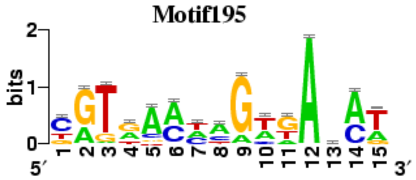	46.90	0.9	0.093	0.004
	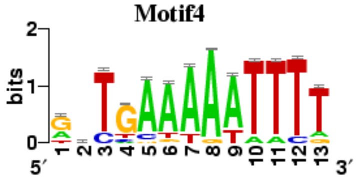	40.20	0.6	0.500	0.164
	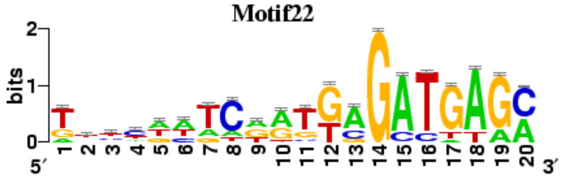	38.88	0.95	0.037	0.000
18	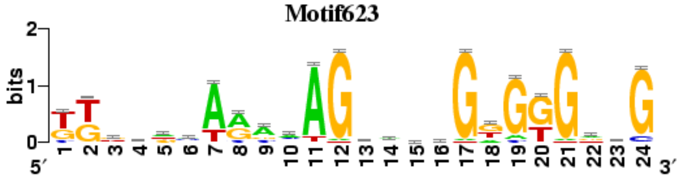	347.59	0.7	0.271	0.003
	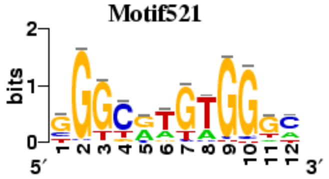	139.55	0.85	0.146	0.003
		99.40	0.8	0.146	0.005

	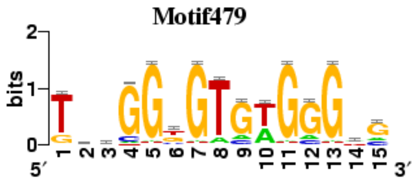				
	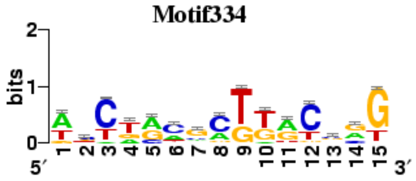	97.65	0.95	0.104	0.002
	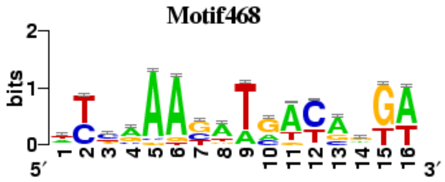	57.51	0.9	0.062	0.001
**19**	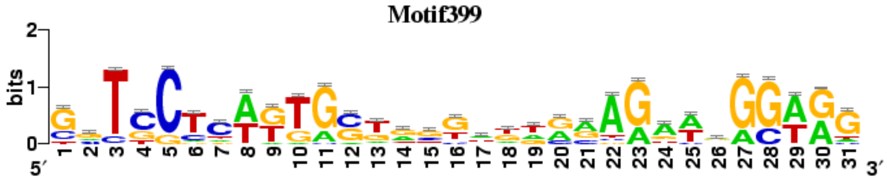	400.35	0.65	0.245	0.001
	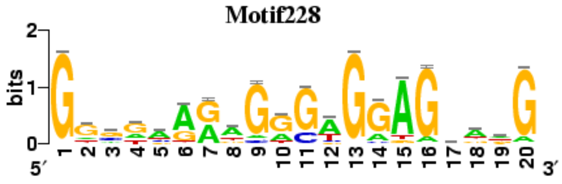	227.27	0.7	0.204	0.003
	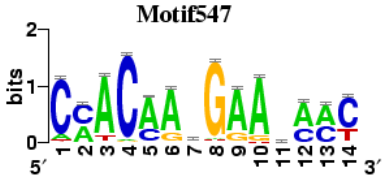	187.17	0.8	0.224	0.006
	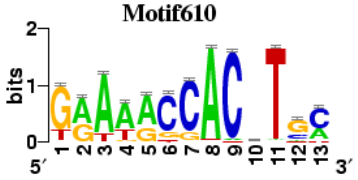	178.26	0.8	0.224	0.007
	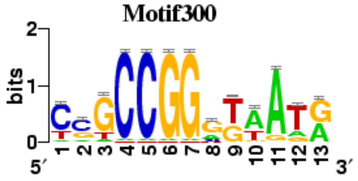	170.10	0.8	0.224	0.007
		402.73	0.85	0.277	0.002

	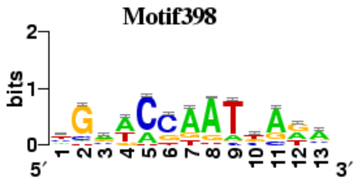				
	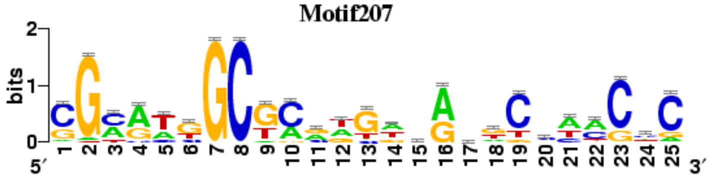	318.37	0.75	0.234	0.002
20	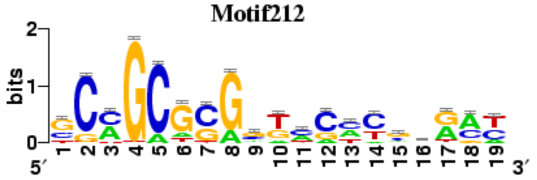	281.48	0.7	0.383	0.014
	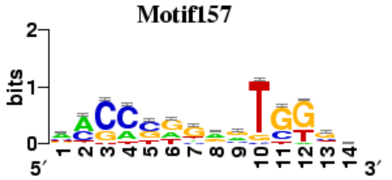	243.32	0.85	0.170	0.001
	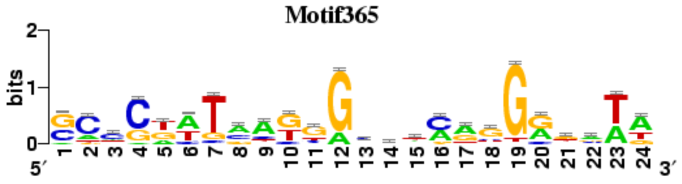	196.74	0.85	0.170	0.002
	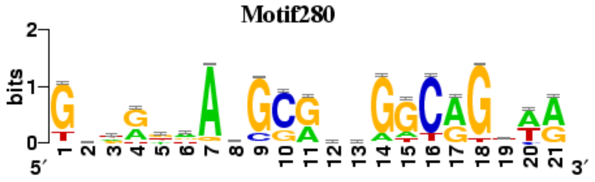	370.34	0.8	0.196	0.000
21	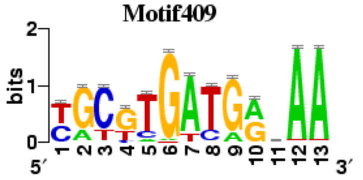	211.38	0.75	0.239	0.006
	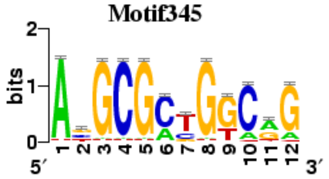	205.31	0.8	0.152	0.001
		174.76	0.75	0.239	0.008

	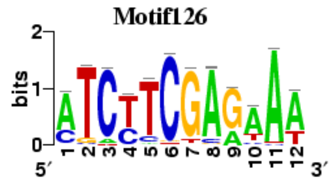				
	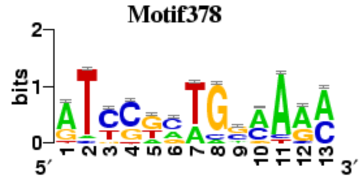	112.47	0.8	0.239	0.014
	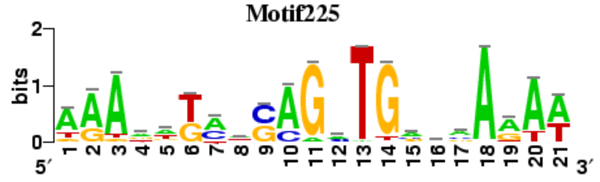	175.11	0.8	0.205	0.005
	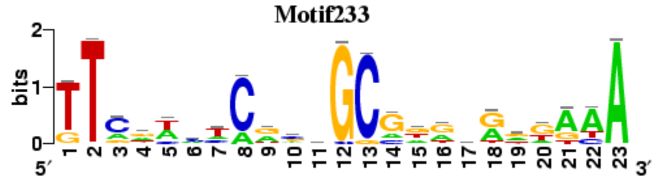	48.38	0.95	0.045	0.000
22	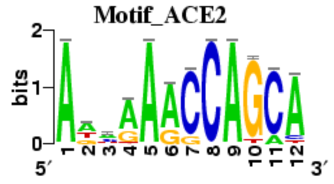	15.48	0.7	0.136	0.024
	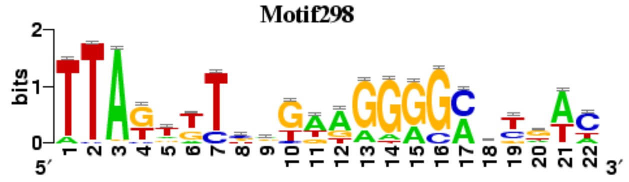	14.83	0.9	0.045	0.001
	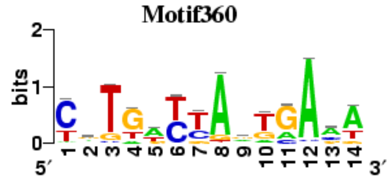	14.83	0.95	0.045	0.001
	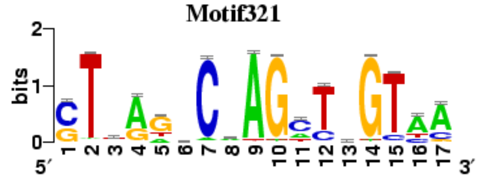	290.46	0.75	0.222	0.002
		106.99	0.9	0.156	0.005

23	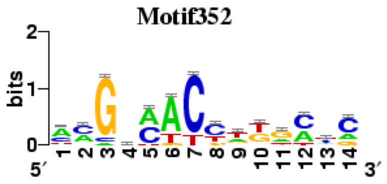				
	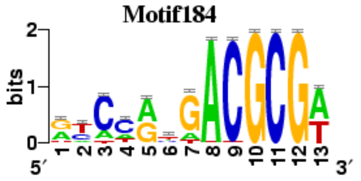	61.71	0.95	0.067	0.001
	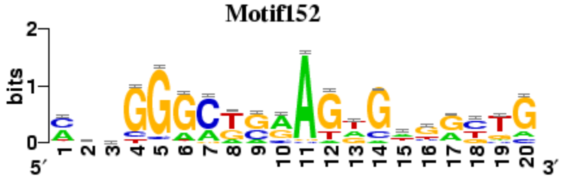	47.23	0.85	0.044	0.000
	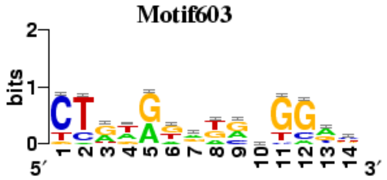	47.23	0.95	0.044	0.000
24	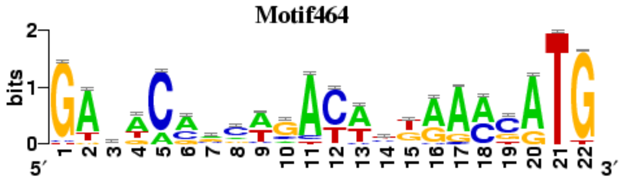	426.53	0.6	0.279	0.002
	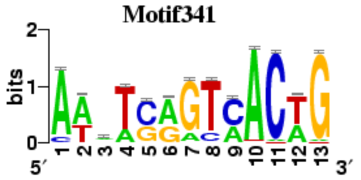	374.13	0.8	0.326	0.005
	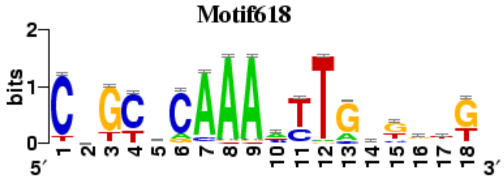	370.82	0.65	0.279	0.003
	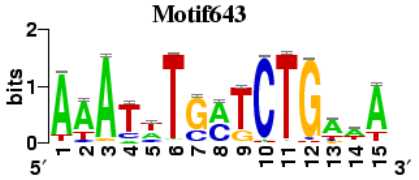	347.89	0.8	0.279	0.003
		333.01	0.6	0.302	0.005

	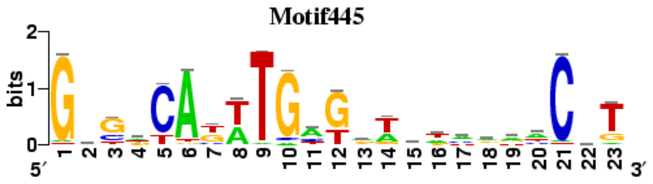				
	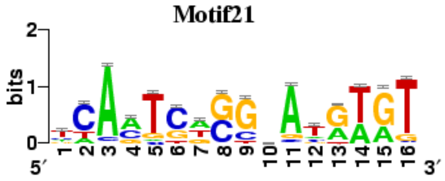	155.70	0.9	0.143	0.002
	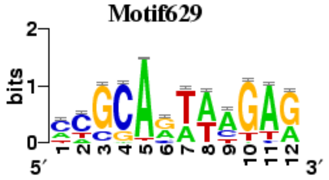	101.75	0.95	0.071	0.000
25	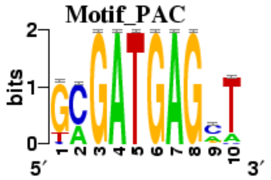	82.15	0.6	0.619	0.128
	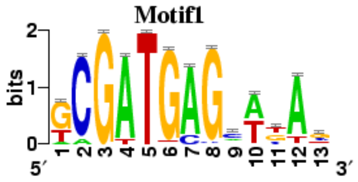	67.03	0.5	0.548	0.117
	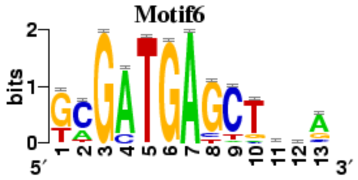	57.05	0.55	0.500	0.111
	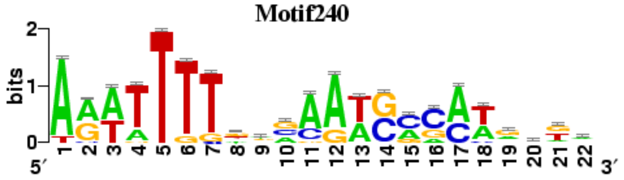	223.46	0.55	0.636	0.057
26	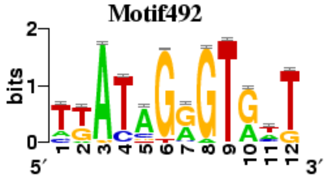	213.35	0.75	0.295	0.010
		135.78	0.85	0.318	0.021

	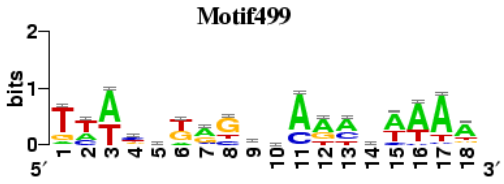				
	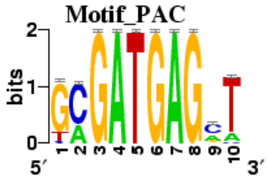	89.18	0.75	0.545	0.096
	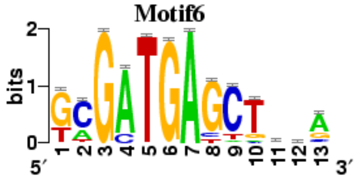	84.77	0.7	0.386	0.050
	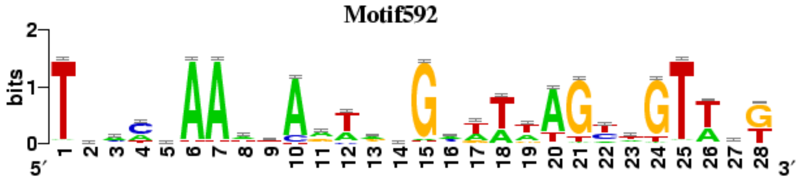	391.78	0.75	0.227	0.001
	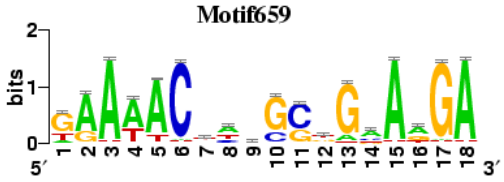	343.59	0.8	0.205	0.001
**27**	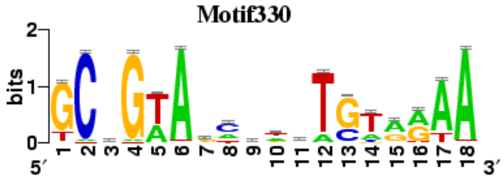	297.66	0.8	0.227	0.002
	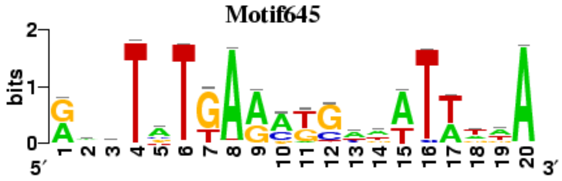	297.66	0.8	0.227	0.002
	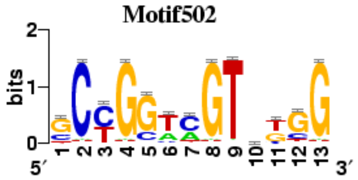	254.07	0.75	0.205	0.002
		643.00	0.85	0.659	0.015

	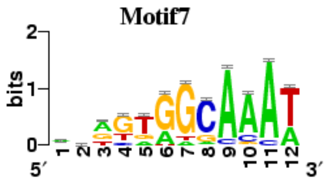				
	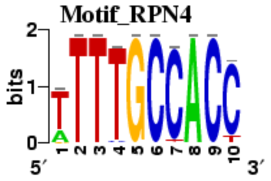	448.87	0.8	0.568	0.018
**28**	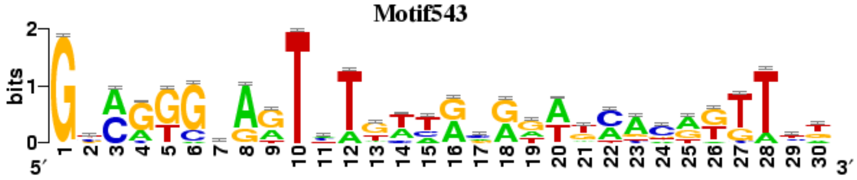	356.20	0.6	0.341	0.006
	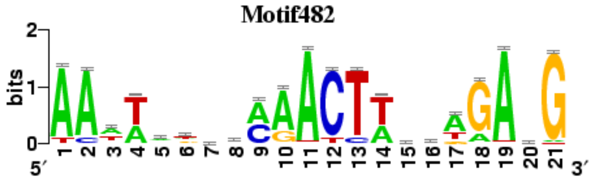	297.66	0.75	0.227	0.002
	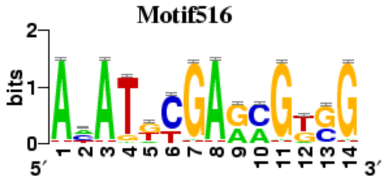	233.90	0.8	0.182	0.002
	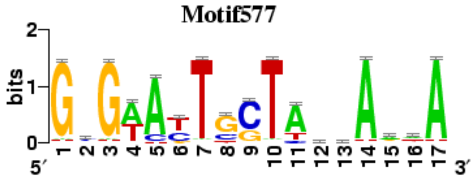	252.59	0.75	0.195	0.002
	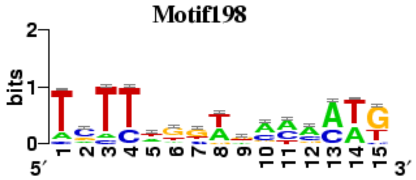	228.47	0.9	0.195	0.002
**29**	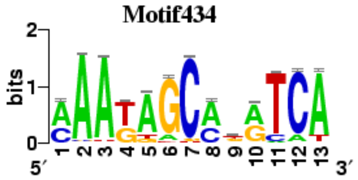	203.40	0.8	0.244	0.006
		199.19	0.75	0.268	0.008

	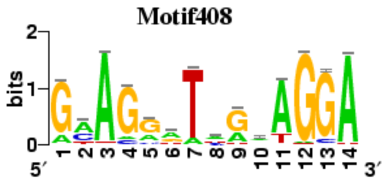				
	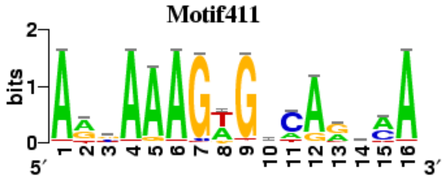	116.07	0.95	0.098	0.001
**30**	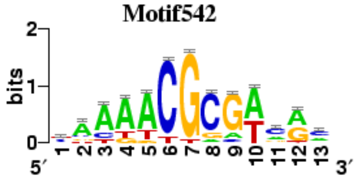	951.39	0.75	0.667	0.006
	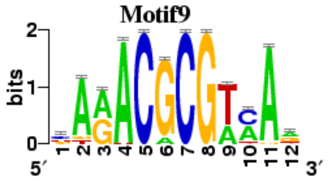	577.78	0.55	0.905	0.040
	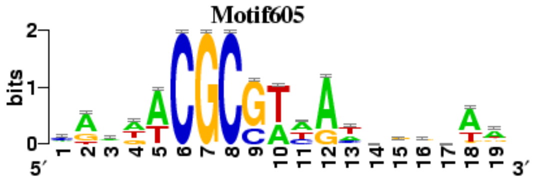	487.52	0.6	0.905	0.050
	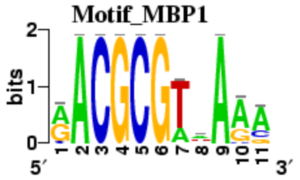	416.42	0.7	0.548	0.018
	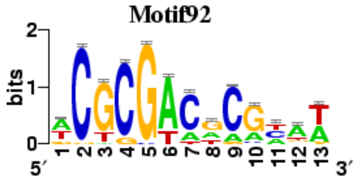	349.24	0.6	0.619	0.030
	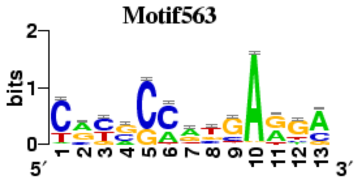	289.72	0.8	0.200	0.001
		241.83	0.7	0.400	0.016

31	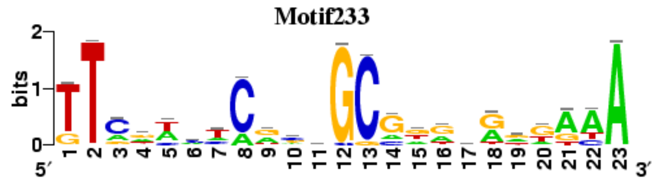				
	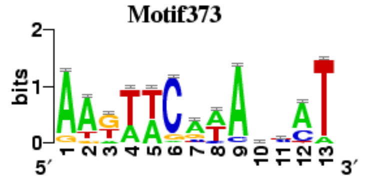	181.73	0.9	0.200	0.004
	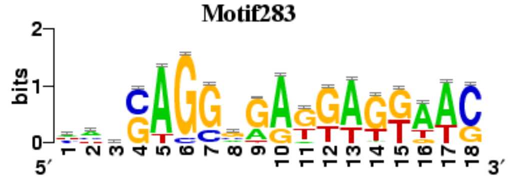	151.05	0.7	0.275	0.012
	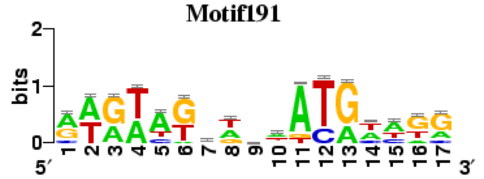	53.58	0.95	0.050	0.000
32	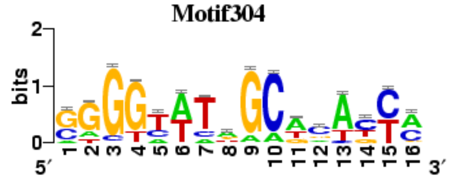	486.31	0.8	0.278	0.001
	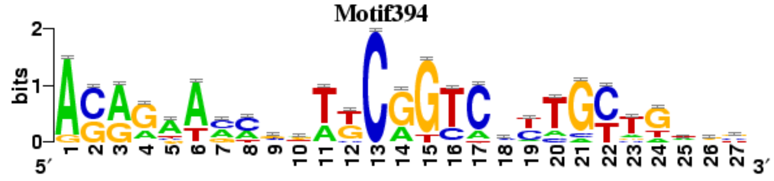	477.60	0.55	0.389	0.004
	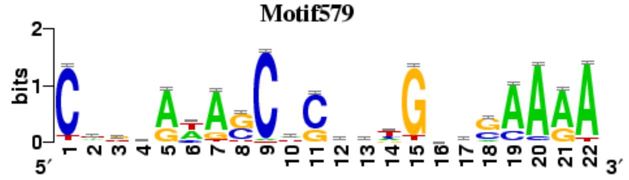	338.58	0.7	0.333	0.005
	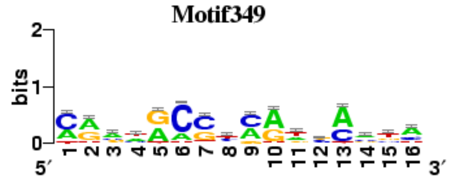	324.81	0.9	0.222	0.001
		291.08	0.8	0.222	0.002

	Motif418 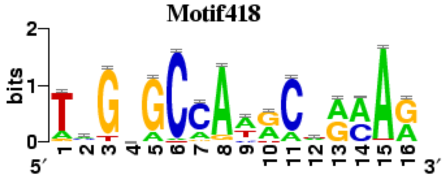				
	Motif315 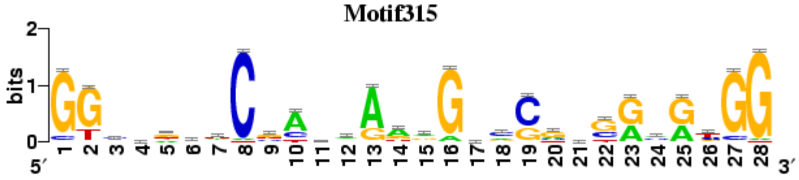	517.33	0.65	0.294	0.001
	Motif108 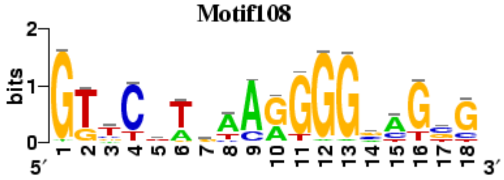	428.52	0.7	0.294	0.002
33	Motif436 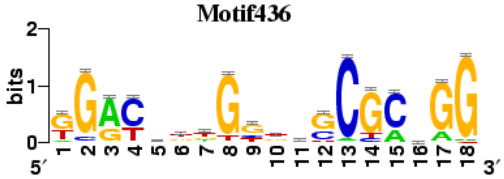	360.75	0.7	0.353	0.005
	Motif481 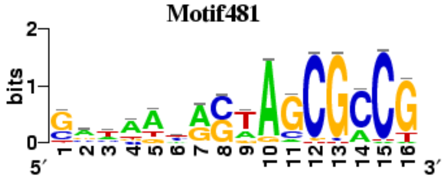	339.71	0.85	0.294	0.003
	Motif223 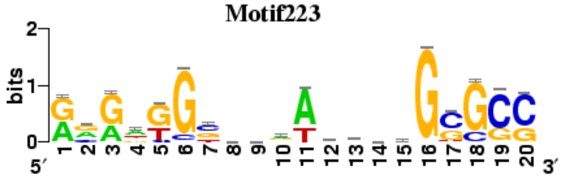	337.83	0.75	0.412	0.009
	Motif586 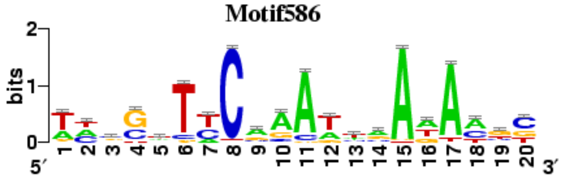	517.33	0.9	0.294	0.001
34	Motif535 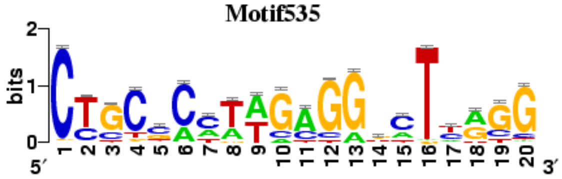	468.88	0.9	0.294	0.001
		447.98	0.95	0.235	0.000

	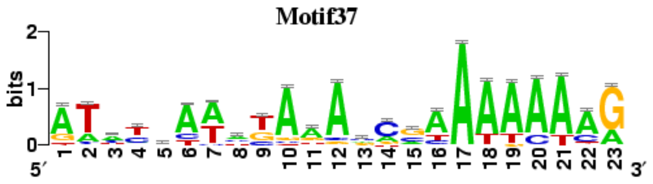				
	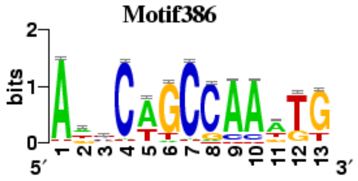	390.42	0.9	0.235	0.001
	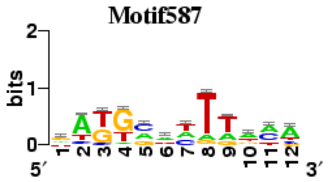	390.42	0.95	0.235	0.001
35	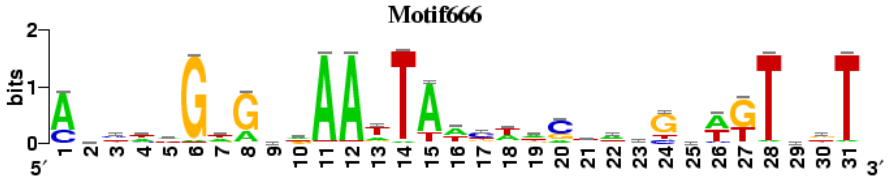	484.35	0.75	0.303	0.001
	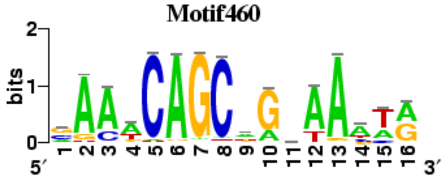	468.67	0.8	0.273	0.001
	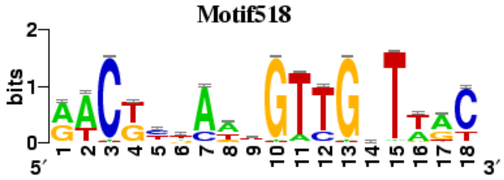	407.37	0.7	0.333	0.003
	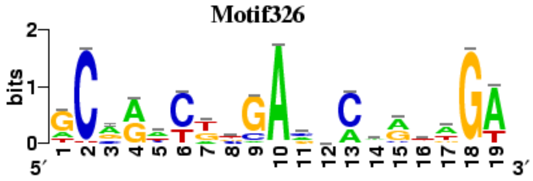	355.14	0.75	0.364	0.006
	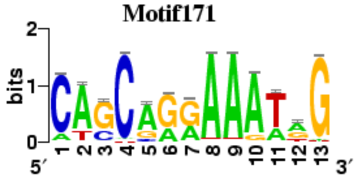	294.56	0.8	0.212	0.001
		372.73	0.8	0.344	0.004

	Motif359 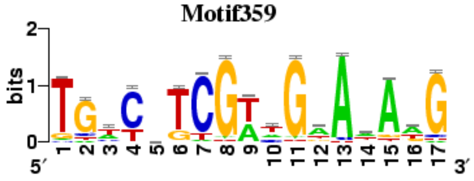				
	Motif606 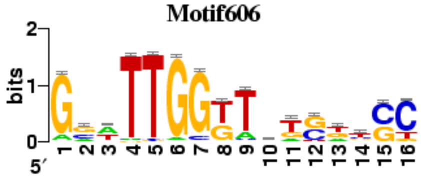	306.38	0.65	0.406	0.010
**36**	Motif555 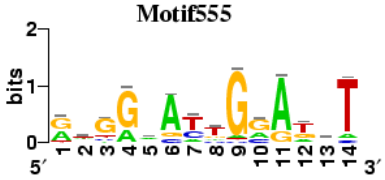	152.76	0.9	0.156	0.002
	Motif148 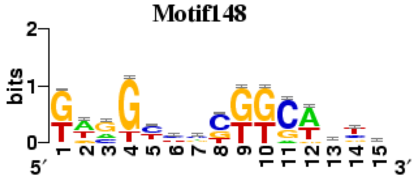	68.13	0.95	0.062	0.000
	Motif469 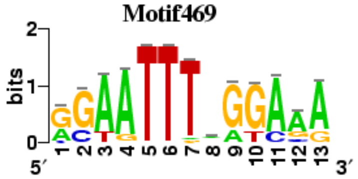	68.13	0.95	0.062	0.000
	Motif309 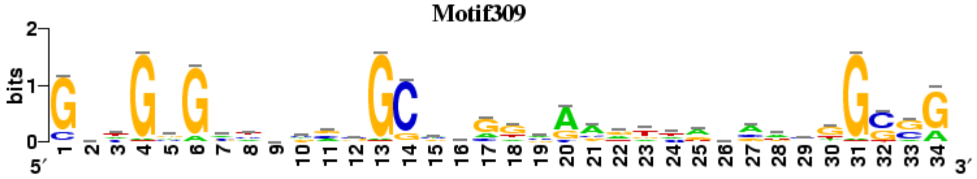	607.97	0.6	0.346	0.001
**37**	Motif538 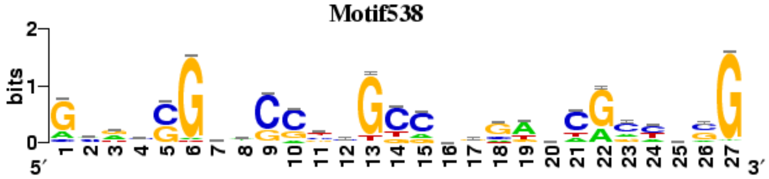	427.91	0.9	0.231	0.000
	Motif142 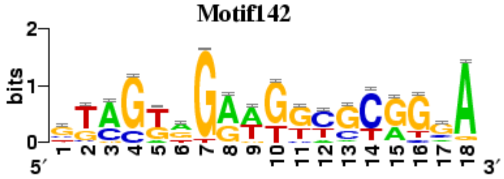	416.16	0.7	0.308	0.002
		342.16	0.75	0.192	0.000

	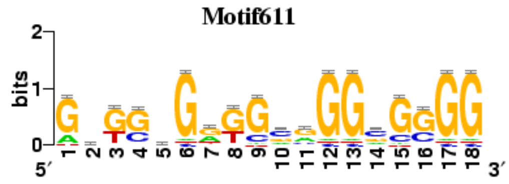				
	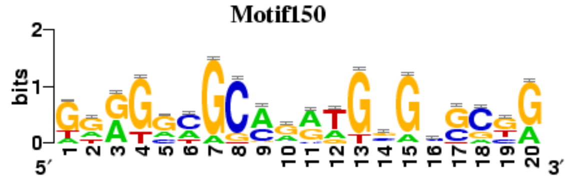	280.97	0.85	0.346	0.006
38	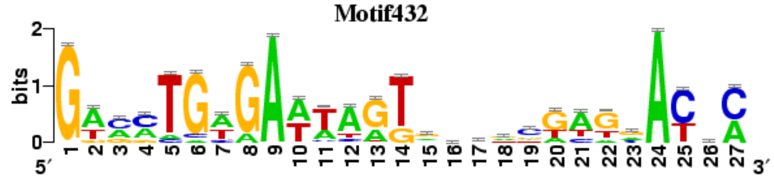	683.29	0.8	0.407	0.001
	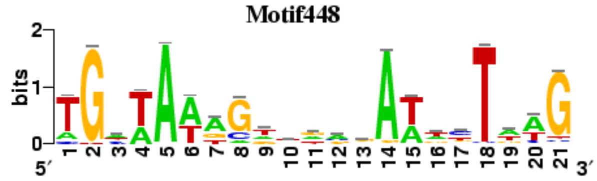	659.74	0.75	0.444	0.002
	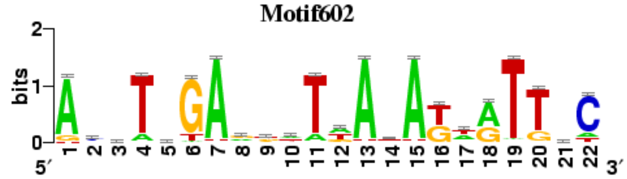	583.38	0.75	0.407	0.002
	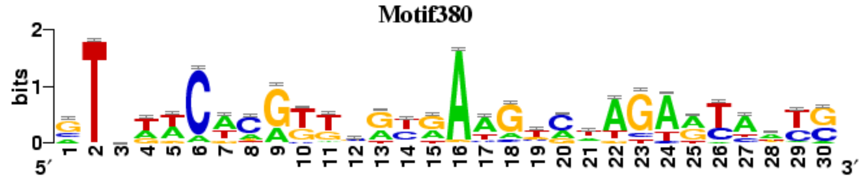	546.93	0.55	0.556	0.008
	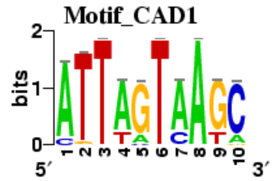	523.62	0.9	0.333	0.001
	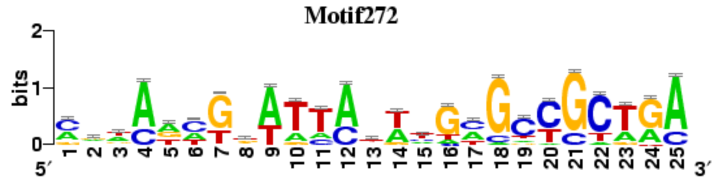	78.77	0.95	0.071	0.000
		38.41	0.95	0.071	0.001

39	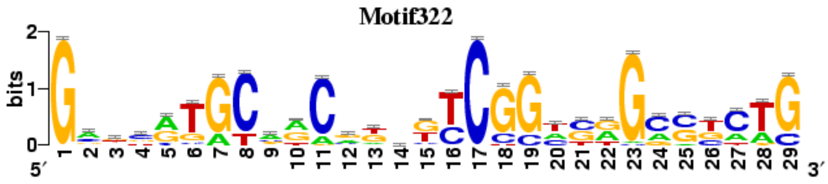				
	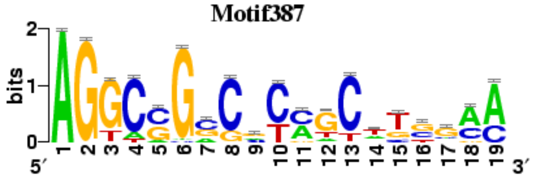	38.41	0.9	0.071	0.001
	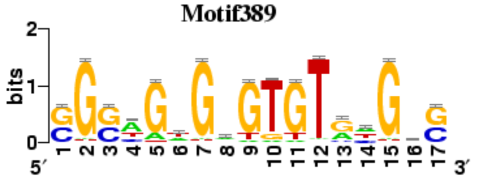	38.41	0.95	0.071	0.001
	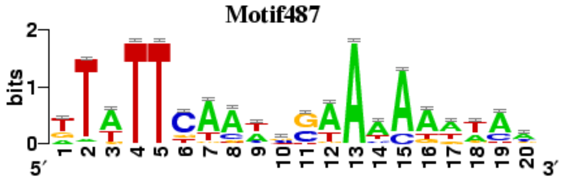	38.41	0.95	0.071	0.001
40	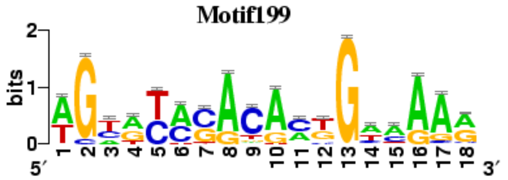	472.78	0.75	0.360	0.002
	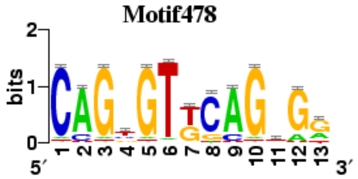	370.79	0.8	0.240	0.001
	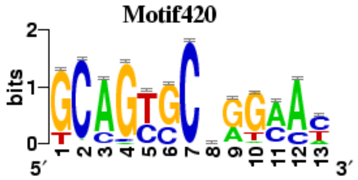	293.73	0.75	0.360	0.006
	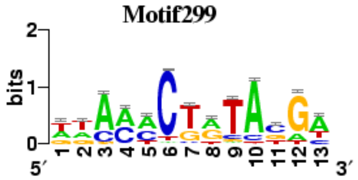	284.31	0.95	0.200	0.001
		267.78	0.85	0.160	0.000

	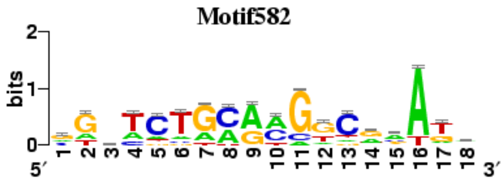				
41	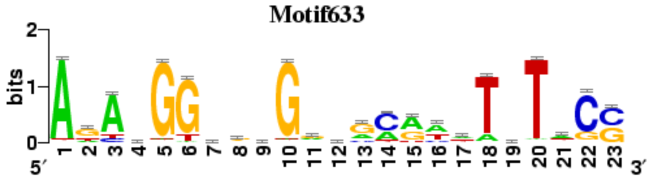	625.19	0.75	0.391	0.001
	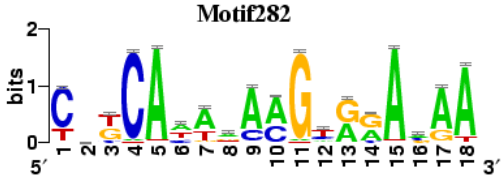	587.99	0.9	0.304	0.000
	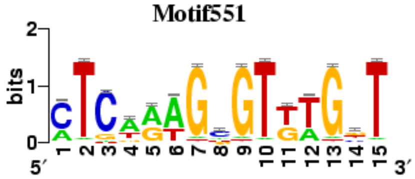	587.99	0.75	0.304	0.000
	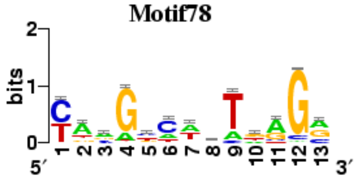	476.91	0.85	0.348	0.002
	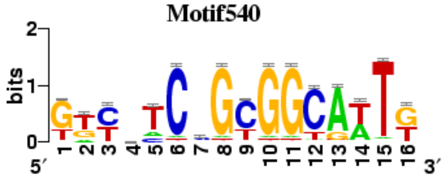	438.41	0.75	0.304	0.001
42	NA	NA	NA	NA	NA
43	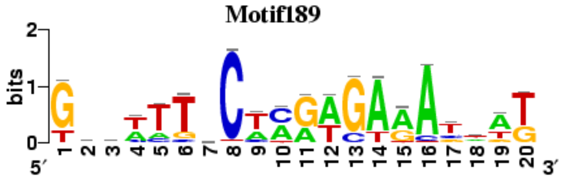	739.05	0.75	0.588	0.003
	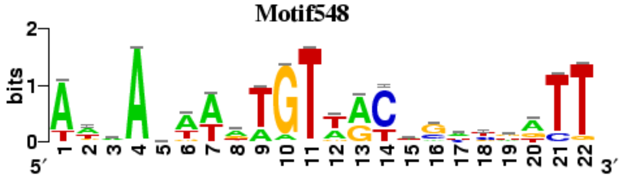	609.32	0.8	0.471	0.002
		571.66	0.95	0.353	0.001

	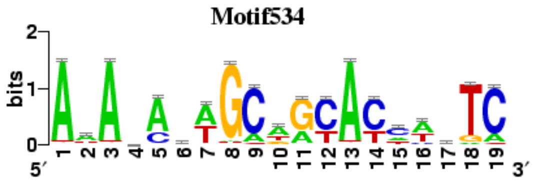				
	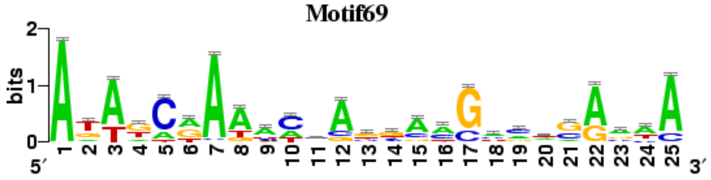	364.26	0.85	0.294	0.001
	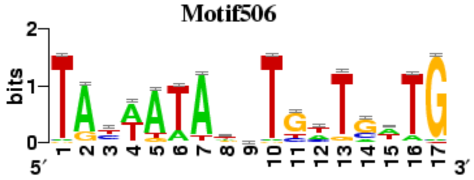	324.47	0.85	0.412	0.005
44	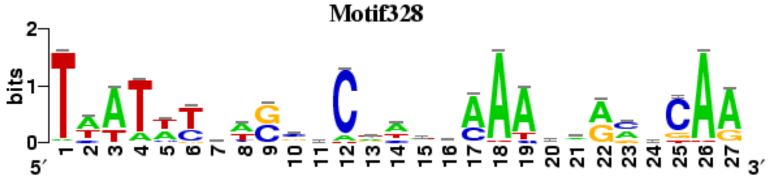	1933.86	0.7	0.882	0.000
	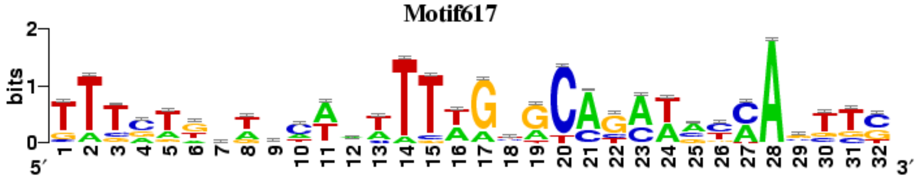	1803.93	0.6	0.882	0.001
	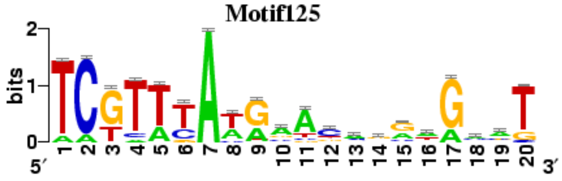	1690.25	0.65	0.882	0.001
	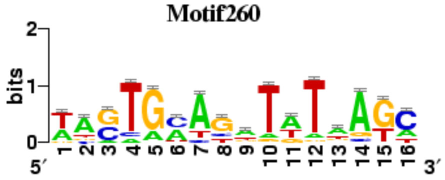	1665.59	0.85	0.824	0.001
	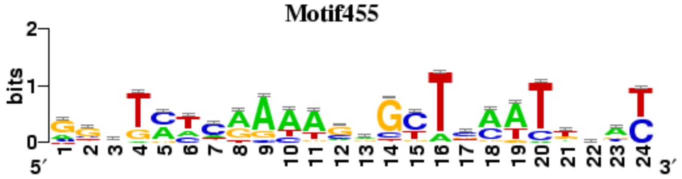	1655.99	0.85	0.765	0.000
		940.07	0.5	0.765	0.005

45	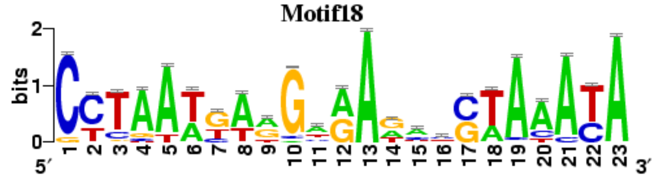				
	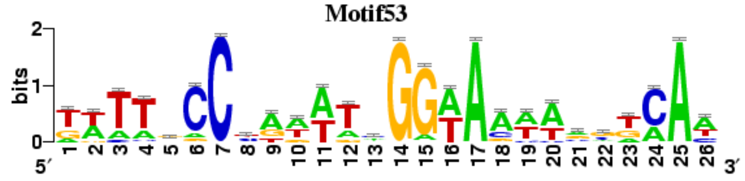	747.11	0.7	0.471	0.001
	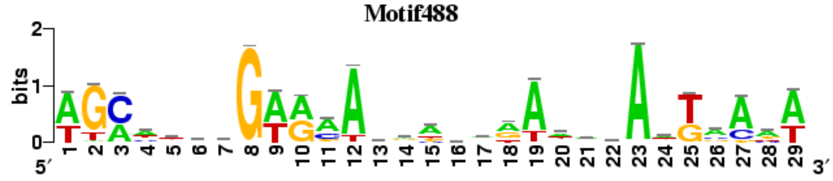	587.55	0.8	0.706	0.009
	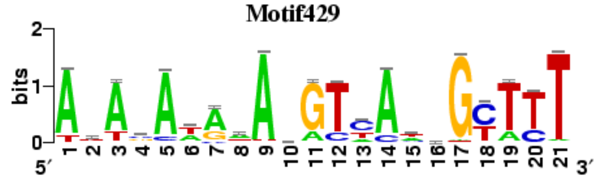	512.28	0.85	0.529	0.005
	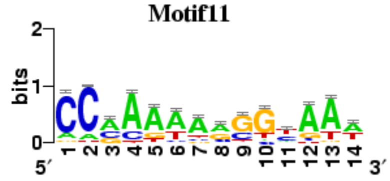	499.87	0.85	0.588	0.007
46	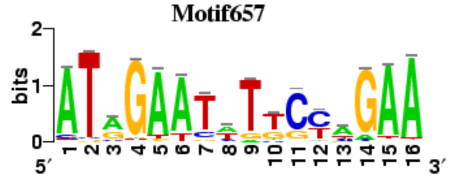	716.03	0.75	0.429	0.001
	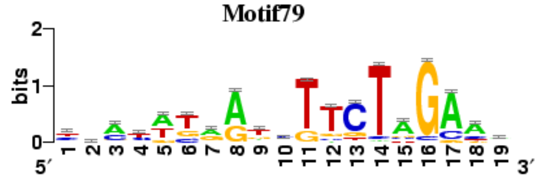	643.42	0.8	0.643	0.005
	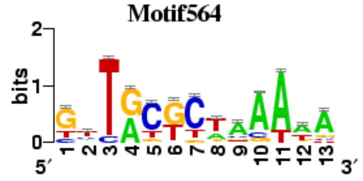	474.71	0.85	0.429	0.002
		189.53	0.8	0.429	0.009

	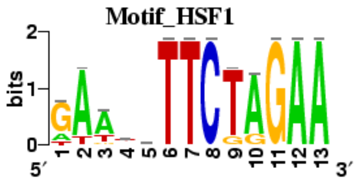				
	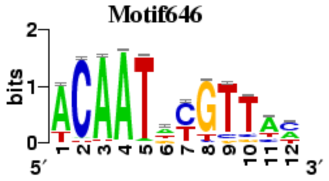	186.47	0.8	0.500	0.013
	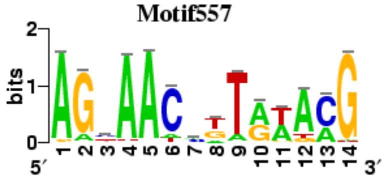	421.12	0.95	0.333	0.001
	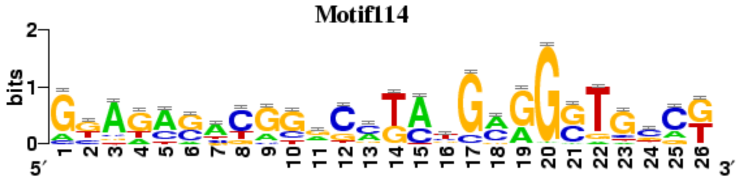	265.84	0.75	0.467	0.008
47	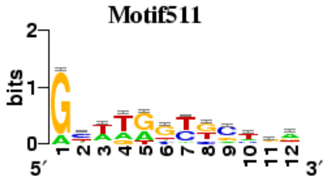	221.83	0.9	0.467	0.010
	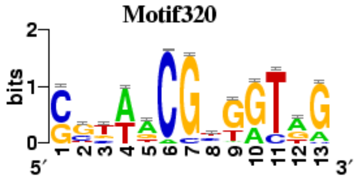	175.22	0.85	0.267	0.003
	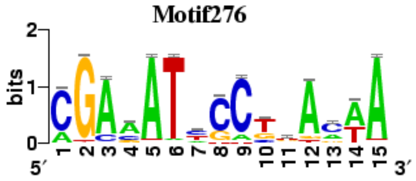	113.71	0.6	0.467	0.020
	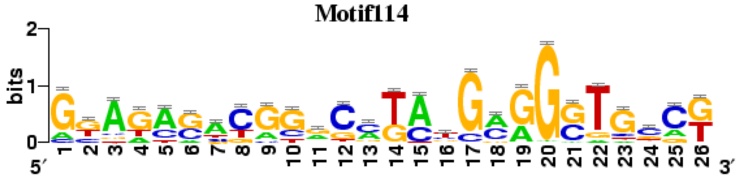	392.76	0.9	0.333	0.001
		295.29	0.9	0.222	0.000

48	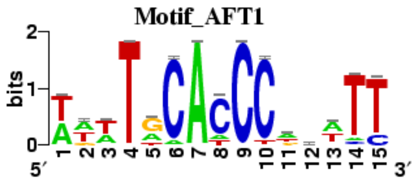				
	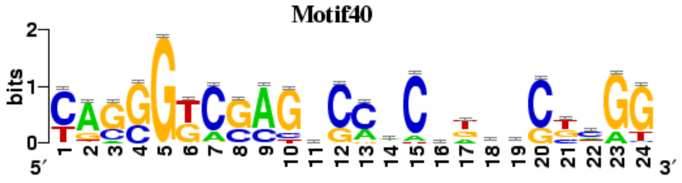	293.71	0.9	0.333	0.001
	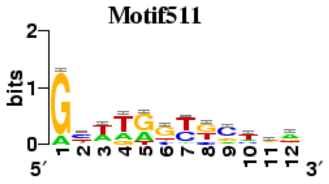	290.70	0.9	0.667	0.010
	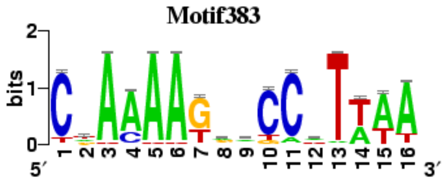	233.19	0.7	0.667	0.013
49	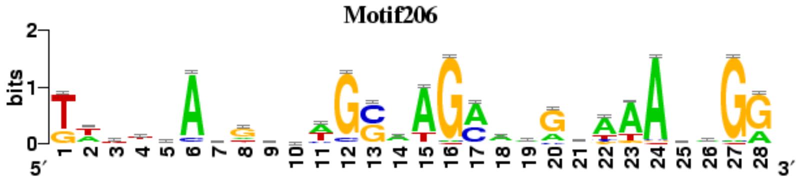	1379.99	0.85	0.636	0.000
	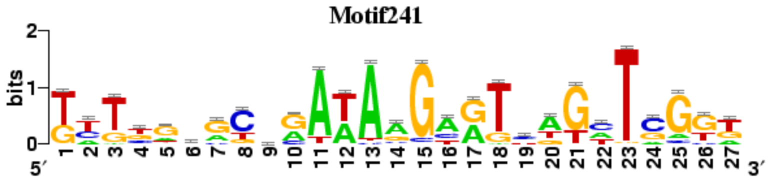	1379.99	0.7	0.636	0.000
	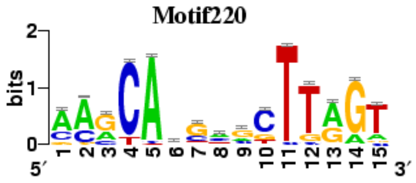	1149.44	0.9	0.545	0.000
	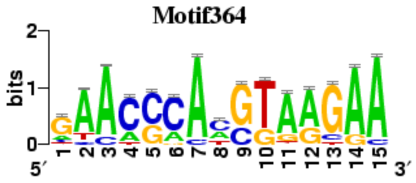	749.08	0.8	0.636	0.003
		715.68	0.9	0.545	0.002

	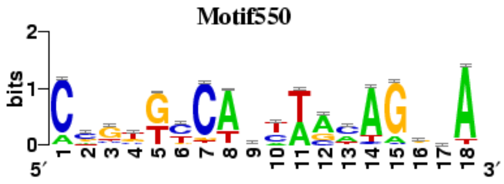				

CV03:

Cluster	Motif	Chi-square statistic	Score cutoff	Posterior probability within cluster	Posterior probability outside cluster
1	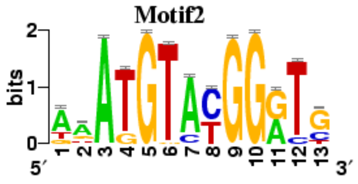	684.70	0.5	0.624	0.024
	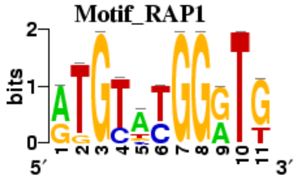	442.99	0.8	0.545	0.035
	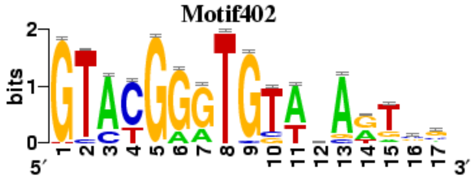	385.28	0.5	0.317	0.009
	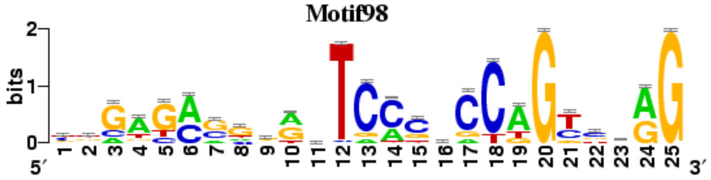	266.87	0.5	0.257	0.010
	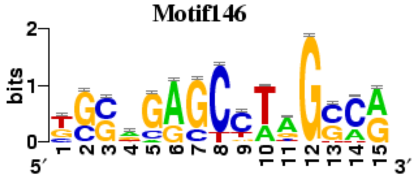	261.26	0.6	0.317	0.019
	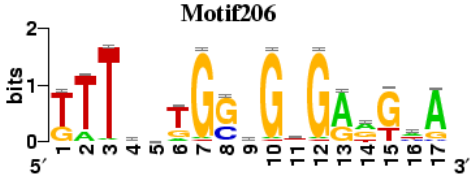	293.00	0.75	0.239	0.006
	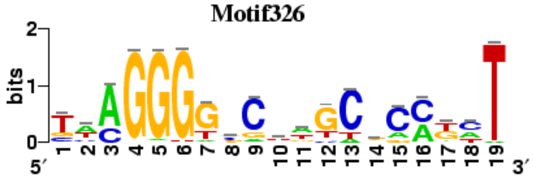	262.89	0.65	0.185	0.003

2	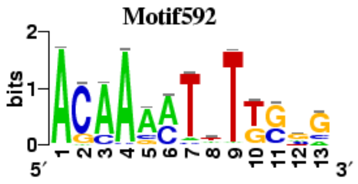	262.89	0.85	0.185	0.003
	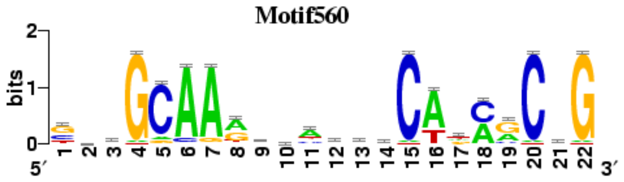	235.06	0.75	0.163	0.003
	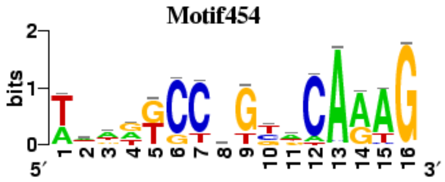	224.04	0.8	0.141	0.002
3	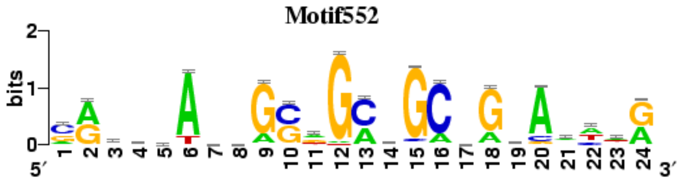	173.79	0.75	0.182	0.007
	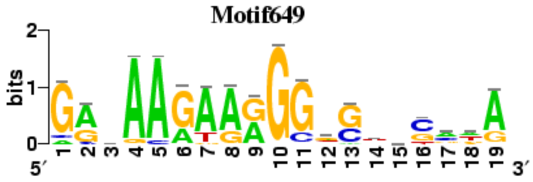	123.81	0.75	0.193	0.014
	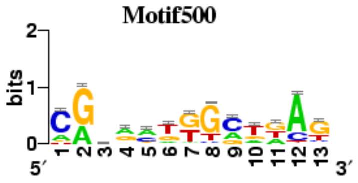	106.42	0.85	0.125	0.006
	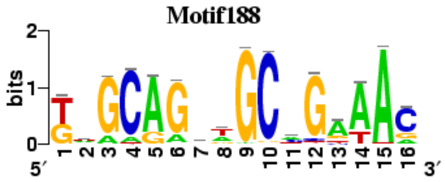	98.06	0.7	0.148	0.010
	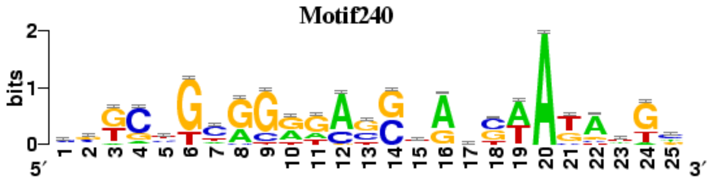	94.37	0.55	0.295	0.046
		401.01	0.5	0.744	0.075

4	Motif1 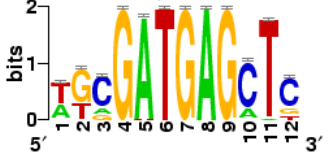				
	Motif_PAC 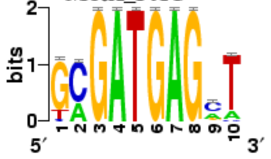	351.63	0.75	0.733	0.084
	Motif4 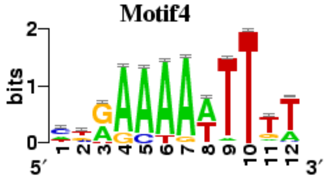	243.95	0.75	0.779	0.140
	Motif213 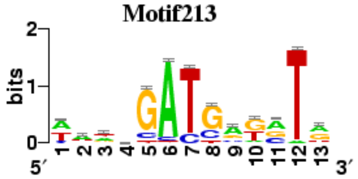	151.13	0.7	0.674	0.156
	Motif195 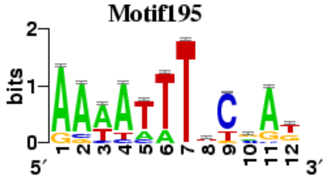	148.09	0.7	0.674	0.159
5	Motif44 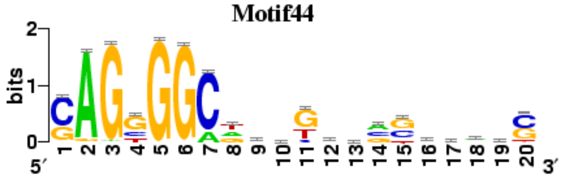	321.11	0.75	0.243	0.004
	Motif58 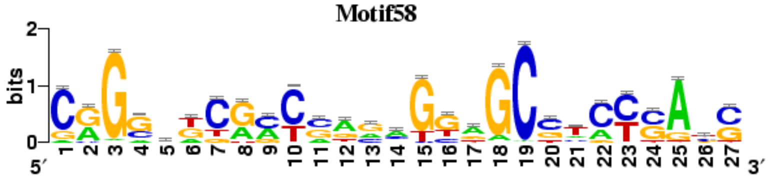	267.86	0.5	0.271	0.008
	Motif31 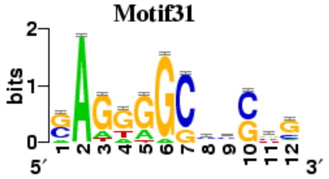	265.84	0.75	0.400	0.024
		245.14	0.5	0.257	0.008

	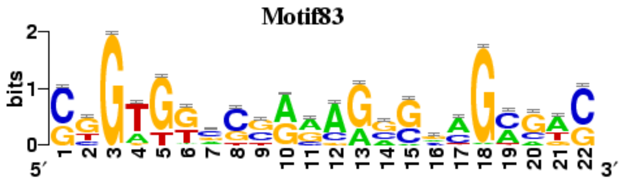				
	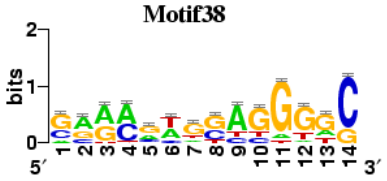	222.94	0.85	0.157	0.002
6	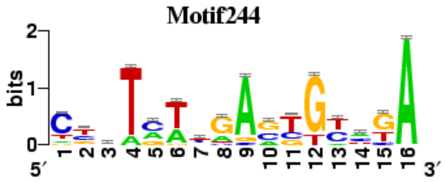	152.64	0.8	0.232	0.013
	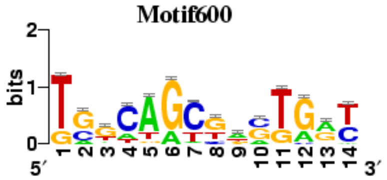	78.88	0.85	0.087	0.002
	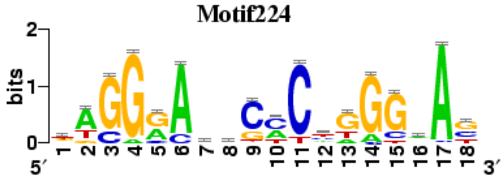	29.87	0.95	0.029	0.000
	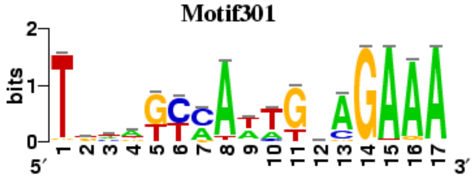	29.87	0.95	0.029	0.000
	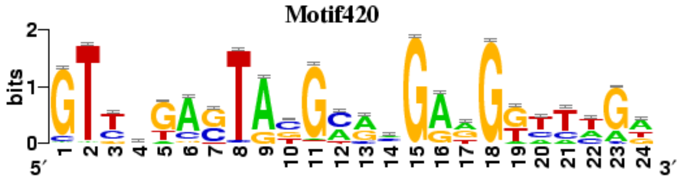	29.87	0.95	0.029	0.000
	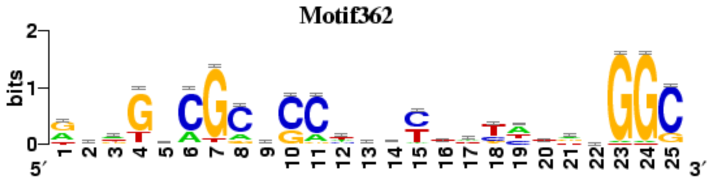	356.02	0.65	0.235	0.002
		259.10	0.7	0.176	0.002

7	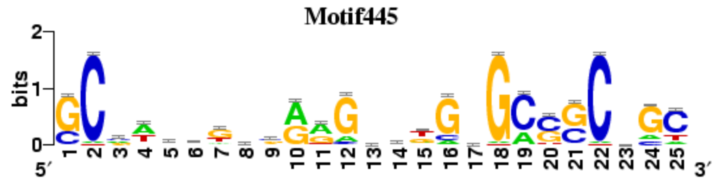				
	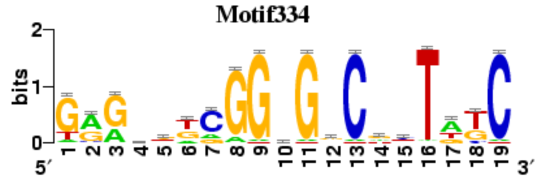	240.48	0.7	0.176	0.002
	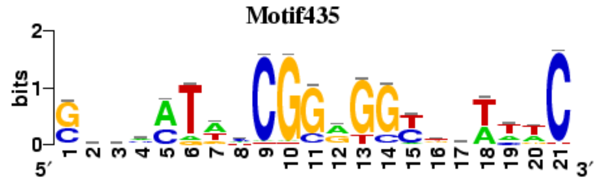	240.48	0.75	0.176	0.002
	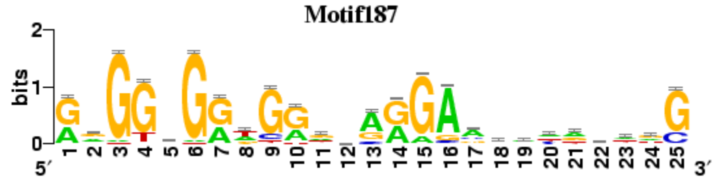	233.06	0.7	0.235	0.007
8	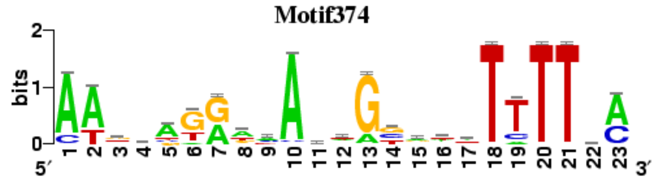	215.52	0.8	0.258	0.010
	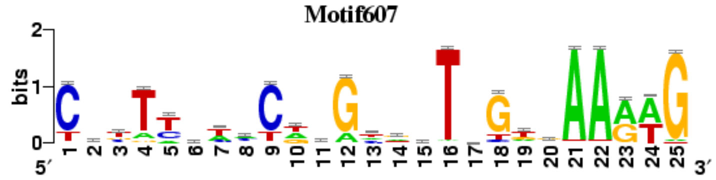	201.71	0.75	0.212	0.006
	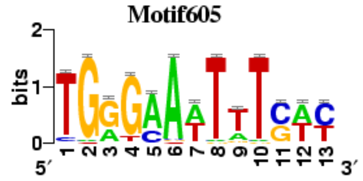	186.71	0.8	0.197	0.006
	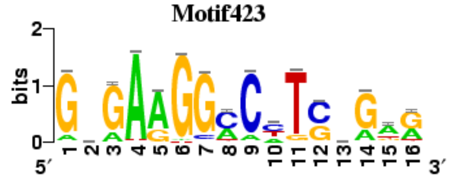	175.89	0.85	0.152	0.003
		159.79	0.9	0.106	0.001

	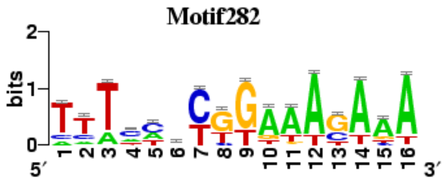				
9	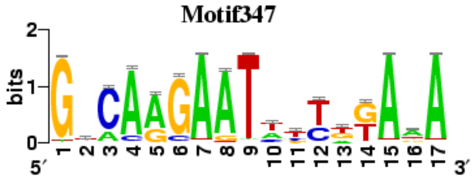	277.03	0.75	0.177	0.001
	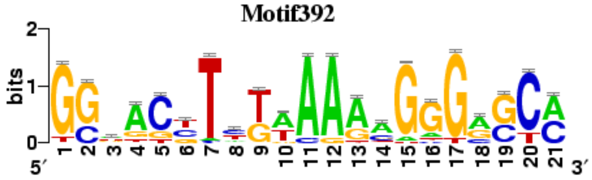	248.06	0.6	0.194	0.003
	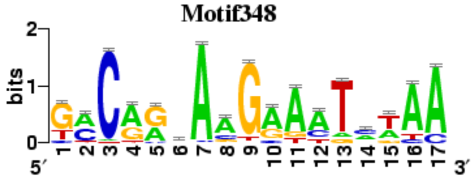	215.44	0.7	0.258	0.009
	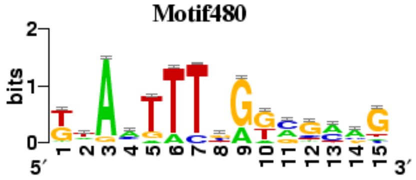	211.92	0.8	0.145	0.001
	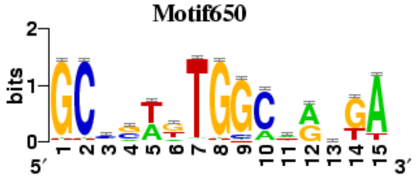	205.75	0.8	0.194	0.004
10	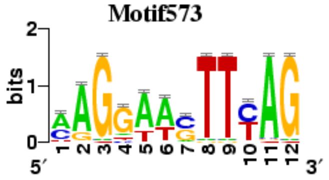	204.90	0.75	0.232	0.007
	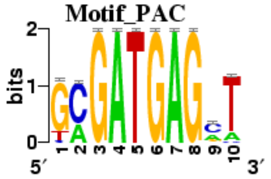	161.92	0.65	0.696	0.115
		147.30	0.85	0.143	0.003

	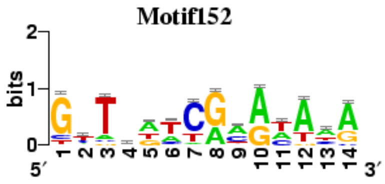				
	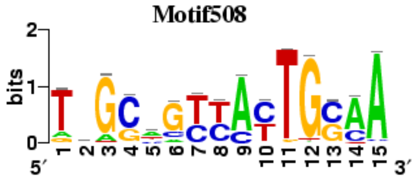	134.97	0.8	0.143	0.003
	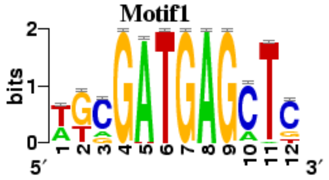	131.21	0.6	0.500	0.070
**11**	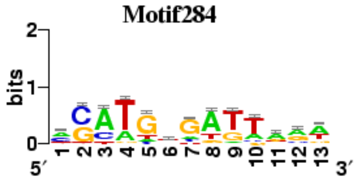	377.52	0.9	0.213	0.001
	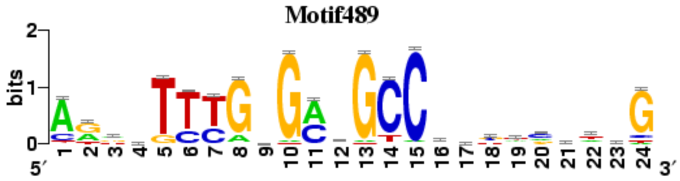	377.52	0.8	0.213	0.001
	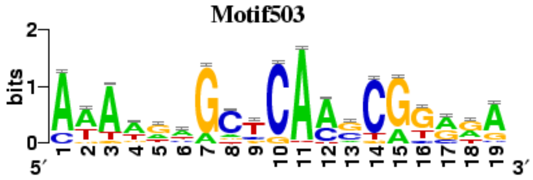	327.02	0.75	0.279	0.005
	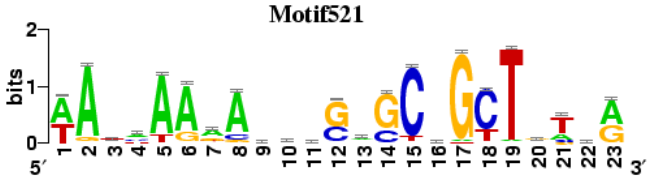	281.28	0.7	0.230	0.004
	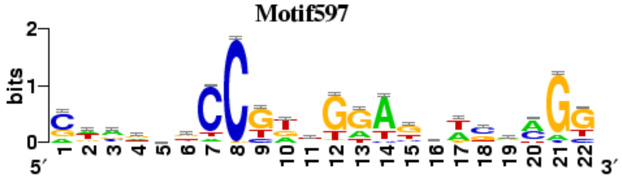	273.33	0.9	0.148	0.000
		367.52	0.75	0.250	0.002

	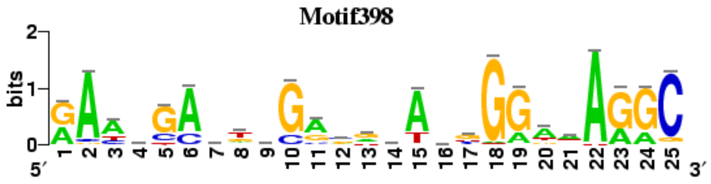				
	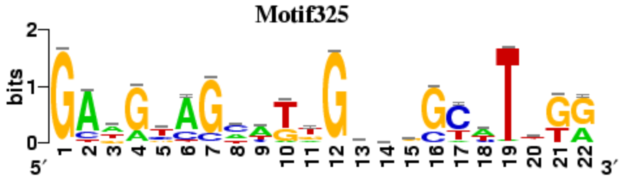	262.36	0.7	0.196	0.002
**12**	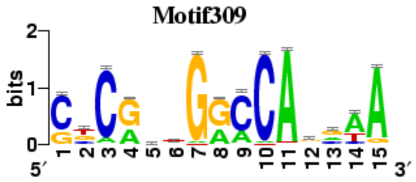	253.66	0.7	0.250	0.006
	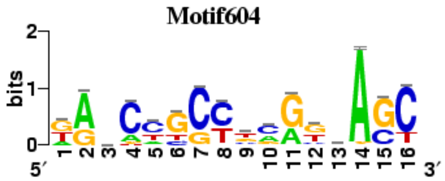	228.20	0.75	0.179	0.002
	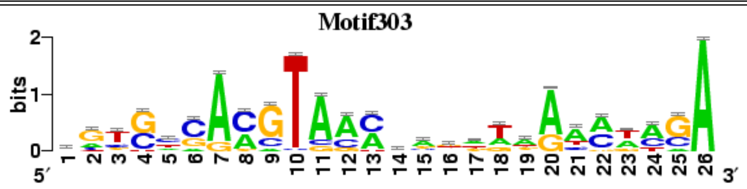	203.03	0.65	0.321	0.016
	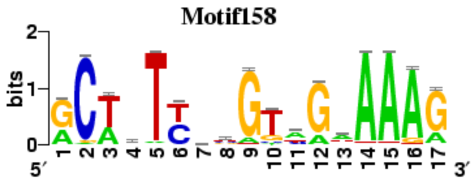	187.43	0.7	0.246	0.009
**13**	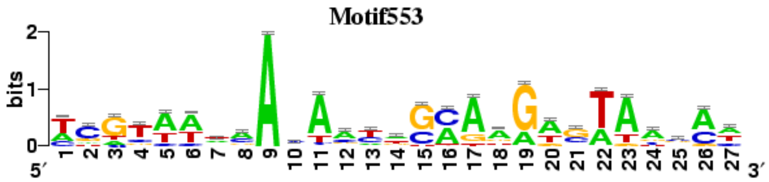	109.90	0.95	0.070	0.000
	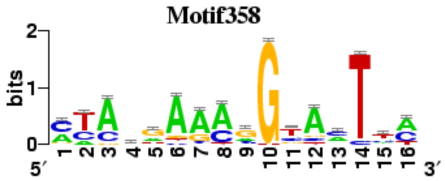	36.60	0.95	0.035	0.000
		36.60	0.95	0.035	0.000

	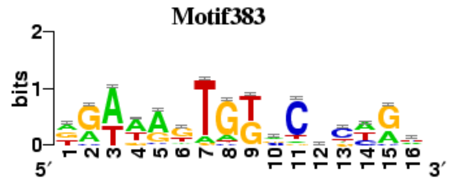				
	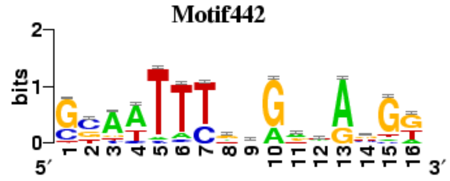	36.60	0.95	0.035	0.000
14	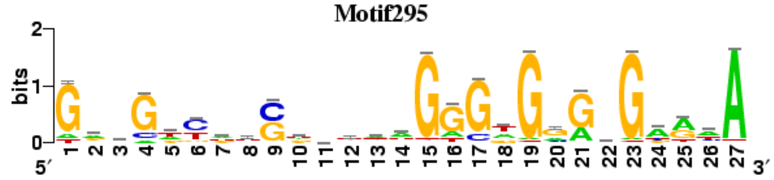	411.68	0.65	0.246	0.001
	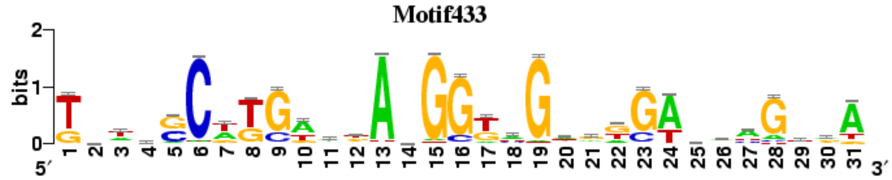	339.25	0.65	0.211	0.001
	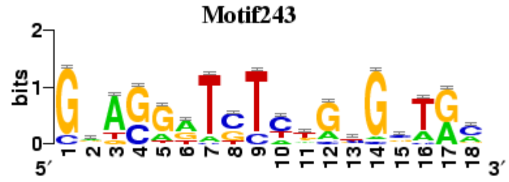	295.95	0.85	0.175	0.001
	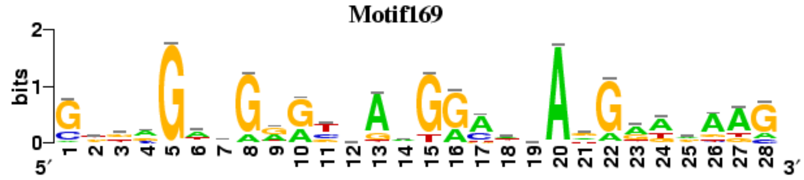	266.35	0.6	0.263	0.006
	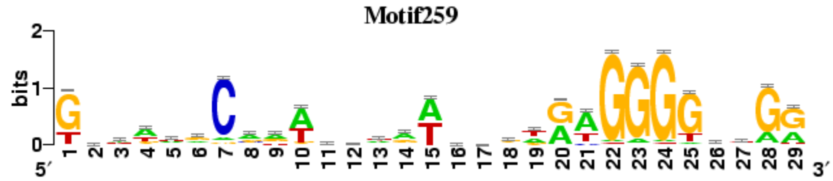	254.07	0.65	0.281	0.008
	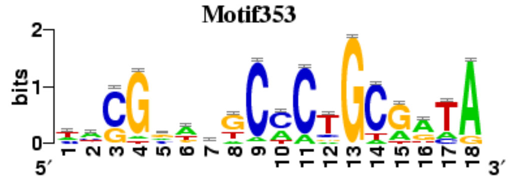	330.53	0.75	0.245	0.003
		315.25	0.7	0.358	0.011

15	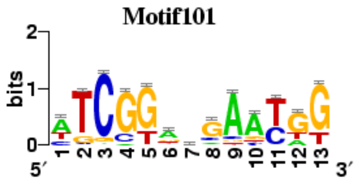				
	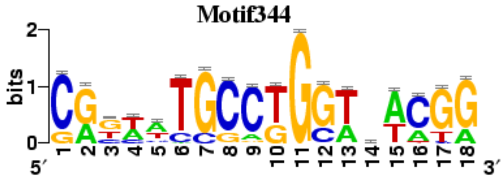	306.72	0.55	0.396	0.015
	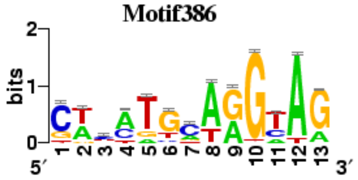	263.55	0.8	0.245	0.005
	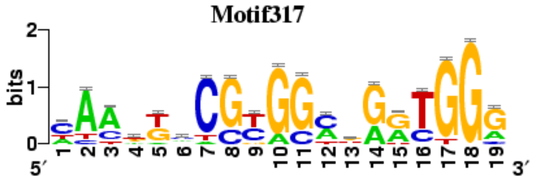	238.38	0.65	0.283	0.009
16	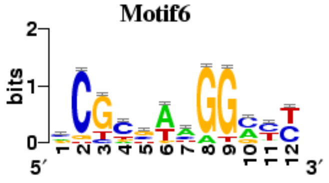	70.88	0.9	0.089	0.002
	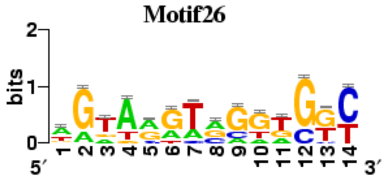	37.30	0.95	0.036	0.000
	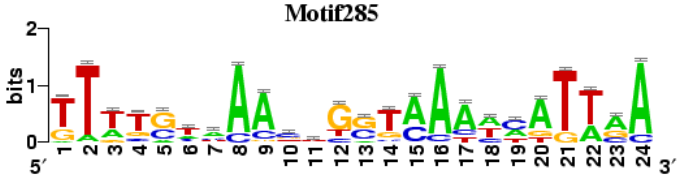	37.30	0.9	0.036	0.000
	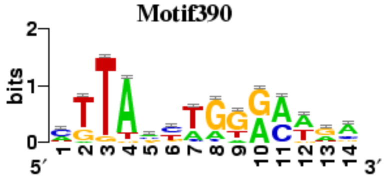	37.30	0.95	0.036	0.000
		37.30	0.95	0.036	0.000

	Motif_MCM1 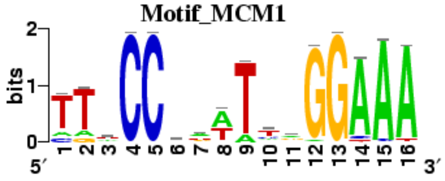				
17	Motif421 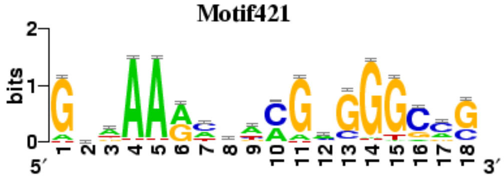	132.34	0.7	0.151	0.004
	Motif82 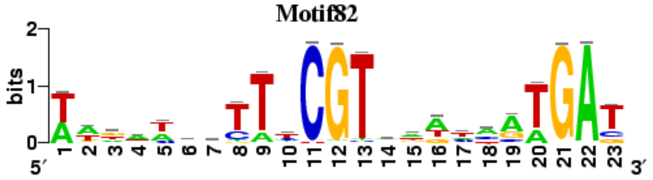	122.20	0.6	0.415	0.049
	Motif62 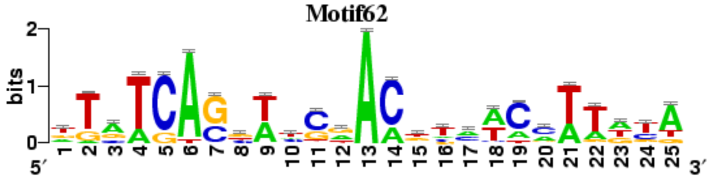	118.45	0.5	0.302	0.025
	Motif630 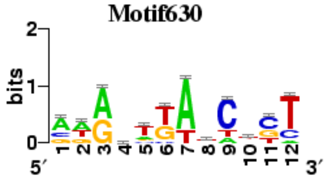	87.62	0.95	0.075	0.001
	Motif19 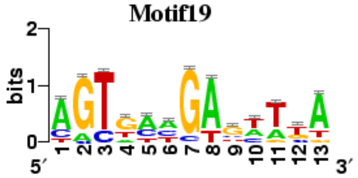	72.43	0.75	0.302	0.041
18	Motif595 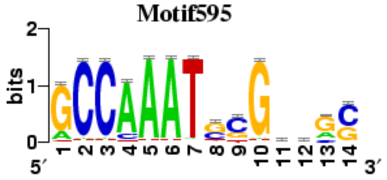	211.51	0.75	0.188	0.003
	Motif436 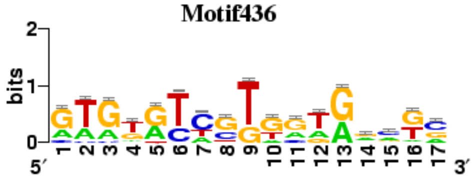	160.21	0.8	0.167	0.003
		137.15	0.85	0.167	0.004

	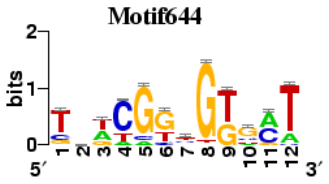				
	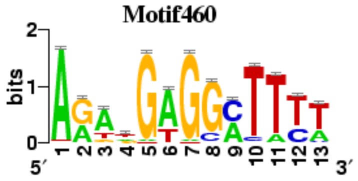	136.76	0.75	0.271	0.015
	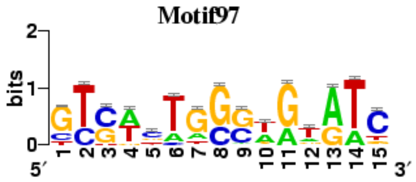	123.11	0.75	0.271	0.017
19	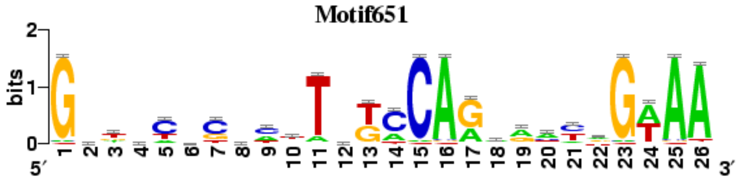	369.26	0.75	0.245	0.002
	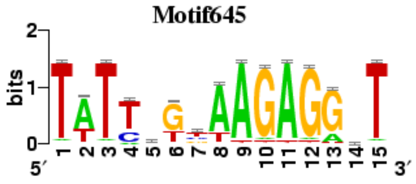	215.41	0.8	0.122	0.000
	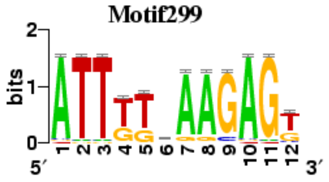	207.01	0.85	0.224	0.005
	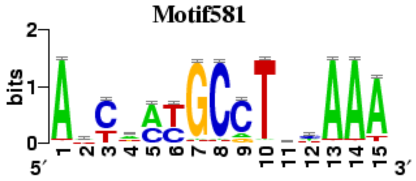	187.39	0.8	0.163	0.002
	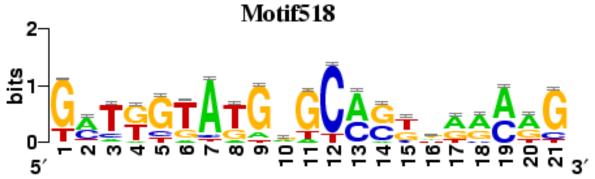	127.81	0.5	0.245	0.013
		508.46	0.85	0.261	0.000

	Motif218 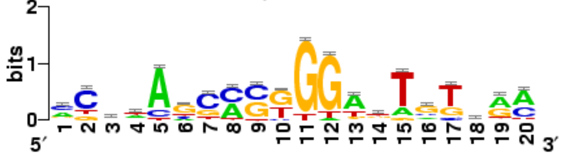				
	Motif133 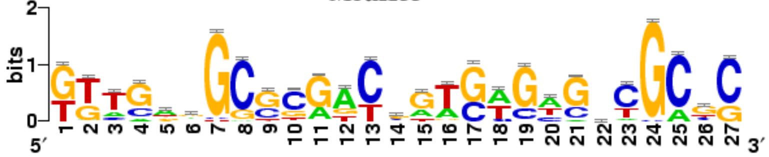	489.05	0.6	0.348	0.003
**20**	Motif312 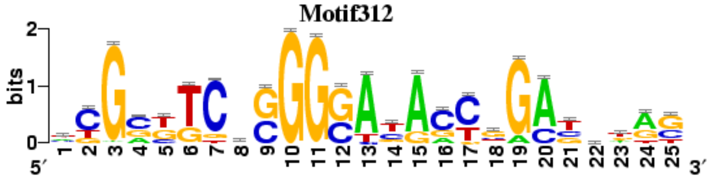	392.60	0.5	0.391	0.008
	Motif126 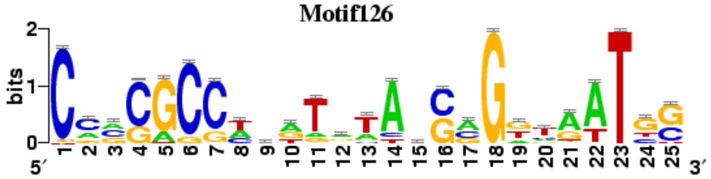	323.53	0.5	0.435	0.015
	Motif156 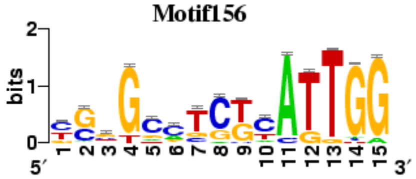	322.18	0.65	0.261	0.003
	Motif378 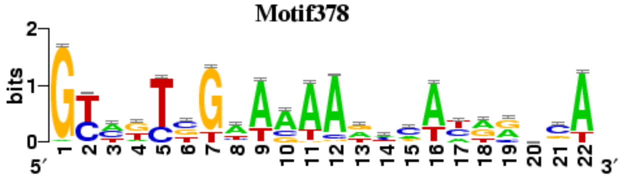	292.48	0.85	0.196	0.001
**21**	Motif485 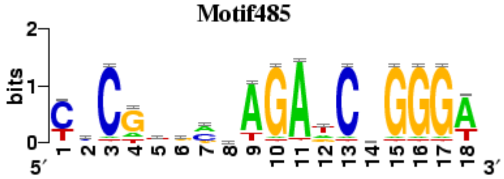	235.54	0.75	0.152	0.001
	Motif168 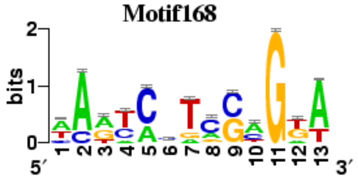	184.27	0.95	0.109	0.000
		183.02	0.85	0.174	0.003

	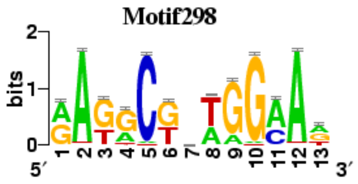				
	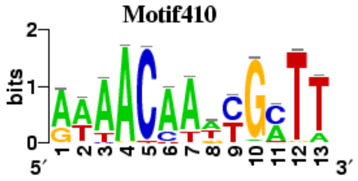	140.50	0.8	0.130	0.002
22	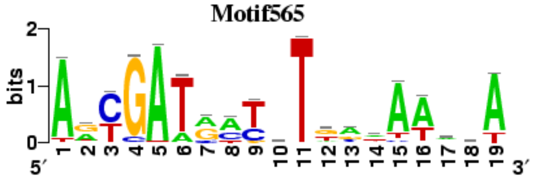	135.58	0.7	0.341	0.024
	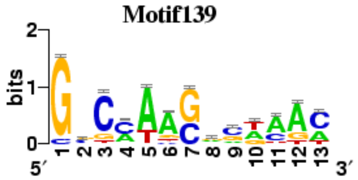	63.05	0.95	0.068	0.001
	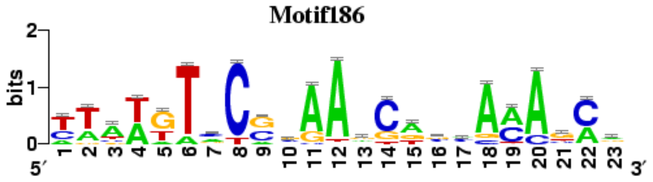	48.24	0.95	0.045	0.000
	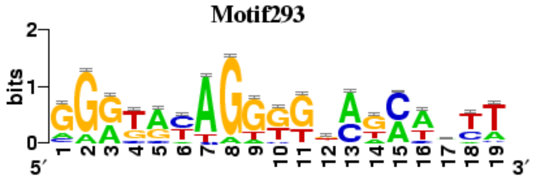	48.24	0.95	0.045	0.000
	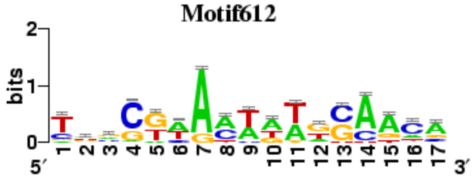	46.06	0.8	0.114	0.006
	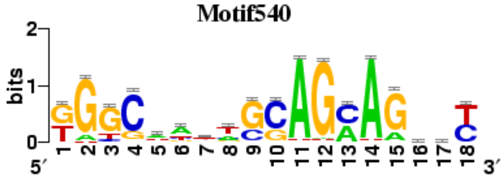	299.50	0.7	0.200	0.001
		123.22	0.85	0.111	0.001

23	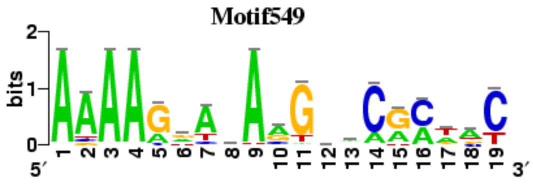				
	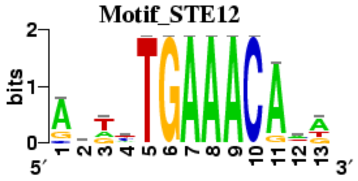	61.52	0.95	0.067	0.001
	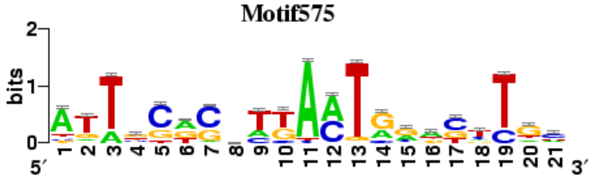	24.07	0.75	0.111	0.011
	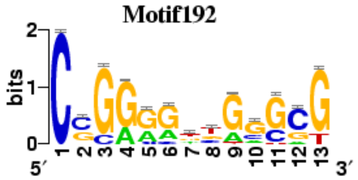	22.57	0.9	0.044	0.001
24	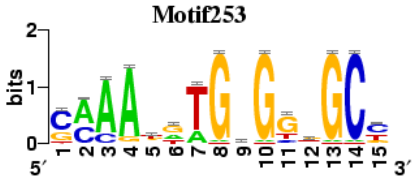	521.11	0.8	0.326	0.002
	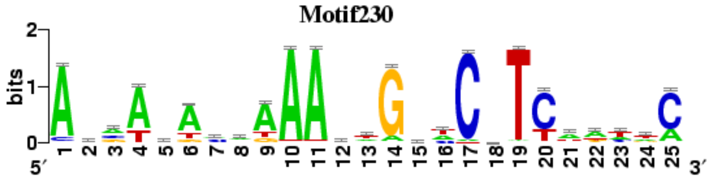	434.74	0.75	0.349	0.004
	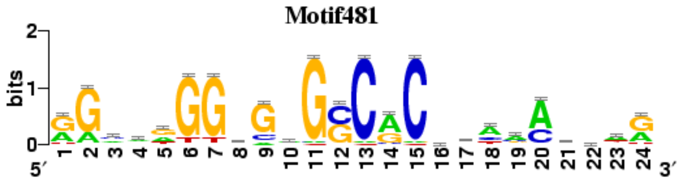	400.40	0.85	0.233	0.001
	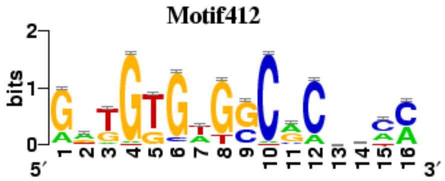	390.76	0.75	0.302	0.003
		379.78	0.65	0.349	0.006

	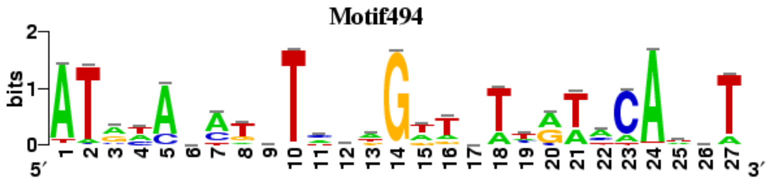				
25	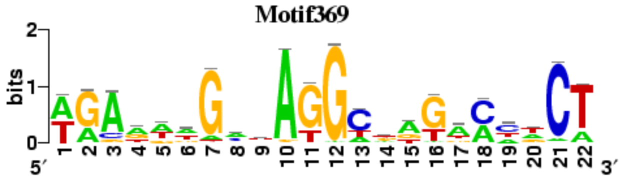	340.74	0.7	0.310	0.005
	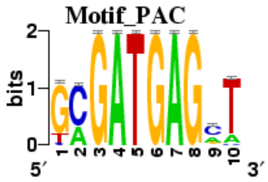	106.92	0.6	0.690	0.129
	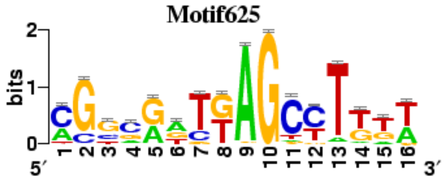	105.19	0.7	0.262	0.017
	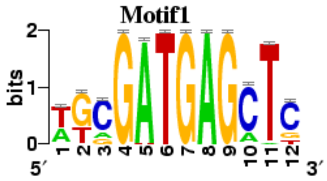	53.87	0.5	0.452	0.096
	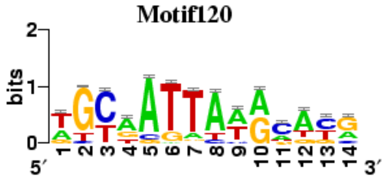	50.70	0.9	0.048	0.000
26	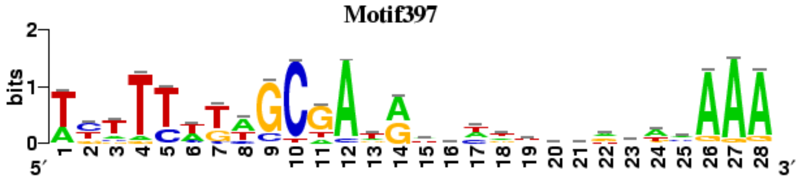	238.94	0.7	0.267	0.006
	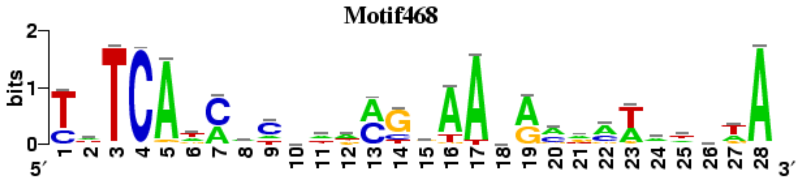	237.06	0.7	0.311	0.010
		141.41	0.95	0.089	0.000

	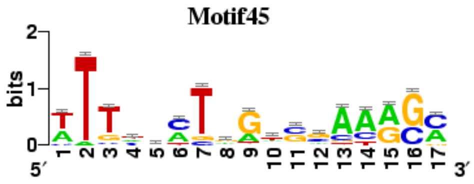				
	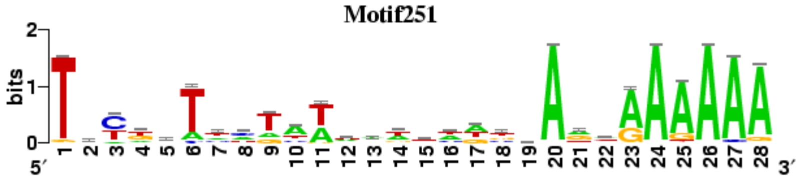	107.44	0.85	0.178	0.007
	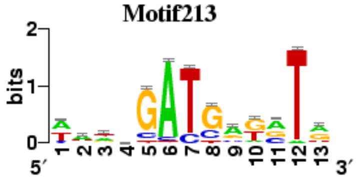	97.60	0.75	0.622	0.118
27	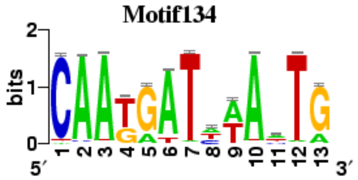	227.66	0.8	0.244	0.005
	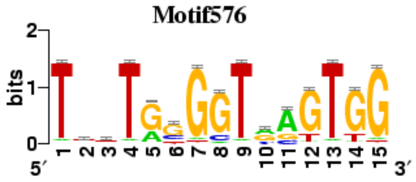	227.56	0.8	0.178	0.002
	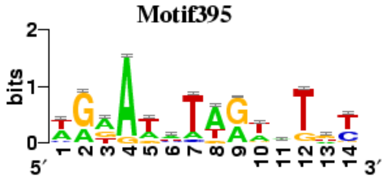	217.67	0.85	0.222	0.004
	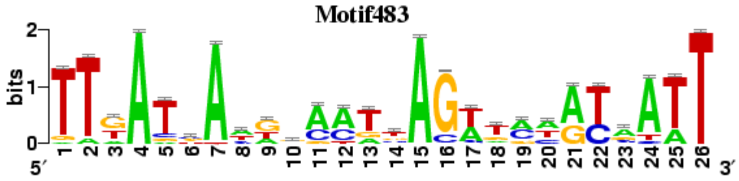	216.11	0.5	0.422	0.024
	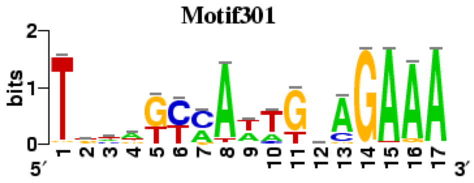	215.98	0.8	0.244	0.006
		477.17	0.7	0.545	0.015

	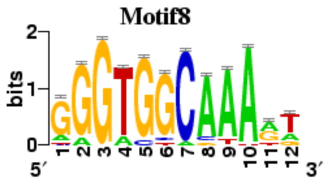				
	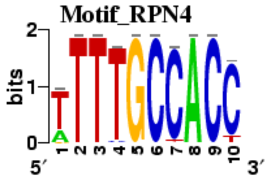	356.81	0.8	0.500	0.018
**28**	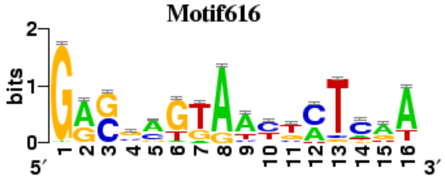	174.58	0.75	0.205	0.005
	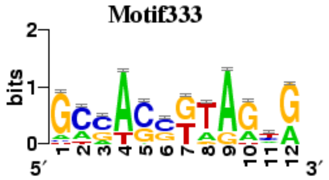	110.36	0.85	0.273	0.018
	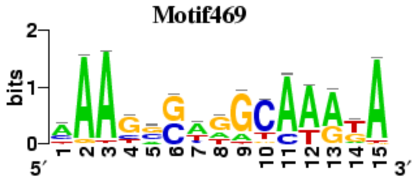	96.52	0.95	0.068	0.000
	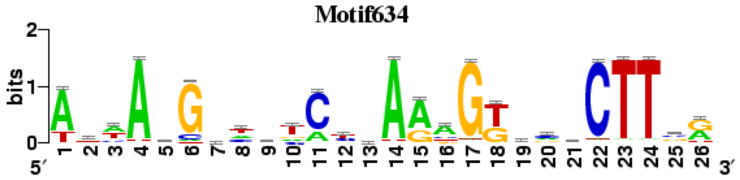	417.62	0.7	0.220	0.000
**29**	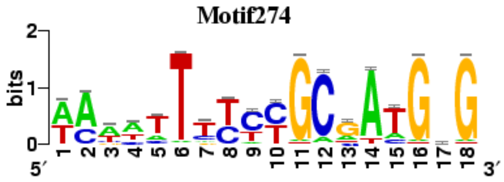	192.37	0.75	0.244	0.006
	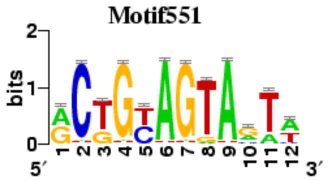	159.39	0.9	0.146	0.002
		140.65	0.8	0.146	0.002

	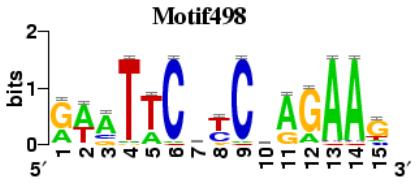				
	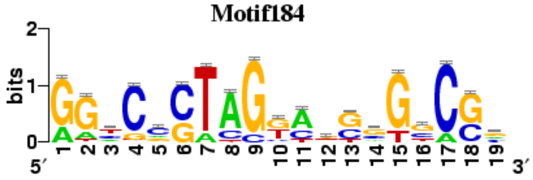	50.12	0.95	0.073	0.001
30	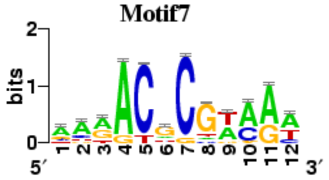	834.11	0.9	0.512	0.003
	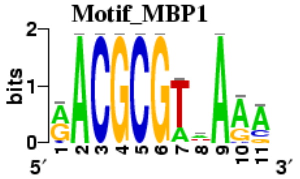	506.13	0.9	0.366	0.003
	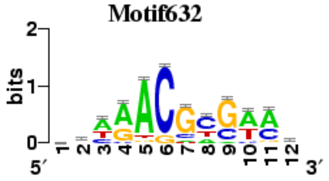	455.59	0.85	0.829	0.043
	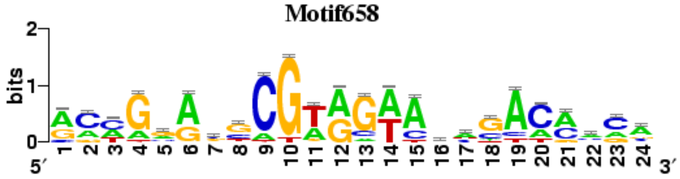	369.61	0.9	0.220	0.001
	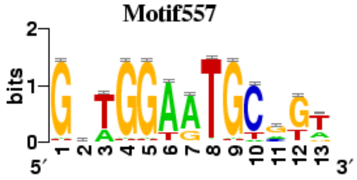	365.24	0.8	0.195	0.000
	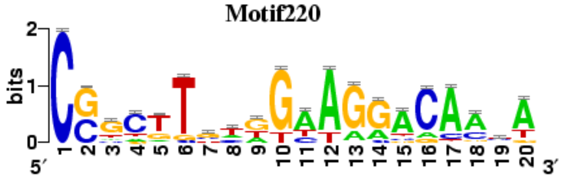	378.26	0.85	0.275	0.002
		234.03	0.85	0.200	0.002

31	Motif365 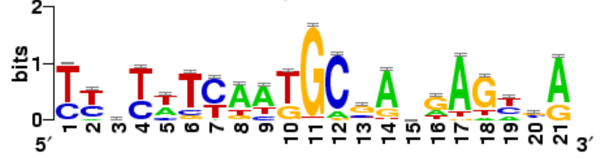				
	Motif624 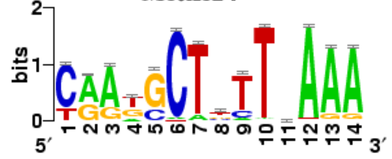	220.57	0.75	0.300	0.009
	Motif555 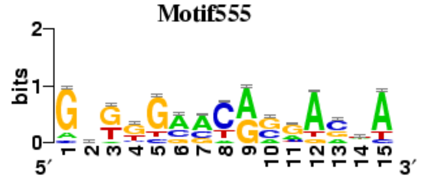	53.42	0.9	0.050	0.000
	Motif314 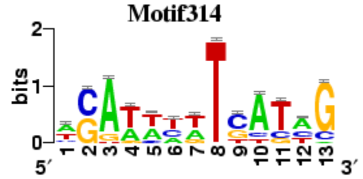	44.69	0.9	0.125	0.007
32	Motif506 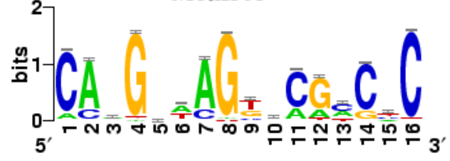	472.49	0.7	0.314	0.002
	Motif534 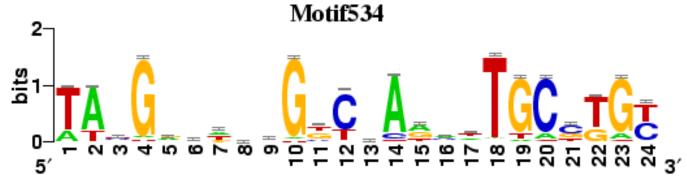	438.44	0.75	0.257	0.001
	Motif227 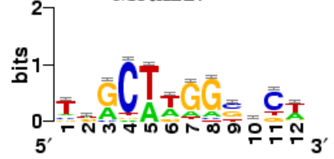	386.87	0.85	0.343	0.004
	Motif257 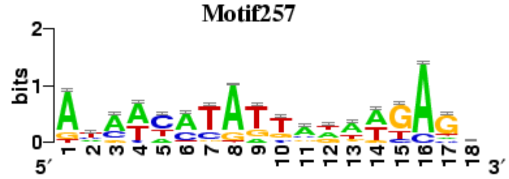	299.28	0.9	0.229	0.002
		299.20	0.8	0.257	0.003

	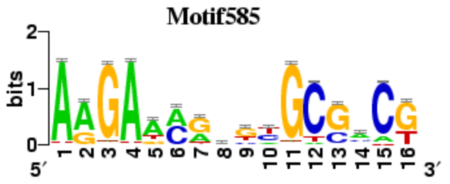				
33	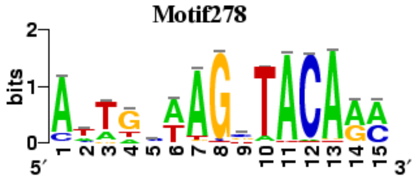	461.30	0.8	0.242	0.000
	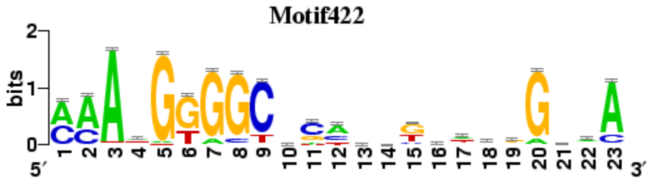	461.30	0.85	0.242	0.000
	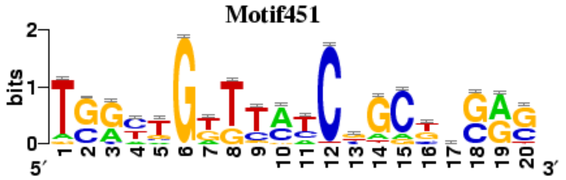	327.20	0.65	0.303	0.004
	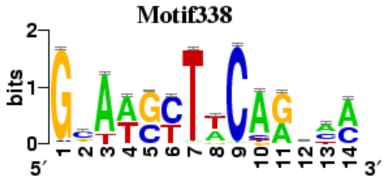	325.98	0.8	0.424	0.010
	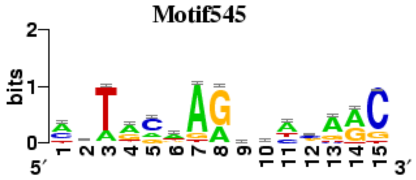	263.22	0.95	0.152	0.000
34	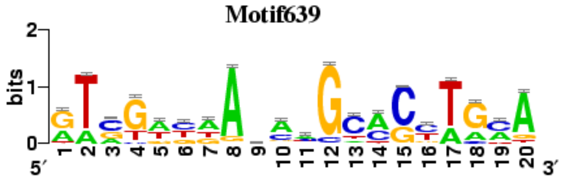	225.25	0.85	0.206	0.002
	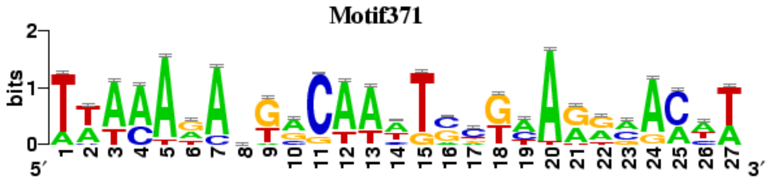	220.68	0.5	0.441	0.020
		92.68	0.95	0.118	0.002

	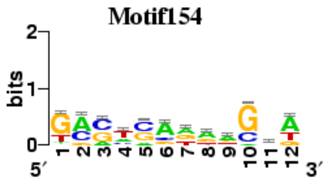				
	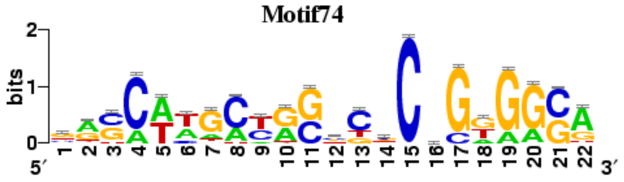	63.62	0.9	0.059	0.000
	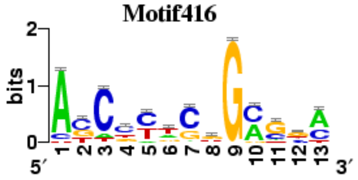	63.62	0.95	0.059	0.000
35	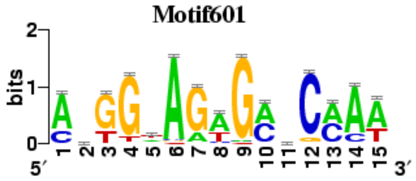	412.84	0.8	0.364	0.004
	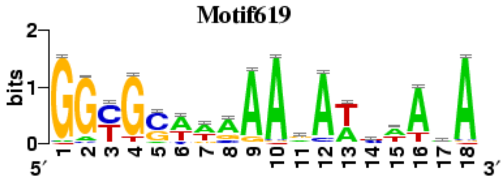	350.03	0.8	0.303	0.003
	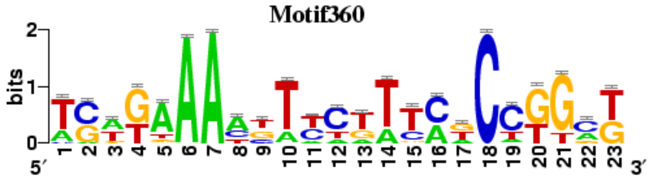	344.78	0.5	0.515	0.016
	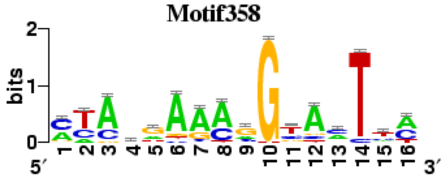	323.07	0.8	0.364	0.007
	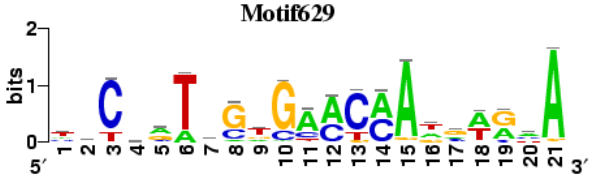	295.42	0.75	0.273	0.003
		211.13	0.85	0.129	0.000

36	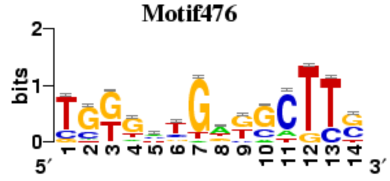				
	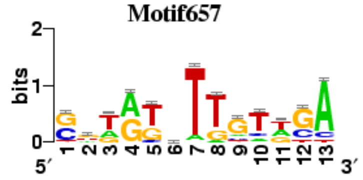	71.16	0.8	0.323	0.029
	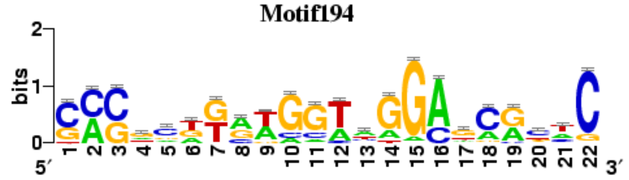	70.31	0.85	0.065	0.000
	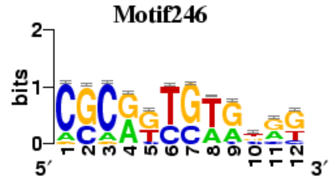	70.31	0.95	0.065	0.000
	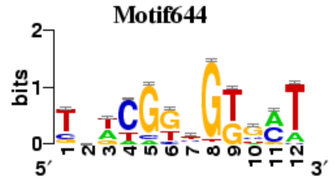	37.48	0.9	0.097	0.003
37	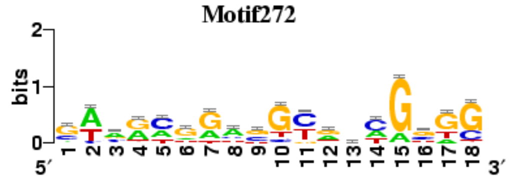	365.98	0.8	0.259	0.001
	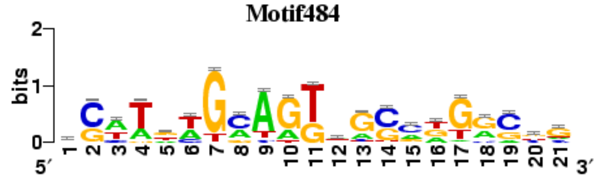	329.63	0.8	0.296	0.003
	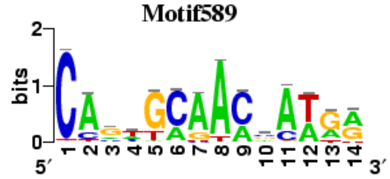	329.63	0.9	0.296	0.003
		327.35	0.8	0.185	0.000

	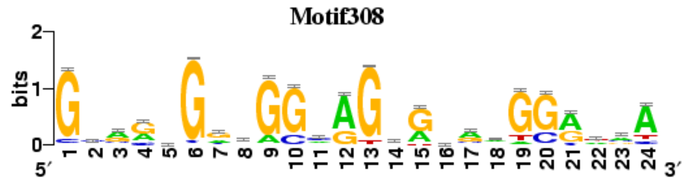				
	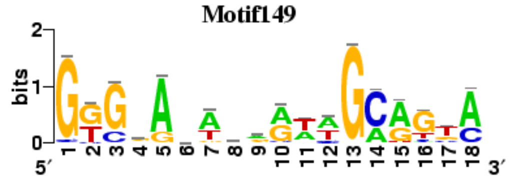	317.41	0.75	0.519	0.015
	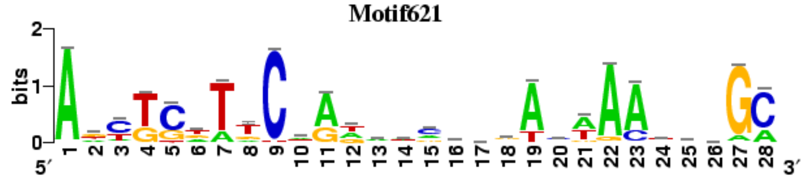	651.13	0.65	0.481	0.003
	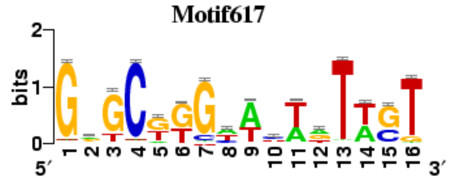	515.69	0.75	0.444	0.004
**38**	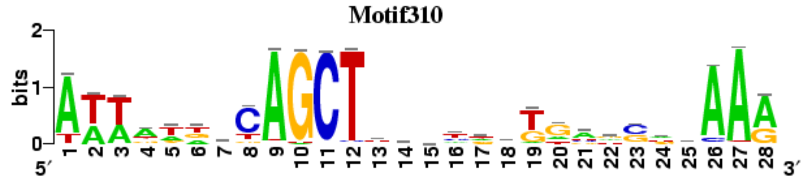	506.95	0.75	0.407	0.003
	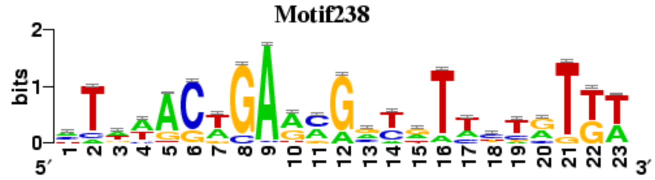	506.59	0.7	0.370	0.002
	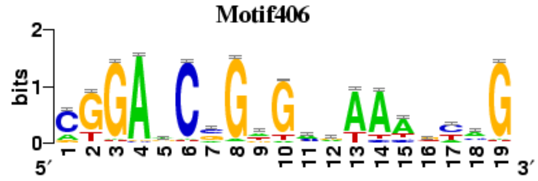	500.48	0.85	0.296	0.001
	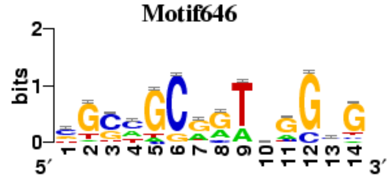	132.93	0.75	0.259	0.008
		44.65	0.85	0.148	0.006

39	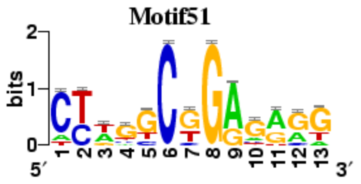				
	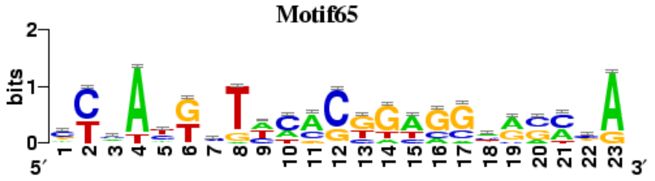	39.88	0.75	0.074	0.001
	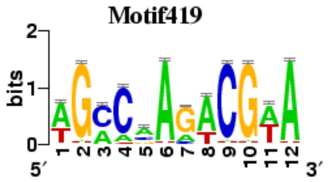	39.88	0.95	0.074	0.001
	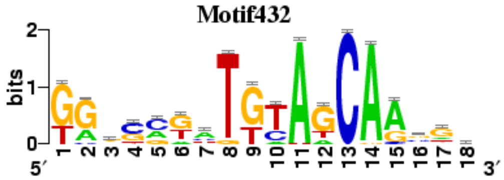	39.88	0.9	0.074	0.001
40	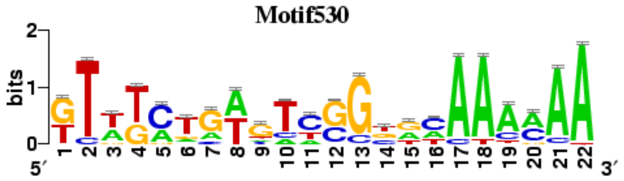	626.90	0.75	0.385	0.001
	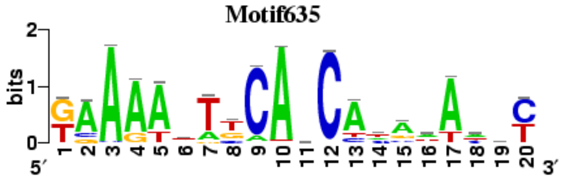	493.55	0.85	0.346	0.002
	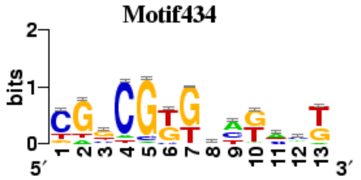	437.55	0.9	0.269	0.001
	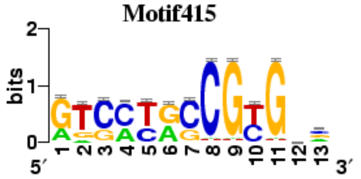	415.56	0.85	0.346	0.003
		381.55	0.8	0.269	0.001

	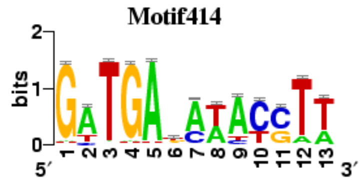				
**41**	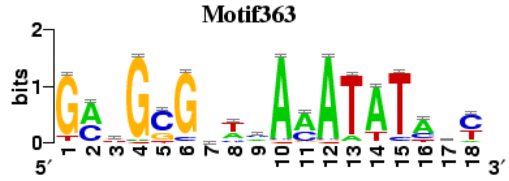	594.59	0.75	0.375	0.001
	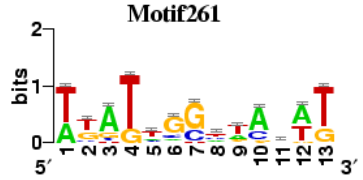	493.30	0.9	0.375	0.002
	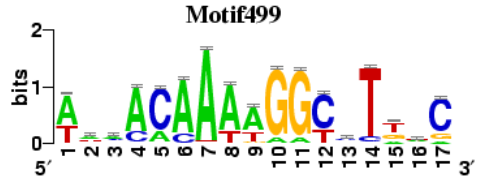	493.30	0.85	0.375	0.002
	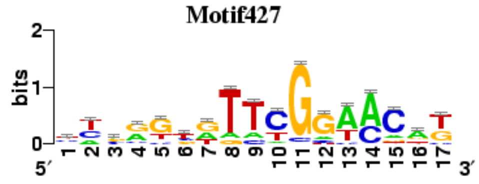	465.90	0.95	0.250	0.000
	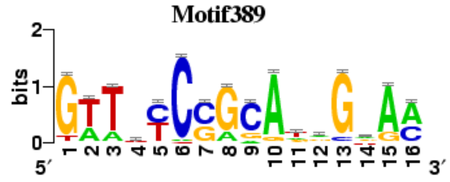	372.54	0.9	0.208	0.000
**42**	NA	NA	NA	NA	NA
**43**	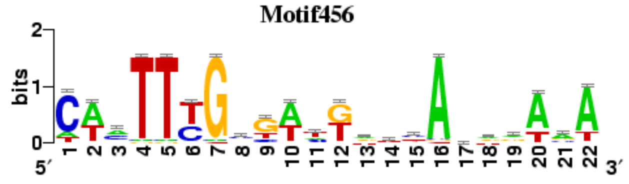	587.71	0.85	0.312	0.000
	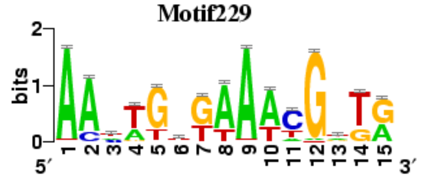	566.77	0.75	0.750	0.010
		549.75	0.75	0.500	0.003

	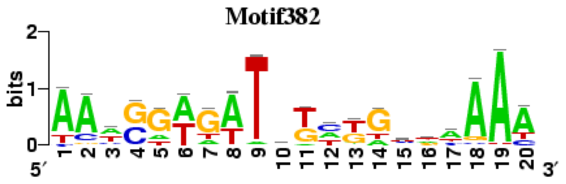				
	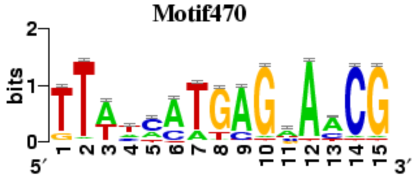	522.64	0.85	0.375	0.001
	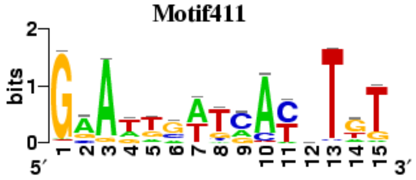	468.79	0.85	0.312	0.001
44	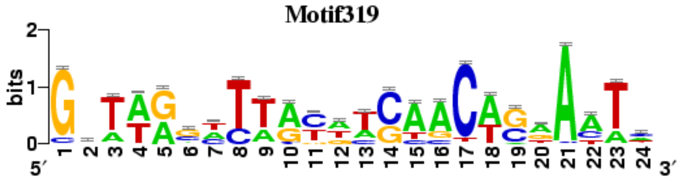	1806.86	0.65	0.938	0.001
	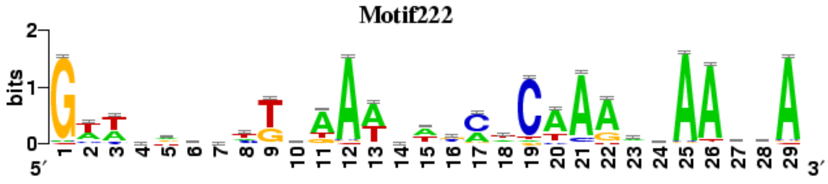	1660.76	0.75	0.875	0.001
	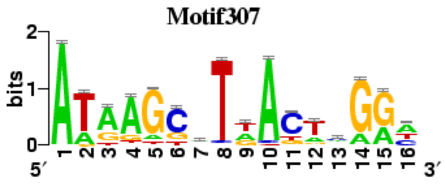	1632.78	0.8	0.812	0.001
	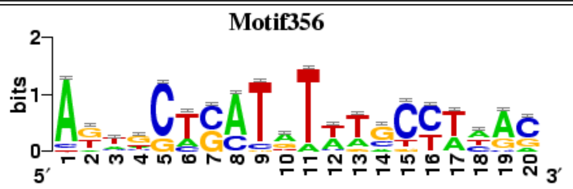	1485.44	0.7	0.750	0.001
	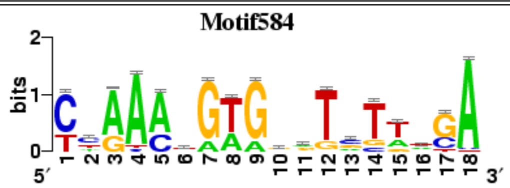	1485.44	0.8	0.750	0.001
		499.66	0.65	0.688	0.010

45	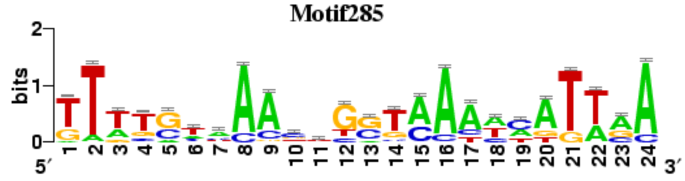				
	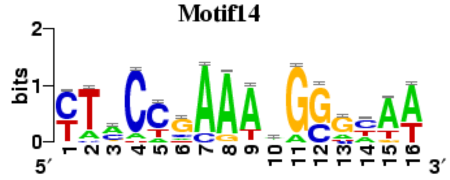	482.57	0.7	0.688	0.010
	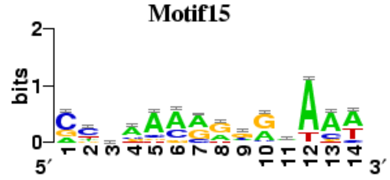	402.18	0.9	0.438	0.004
	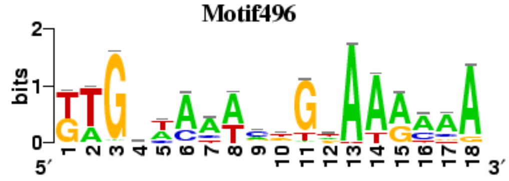	336.31	0.85	0.500	0.007
	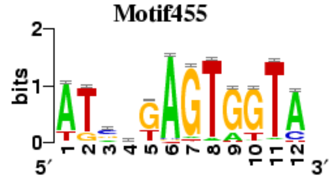	332.89	0.9	0.312	0.002
46	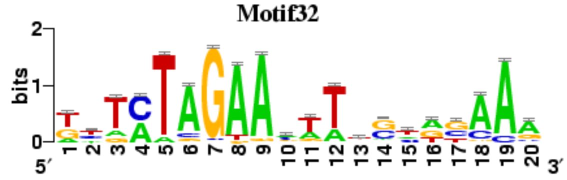	604.60	0.65	0.857	0.012
	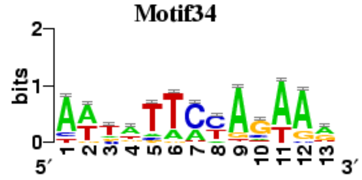	401.45	0.8	0.714	0.013
	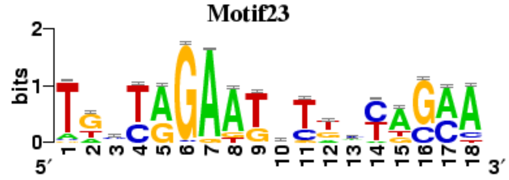	283.28	0.6	0.857	0.030
		171.33	0.95	0.143	0.000

	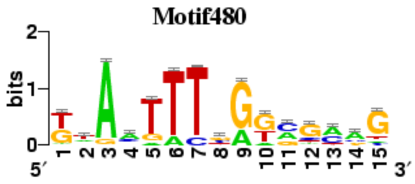				
	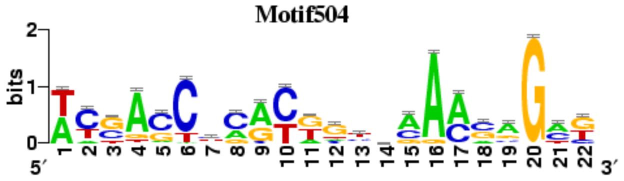	171.33	0.95	0.143	0.000
	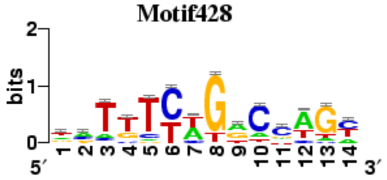	697.50	0.9	0.571	0.003
	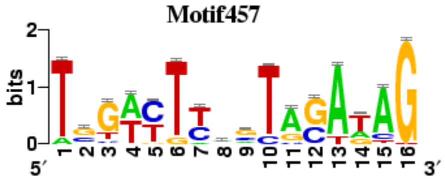	520.64	0.8	0.571	0.005
47	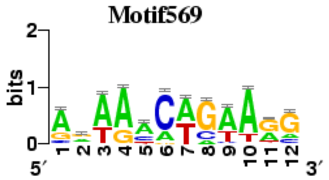	262.16	0.85	0.500	0.009
	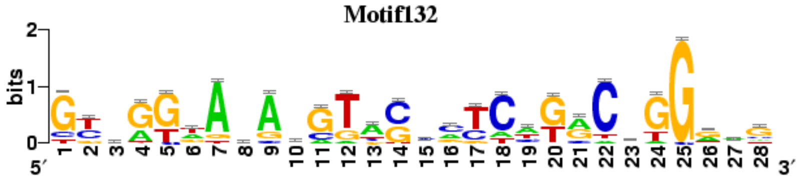	134.94	0.95	0.214	0.002
	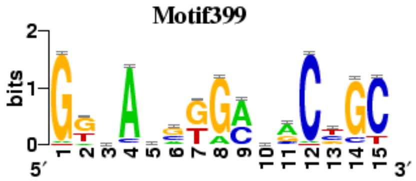	134.94	0.9	0.214	0.002
	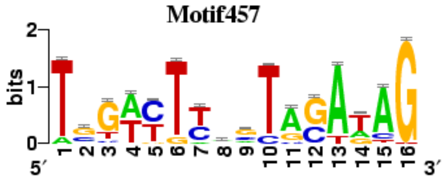	857.91	0.85	0.750	0.003
		455.33	0.9	0.625	0.004

<table>
<tr><td rowspan="4">48</td><td>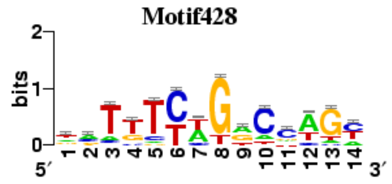</td><td></td><td></td><td></td><td></td></tr>
<tr><td>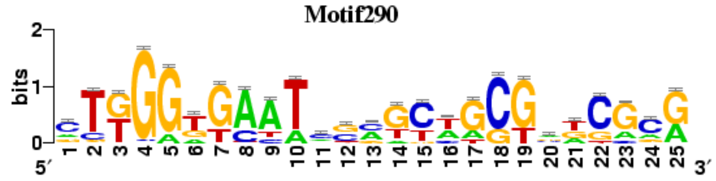</td><td>273.07</td><td>0.65</td><td>0.375</td><td>0.002</td></tr>
<tr><td>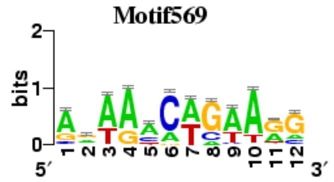</td><td>235.01</td><td>0.85</td><td>0.625</td><td>0.010</td></tr>
<tr><td>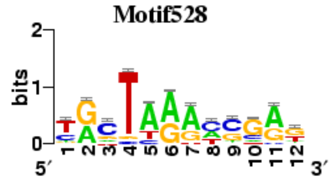</td><td>204.83</td><td>0.8</td><td>0.750</td><td>0.018</td></tr>
<tr><td rowspan="5">49</td><td>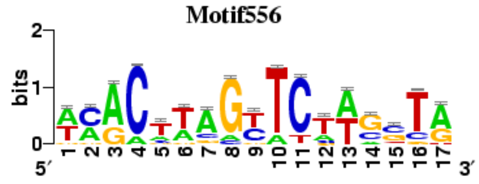</td><td>953.88</td><td>0.8</td><td>0.545</td><td>0.001</td></tr>
<tr><td>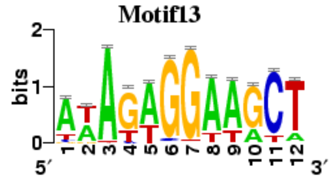</td><td>687.00</td><td>0.95</td><td>0.364</td><td>0.000</td></tr>
<tr><td>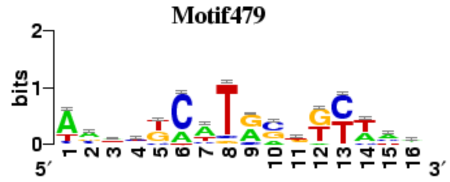</td><td>514.00</td><td>0.95</td><td>0.364</td><td>0.001</td></tr>
<tr><td>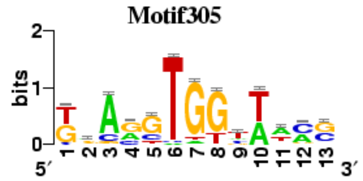</td><td>443.53</td><td>0.75</td><td>0.727</td><td>0.009</td></tr>
<tr><td></td><td>369.44</td><td>0.85</td><td>0.636</td><td>0.008</td></tr>
</table>

	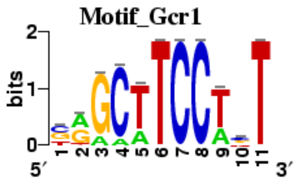				

CV04:

Cluster	Motif	Chi-square statistic	Score cutoff	Posterior probability within cluster	Posterior probability outside cluster
1	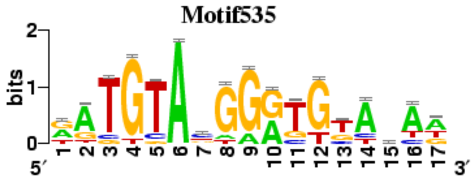	703.98	0.55	0.634	0.024
	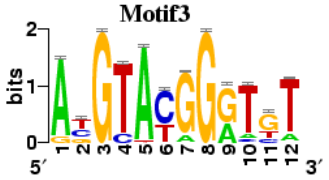	685.59	0.55	0.713	0.036
	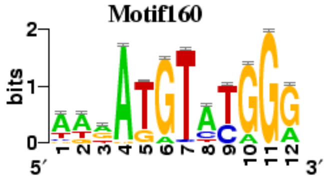	552.18	0.6	0.723	0.053
	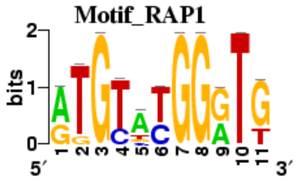	471.27	0.8	0.535	0.030
	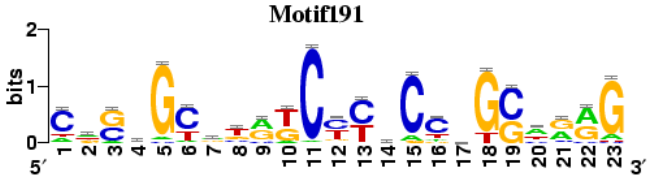	303.04	0.5	0.455	0.038
	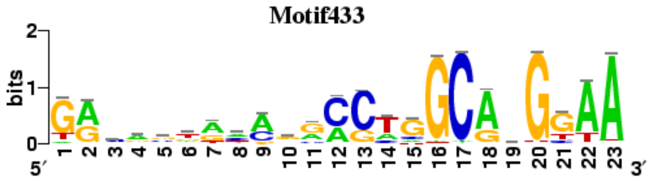	177.53	0.7	0.109	0.001
	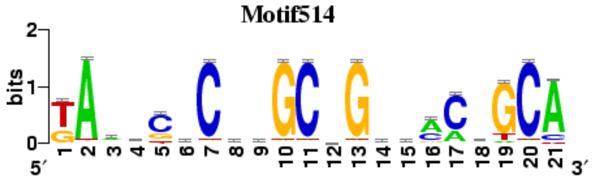	159.84	0.75	0.109	0.002

2	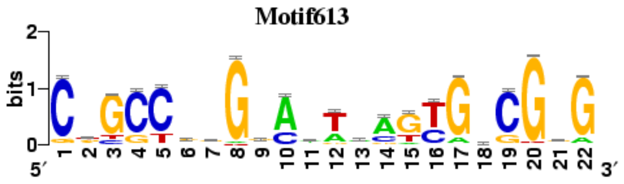	159.84	0.75	0.109	0.002
	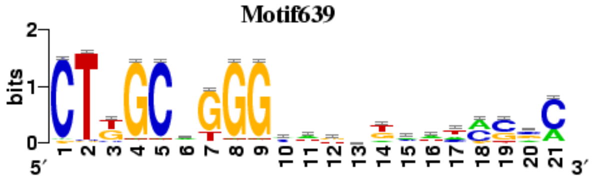	154.76	0.75	0.087	0.001
	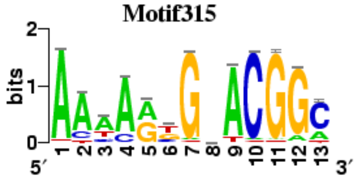	150.39	0.8	0.130	0.004
3	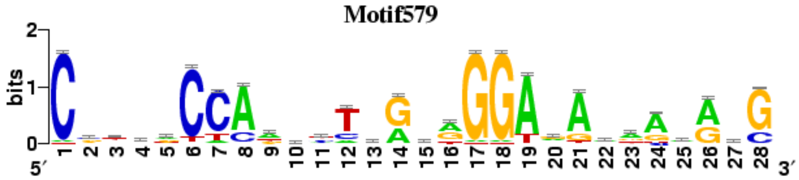	282.38	0.7	0.161	0.001
	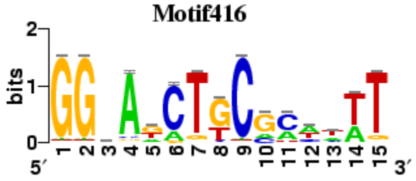	221.35	0.8	0.149	0.002
	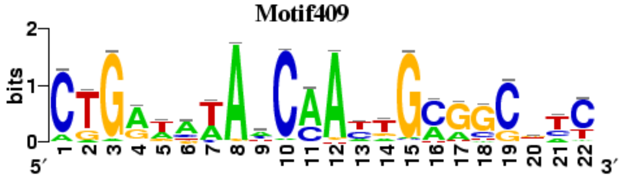	192.70	0.7	0.126	0.002
	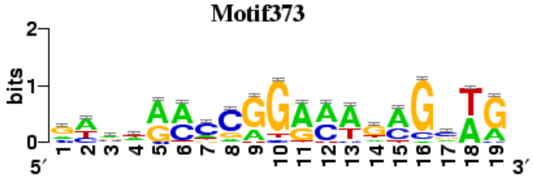	165.26	0.7	0.103	0.001
	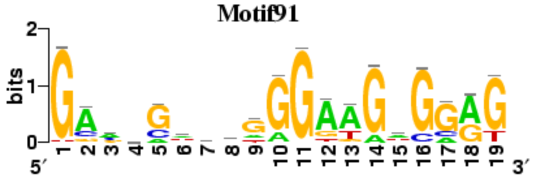	151.23	0.65	0.184	0.009
		353.11	0.5	0.709	0.078

	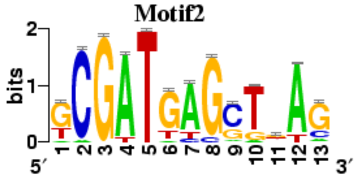				
	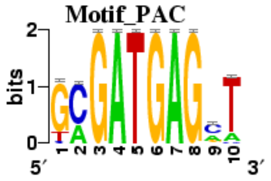	308.77	0.75	0.698	0.087
4	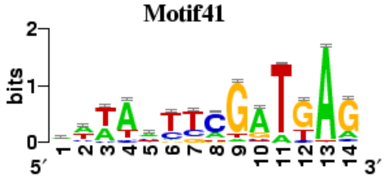	283.76	0.7	0.477	0.040
	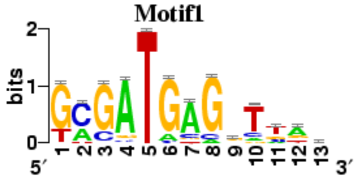	280.34	0.7	0.640	0.079
	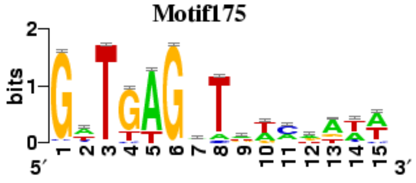	202.08	0.8	0.465	0.056
	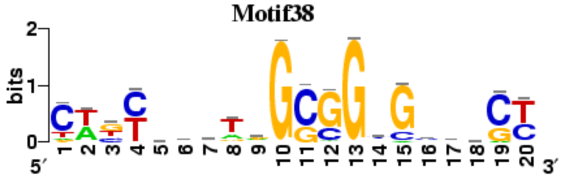	339.41	0.75	0.261	0.004
5	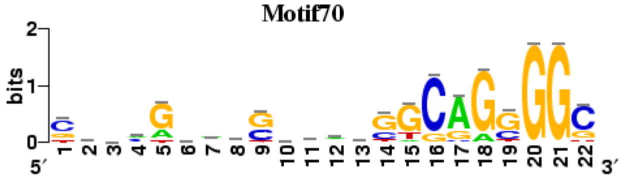	298.78	0.65	0.246	0.005
	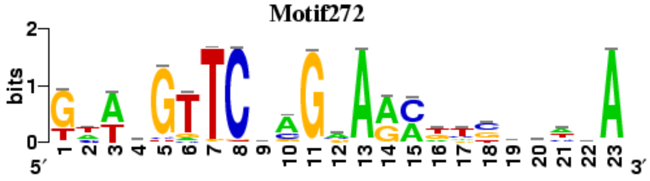	287.85	0.7	0.217	0.003
		255.80	0.6	0.174	0.002

	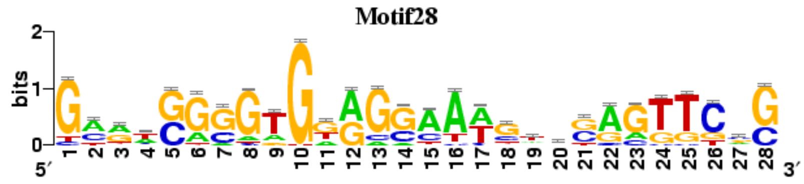				
	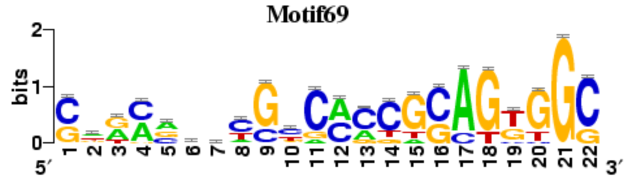	216.64	0.5	0.333	0.020
6	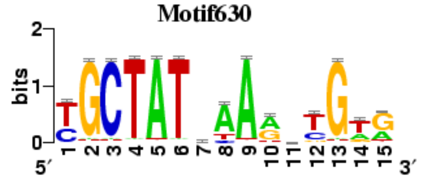	156.40	0.75	0.157	0.004
	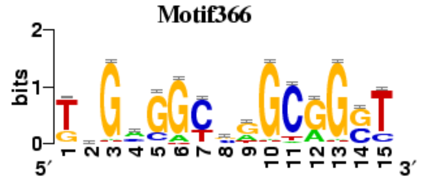	128.89	0.7	0.129	0.003
	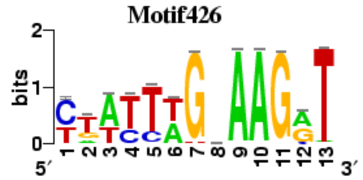	113.15	0.8	0.200	0.013
	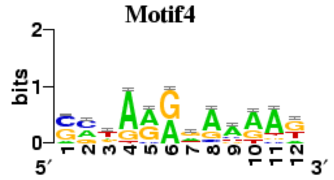	42.13	0.5	0.814	0.970
	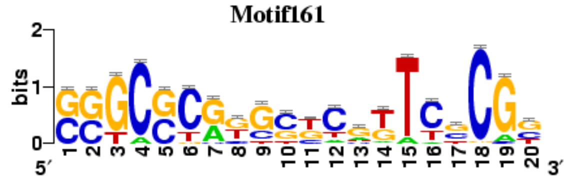	38.05	0.85	0.043	0.001
	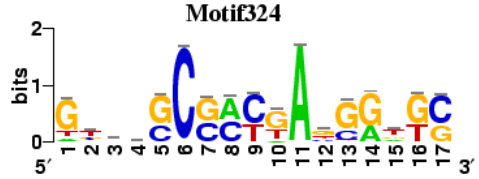	290.88	0.7	0.250	0.005
		260.50	0.65	0.279	0.009

7	Motif358 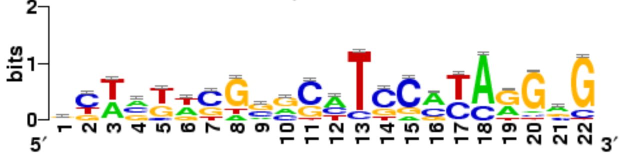				
	Motif217 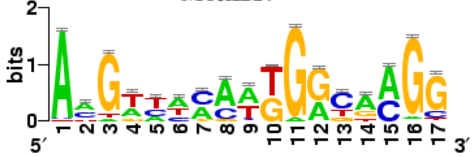	250.35	0.65	0.206	0.004
	Motif442 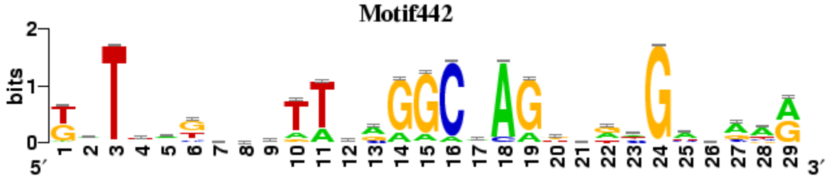	223.76	0.65	0.191	0.004
	Motif162 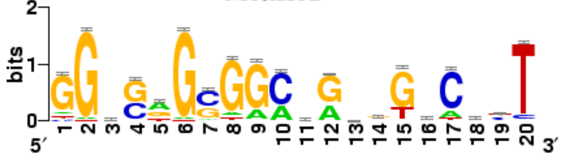	210.47	0.65	0.176	0.003
8	Motif296 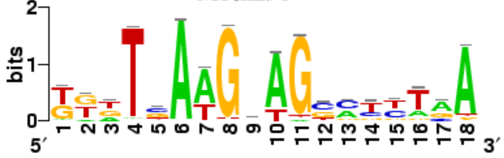	220.48	0.8	0.121	0.000
	Motif615 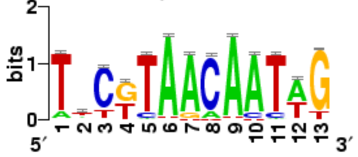	220.48	0.85	0.121	0.000
	Motif624 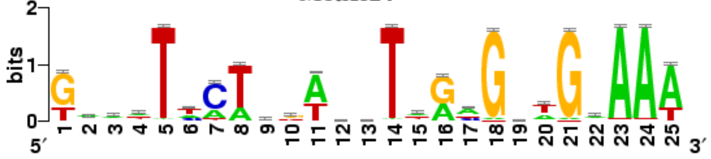	220.03	0.75	0.167	0.002
	Motif357 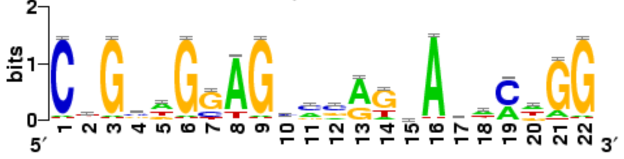	208.62	0.7	0.152	0.002
		204.32	0.85	0.182	0.004

	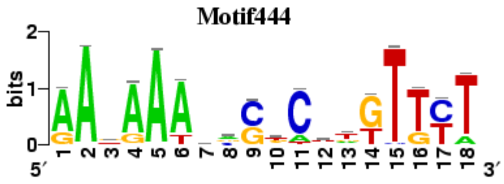				
	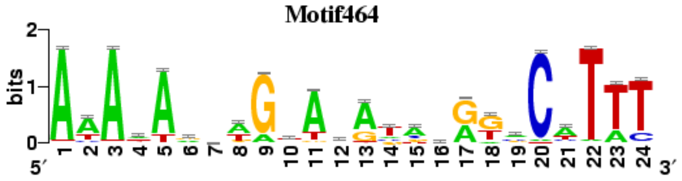	266.66	0.75	0.159	0.001
	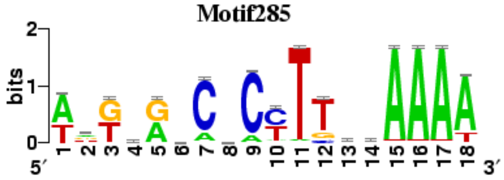	244.50	0.8	0.190	0.003
**9**	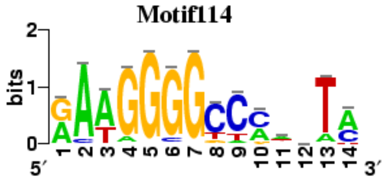	218.22	0.7	0.206	0.005
	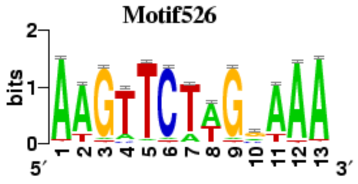	177.25	0.8	0.127	0.001
	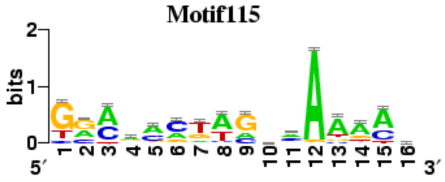	165.77	0.9	0.175	0.005
	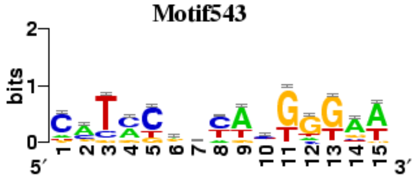	304.19	0.9	0.193	0.001
**10**	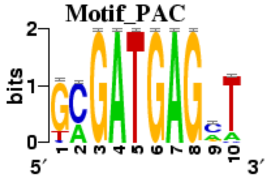	186.58	0.8	0.632	0.083
		155.86	0.65	0.667	0.110

	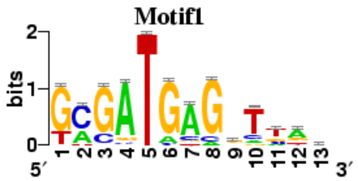				
	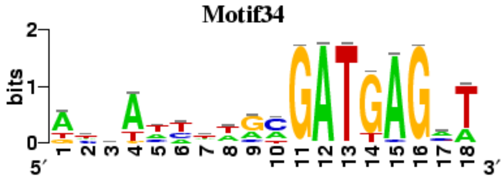	109.30	0.5	0.632	0.133
	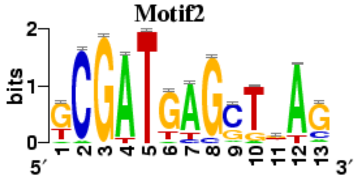	100.53	0.5	0.509	0.093
	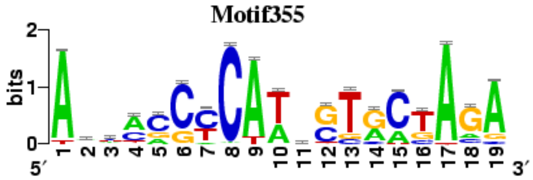	329.36	0.65	0.246	0.003
	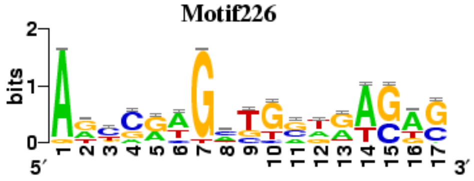	267.61	0.75	0.230	0.004
**11**	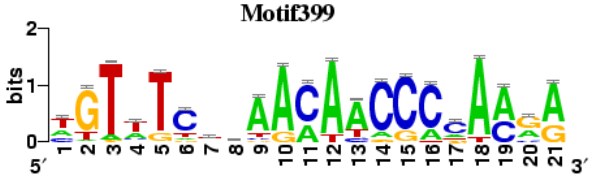	244.37	0.7	0.279	0.009
	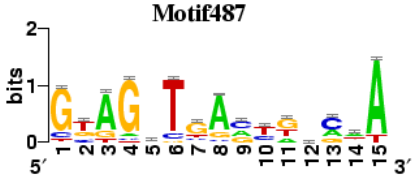	239.76	0.95	0.180	0.002
	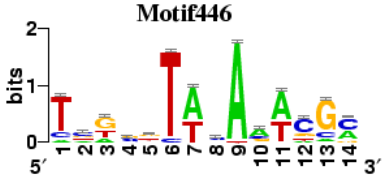	239.76	0.9	0.131	0.000
		316.07	0.75	0.255	0.004

	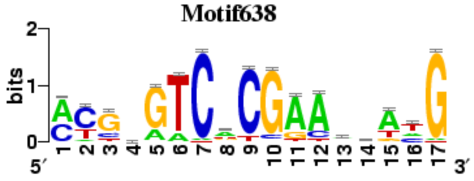				
	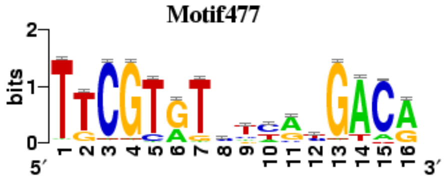	241.90	0.75	0.164	0.001
**12**	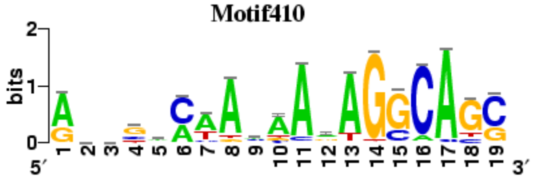	200.12	0.75	0.182	0.003
	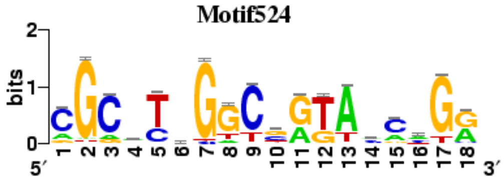	186.59	0.7	0.182	0.004
	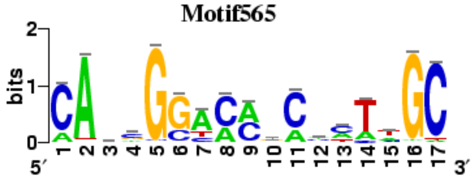	174.66	0.7	0.182	0.004
	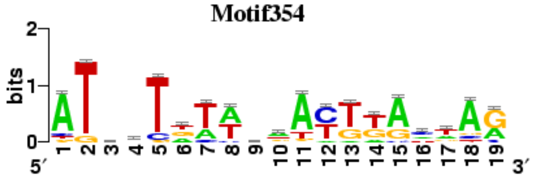	228.43	0.85	0.155	0.001
**13**	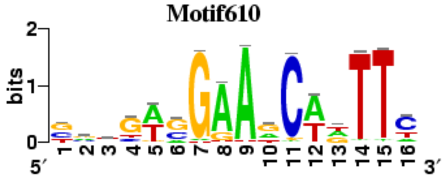	120.37	0.75	0.155	0.005
	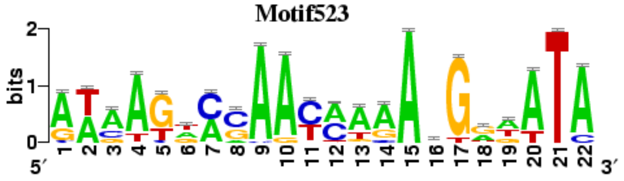	36.04	0.95	0.034	0.000
		19.51	0.5	0.414	0.179

	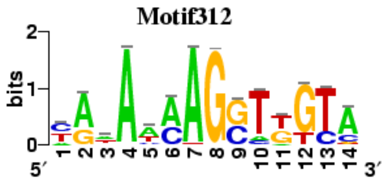				
	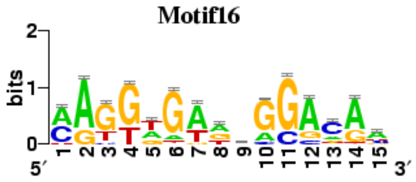	18.73	0.5	0.103	0.371
	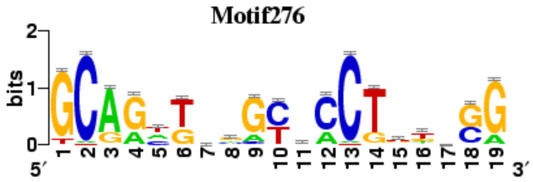	333.96	0.7	0.207	0.001
	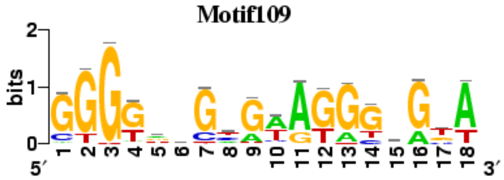	316.08	0.75	0.276	0.005
**14**	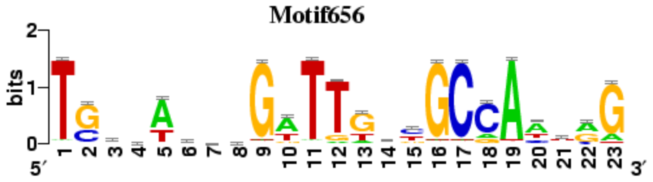	291.35	0.6	0.172	0.001
	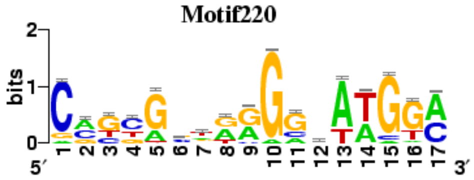	279.44	0.8	0.276	0.007
	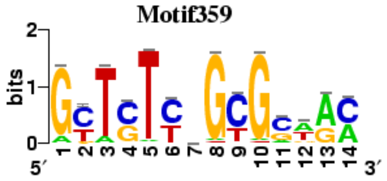	267.62	0.75	0.207	0.003
	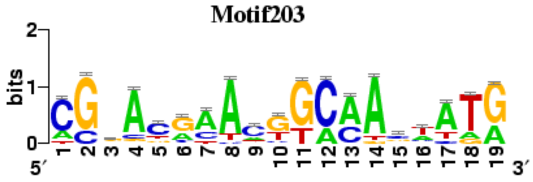	455.92	0.7	0.283	0.002
		401.39	0.65	0.340	0.006

15	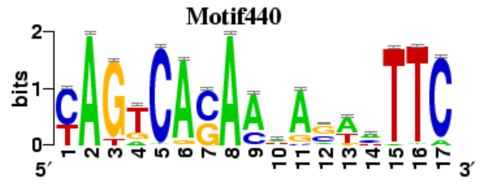				
	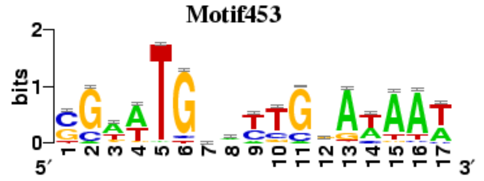	358.38	0.85	0.189	0.000
	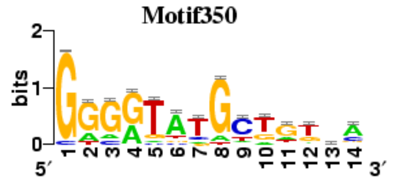	353.58	0.75	0.245	0.002
	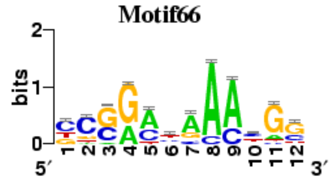	344.01	0.8	0.453	0.018
16	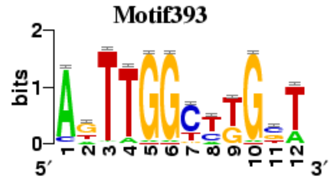	293.71	0.85	0.232	0.003
	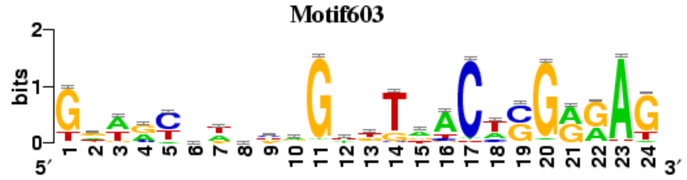	273.51	0.75	0.179	0.001
	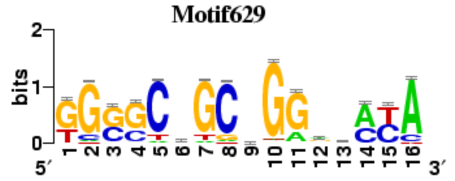	214.33	0.8	0.161	0.002
	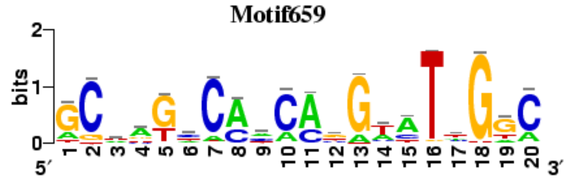	196.18	0.6	0.179	0.003
		151.45	0.75	0.179	0.005

	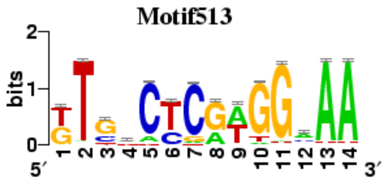				
17	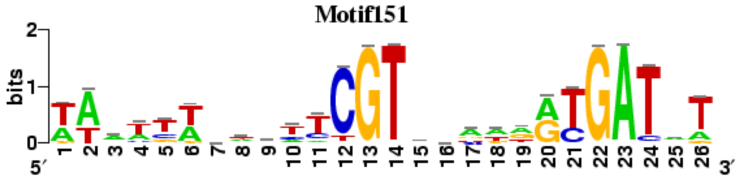	171.86	0.7	0.358	0.025
	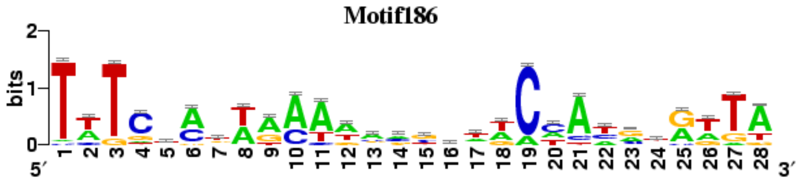	94.21	0.5	0.547	0.107
	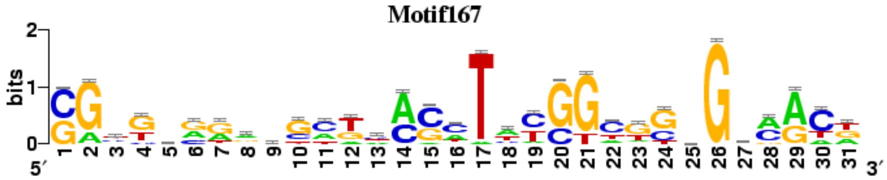	79.37	0.9	0.057	0.000
	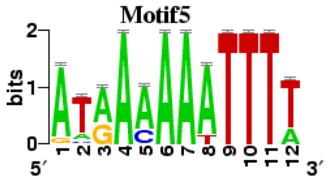	65.49	0.6	0.566	0.149
	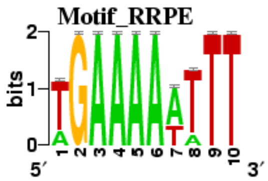	57.78	0.8	0.585	0.172
18	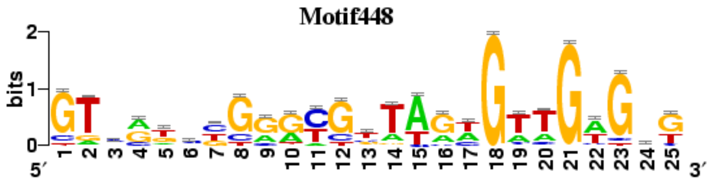	398.49	0.75	0.340	0.005
	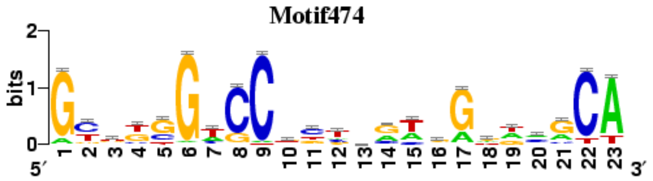	286.62	0.8	0.191	0.001
		230.84	0.7	0.149	0.001

	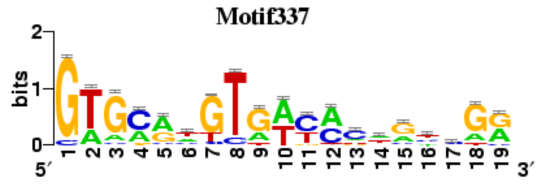				
	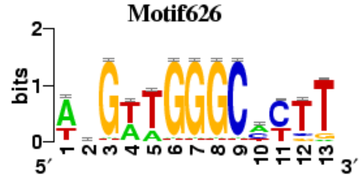	230.84	0.9	0.149	0.001
	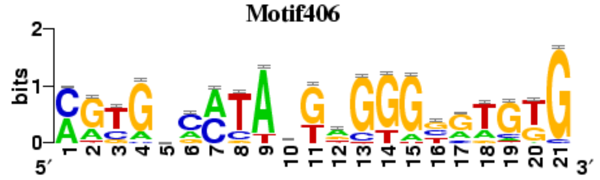	217.12	0.7	0.191	0.003
	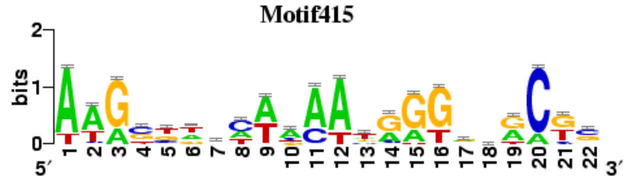	308.45	0.85	0.250	0.003
	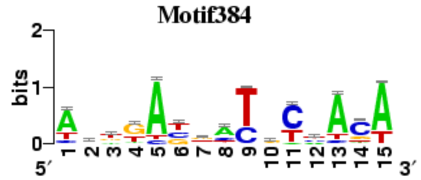	259.29	0.8	0.250	0.005
**19**	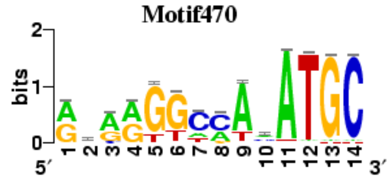	220.86	0.9	0.125	0.000
	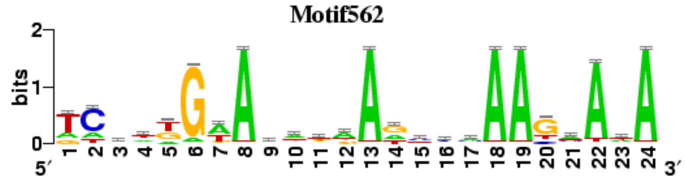	196.06	0.8	0.146	0.001
	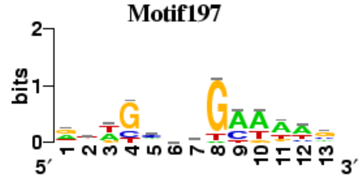	132.39	0.95	0.083	0.000
		435.33	0.7	0.340	0.004

20	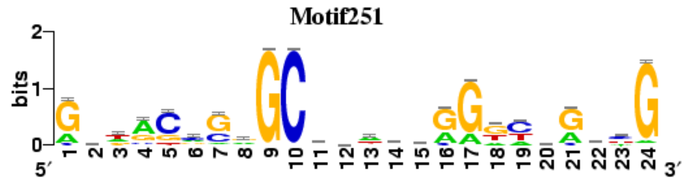				
	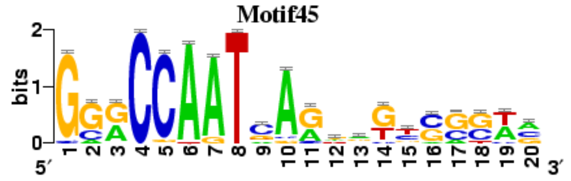	217.70	0.65	0.170	0.002
	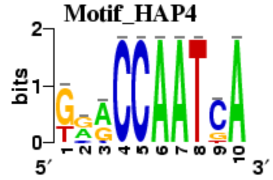	181.76	0.8	0.298	0.013
	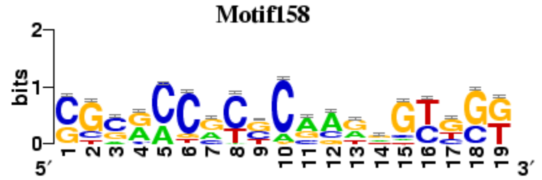	175.41	0.55	0.468	0.039
	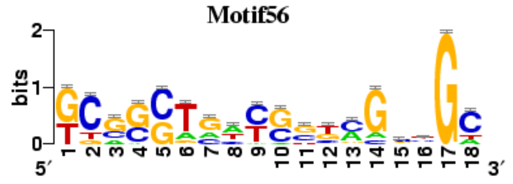	172.38	0.65	0.426	0.033
21	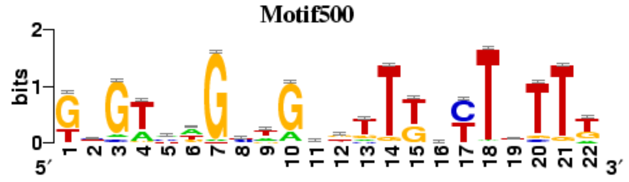	186.06	0.65	0.326	0.016
	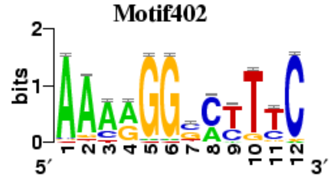	157.24	0.75	0.261	0.011
	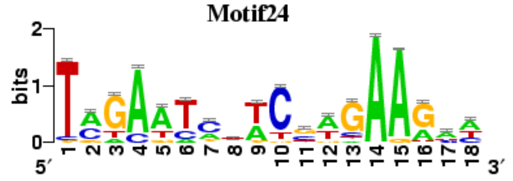	46.14	0.9	0.043	0.000
		46.14	0.95	0.043	0.000

	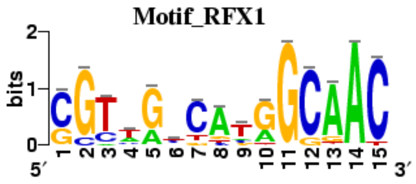				
	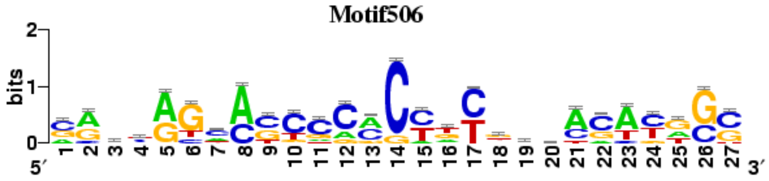	44.22	0.85	0.065	0.001
	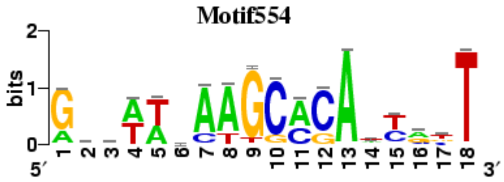	234.75	0.7	0.273	0.007
	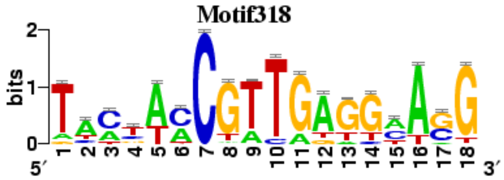	151.82	0.5	0.318	0.018
22	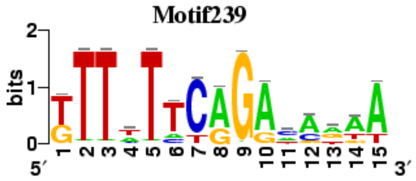	141.82	0.7	0.318	0.020
	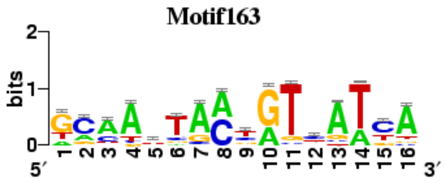	106.89	0.75	0.295	0.023
	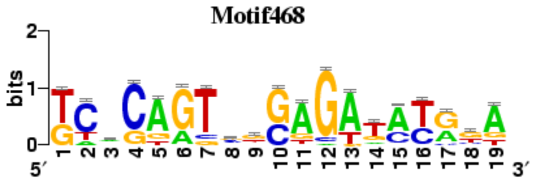	48.38	0.95	0.045	0.000
	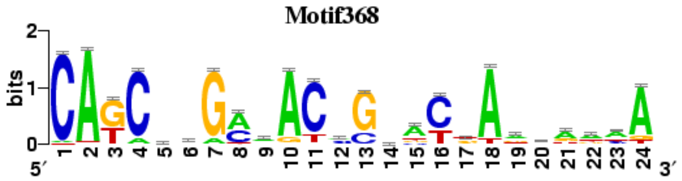	281.79	0.8	0.174	0.001
		178.72	0.75	0.217	0.006

23	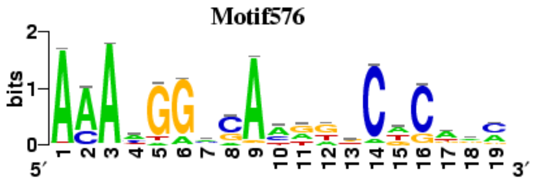				
	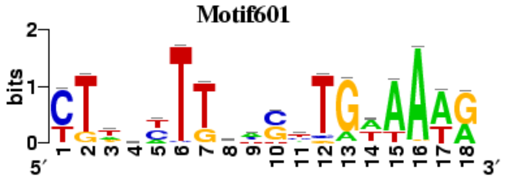	138.21	0.8	0.261	0.013
	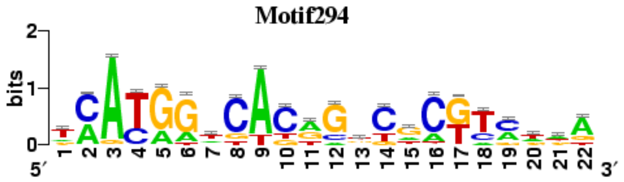	123.25	0.5	0.304	0.022
	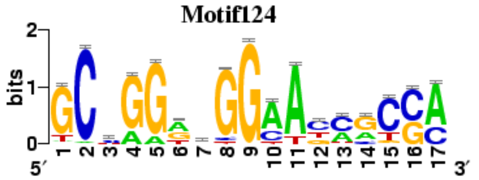	46.14	0.95	0.043	0.000
24	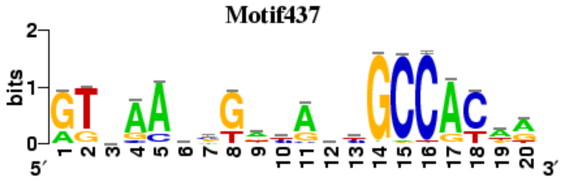	412.07	0.8	0.256	0.001
	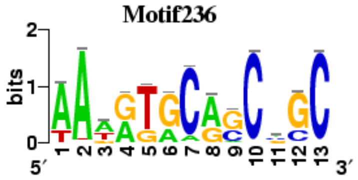	398.02	0.9	0.209	0.000
	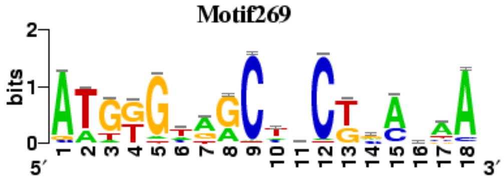	396.82	0.7	0.279	0.002
	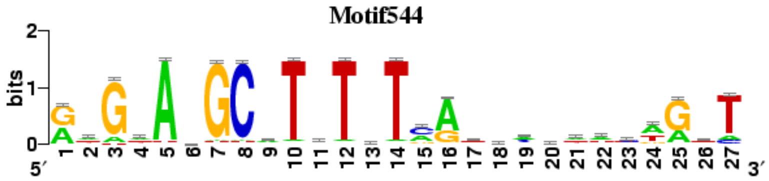	379.01	0.75	0.256	0.002
		333.01	0.8	0.302	0.005

	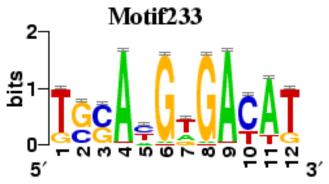				
25	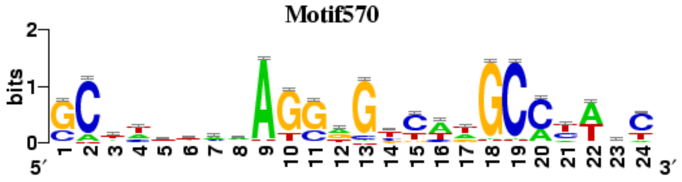	372.97	0.65	0.238	0.001
	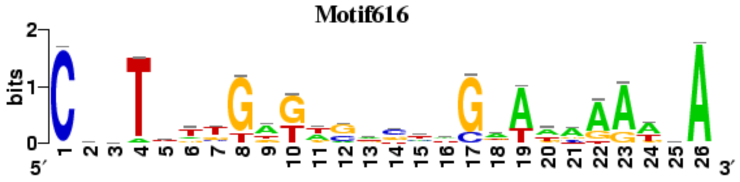	312.61	0.8	0.262	0.003
	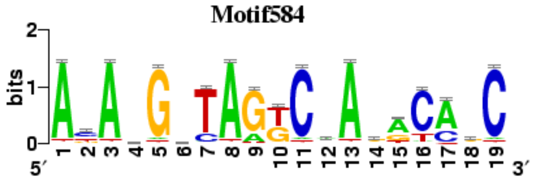	305.84	0.7	0.167	0.000
	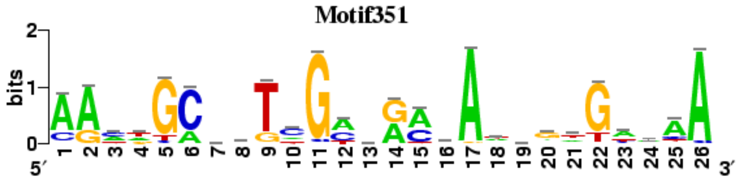	293.20	0.7	0.262	0.004
	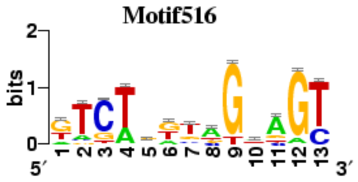	246.05	0.85	0.190	0.002
26	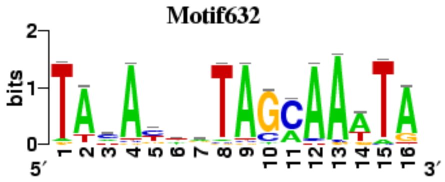	115.41	0.85	0.156	0.004
	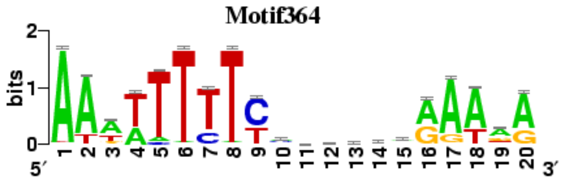	108.94	0.75	0.356	0.034
		86.92	0.7	0.378	0.048

	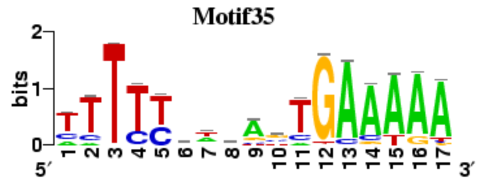				
	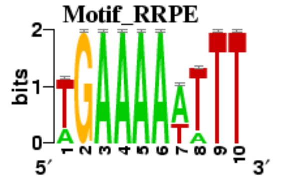	74.70	0.85	0.644	0.156
	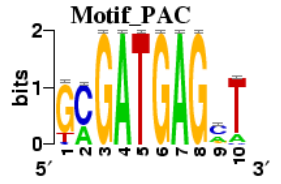	71.03	0.75	0.511	0.103
27	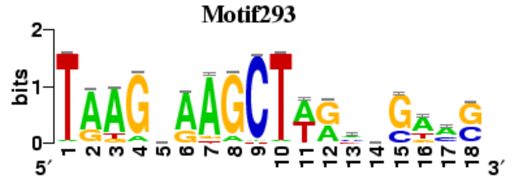	275.25	0.65	0.227	0.003
	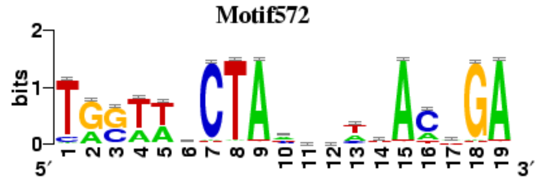	262.11	0.75	0.250	0.004
	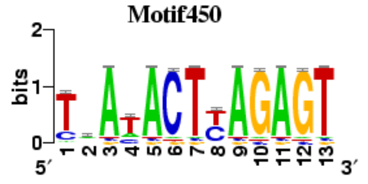	215.45	0.8	0.159	0.001
	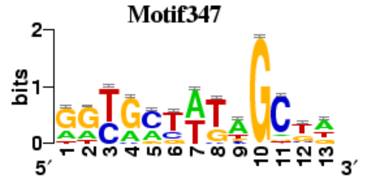	170.14	0.8	0.159	0.002
	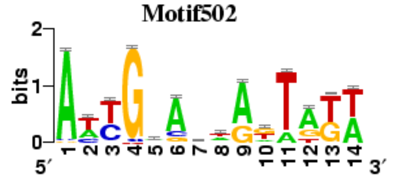	145.28	0.95	0.091	0.000
		427.89	0.85	0.488	0.013

**28**	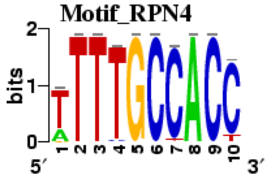				
	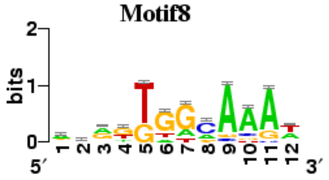	303.06	0.85	0.465	0.019
	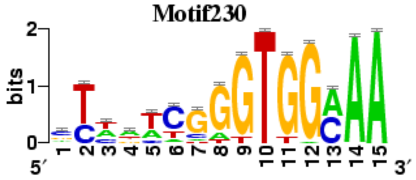	295.32	0.55	0.558	0.030
	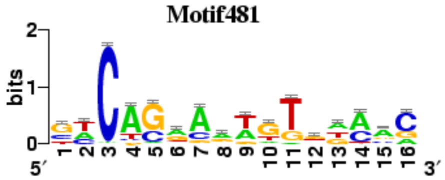	260.53	0.85	0.209	0.002
	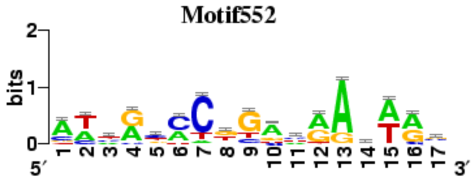	220.89	0.9	0.163	0.001
**29**	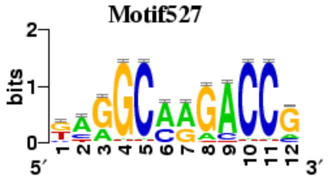	323.65	0.8	0.214	0.001
	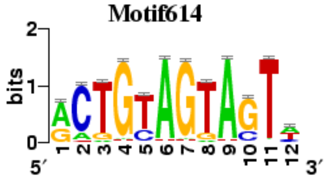	133.27	0.85	0.119	0.001
	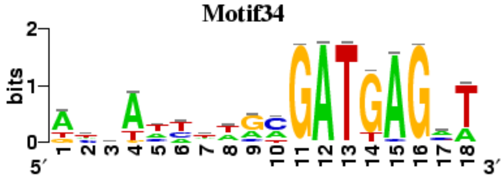	88.19	0.65	0.452	0.064
		69.29	0.85	0.119	0.004

	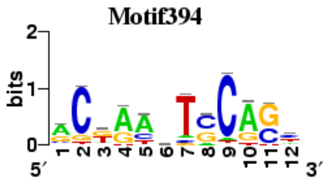				
	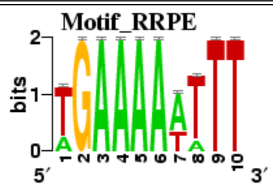	28.15	0.85	0.476	0.160
**30**	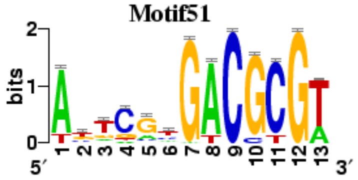	632.49	0.6	0.537	0.008
	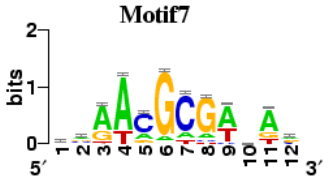	616.35	0.9	0.610	0.012
	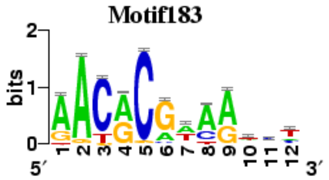	475.69	0.8	0.756	0.032
	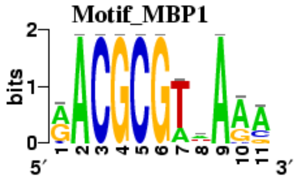	378.38	0.8	0.488	0.015
	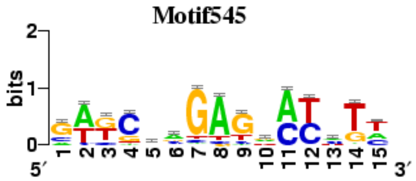	366.31	0.85	0.195	0.000
	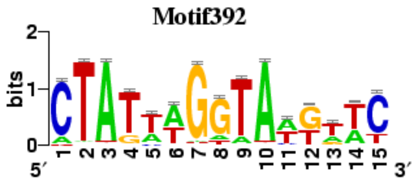	322.25	0.85	0.175	0.000
		308.87	0.75	0.225	0.002

31	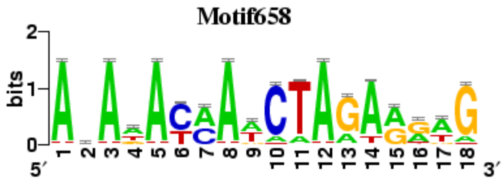				
	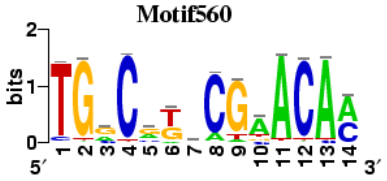	274.63	0.85	0.175	0.001
	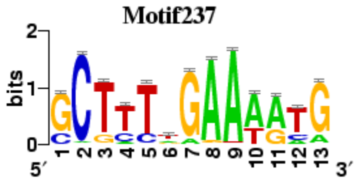	268.41	0.9	0.150	0.000
	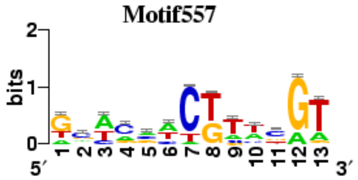	214.62	0.85	0.125	0.000
32	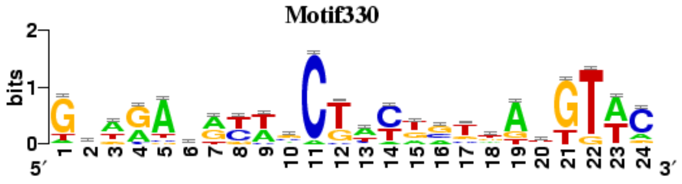	542.09	0.85	0.278	0.000
	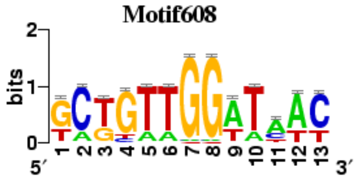	338.58	0.8	0.333	0.005
	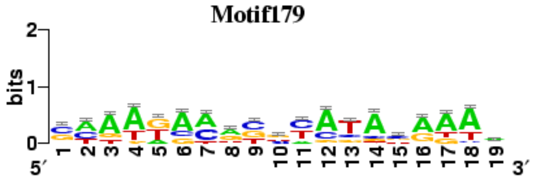	263.47	0.85	0.222	0.002
	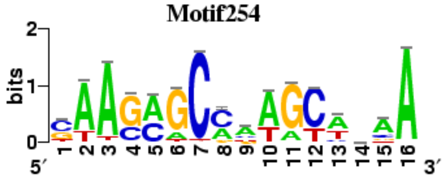	252.36	0.7	0.306	0.007
		189.88	0.8	0.222	0.004

	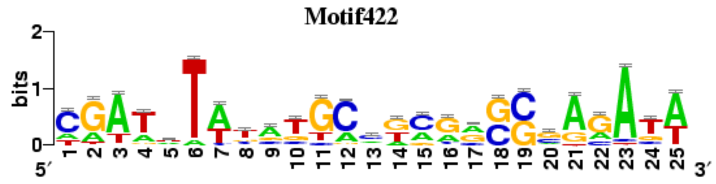				
33	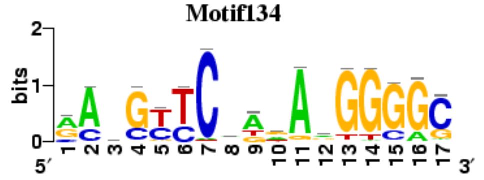	407.37	0.7	0.333	0.003
	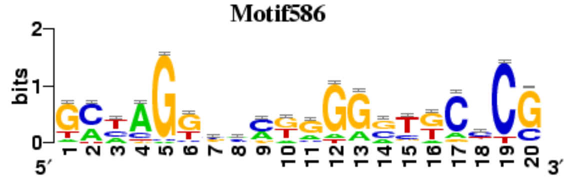	403.27	0.75	0.242	0.001
	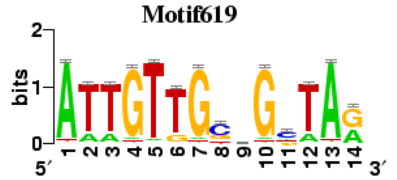	403.27	0.8	0.242	0.001
	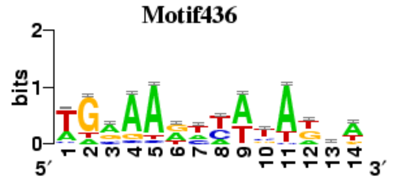	227.38	0.9	0.273	0.005
	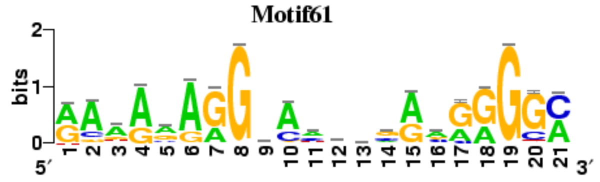	219.02	0.75	0.394	0.015
34	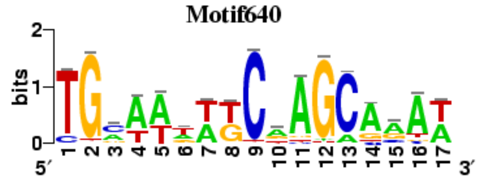	576.53	0.85	0.294	0.000
	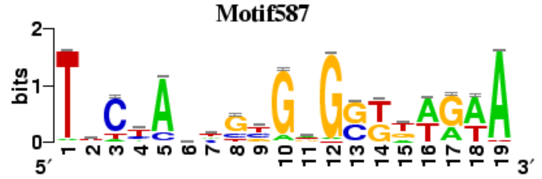	360.75	0.7	0.353	0.005
		345.65	0.8	0.235	0.001

	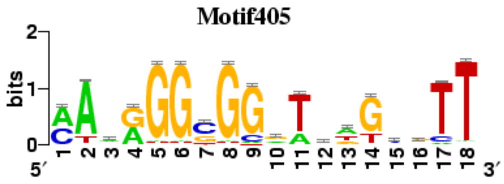				
	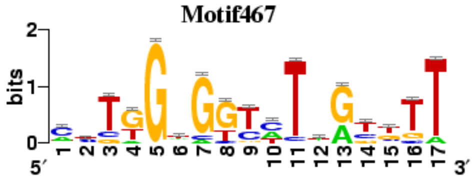	339.71	0.95	0.294	0.003
	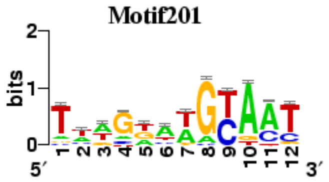	233.64	0.95	0.265	0.005
	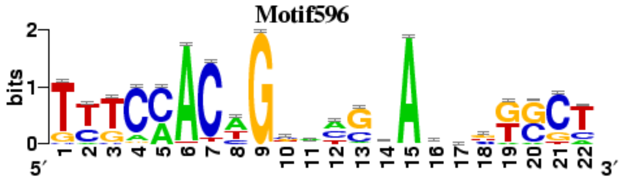	370.03	0.65	0.324	0.004
	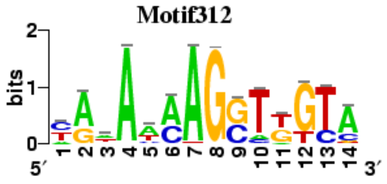	343.53	0.8	0.353	0.006
**35**	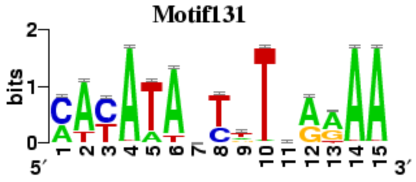	326.63	0.8	0.412	0.010
	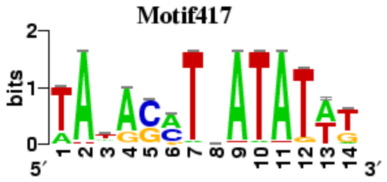	243.61	0.8	0.382	0.012
	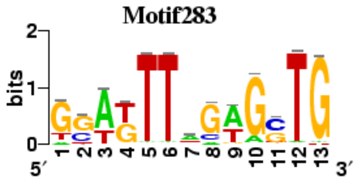	238.25	0.75	0.294	0.006
		285.21	0.8	0.281	0.004

36	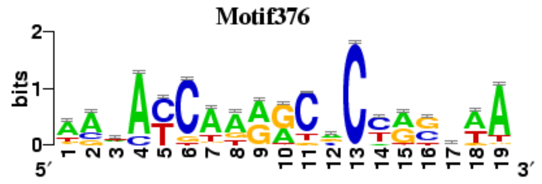				
	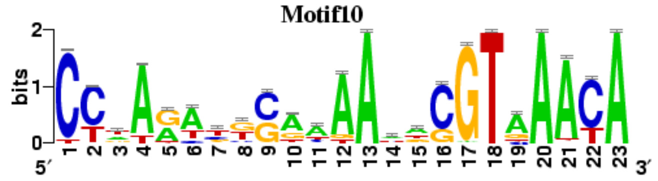	68.13	0.9	0.062	0.000
	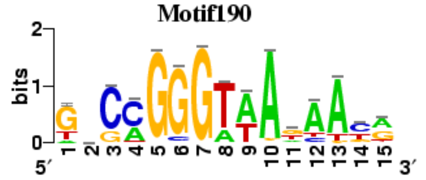	68.13	0.95	0.062	0.000
	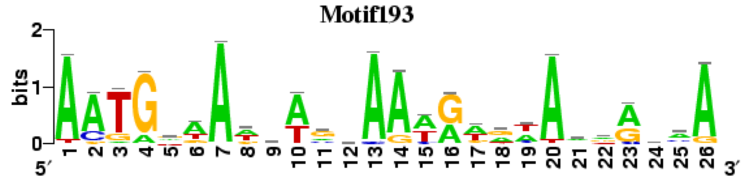	68.13	0.95	0.062	0.000
	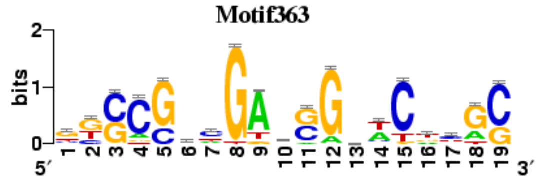	68.13	0.95	0.062	0.000
37	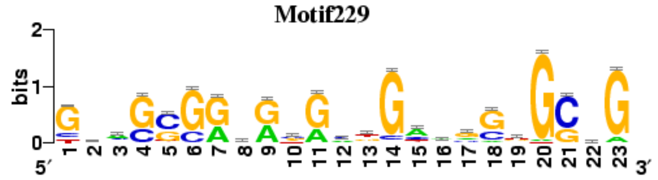	551.66	0.7	0.370	0.002
	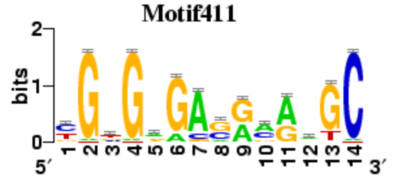	501.95	0.85	0.296	0.001
	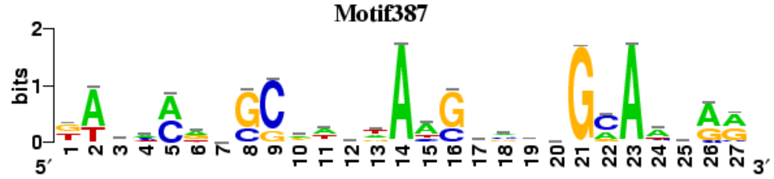	477.60	0.8	0.407	0.004
		367.06	0.8	0.259	0.001

	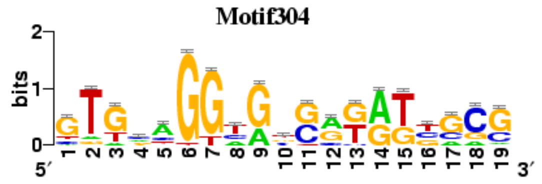				
	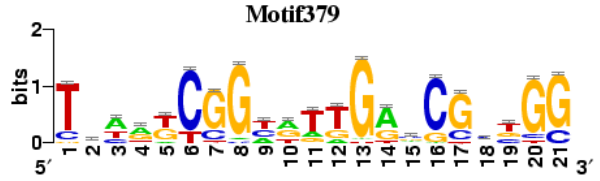	361.76	0.85	0.296	0.002
	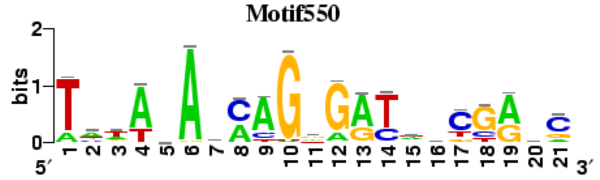	617.43	0.85	0.444	0.003
	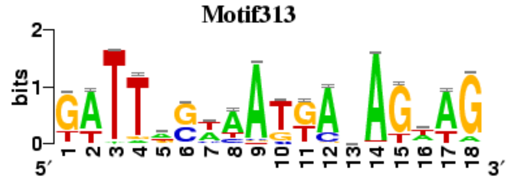	575.38	0.8	0.296	0.000
**38**	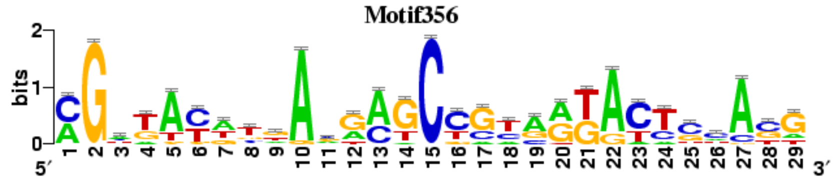	568.96	0.5	0.593	0.009
	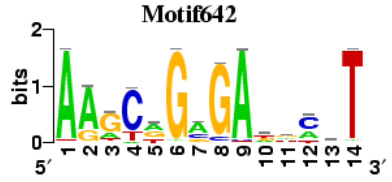	508.45	0.8	0.407	0.003
	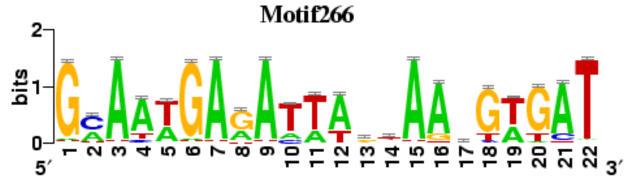	492.95	0.6	0.259	0.000
	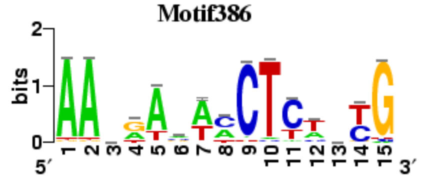	253.58	0.8	0.250	0.003
		152.75	0.75	0.286	0.009

39	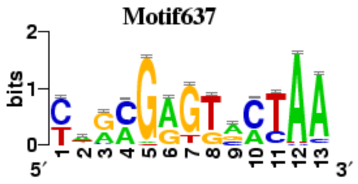			
	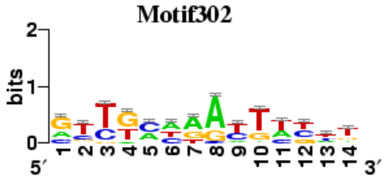	26.41	0.5	0.500	0.858
	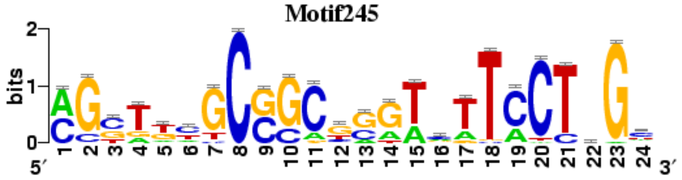	24.96	0.85	0.071	0.001
	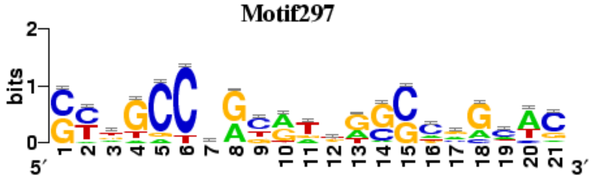	24.96	0.95	0.071	0.001
40	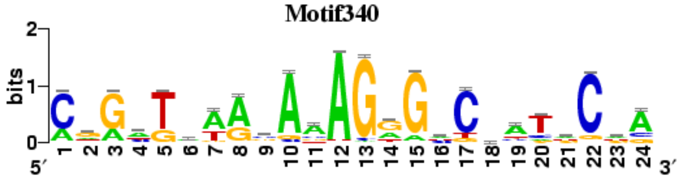	812.77	0.7	0.440	0.001
	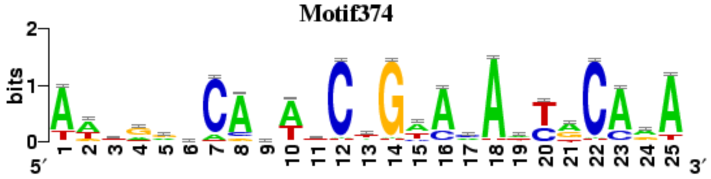	805.67	0.75	0.400	0.000
	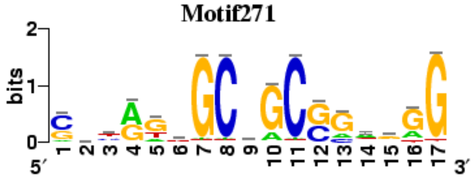	484.26	0.85	0.320	0.001
	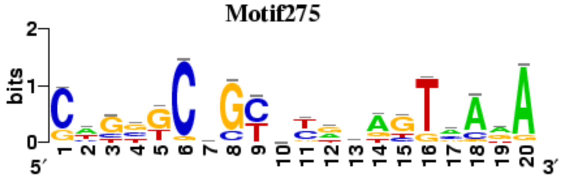	377.02	0.7	0.480	0.009
		317.62	0.85	0.280	0.002

	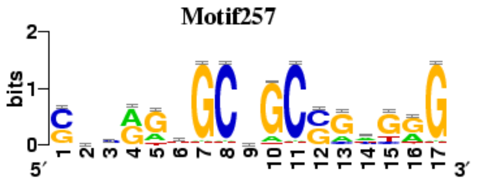				
41	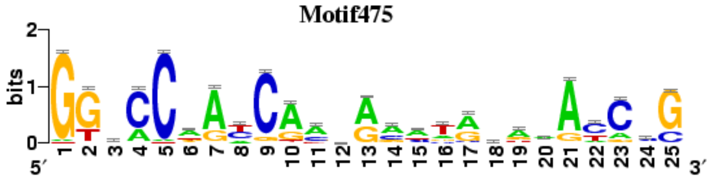	850.21	0.75	0.458	0.001
	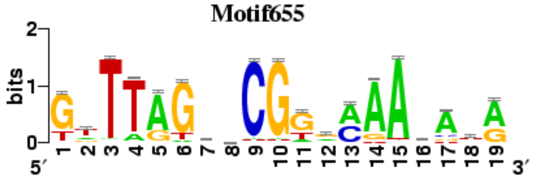	596.34	0.7	0.375	0.001
	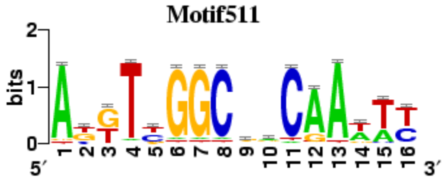	418.14	0.8	0.292	0.001
	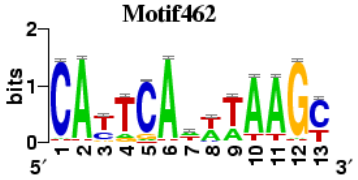	321.50	0.85	0.333	0.004
	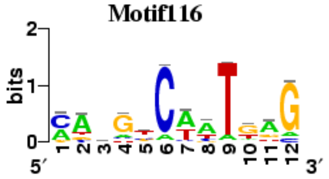	297.45	0.85	0.208	0.001
42	NA	NA	NA	NA	NA
43	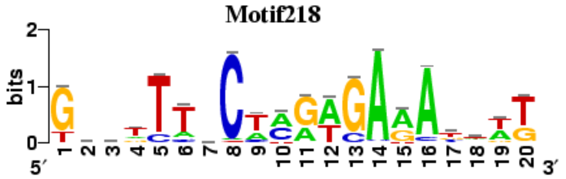	692.06	0.75	0.588	0.004
	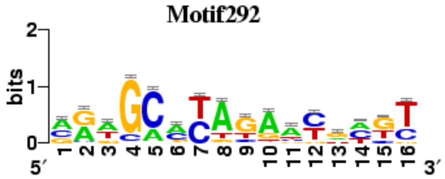	571.66	0.8	0.353	0.001
		314.05	0.75	0.471	0.007

	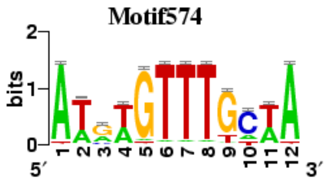				
	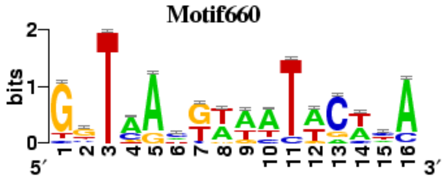	269.25	0.7	0.588	0.016
	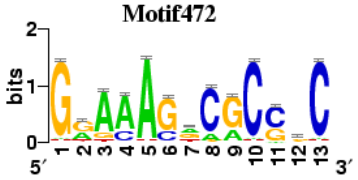	215.79	0.8	0.294	0.003
	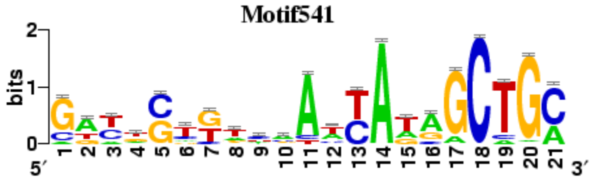	1637.52	0.85	0.812	0.001
	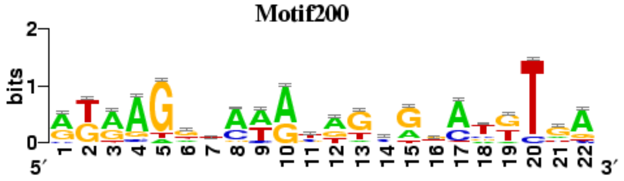	1342.32	0.85	0.688	0.001
**44**	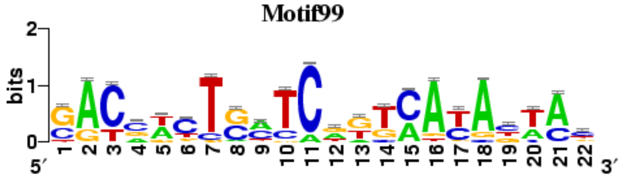	1195.25	0.8	0.625	0.001
	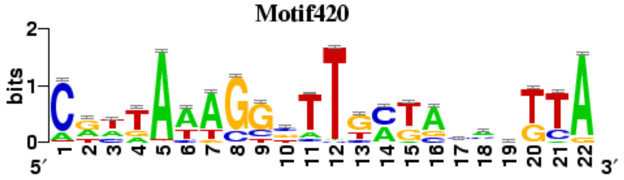	1085.47	0.8	0.625	0.001
	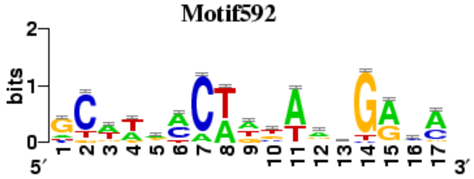	1085.47	0.9	0.625	0.001
		1146.64	0.7	0.647	0.001

45	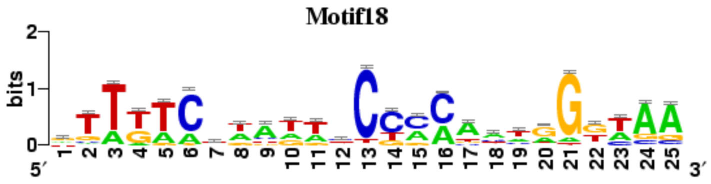				
	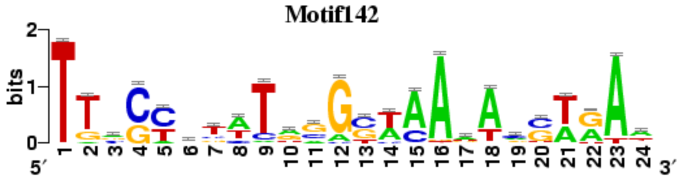	1101.85	0.8	0.529	0.000
	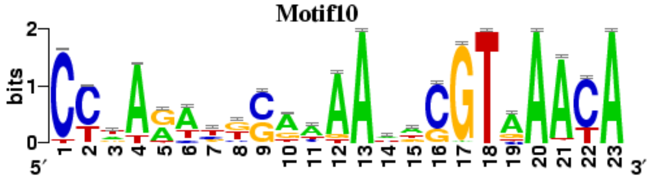	976.80	0.5	0.706	0.003
	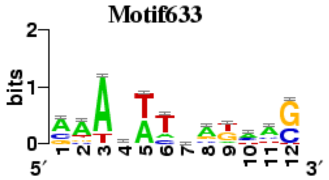	687.66	0.95	0.353	0.000
	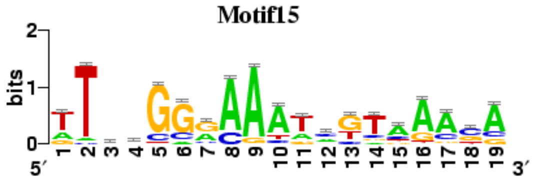	609.32	0.85	0.471	0.002
46	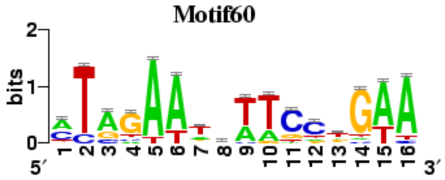	643.42	0.8	0.643	0.005
	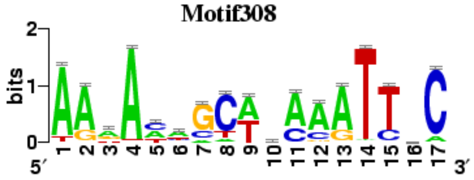	643.42	0.8	0.643	0.005
	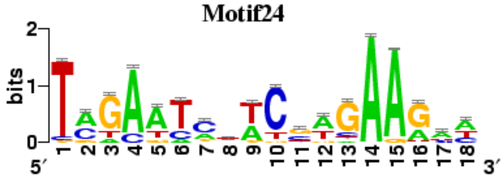	474.71	0.7	0.429	0.002
		386.96	0.85	0.429	0.003

	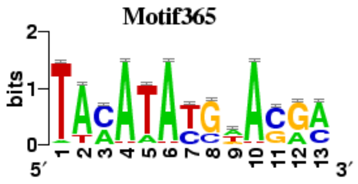				
	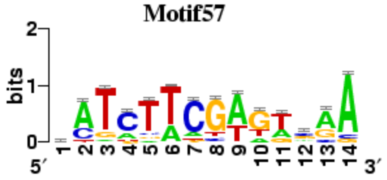	302.34	0.85	0.429	0.005
	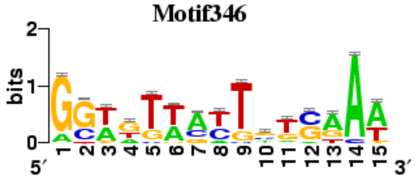	638.06	0.8	0.667	0.006
	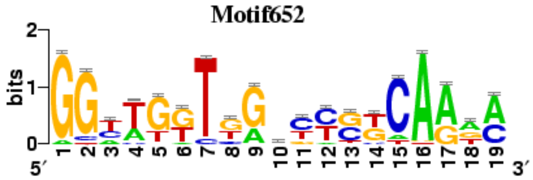	594.56	0.75	0.533	0.003
47	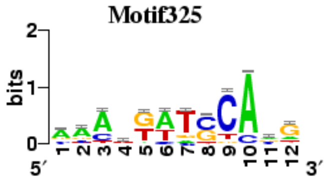	568.41	0.95	0.467	0.002
	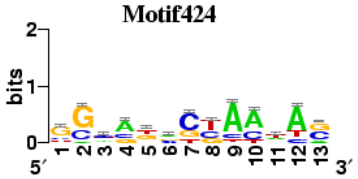	349.23	0.85	0.667	0.014
	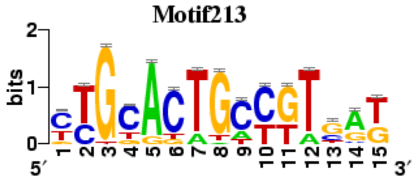	156.77	0.8	0.200	0.001
	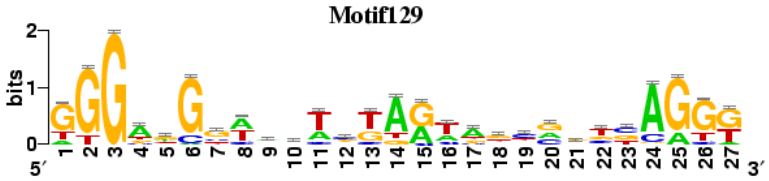	458.66	0.95	0.375	0.001
		390.66	0.85	0.625	0.005

48	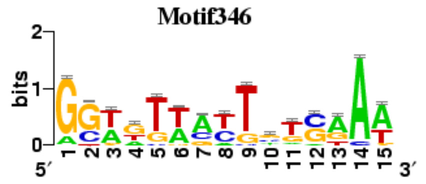				
	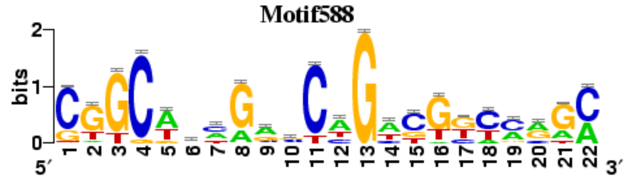	344.67	0.95	0.250	0.000
	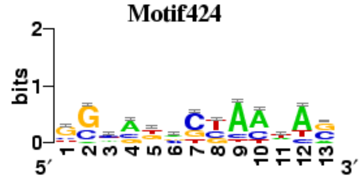	235.38	0.9	0.500	0.005
	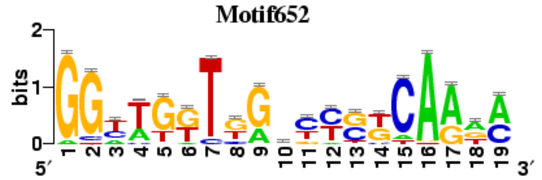	235.38	0.75	0.500	0.005
49	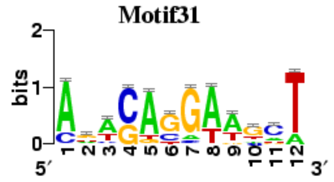	922.13	0.9	0.600	0.001
	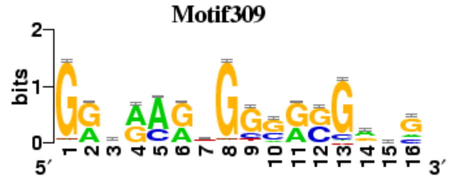	580.40	0.95	0.400	0.001
	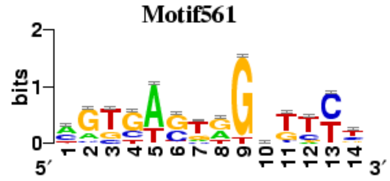	516.75	0.9	0.300	0.000
	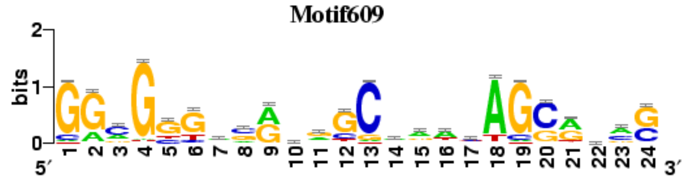	516.75	0.95	0.300	0.000
		438.41	0.65	0.700	0.008

	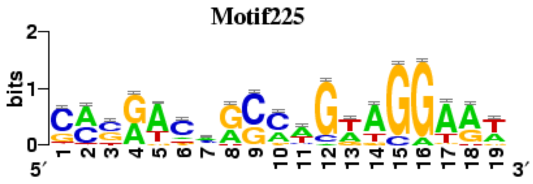				

CV05:

Cluster	Motif	Chi-square statistic	Score cutoff	Posterior probability within cluster	Posterior probability outside cluster
1	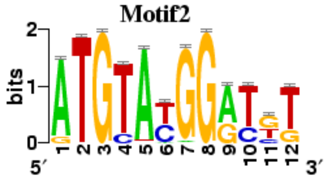	802.52	0.55	0.782	0.036
	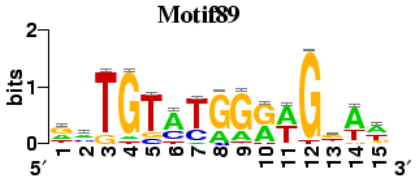	471.39	0.65	0.594	0.040
	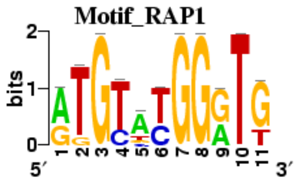	454.52	0.7	0.644	0.052
	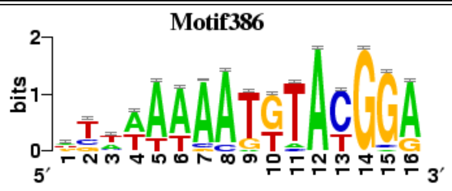	275.92	0.55	0.317	0.017
	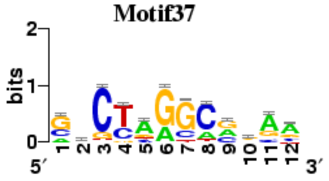	207.09	0.85	0.228	0.011
	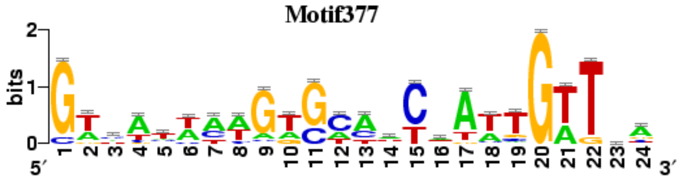	247.47	0.8	0.161	0.002
	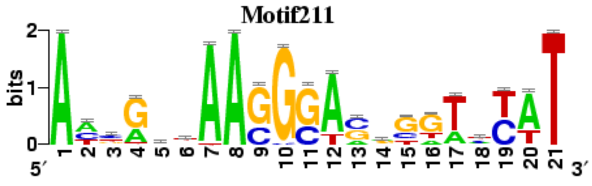	241.55	0.55	0.290	0.016

2	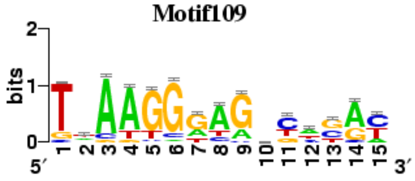	227.79	0.75	0.226	0.009
	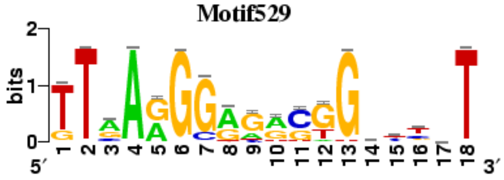	226.46	0.7	0.172	0.004
	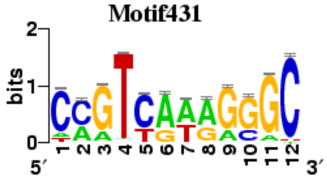	196.28	0.8	0.108	0.001
3	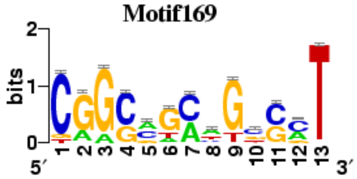	208.28	0.7	0.193	0.006
	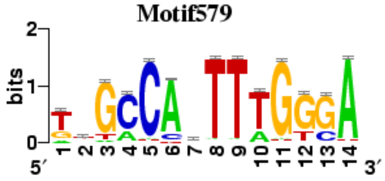	162.74	0.8	0.102	0.001
	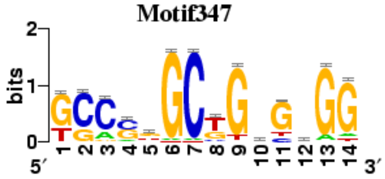	159.95	0.75	0.125	0.003
	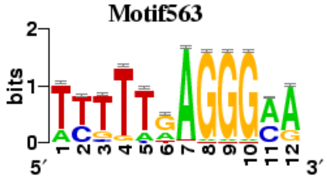	141.57	0.8	0.148	0.006
	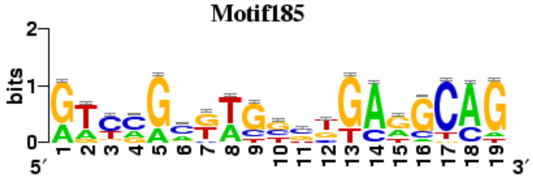	121.97	0.75	0.148	0.007
		416.09	0.55	0.814	0.089

4	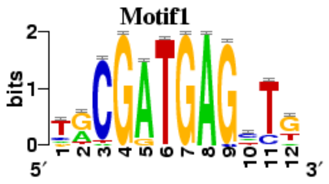				
	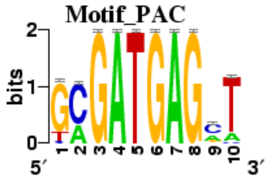	367.01	0.75	0.756	0.086
	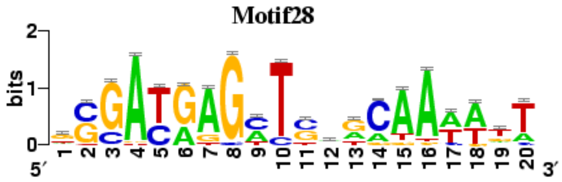	283.09	0.5	0.453	0.036
	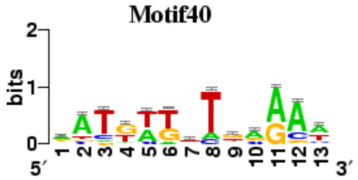	185.00	0.75	0.651	0.123
	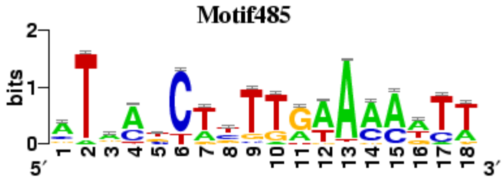	164.17	0.75	0.163	0.006
5	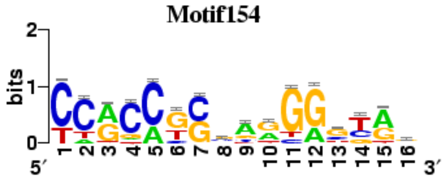	311.11	0.75	0.261	0.005
	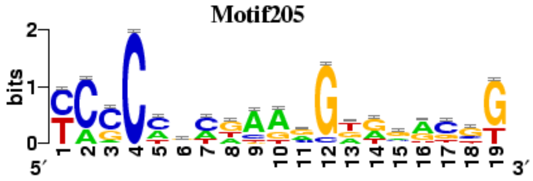	214.52	0.6	0.377	0.027
	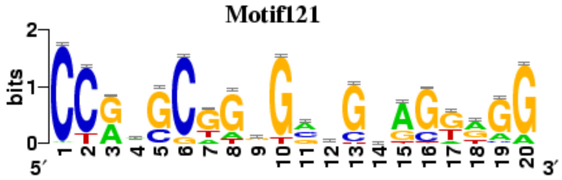	193.80	0.7	0.159	0.003
		182.63	0.85	0.174	0.004

	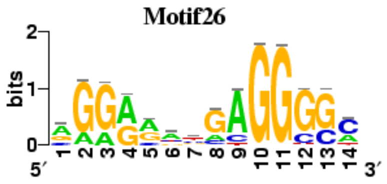				
	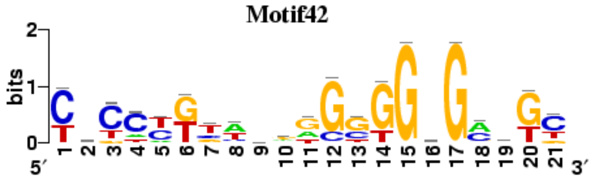	172.82	0.6	0.290	0.019
	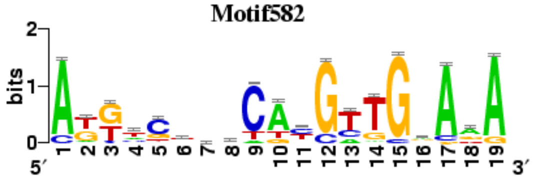	217.71	0.85	0.145	0.001
	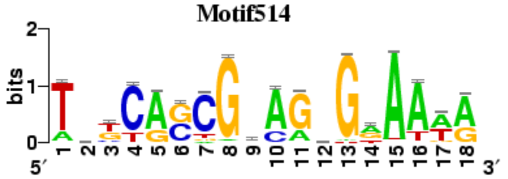	180.54	0.75	0.159	0.003
6	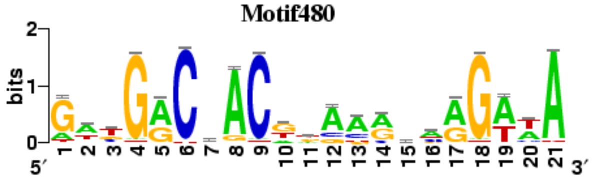	167.47	0.75	0.145	0.003
	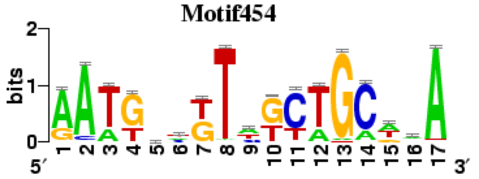	154.60	0.75	0.188	0.007
	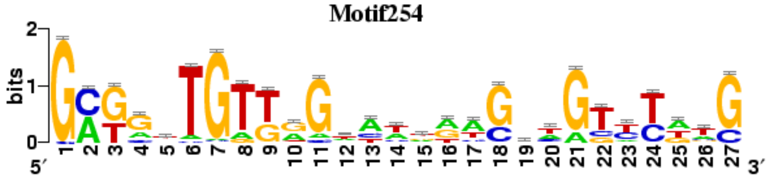	126.58	0.7	0.145	0.005
	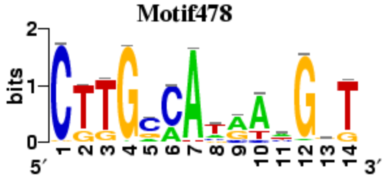	226.76	0.75	0.194	0.004
		177.47	0.55	0.448	0.048

	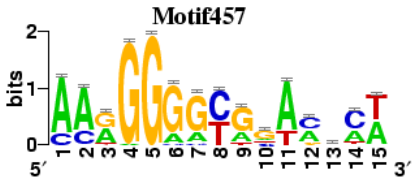				
	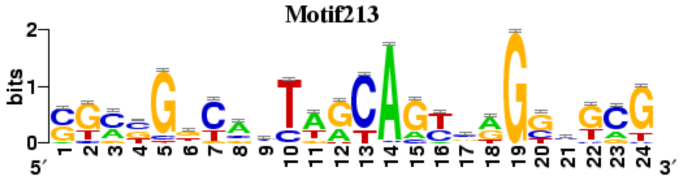	158.66	0.5	0.254	0.015
7	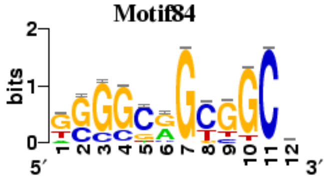	153.69	0.8	0.224	0.011
	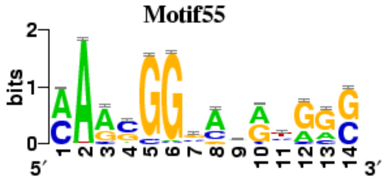	144.60	0.75	0.373	0.040
	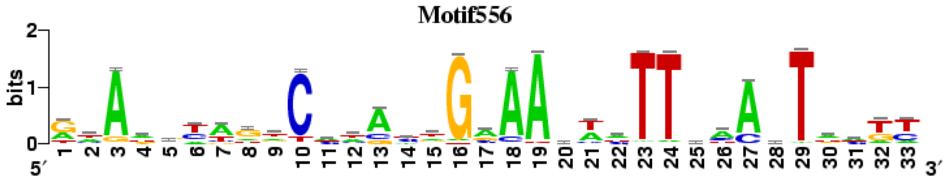	297.83	0.7	0.197	0.002
	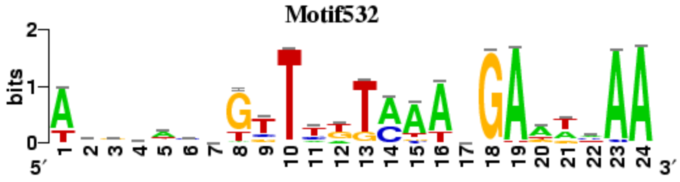	277.85	0.75	0.197	0.002
8	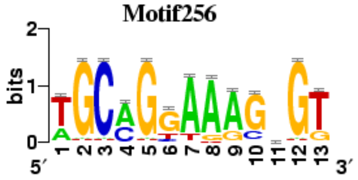	138.39	0.8	0.106	0.001
	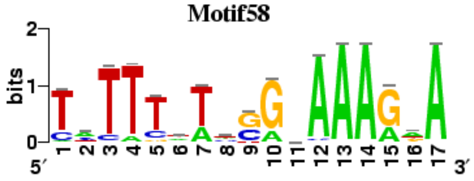	107.70	0.7	0.258	0.024
		94.03	0.9	0.061	0.000

	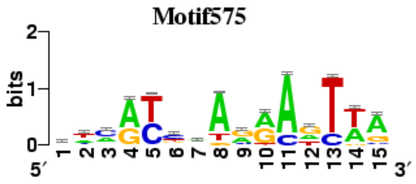				
9	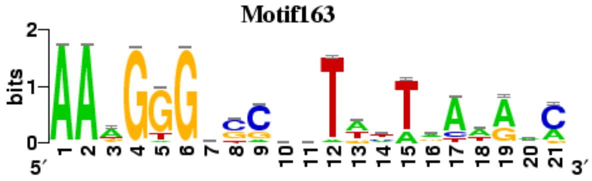	286.99	0.7	0.222	0.003
	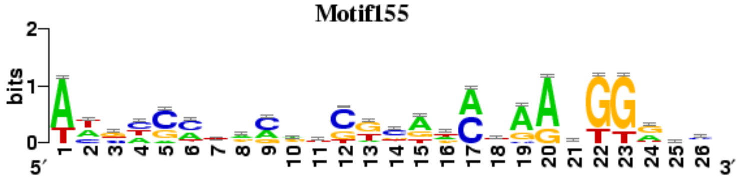	261.34	0.7	0.190	0.002
	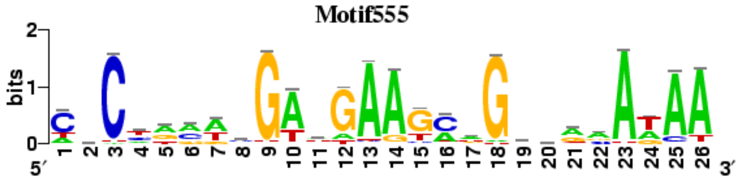	230.78	0.75	0.175	0.002
	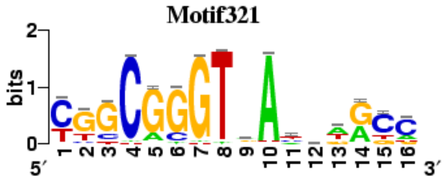	175.44	0.75	0.175	0.004
	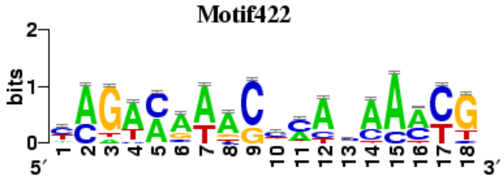	167.98	0.9	0.111	0.001
10	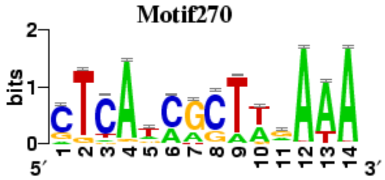	192.31	0.75	0.228	0.007
	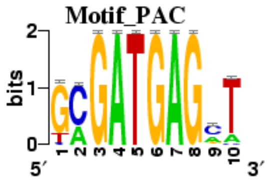	162.56	0.65	0.702	0.118
		132.33	0.9	0.140	0.003

	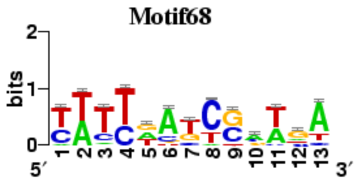				
	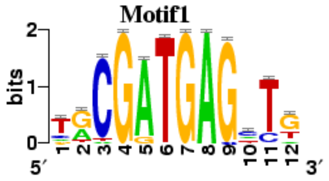	102.02	0.5	0.596	0.125
	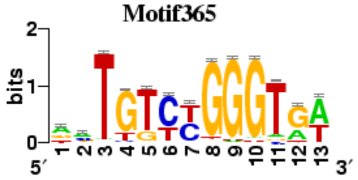	95.18	0.9	0.088	0.001
11	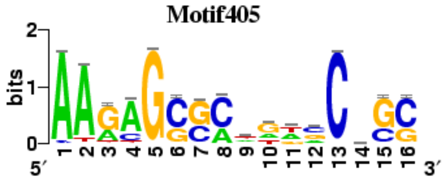	246.91	0.75	0.246	0.006
	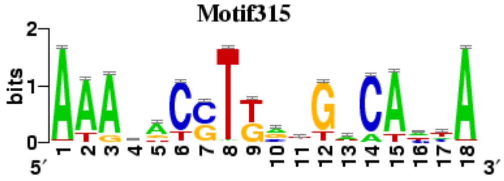	244.02	0.7	0.262	0.008
	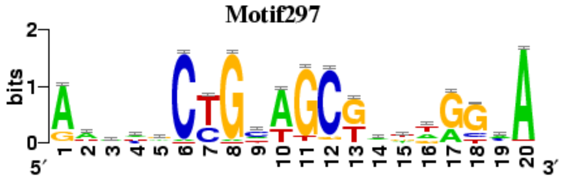	239.04	0.75	0.180	0.002
	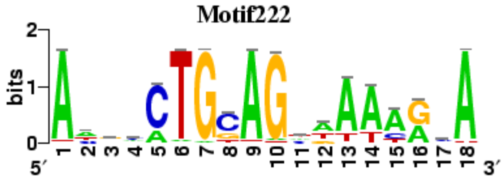	215.67	0.75	0.148	0.001
	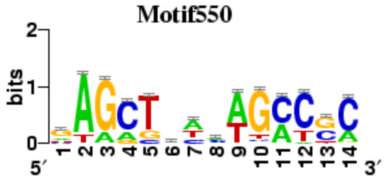	177.25	0.8	0.148	0.002
		237.00	0.75	0.273	0.008

12	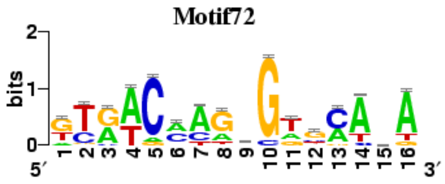				
	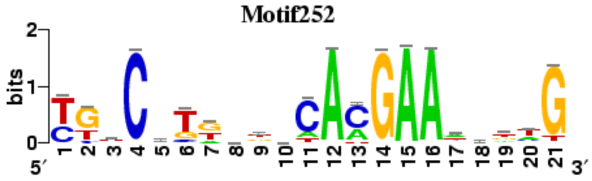	227.53	0.65	0.255	0.007
	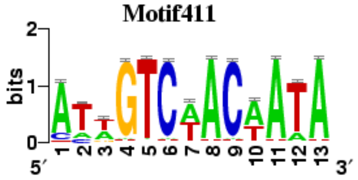	204.68	0.85	0.145	0.001
	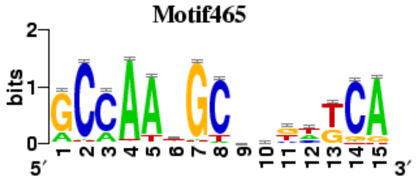	198.52	0.8	0.164	0.002
	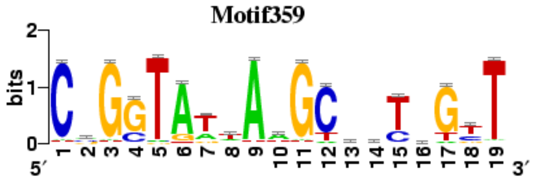	190.46	0.8	0.109	0.000
13	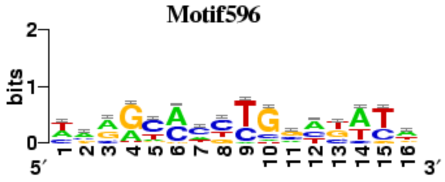	37.47	0.5	0.158	0.562
	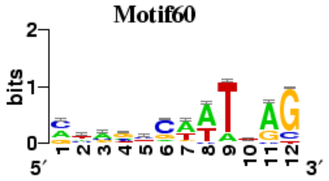	25.09	0.5	0.474	0.766
	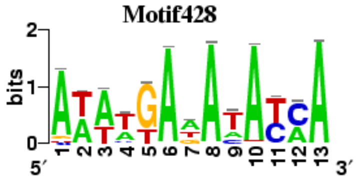	19.17	0.9	0.070	0.006
		18.21	0.85	0.053	0.003

	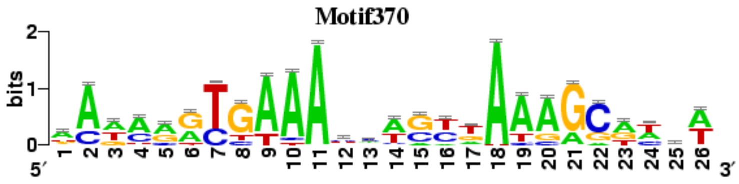				
	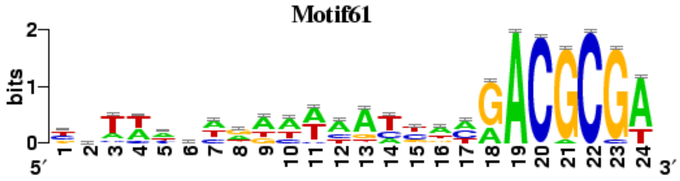	17.32	0.9	0.035	0.001
14	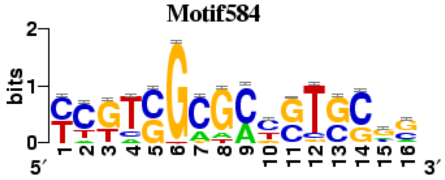	332.52	0.75	0.193	0.001
	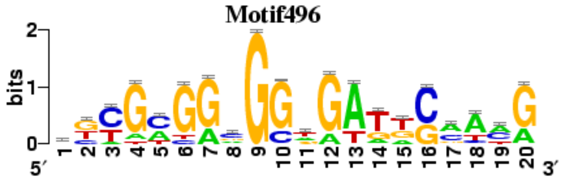	208.42	0.55	0.368	0.022
	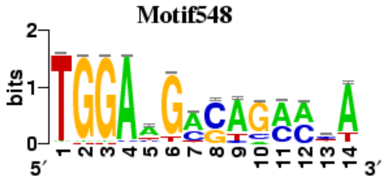	196.15	0.8	0.193	0.004
	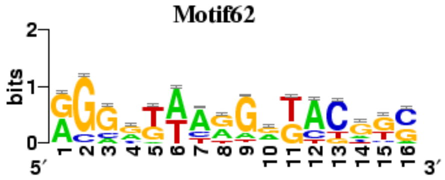	183.35	0.85	0.105	0.000
	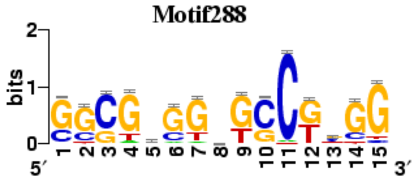	127.02	0.65	0.263	0.018
	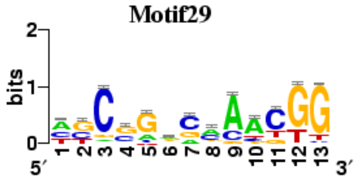	524.17	0.85	0.377	0.004
		454.56	0.9	0.283	0.002

15	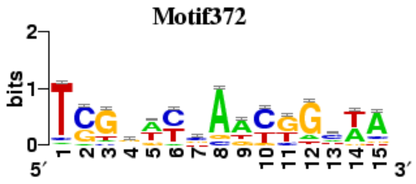				
	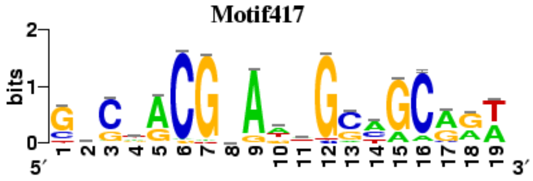	367.15	0.65	0.264	0.003
	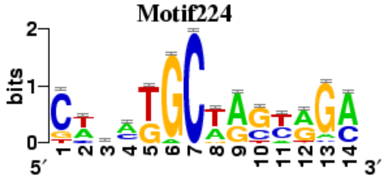	334.60	0.75	0.377	0.011
	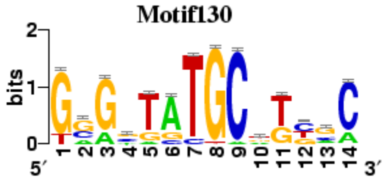	311.45	0.75	0.264	0.004
16	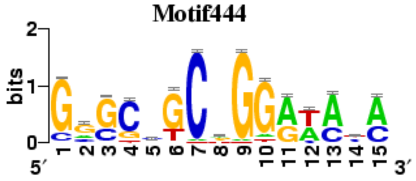	269.62	0.85	0.164	0.001
	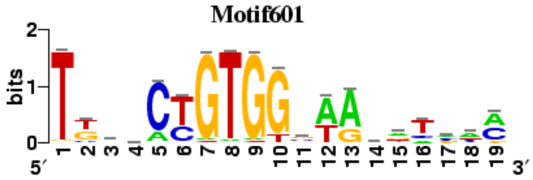	269.62	0.8	0.164	0.001
	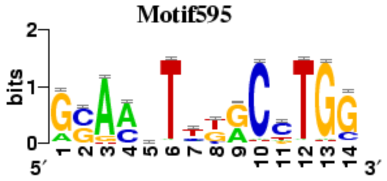	241.18	0.75	0.164	0.001
	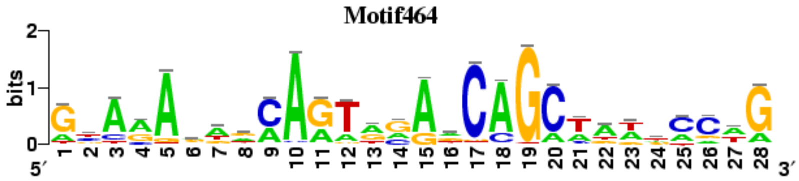	217.91	0.7	0.164	0.002
		214.93	0.75	0.182	0.003

	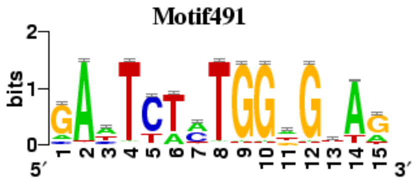				
17	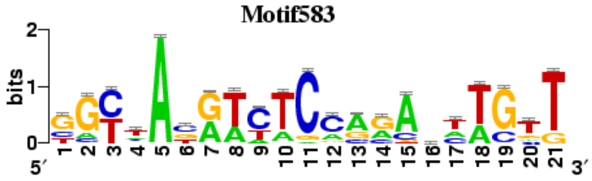	263.55	0.7	0.245	0.005
	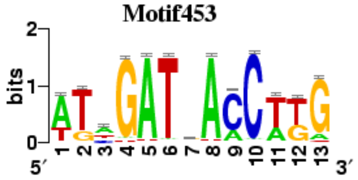	170.48	0.75	0.189	0.005
	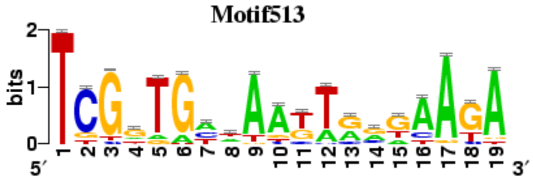	79.14	0.95	0.057	0.000
	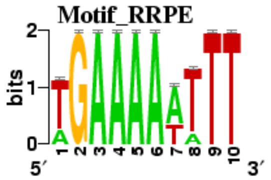	69.93	0.85	0.585	0.152
	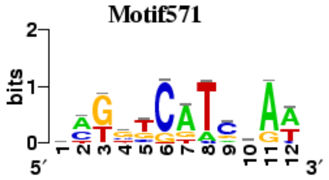	67.13	0.85	0.208	0.020
18	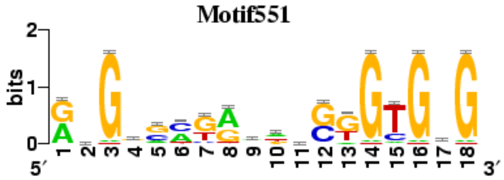	347.26	0.75	0.292	0.004
	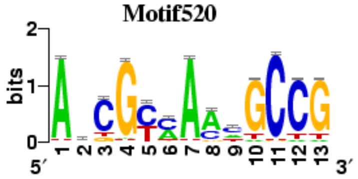	293.78	0.85	0.208	0.002
		264.38	0.9	0.146	0.000

	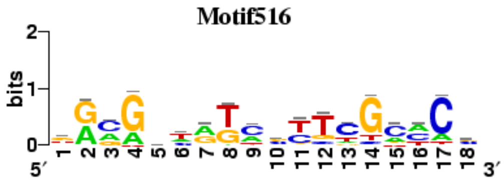 Motif516				
	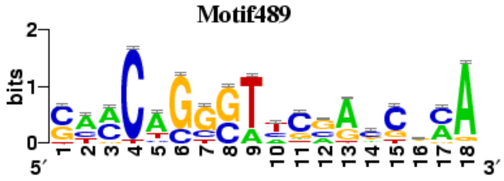 Motif489	237.13	0.75	0.167	0.001
	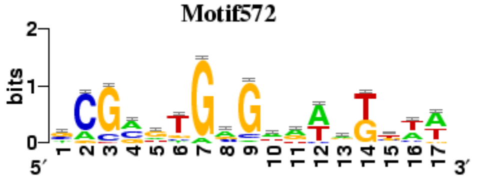 Motif572	225.00	0.9	0.146	0.001
19	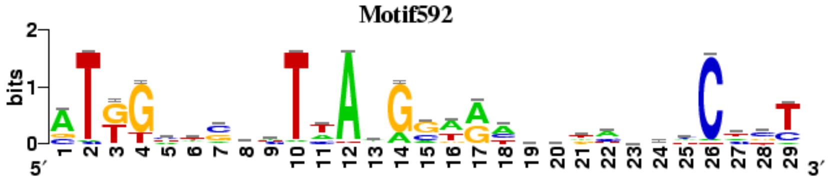 Motif592	441.48	0.75	0.229	0.000
	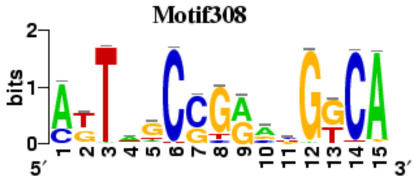 Motif308	335.47	0.75	0.229	0.002
	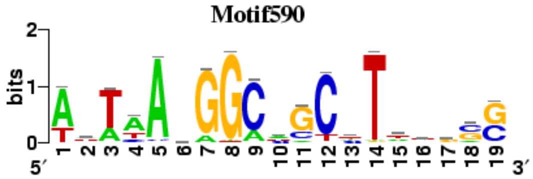 Motif590	310.23	0.8	0.229	0.002
	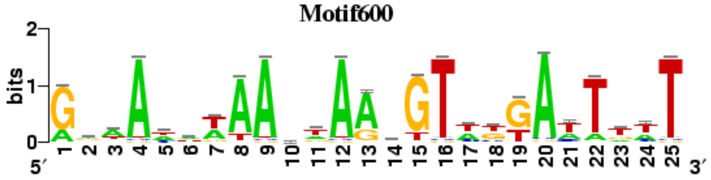 Motif600	252.62	0.75	0.188	0.002
	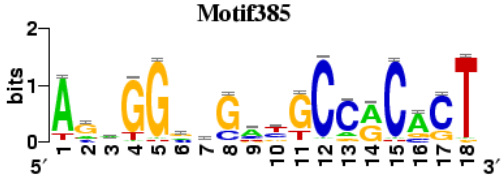 Motif385	237.13	0.8	0.167	0.001
		460.42	0.65	0.319	0.003

20	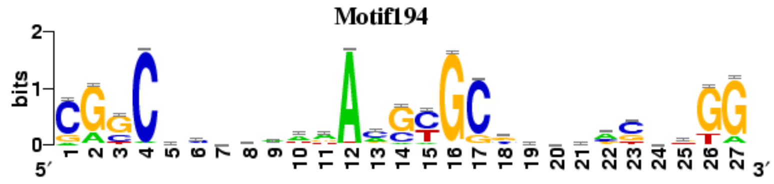				
	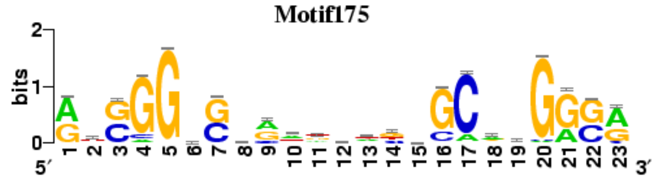	444.25	0.8	0.298	0.002
	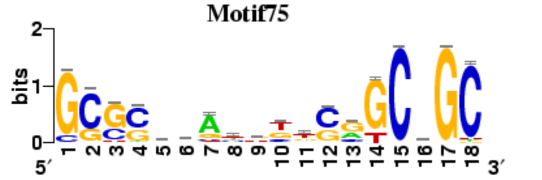	366.01	0.8	0.340	0.006
	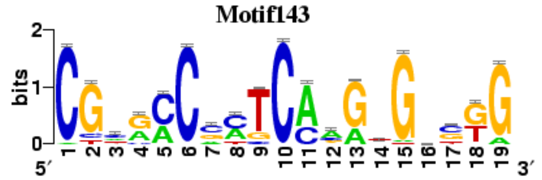	360.97	0.55	0.404	0.011
	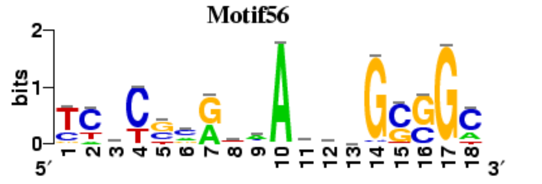	341.71	0.65	0.511	0.022
21	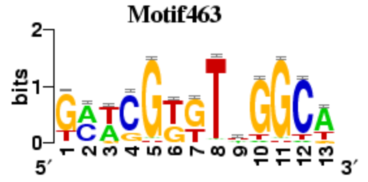	242.89	0.75	0.217	0.003
	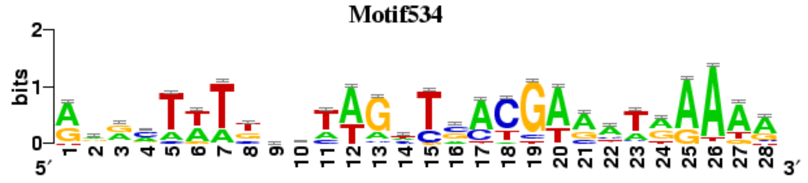	241.31	0.7	0.196	0.002
	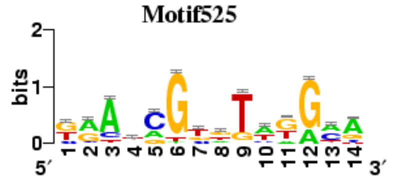	200.82	0.85	0.174	0.002
		46.00	0.95	0.043	0.000

	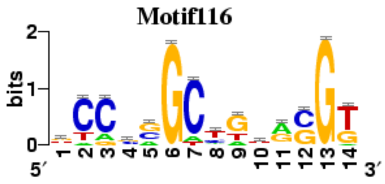				
	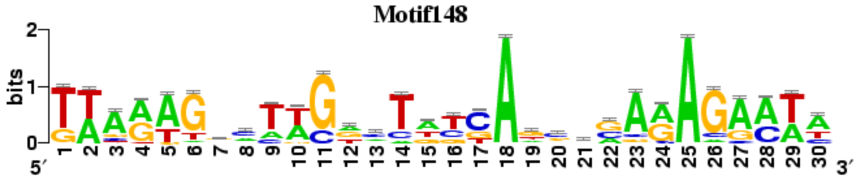	46.00	0.95	0.043	0.000
22	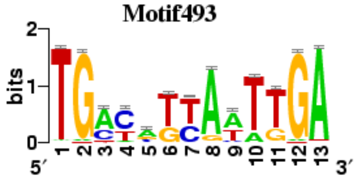	209.79	0.85	0.200	0.003
	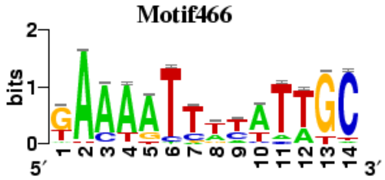	83.82	0.75	0.200	0.012
	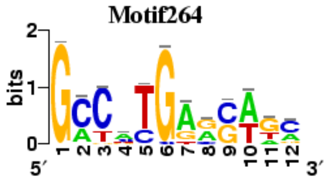	79.78	0.8	0.178	0.010
	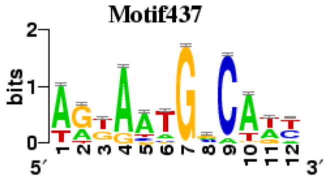	73.02	0.65	0.556	0.119
	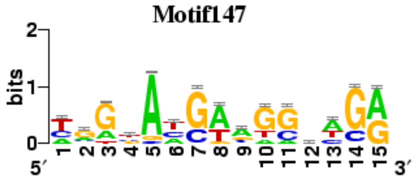	47.09	0.95	0.044	0.000
	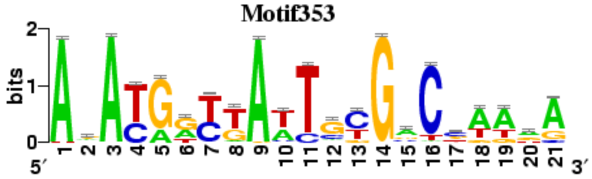	414.31	0.55	0.311	0.003
		270.96	0.75	0.200	0.002

23	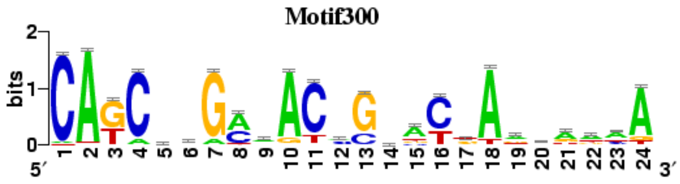				
	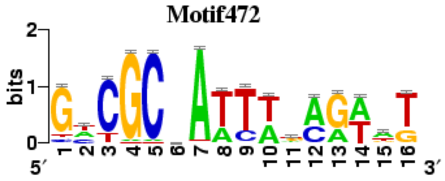	204.69	0.75	0.222	0.005
	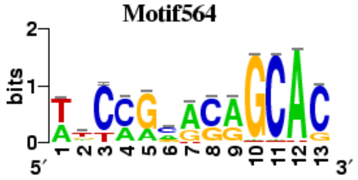	187.50	0.85	0.178	0.003
	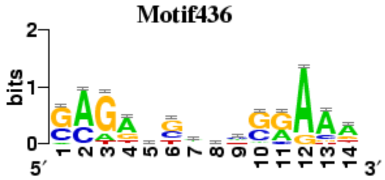	115.06	0.9	0.156	0.004
24	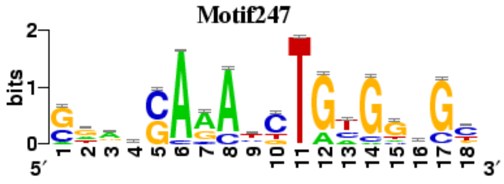	473.03	0.85	0.302	0.002
	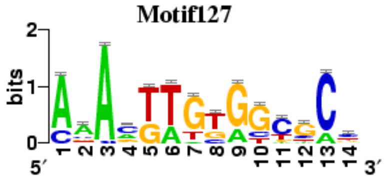	369.72	0.8	0.279	0.003
	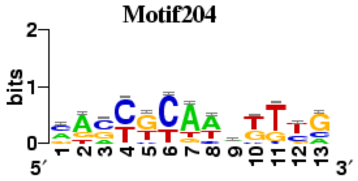	302.09	0.85	0.186	0.001
	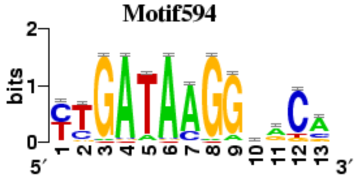	284.84	0.8	0.256	0.004
		267.10	0.85	0.186	0.001

	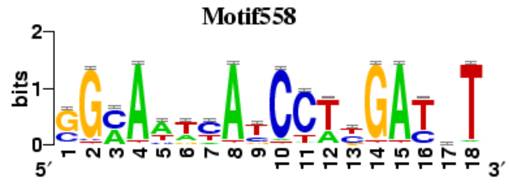				
25	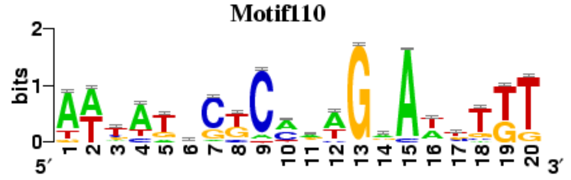	288.75	0.75	0.238	0.003
	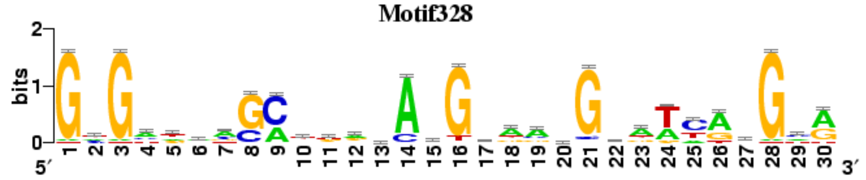	266.51	0.85	0.214	0.002
	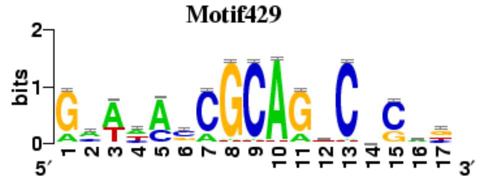	266.51	0.8	0.214	0.002
	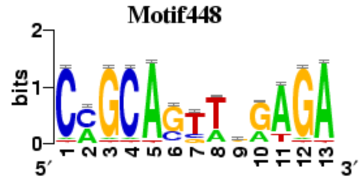	178.74	0.85	0.143	0.001
	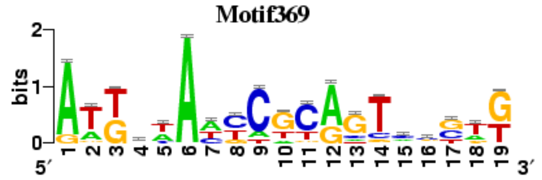	175.51	0.65	0.357	0.019
26	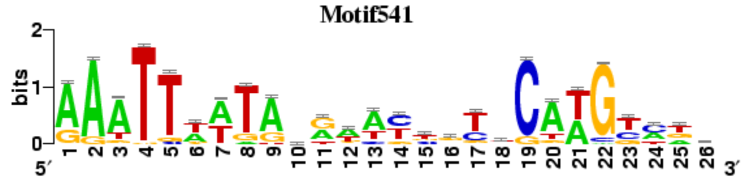	253.31	0.85	0.205	0.002
	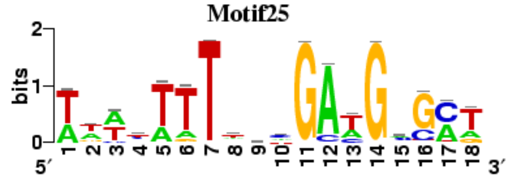	176.63	0.7	0.477	0.038
		107.19	0.95	0.091	0.001

	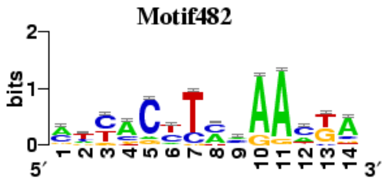				
	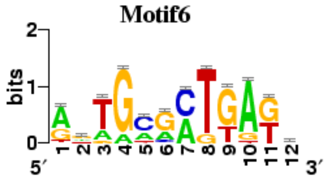	95.36	0.75	0.591	0.107
	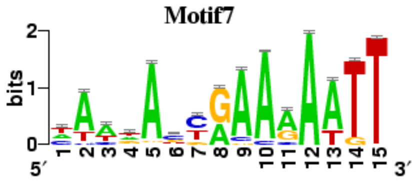	87.72	0.5	0.841	0.231
	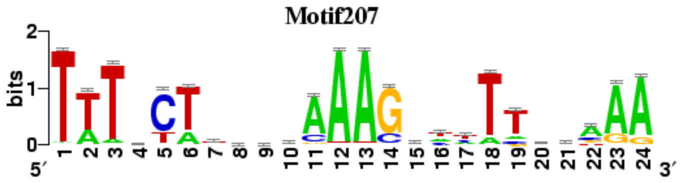	318.38	0.75	0.273	0.004
	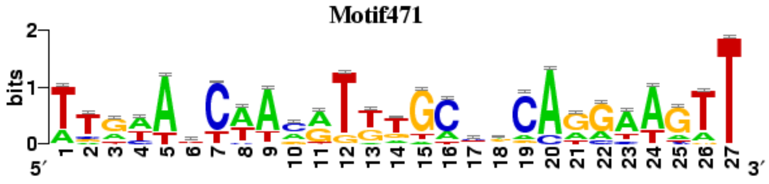	306.86	0.65	0.205	0.001
27	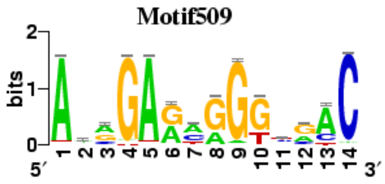	233.20	0.85	0.182	0.002
	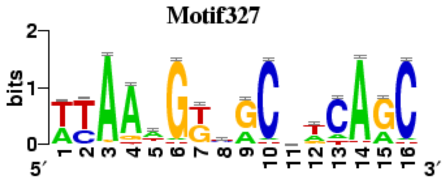	210.83	0.75	0.182	0.002
	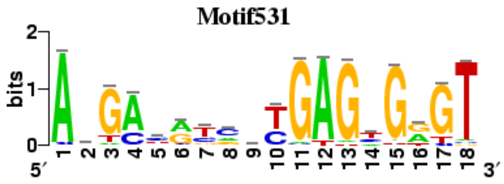	199.77	0.65	0.205	0.004
		382.33	0.8	0.535	0.019

28	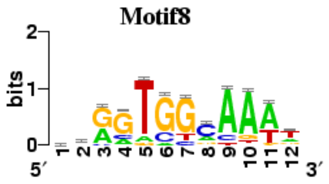				
	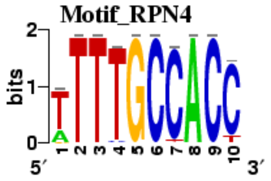	325.96	0.85	0.372	0.009
	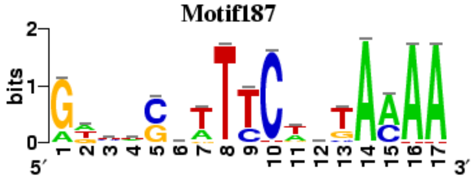	204.92	0.9	0.209	0.004
	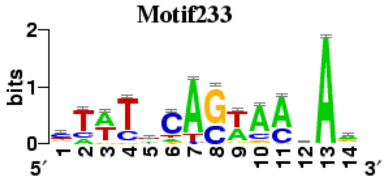	150.61	0.85	0.233	0.008
	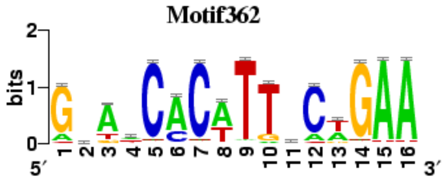	133.44	0.75	0.140	0.002
29	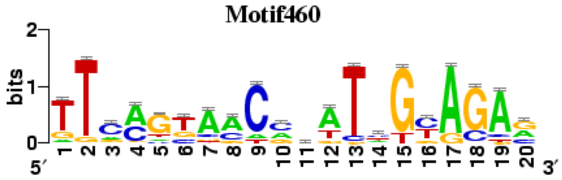	91.44	0.8	0.098	0.001
	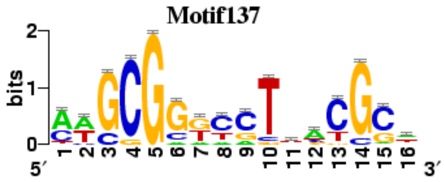	52.03	0.95	0.049	0.000
	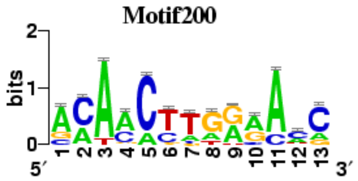	52.03	0.95	0.049	0.000
		41.00	0.75	0.463	0.119

	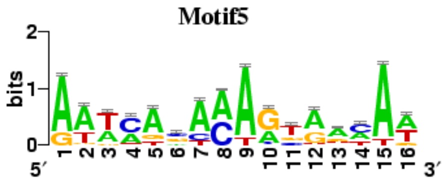				
	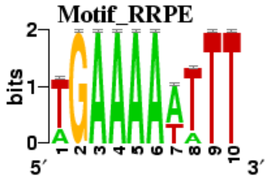	36.50	0.8	0.537	0.168
**30**	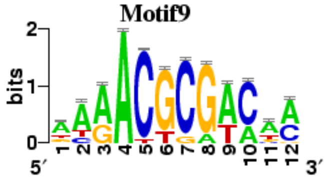	553.36	0.75	0.610	0.015
	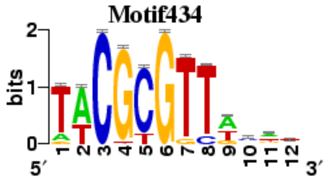	477.27	0.7	0.829	0.040
	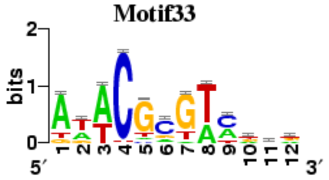	432.27	0.85	0.439	0.009
	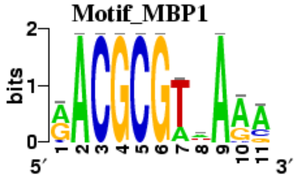	362.81	0.7	0.512	0.018
	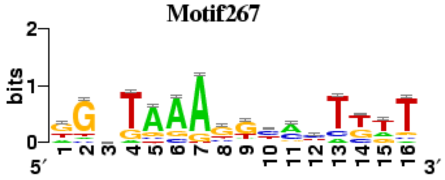	299.79	0.85	0.220	0.002
	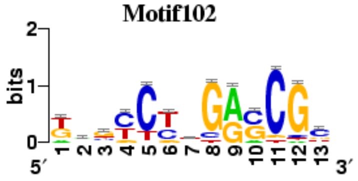	52.03	0.95	0.049	0.000
		52.03	0.8	0.049	0.000

	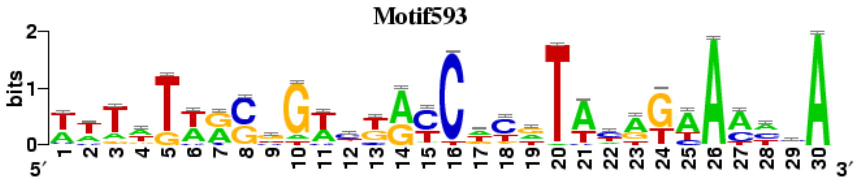				
	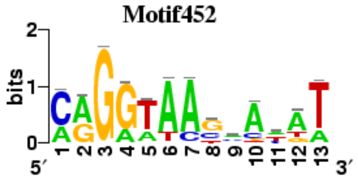	32.32	0.7	0.317	0.069
31	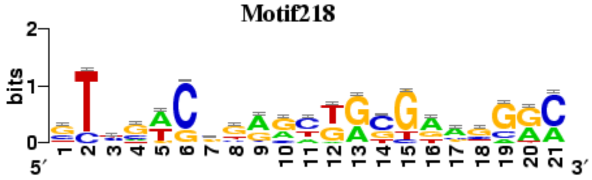	32.14	0.85	0.073	0.002
	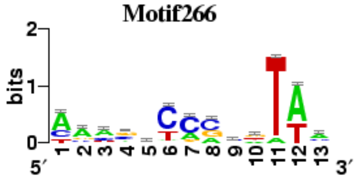	25.03	0.95	0.049	0.001
	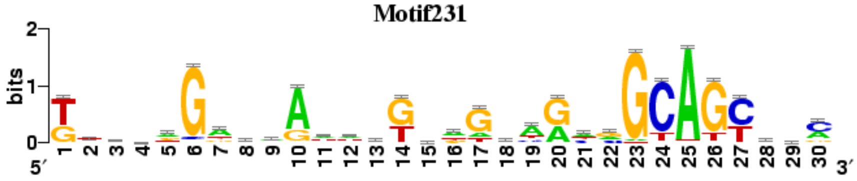	462.59	0.7	0.371	0.004
	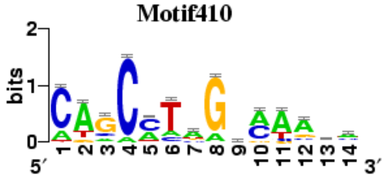	407.22	0.85	0.314	0.003
32	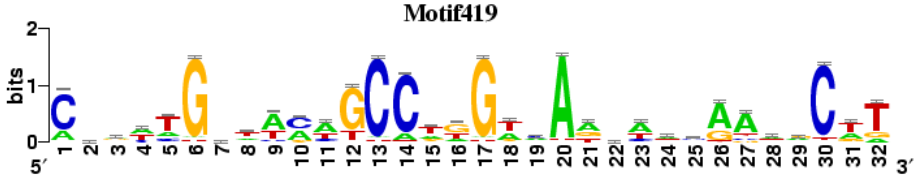	393.18	0.75	0.257	0.001
	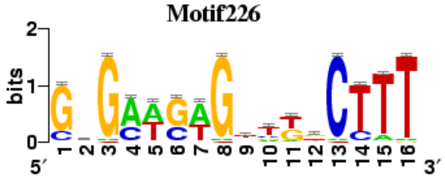	356.16	0.8	0.257	0.002
		276.83	0.8	0.257	0.003

	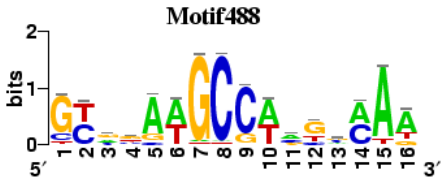				
	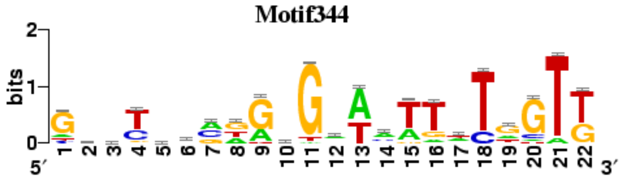	461.30	0.85	0.242	0.000
	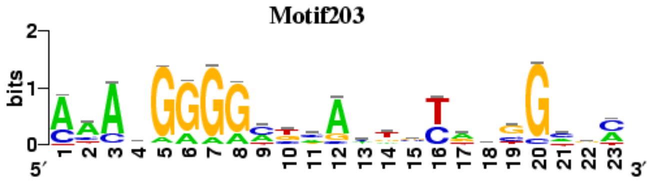	376.14	0.7	0.303	0.003
33	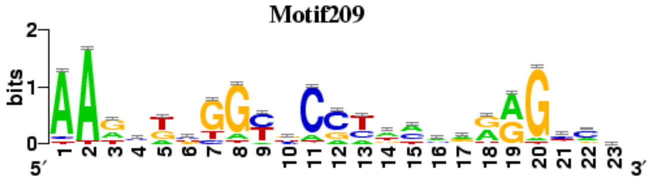	346.98	0.85	0.273	0.002
	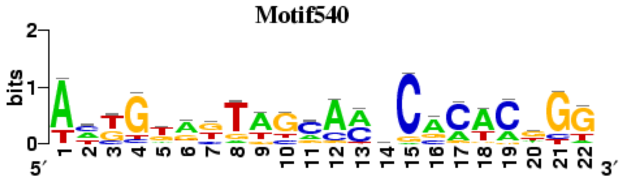	337.20	0.85	0.212	0.001
	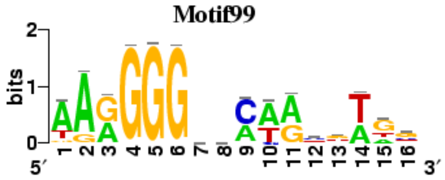	272.78	0.95	0.182	0.001
	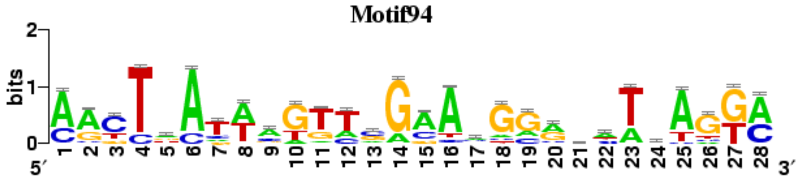	477.78	0.55	0.353	0.003
34	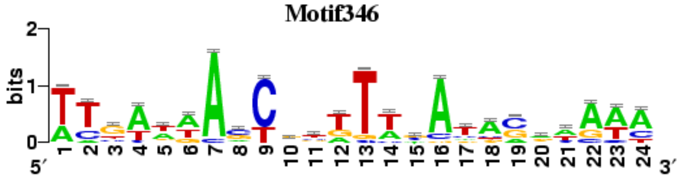	367.61	0.9	0.265	0.002
		338.70	0.95	0.294	0.003

	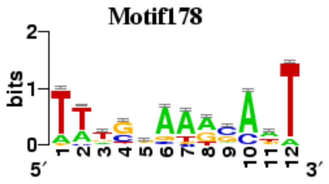				
	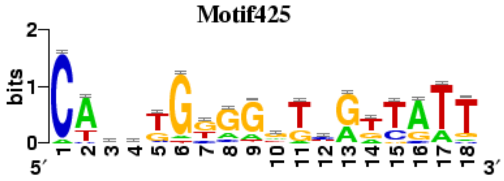	265.85	0.8	0.265	0.004
	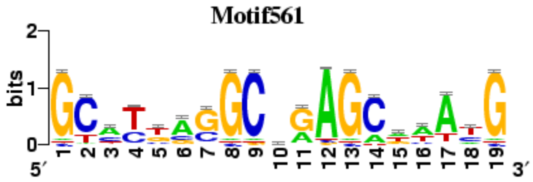	264.08	0.85	0.176	0.001
35	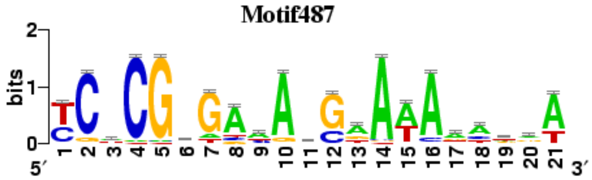	356.02	0.85	0.242	0.001
	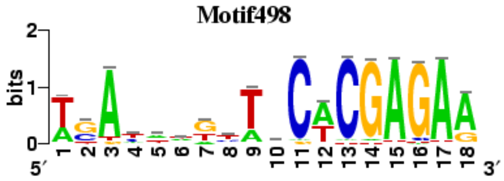	242.66	0.8	0.242	0.003
	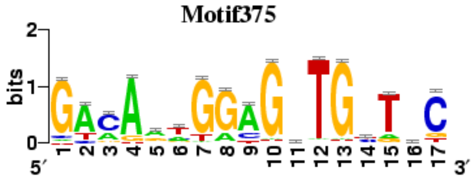	240.84	0.75	0.273	0.005
	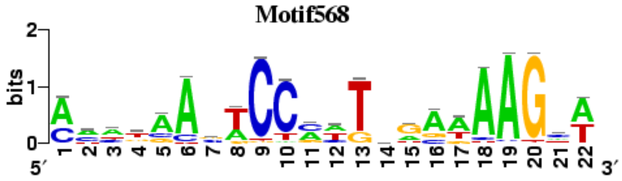	178.80	0.75	0.182	0.002
	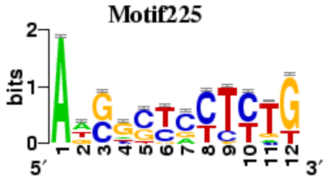	111.60	0.7	0.576	0.070
		217.96	0.9	0.219	0.003

36	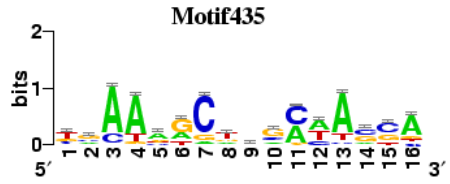				
	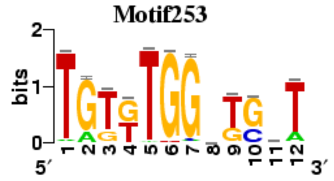	130.52	0.85	0.281	0.012
	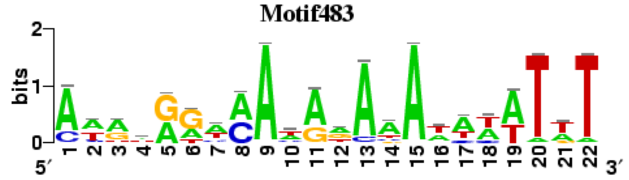	70.37	0.9	0.188	0.009
	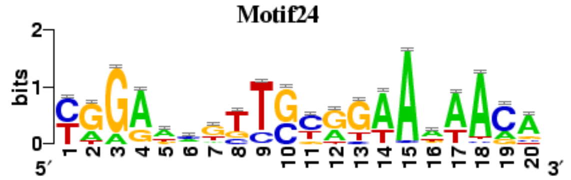	67.93	0.9	0.062	0.000
	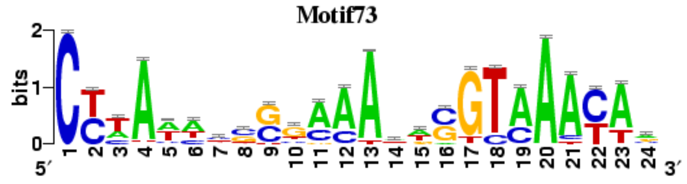	67.93	0.8	0.062	0.000
37	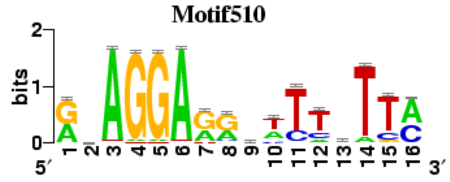	489.05	0.75	0.444	0.005
	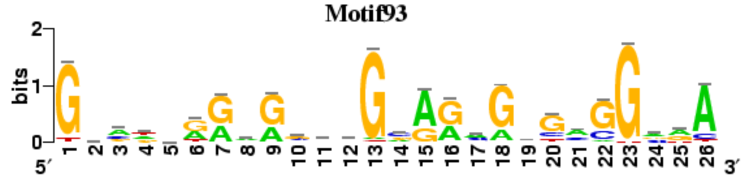	424.37	0.75	0.407	0.005
	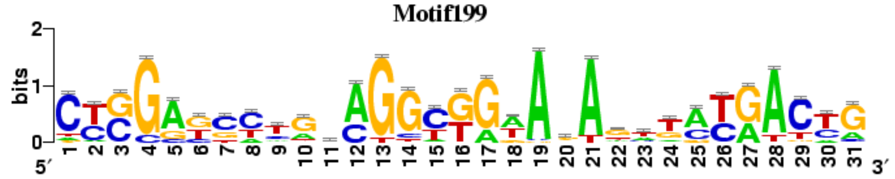	365.98	0.6	0.259	0.001
		327.35	0.8	0.185	0.000

	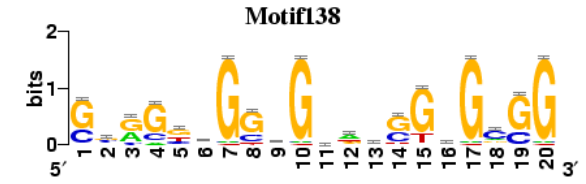				
	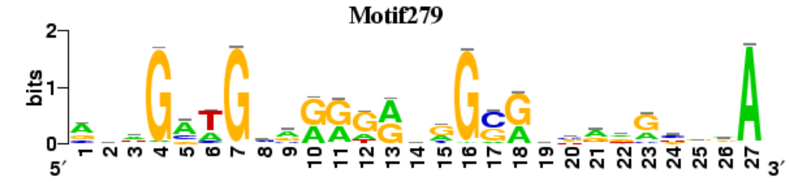	316.43	0.65	0.481	0.012
	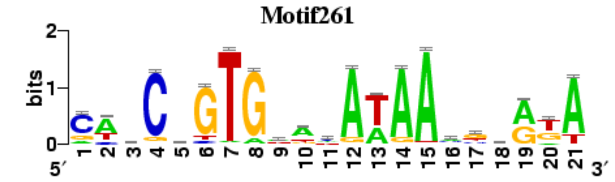	606.20	0.8	0.346	0.001
	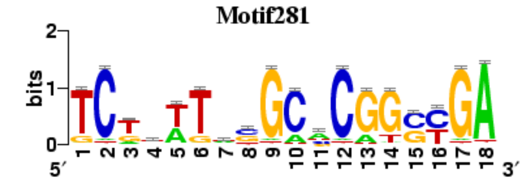	512.24	0.8	0.269	0.000
**38**	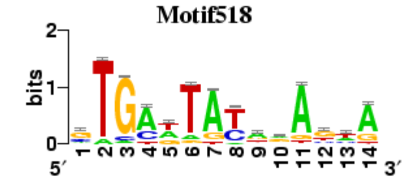	496.62	0.85	0.423	0.004
	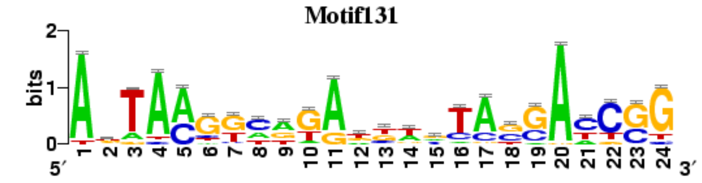	493.55	0.65	0.346	0.002
	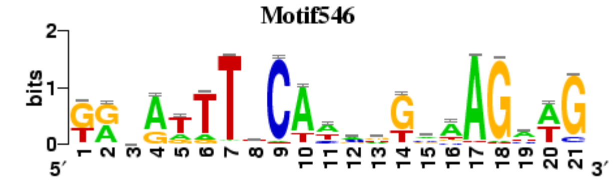	489.42	0.8	0.385	0.003
	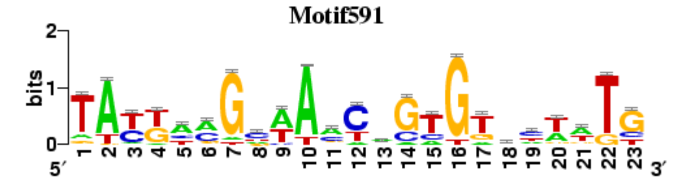	407.48	0.7	0.393	0.005
		393.45	0.85	0.214	0.000

39	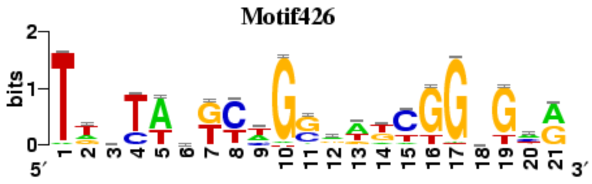				
	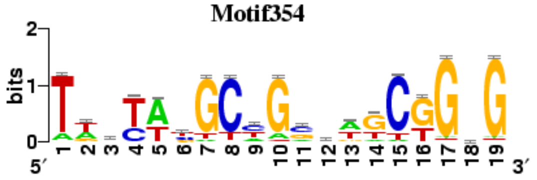	382.31	0.75	0.286	0.002
	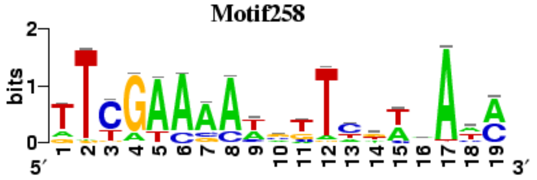	175.47	0.85	0.143	0.001
	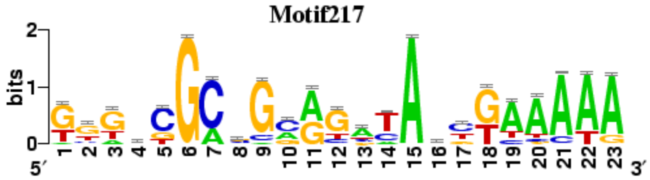	78.54	0.95	0.071	0.000
40	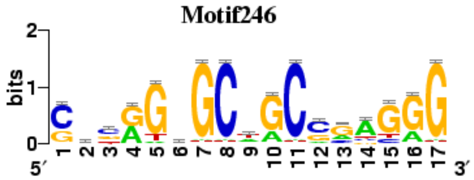	544.69	0.75	0.320	0.001
	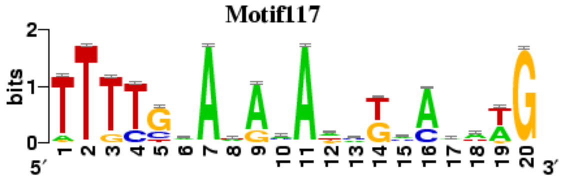	398.46	0.85	0.280	0.001
	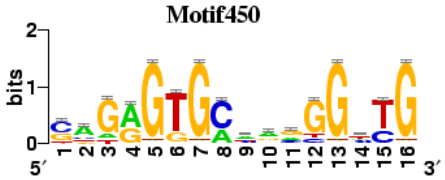	372.88	0.7	0.400	0.005
	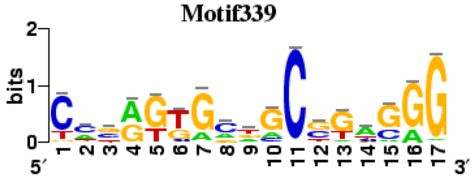	356.17	0.85	0.200	0.000
		154.25	0.85	0.200	0.003

	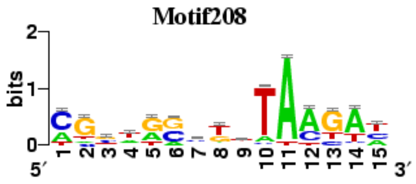				
41	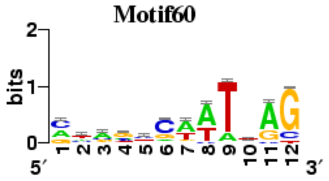	331.46	0.9	0.292	0.002
	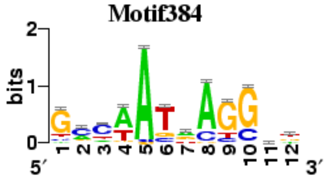	279.27	0.9	0.167	0.000
	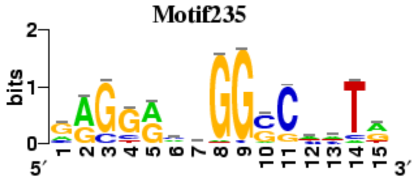	276.28	0.8	0.375	0.007
	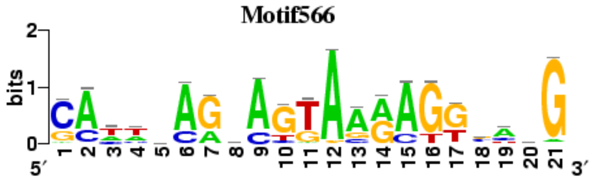	228.52	0.85	0.250	0.003
	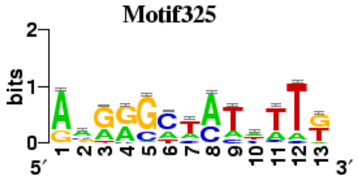	199.34	0.8	0.417	0.014
42	NA	NA	NA	NA	NA
43	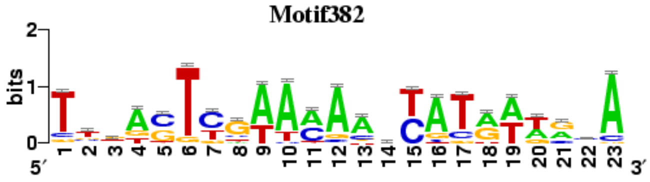	587.71	0.8	0.312	0.000
	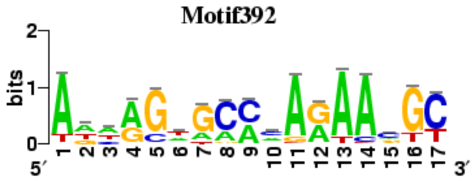	522.64	0.85	0.375	0.001
		440.57	0.95	0.250	0.000

	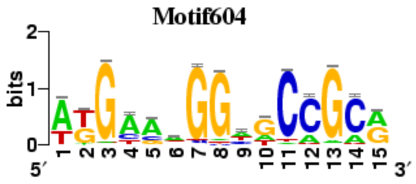				
	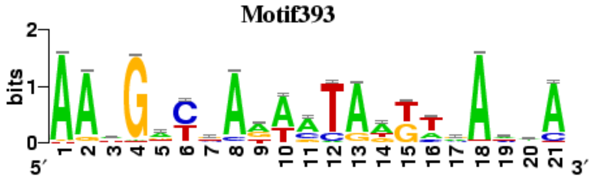	329.08	0.8	0.250	0.001
	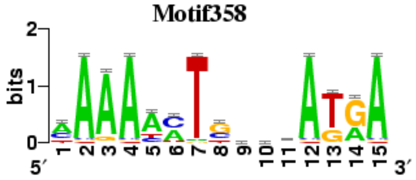	305.65	0.85	0.562	0.011
	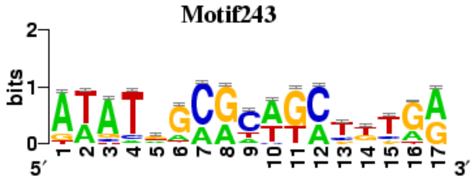	1936.87	0.8	0.941	0.001
	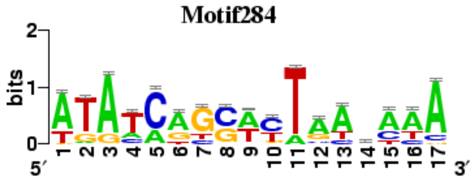	1798.71	0.75	0.882	0.001
44	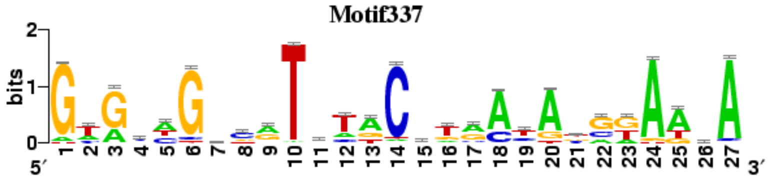	1798.71	0.7	0.882	0.001
	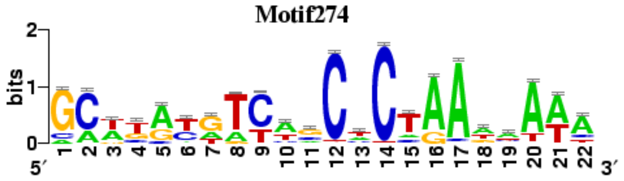	1685.35	0.65	0.882	0.001
	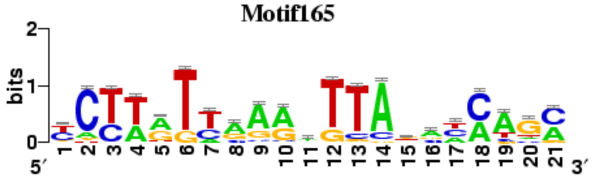	1660.76	0.75	0.824	0.001
		1225.81	0.5	0.688	0.001

	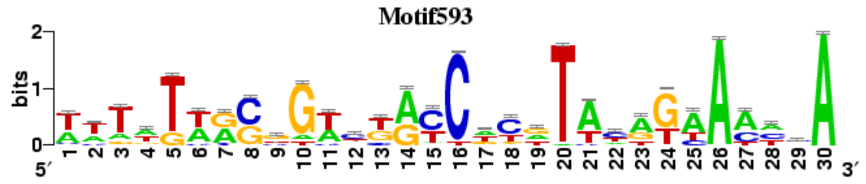				
	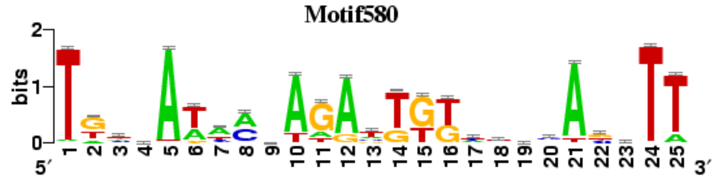	939.86	0.8	0.562	0.001
**45**	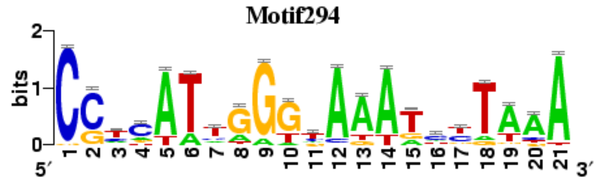	916.12	0.65	0.688	0.003
	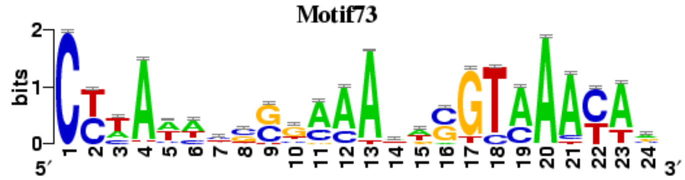	812.90	0.5	0.688	0.004
	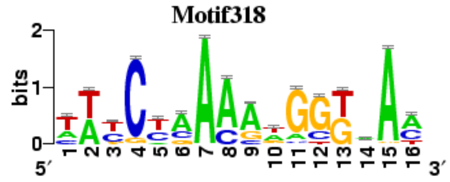	375.39	0.7	0.812	0.021
	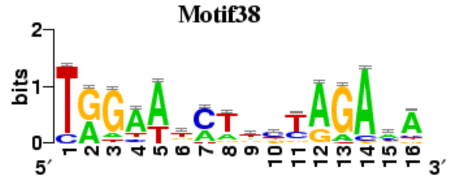	1051.23	0.85	0.571	0.001
**46**	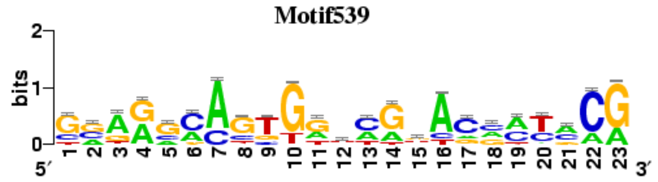	713.95	0.85	0.429	0.001
	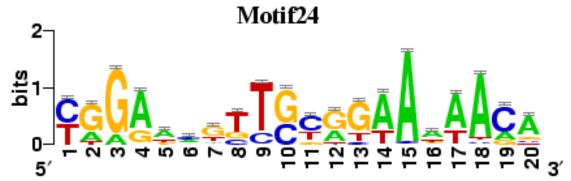	697.50	0.65	0.571	0.003
		533.48	0.75	0.429	0.002

	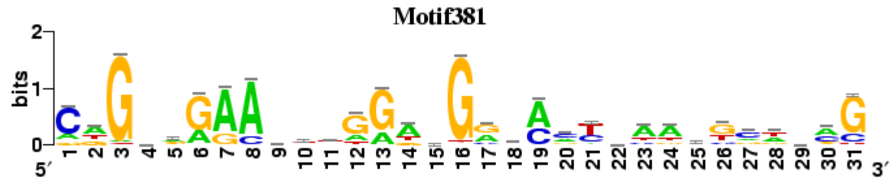				
	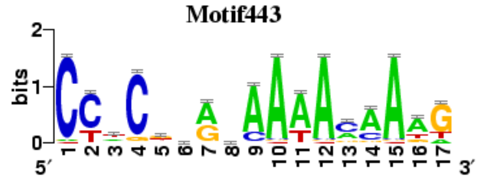	470.75	0.85	0.500	0.004
47	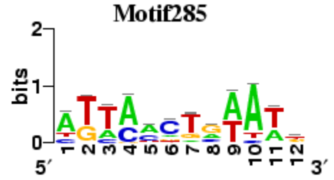	852.20	0.95	0.667	0.003
	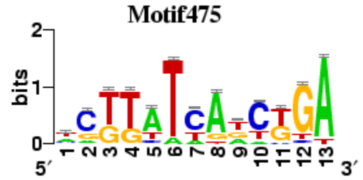	829.00	0.85	0.800	0.006
	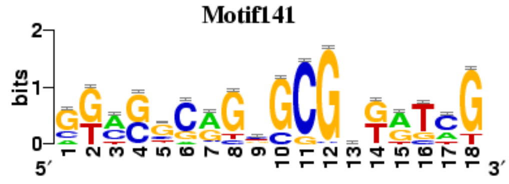	391.91	0.9	0.400	0.003
	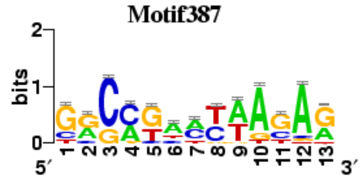	374.75	0.8	0.467	0.005
	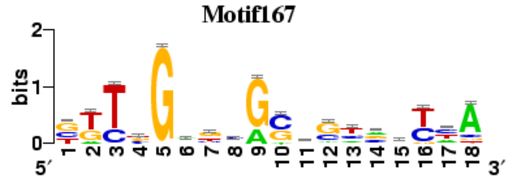	316.31	0.95	0.200	0.000
	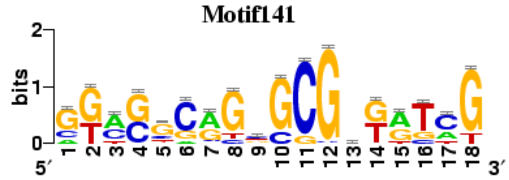	261.94	0.9	0.444	0.004
		165.82	0.65	0.667	0.019

48	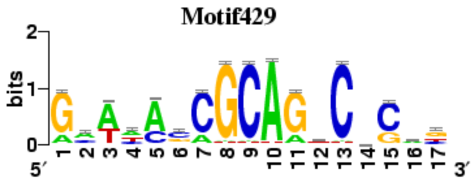				
	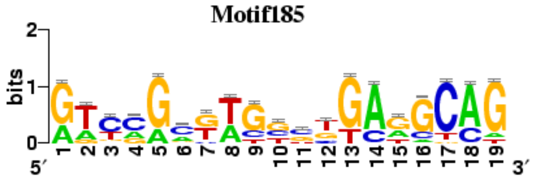	146.29	0.9	0.222	0.001
	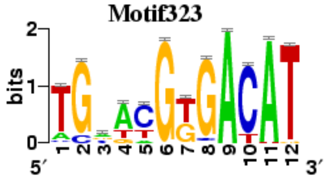	122.41	0.65	0.556	0.016
	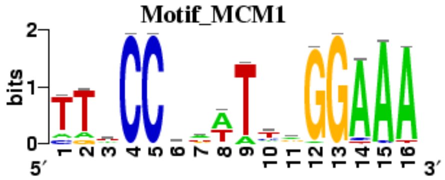	115.85	0.75	0.556	0.017
49	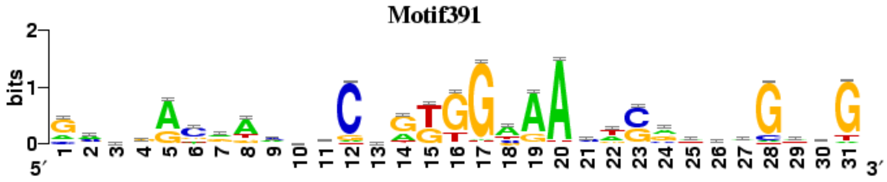	1404.27	0.8	0.727	0.001
	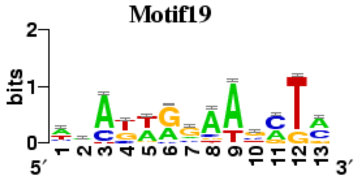	746.89	0.9	0.636	0.003
	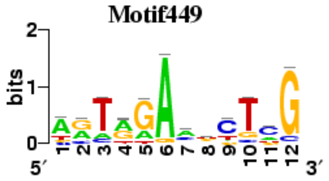	731.90	0.8	0.455	0.001
	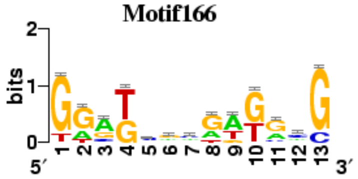	687.00	0.95	0.364	0.000
		291.57	0.95	0.364	0.002

	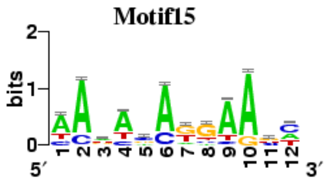Motif15<br>bits<br>5′<br>3′				
