# Supplementary material for: Predicting Gene Expression from Sequence: A Reexamination
Source: PLoS Comput Biol. 2007 Nov 30;3(11):e243. doi: 10.1371/journal.pcbi.0030243 (PMC2098866; doi:10.1371/journal.pcbi.0030243)
Supplement: Table S1 — (126 KB DOC) [file pcbi.0030243.st001.doc]

**Table S1.** Classification accuracy of 49 clusters using top 5/20 motifs in each cluster.

| Expression Pattern | Number of Genes | | Fraction | Number of Genes | | Fraction |
| --- | --- | --- | --- | --- | --- | --- |
|  | In Training Sets | Correctly Predicted  (5/20 motifs) | Correctly Predicted (5/20 motifs) | In Test Sets | Correctly Predicted (5/20 motifs) | Correctly Predicted (5/20 motifs) |
| 1 | 124 | 110/110 | 0.89/0.89 | 124 | 107/109 | 0.86/0.88 |
| 2 | 113 | 84/79 | 0.74/0.70 | 113 | 86/74 | 0.76/0.65 |
| 3 | 107 | 81/85 | 0.76/0.79 | 107 | 74/80 | 0.69/0.75 |
| 4 | 105 | 95/97 | 0.90/0.92 | 105 | 91/98 | 0.87/0.93 |
| 5 | 84 | 61/66 | 0.73/0.79 | 84 | 60/66 | 0.71/0.79 |
| 6 | 84 | 70/71 | 0.83/0.85 | 84 | 73/71 | 0.87/0.85 |
| 7 | 82 | 65/70 | 0.79/0.85 | 82 | 59/67 | 0.72/0.82 |
| 8 | 80 | 61/59 | 0.76/0.74 | 80 | 59/58 | 0.74/0.73 |
| 9 | 76 | 57/57 | 0.75/0.75 | 76 | 53/52 | 0.70/0.68 |
| 10 | 68 | 58/54 | 0.85/0.79 | 68 | 57/54 | 0.84/0.79 |
| 11 | 74 | 56/56 | 0.76/0.76 | 74 | 48/55 | 0.65/0.74 |
| 12 | 67 | 51/58 | 0.76/0.87 | 67 | 40/57 | 0.60/0.85 |
| 13 | 69 | 56/57 | 0.81/0.83 | 69 | 53/54 | 0.77/0.78 |
| 14 | 69 | 54/53 | 0.78/0.77 | 69 | 51/57 | 0.74/0.83 |
| 15 | 64 | 55/56 | 0.86/0.88 | 64 | 53/55 | 0.83/0.86 |
| 16 | 67 | 50/57 | 0.75/0.85 | 67 | 52/59 | 0.78/0.88 |
| 17 | 64 | 57/57 | 0.89/0.89 | 64 | 54/56 | 0.84/0.88 |
| 18 | 57 | 50/51 | 0.88/0.89 | 57 | 48/53 | 0.84/0.93 |
| 19 | 58 | 37/48 | 0.64/0.83 | 58 | 36/48 | 0.62/0.83 |
| 20 | 56 | 44/46 | 0.79/0.82 | 56 | 41/46 | 0.73/0.82 |
| 21 | 55 | 32/42 | 0.58/0.76 | 55 | 29/40 | 0.53/0.73 |
| 22 | 53 | 36/36 | 0.68/0.68 | 53 | 35/34 | 0.66/0.64 |
| 23 | 54 | 35/35 | 0.65/0.65 | 54 | 36/37 | 0.67/0.69 |
| 24 | 51 | 40/45 | 0.78/0.88 | 51 | 37/43 | 0.73/0.84 |
| 25 | 50 | 37/35 | 0.74/0.70 | 50 | 36/31 | 0.72/0.62 |
| 26 | 53 | 47/48 | 0.89/0.91 | 53 | 46/47 | 0.87/0.89 |
| 27 | 53 | 38/40 | 0.72/0.75 | 53 | 38/41 | 0.72/0.77 |
| 28 | 52 | 40/39 | 0.77/0.75 | 52 | 36/37 | 0.69/0.71 |
| 29 | 49 | 40/43 | 0.82/0.88 | 49 | 41/46 | 0.84/0.94 |
| 30 | 49 | 45/44 | 0.92/0.90 | 49 | 44/43 | 0.90/0.88 |
| 31 | 48 | 33/32 | 0.69/0.67 | 48 | 34/31 | 0.71/0.65 |
| 32 | 42 | 33/34 | 0.79/0.81 | 42 | 31/33 | 0.74/0.79 |
| 33 | 39 | 27/30 | 0.69/0.77 | 39 | 27/30 | 0.69/0.77 |
| 34 | 40 | 23/16 | 0.58/0.40 | 40 | 19/17 | 0.48/0.43 |
| 35 | 39 | 26/34 | 0.67/0.87 | 39 | 23/32 | 0.59/0.82 |
| 36 | 37 | 26/28 | 0.70/0.76 | 37 | 27/28 | 0.73/0.76 |
| 37 | 31 | 26/27 | 0.84/0.87 | 31 | 25/26 | 0.81/0.84 |
| 38 | 31 | 26/29 | 0.84/0.94 | 31 | 24/28 | 0.77/0.90 |
| 39 | 32 | 21/24 | 0.66/0.75 | 32 | 21/25 | 0.66/0.78 |
| 40 | 29 | 25/26 | 0.86/0.90 | 29 | 23/24 | 0.79/0.83 |
| 41 | 27 | 20/21 | 0.74/0.78 | 27 | 16/20 | 0.59/0.74 |
| 42 | 1 | 0/0 | 0.00/0.00 | 1 | 0/0 | 0.00/0.00 |
| 43 | 18 | 14/16 | 0.78/0.89 | 18 | 13/14 | 0.72/0.78 |
| 44 | 18 | 18/17 | 1.00/0.94 | 18 | 17/17 | 0.94/0.94 |
| 45 | 18 | 14/14 | 0.78/0.78 | 18 | 14/13 | 0.78/0.72 |
| 46 | 15 | 14/15 | 0.93/1.00 | 15 | 12/14 | 0.80/0.93 |
| 47 | 16 | 13/13 | 0.81/0.81 | 16 | 13/13 | 0.81/0.81 |
| 48 | 8 | 8/8 | 1.00/1.00 | 8 | 8/8 | 1.00/1.00 |
| 49 | 11 | 10/9 | 0.91/0.82 | 11 | 8/9 | 0.73/0.82 |
|  | 2587 | 2019/2087 | 0.78/0.81 | 2587 | 1928/2050 | 0.75/0.79 |
